# Supplementary material for: Natural variation of the streptococcal group A carbohydrate biosynthesis genes impacts host–pathogen interaction
Source: Microb Genom. 2025 Jul 17;11(7):001443. doi: 10.1099/mgen.0.001443 (PMC12282279; doi:10.1099/mgen.0.001443)
Supplement: Uncited Supplementary Material 1. [file mgen-11-01443-s001.pdf]

Natural variation of the streptococcal Group A carbohydrate biosynthesis genes impacts host-pathogen interaction

Kim Schipper[1*], Sara M. Tamminga[1*], Nicholas Murner[2], Matthew Davies[1], Paul Berkhout[1], Debra E. Bessen[4], Astrid Hendriks[1], Natalia Korotkova[2,3], Yvonne Pannekoek[1] and Nina M. van Sorge[1,5,#]

Supplemental information

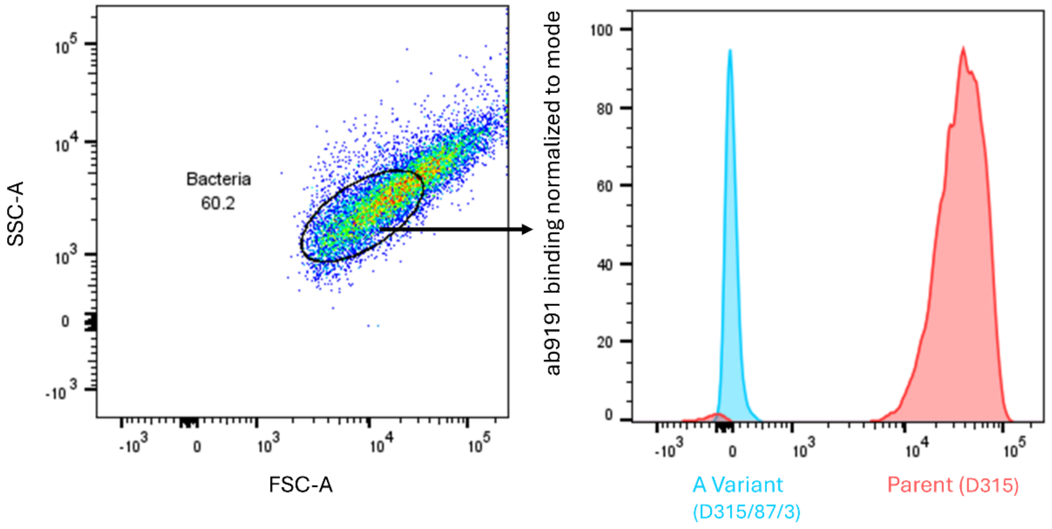

Figure S1 Gating strategy for ab9191 binding to *S. pyogenes* parent and A-variant strain

Representable forward- and side scatter with bacteria gate (left), and histograms of ab9191 (protein G-AF488) binding to A-variant (D315/87/3, animal-passaged; turquoise) strain and the parent (D315, wild-type; red) strain (right).

Table S1. Bacterial strains and plasmids used in this study.

Strain	Source	Information
S. pyogenes (M3)	(1)	
S. pyogenes (M3) Δ*gacI*	(1)	
S. pyogenes D315 (M58) (NCTC10876; R67/3884)	(2, 3). Obtained from UK Health Security Agency	Wild type
S. pyogenes D315/87/3	(4). Provided by Dr. D. Bessen, New York Medical College	'A variant', passaged in mice
E. coli MC1061 pDCerm_*gacH*	(5)	
S. pyogenes 5448	(5)	
S. pyogenes 5448 Δ*gacH*	(5)	
S. pyogenes 5448 Δ*gacH* + pDCerm_*gacH*	(5)	*gacH* from 5448
S. pyogenes 5448 Δ*gacH* + pDCerm_*gacH*_STOP309	This study	
S. pyogenes 5448 Δ*gacH* + pDCerm_*gacH*_STOP312	This study	
S. pyogenes 5448 Δ*gacH* + pDCerm_*gacH*_STOP773	This study	*gacH* from 20162146
S. pyogenes 20162146	CDC, (6)	Wildtype but *gacH* stop codon at position 773

Table S2. Primers used in this study

Primer	Sequence (5' to 3')
STOP309AAF	CCGGAATTCATGATTAAAGATACTTTCCTTAAGACTAAC
STOP309AAR	GAAGATCTTCAGCGTGAGATTTTAAAGAAGGTCTTGTG
gacH309_checkF1	CTTAGTTGCCTATTTCGCAATCG
gacH309_checkR1	CTCTCAGACGGATTGATCAATG
STOP312AAF	CCGGAATTCATGATTAAGGACACATTTTTAAAAACCAATTG
STOP312AAR	GAAGATCTTCAGCGTGAGATTTTAAAGAAGGTCTTGTG
STOP312checkF	GTGGCCTACTTTGCTATTG
STOP312checkR	GATCACGCTAAAGAATTGAG
gacHEcoRIF	CCGGAATTCATGATTAAAGACACATTTTTAAAAACCAAT
gacHBglIIR	GAAGATCTTTAACGTGATATCTTAAAAAAAGTTTTGTGT
gacHcheck1	CTGAATTAGTAACAGCAGGTAATAG
gacHcheck2	GCTAGGTACAAATCTTTAGC
gacHcheck3	GGTAGCTTGGTATCTACATG
pDCermF	GTACCGTTACTTATGAGCAAG
pDCermR	CACGGCATAAATCGCTCAG

References

1. Henningham A, Davies MR, Uchiyama S, van Sorge NM, Lund S, Chen KT, et al. Virulence Role of the GlcNAc Side Chain of the Lancefield Cell Wall Carbohydrate Antigen in Non-M1-Serotype Group A Streptococcus. mBio. 2018;9(1).

2. Parker MT, Bassett DC, Maxted WR, Arneaud JD. Acute glomerulonephritis in Trinidad: serological typing of group A streptococci. J Hyg (Lond). 1968;66(4):657-75.

3. Fox EN. M proteins of group A streptococci. Bacteriol Rev. 1974;38(1):57-86.

4. Scott JR, Pulliam WM, Hollingshead SK, Fischetti VA. Relationship of M protein genes in group A streptococci. Proc Natl Acad Sci U S A. 1985;82(6):1822-6.

5. Edgar RJ, van Hensbergen VP, Ruda A, Turner AG, Deng P, Le Breton Y, et al. Discovery of glycerol phosphate modification on streptococcal rhamnose polysaccharides. Nat Chem Biol. 2019;15(5):463-71.

6. Chochua S, Metcalf BJ, Li Z, Rivers J, Mathis S, Jackson D, et al. Population and Whole Genome Sequence Based Characterization of Invasive Group A Streptococci Recovered in the United States during 2015. mBio. 2017;8(5).

Supplementary Table S3. Overview of genetic changes between parent strain D315 and A-variant derivative D315/87/3

CHROM	POS	TYPE	REF	ALT	FTYPE	STRAND	EFFECT	Nucleotide substitution	Amino acid substitution	LOCUS_TAG	GENE	PRODUCT
LS483360	6371	snp	T	G	CDS	+	missense_variant	c.1366T>G	p.Leu456Val	NCTC10876_00006	mfd	transcription-repair coupling factor
LS483360	6585	snp	C	A	CDS	+	stop_gained	c.1580C>A	p.Ser527*	NCTC10876_00006	mfd	transcription-repair coupling factor
LS483360	45225	snp	C	A	CDS	+	missense_variant	c.1216C>A	p.Leu406Ile	NCTC10876_00059	purH	IMP cyclohydrolase / Phosphoribosylaminoimidazolecarboxamide formyltransferase
LS483360	49840	snp	C	A	CDS	+	stop_gained	c.828C>A	p.Cys276*	NCTC10876_00063	purK	phosphoribosylaminoimidazole carboxylase ATPase subunit
LS483360	59414	del	AAACCAGCTACTTGGATTG	A	CDS	+	disruptive_inframe_deletion	c.947_964delAACCAGCTACTTGGATTG	p.Lys316_Ala322delinsThr	NCTC10876_00072	adhE	Alcohol dehydrogenase
LS483360	59466	snp	C	A	CDS	+	missense_variant	c.998C>A	p.Thr333Asn	NCTC10876_00072	adhE	Alcohol dehydrogenase
LS483360	59485	snp	A	T	CDS	+	missense_variant	c.1017A>T	p.Glu339Asp	NCTC10876_00072	adhE	Alcohol dehydrogenase
LS483360	136470	snp	A	G	CDS	-	missense_variant	c.644T>C	p.Val215Ala	NCTC10876_00164		sensory box protein
LS483360	138536	snp	C	T	CDS	+	missense_variant	c.848C>T	p.Thr283Ile	NCTC10876_00166		Transcriptional regulator pfoR
LS483360	209033	snp	T	G	CDS	+	missense_variant	c.24T>G	p.Phe8Leu	NCTC10876_00235	radA	DNA repair protein RadA
LS483360	209059	snp	C	A	CDS	+	stop_gained	c.50C>A	p.Ser17*	NCTC10876_00235	radA	DNA repair protein RadA
LS483360	260275	ins	A	AT	Intergenic							
LS483360	278065	snp	A	C	CDS	+	missense_variant	c.208A>C	p.Ile70Leu	NCTC10876_00299	tig	trigger factor
LS483360	278079	snp	A	C	CDS	+	missense_variant	c.222A>C	p.Glu74Asp	NCTC10876_00299	tig	trigger factor
LS483360	278318	snp	T	G	CDS	+	missense_variant	c.461T>G	p.Ile154Ser	NCTC10876_00299	tig	trigger factor
LS483360	309642	snp	C	A	CDS	-	missense_variant	c.15G>T	p.Gln5His	NCTC10876_00332		hypothetical cytosolic protein
LS483360	311526	snp	A	C	CDS	-	missense_variant	c.1451T>G	p.Leu484Arg	NCTC10876_00335	pepX	xaa-Pro dipeptidyl-peptidase
LS483360	335469	snp	T	C	CDS	+	synonymous_variant	c.75T>C	p.Ala25Ala	NCTC10876_00357	ssb_2	single strand binding protein
LS483360	352450	snp	C	A	Intergenic							
LS483360	380388	snp	A	C	CDS	+	missense_variant	c.204A>C	p.Gln68His	NCTC10876_00392	yheD	cof-like hydrolase family protein
LS483360	381408	snp	A	C	CDS	+	missense_variant	c.1224A>C	p.Glu408Asp	NCTC10876_00392	yheD	cof-like hydrolase family protein
LS483360	383894	snp	T	G	CDS	+	stop_gained	c.84T>G	p.Tyr28*	NCTC10876_00395	codY	transcriptional repressor
LS483360	412882	snp	G	C	CDS	-	missense_variant	c.906C>G	p.Asp302Glu	NCTC10876_00428	manL	PTS system mannose-specific transporter subunit IIAB
LS483360	412894	snp	T	G	CDS	-	missense_variant	c.894A>C	p.Glu298Asp	NCTC10876_00428	manL	PTS system mannose-specific transporter subunit IIAB
LS483360	412925	del	GATAAAACGTTGTTAACCATTGTTTTAC	G	CDS	-	disruptive_inframe_deletion	c.836_862delGTAAAACAATGGTTAACAACGTTTTAT	p.Gly279_Ser288delinsAla	NCTC10876_00428	manL	PTS system mannose-specific transporter subunit IIAB
LS483360	413008	snp	A	C	CDS	-	synonymous_variant	c.780T>G	p.Val260Val	NCTC10876_00428	manL	PTS system mannose-specific transporter subunit IIAB
LS483360	425415	snp	A	C	CDS	+	stop_lost&splice_region_variant	c.1146A>C	p.Ter382Tyrext*?	NCTC10876_00442	nusA	transcription elongation factor
LS483360	425667	snp	C	T	CDS	+	synonymous_variant	c.237C>T	p.Tyr79Tyr	NCTC10876_00443		putative cytoplasmic protein
LS483360	426783	del	GCAAGA	G	CDS	+	frameshift_variant	c.748_752delCAAGA	p.Gln250fs	NCTC10876_00445	infB	translation initiation factor IF-2
LS483360	453667	snp	A	C	CDS	+	missense_variant	c.1094A>C	p.Tyr365Ser	NCTC10876_00474		Sodium-dependent phosphate transporter
LS483360	491565	snp	T	G	CDS	+	synonymous_variant	c.666T>G	p.Pro222Pro	NCTC10876_00507	pepC	aminopeptidase C
LS483360	503847	snp	C	A	CDS	-	missense_variant	c.1171G>T	p.Val391Leu	NCTC10876_00520	atoB	acetyl-CoA acetyltransferase
LS483360	503862	snp	C	A	CDS	-	stop_gained	c.1156G>T	p.Gly386*	NCTC10876_00520	atoB	acetyl-CoA acetyltransferase
LS483360	503876	snp	A	C	CDS	-	missense_variant	c.1142T>G	p.Ile381Ser	NCTC10876_00520	atoB	acetyl-CoA acetyltransferase
LS483360	522886	snp	A	C	CDS	+	missense_variant	c.488A>C	p.Gln163Pro	NCTC10876_00538		helicase conserved C-terminal domain protein
LS483360	589308	complex	TT	GG	CDS	+	missense_variant	c.382_383delTTinsGG	p.Leu128Gly	NCTC10876_00601		arginine/ornithine antiporter
LS483360	594572	snp	A	T	CDS	+	missense_variant	c.407A>T	p.Asn136Ile	NCTC10876_00605	rsmD	methyltransferase
LS483360	596827	snp	T	G	CDS	+	missense_variant	c.213T>G	p.Asn71Lys	NCTC10876_00608	rbsR	LacI family transcriptional regulator
LS483360	596835	snp	T	G	CDS	+	missense_variant	c.221T>G	p.Phe74Cys	NCTC10876_00608	rbsR	LacI family transcriptional regulator
LS483360	596855	snp	G	T	CDS	+	missense_variant	c.241G>T	p.Ala81Ser	NCTC10876_00608	rbsR	LacI family transcriptional regulator
LS483360	612145	snp	G	T	CDS	+	missense_variant	c.1279G>T	p.Asp427Tyr	NCTC10876_00623	ftsZ	cell division protein FtsZ
LS483360	618463	snp	A	C	Intergenic							
LS483360	632871	snp	A	T	CDS	+	missense_variant	c.545A>T	p.Glu182Val	NCTC10876_00644	recN	DNA repair protein recN
LS483360	648033	snp	A	C	CDS	+	missense_variant	c.719A>C	p.Gln240Pro	NCTC10876_00658	murF	UDP-N-acetylmuramoyl-tripeptide--D-alanyl-D- alanine ligase
LS483360	656645	snp	T	G	CDS	+	missense_variant	c.134T>G	p.Phe45Cys	NCTC10876_00665	plsC	1-acyl-sn-glycerol-3-phosphate acyltransferase
LS483360	659854	snp	G	T	CDS	+	missense_variant	c.1750G>T	p.Gly584Cys	NCTC10876_00667	comEC	DNA internalization-related competence protein ComEC/Rec2
LS483360	664616	del	TTTTCAAAGGCAATCCGCTGATAGTCTTTC	T	CDS	-	frameshift_variant	c.33_61delGAAAGACTATCAGCGGATTGCCTTTGAAA	p.Lys12fs	NCTC10876_00672		hypothetical cytosolic protein
LS483360	690332	snp	T	A	CDS	+	missense_variant	c.839T>A	p.Val280Glu	NCTC10876_00696	deadD	polysaccharide deacetylase family protein
LS483360	690337	snp	A	C	CDS	+	missense_variant	c.844A>C	p.Asn282His	NCTC10876_00696	deadD	polysaccharide deacetylase family protein
LS483360	694166	complex	CAAT	GCAG	CDS	+	missense_variant	c.129_132delCAATinsGCAG	p.Asn44Gln	NCTC10876_00701	msrC	GAF domain-containing protein
LS483360	694190	snp	C	G	CDS	+	stop_gained	c.153C>G	p.Tyr51*	NCTC10876_00701	msrC	GAF domain-containing protein
LS483360	694209	complex	TTA	GTC	CDS	+	missense_variant	c.172_174delTTAinsGTC	p.Leu58Val	NCTC10876_00701	msrC	GAF domain-containing protein
LS483360	694222	snp	C	A	CDS	+	missense_variant	c.185C>A	p.Pro62His	NCTC10876_00701	msrC	GAF domain-containing protein
LS483360	694234	snp	G	T	CDS	+	missense_variant	c.197G>T	p.Arg66Met	NCTC10876_00701	msrC	GAF domain-containing protein
LS483360	694368	snp	A	T	CDS	+	missense_variant	c.331A>T	p.Ile111Phe	NCTC10876_00701	msrC	GAF domain-containing protein
LS483360	694381	snp	T	G	CDS	+	missense_variant	c.344T>G	p.Met115Arg	NCTC10876_00701	msrC	GAF domain-containing protein
LS483360	707059	snp	G	T	CDS	+	missense_variant	c.553G>T	p.Ala185Ser	NCTC10876_00710	map	methionine aminopeptidase Map
LS483360	761506	snp	G	T	CDS	-	missense_variant	c.95C>A	p.Pro32Gln	NCTC10876_00758		phage protein
LS483360	765067	snp	T	G	CDS	-	missense_variant	c.658A>C	p.Lys220Gln	NCTC10876_00765		Uncharacterised protein
LS483360	765127	snp	C	A	CDS	-	missense_variant	c.598G>T	p.Asp200Tyr	NCTC10876_00765		Uncharacterised protein
LS483360	765158	snp	A	T	CDS	-	synonymous_variant	c.567T>A	p.Pro189Pro	NCTC10876_00765		Uncharacterised protein
LS483360	783367	complex	AGGGAAAG	CGGTAAAC	CDS	+	missense_variant	c.327_334delAGGGAAAGinsCGGTAAAC	p.GluGlyLysAsp109AspGlyLysHis	NCTC10876_00797		phage major capsid protein
LS483360	783387	snp	T	G	CDS	+	missense_variant	c.347T>G	p.Ile116Ser	NCTC10876_00797		phage major capsid protein
LS483360	799782	snp	T	A	CDS	-	missense_variant	c.453A>T	p.Lys151Asn	NCTC10876_00815	speC_1	exotoxin type C
LS483360	825197	snp	A	C	CDS	+	missense_variant	c.470A>C	p.Asn157Thr	NCTC10876_00840		response regulator
LS483360	825217	snp	A	C	CDS	+	missense_variant	c.490A>C	p.Asn164His	NCTC10876_00840		response regulator
LS483360	851134	ins	C	CA	CDS	+	frameshift_variant	c.486dupA	p.Gln163fs	NCTC10876_00867	rsmC	16S rRNA methyltransferase
LS483360	852521	snp	G	A	CDS	+	missense_variant	c.136G>A	p.Gly46Ser	NCTC10876_00869	tmpC	nucleoside-binding protein
LS483360	853529	snp	T	A	Intergenic							
LS483360	909790	snp	G	T	CDS	-	missense_variant	c.644C>A	p.Ser215Tyr	NCTC10876_00925	topA	DNA topoisomerase I
LS483360	912031	complex	TT	GA	CDS	-	missense_variant	c.202_203delAAinsTC	p.Lys68Ser	NCTC10876_00927	rnhB	ribonuclease HII
LS483360	923872	ins	C	CT	CDS	-	frameshift_variant	c.1052dupA	p.Thr352fs	NCTC10876_00940	msbA	ABC transporter
LS483360	930148	snp	T	C	CDS	-	missense_variant	c.398A>G	p.Lys133Arg	NCTC10876_00946	rimN	Sua5/YciO/YrdC/YwlC family protein
LS483360	930282	snp	A	C	CDS	-	synonymous_variant	c.264T>G	p.Ala88Ala	NCTC10876_00946	rimN	Sua5/YciO/YrdC/YwlC family protein
LS483360	930317	snp	A	T	CDS	-	missense_variant	c.229T>A	p.Phe77Ile	NCTC10876_00946	rimN	Sua5/YciO/YrdC/YwlC family protein
LS483360	930326	snp	A	C	CDS	-	missense_variant	c.220T>G	p.Tyr74Asp	NCTC10876_00946	rimN	Sua5/YciO/YrdC/YwlC family protein
LS483360	936059	snp	A	C	CDS	-	synonymous_variant	c.288T>G	p.Val96Val	NCTC10876_00953	xpt	xanthine phosphoribosyltransferase
LS483360	937718	snp	C	A	Intergenic							
LS483360	937907	snp	T	A	Intergenic							
LS483360	937923	snp	T	G	CDS	-	missense_variant	c.1516A>C	p.Ile506Leu	NCTC10876_00955	opuCB	putative ABC transporter
LS483360	961165	snp	T	G	CDS	+	missense_variant	c.1277T>G	p.Ile426Ser	NCTC10876_00977	dcuS	Sensor kinase dpiB
LS483360	977466	snp	T	G	CDS	-	missense_variant	c.182A>C	p.Glu61Ala	NCTC10876_00999	srtF	lantibiotic ABC transporter ATP-binding protein
LS483360	988940	snp	T	A	Intergenic							

LS483360	988946	complex	GAAT	CAAA	Intergenic							
LS483360	999963	snp	T	G	CDS	-	missense_variant	c.1073A>C	p.Lys358Thr	NCTC10876_01023	yehU	two-component sensor kinase
LS483360	1025744	del	TTTCA	T	CDS	-	frameshift_variant	c.51_54delTGAA	p.Glu18fs	NCTC10876_01044		dihydrolipoamide dehydrogenase
LS483360	1033389	snp	G	C	CDS	-	synonymous_variant	c.1530C>G	p.Gly510Gly	NCTC10876_01051		Zn-dependent hydrolase
LS483360	1059087	del	TTCTGACATATTAA	T	CDS	-	bidirectional_gene_fusion	n.1059100_1059088delTTAATATGTCAGA		NCTC10876_01082		phage protein
LS483360	1073224	snp	C	A	CDS	-	missense_variant	c.307G>T	p.Ala103Ser	NCTC10876_01109		phage protein
LS483360	1073289	snp	T	G	CDS	-	missense_variant	c.242A>C	p.Tyr81Ser	NCTC10876_01109		phage protein
LS483360	1073297	snp	A	C	CDS	-	synonymous_variant	c.234T>G	p.Arg78Arg	NCTC10876_01109		phage protein
LS483360	1081070	snp	T	G	CDS	-	missense_variant	c.68A>C	p.Lys23Thr	NCTC10876_01121	rmlA1	glucose-1-phosphate thymidyl transferase
LS483360	1086941	snp	T	C	CDS	-	missense_variant	c.1232A>G	p.His411Arg	NCTC10876_01128	recJ	single-stranded-DNA-specific exonuclease RecJ
LS483360	1111135	snp	G	T	CDS	-	synonymous_variant	c.579C>A	p.Val193Val	NCTC10876_01153	amiC	amidase
LS483360	1140514	snp	T	C	Intergenic							
LS483360	1150889	snp	G	T	Intergenic							
LS483360	1151554	del	GAGGA	G	CDS	-	frameshift_variant	c.1325_1328delTCCT	p.Ile442fs	NCTC10876_01194	fruA	PTS system fructose-specific transporter subunit IIABC
LS483360	1155810	snp	A	C	CDS	-	missense_variant	c.941T>G	p.Ile314Arg	NCTC10876_01198		regulatory protein
LS483360	1181380	snp	C	A	CDS	-	missense_variant	c.130G>T	p.Gly44Trp	NCTC10876_01220	oxyR	LysR family transcriptional regulator
**LS483360**	**1204515**	**snp**	**G**	**A**	**CDS**	**-**	**stop_gained**	**c.382C>T**	**p.Arg128***	**NCTC10876_01248**	**gacI**	**glycosyl transferase 2 family protein**
LS483360	1217554	snp	A	C	CDS	-	missense_variant	c.1471T>G	p.Leu491Val	NCTC10876_01259	dnaG_2	DNA primase
LS483360	1247252	snp	A	C	CDS	-	missense_variant	c.80T>G	p.Val27Gly	NCTC10876_01286		bacteriophage Gp15 family protein
LS483360	1282848	snp	G	T	CDS	-	stop_gained	c.1383C>A	p.Tyr461*	NCTC10876_01345	atpA	F0F1 ATP synthase subunit alpha
LS483360	1282869	snp	C	A	CDS	-	missense_variant	c.1362G>T	p.Leu454Phe	NCTC10876_01345	atpA	F0F1 ATP synthase subunit alpha
LS483360	1282881	snp	T	A	CDS	-	synonymous_variant	c.1350A>T	p.Val450Val	NCTC10876_01345	atpA	F0F1 ATP synthase subunit alpha
LS483360	1325779	snp	T	C	CDS	-	missense_variant	c.611A>G	p.Glu204Gly	NCTC10876_01378	agaS	tagatose-6-phosphate ketose/aldose isomerase
LS483360	1327354	snp	C	G	CDS	-	missense_variant	c.1471G>C	p.Glu491Gln	NCTC10876_01380	znuA	high-affinity zinc uptake system protein
LS483360	1327374	snp	C	G	CDS	-	missense_variant	c.1451G>C	p.Ser484Thr	NCTC10876_01380	znuA	high-affinity zinc uptake system protein
LS483360	1368803	snp	T	G	CDS	-	missense_variant	c.583A>C	p.Ile195Leu	NCTC10876_01415	ftsW	cell division protein
LS483360	1369087	snp	T	C	CDS	-	missense_variant	c.299A>G	p.Lys100Arg	NCTC10876_01415	ftsW	cell division protein
LS483360	1385438	snp	T	G	CDS	+	synonymous_variant	c.210T>G	p.Ala70Ala	NCTC10876_01431		membrane protein
LS483360	1409807	snp	A	T	CDS	-	missense_variant	c.2046T>A	p.Ser682Arg	NCTC10876_01454	mcdM	putative lantibiotic modifying protein
LS483360	1421524	snp	A	T	CDS	-	missense_variant	c.7T>A	p.Ser3Thr	NCTC10876_01468		huntington interacting protein HYPE
LS483360	1452470	ins	A	AT	CDS	-	frameshift_variant	c.41dupA	p.Asn14fs	NCTC10876_01499	drrA_3	antibiotic transport system ATP-binding protein
LS483360	1458388	snp	T	G	CDS	-	missense_variant	c.89A>C	p.Asn30Thr	NCTC10876_01504	ccpA	Catabolite control protein A
LS483360	1491975	snp	T	G	CDS	-	missense_variant	c.145A>C	p.Thr49Pro	NCTC10876_01545	frr	ribosome recycling factor
LS483360	1491986	snp	T	G	CDS	-	missense_variant	c.134A>C	p.Tyr45Ser	NCTC10876_01545	frr	ribosome recycling factor
LS483360	1495349	del	ATCCGCTTCAGCT	A	CDS	-	disruptive_inframe_deletion	c.2130_2141delAGCTGAAGCGGA	p.Gln710_Asp714delinsHis	NCTC10876_01550	ftsK_1	cell division protein FtsK
LS483360	1512152	snp	A	C	CDS	-	missense_variant	c.74T>G	p.Met25Arg	NCTC10876_01568		hypothetical membrane associated protein
LS483360	1514458	snp	T	G	Intergenic							
LS483360	1530113	snp	A	C	CDS	-	missense_variant	c.1022T>G	p.Ile341Ser	NCTC10876_01579		L-lactate oxidase
LS483360	1550073	snp	G	T	CDS	-	missense_variant	c.1193C>A	p.Pro398Gln	NCTC10876_01601	hlyX	hemolysin
LS483360	1561392	snp	A	C	Intergenic							
LS483360	1579923	snp	G	A	CDS	-	missense_variant	c.47C>T	p.Pro16Leu	NCTC10876_01634	csrS	HAMP domain-containing protein
LS483360	1611322	snp	T	G	CDS	+	missense_variant	c.2699T>G	p.Leu900Trp	NCTC10876_01665	hsdR	type I site-specific deoxyribonuclease, HsdR family protein
LS483360	1612597	del	ATTTACAAGGTGGCCAAGGAAATTTATCTGGT	A	CDS	+	frameshift_variant	c.985_1015delTTACAAGGTGGCCAAGGAAATTTATCTGGTT	p.Gln330fs	NCTC10876_01666	hsdS	Type I restriction-modification system specificity subunit
LS483360	1614774	del	AAGCT	A	CDS	-	frameshift_variant	c.7_10delAGCT	p.Ser3fs	NCTC10876_01668		Uncharacterised protein
LS483360	1621385	snp	A	G	CDS	-	synonymous_variant	c.543T>C	p.Ser181Ser	NCTC10876_01674		salivaricin A modification enzyme
LS483360	1630018	snp	A	C	CDS	+	missense_variant	c.432A>C	p.Leu144Phe	NCTC10876_01683	lacR_2	Lactose phosphotransferase system repressor
LS483360	1631598	snp	C	A	CDS	+	missense_variant	c.301C>A	p.Pro101Thr	NCTC10876_01685		Toxin to DNA-damage-inducible protein J
LS483360	1639544	snp	A	C	CDS	+	missense_variant	c.110A>C	p.Lys37Thr	NCTC10876_01694		integrase
LS483360	1647726	snp	A	G	CDS	-	missense_variant	c.166T>C	p.Tyr56His	NCTC10876_01705	cysS	cysteinyl-tRNA synthetase
LS483360	1654902	snp	T	G	CDS	-	missense_variant	c.1958A>C	p.Asp653Ala	NCTC10876_01713	ulaC_2	PTS system mannitol (cryptic)-specific transporter subunit IIA
LS483360	1654971	complex	ATCAA	CTCAC	CDS	-	missense_variant	c.1885_1889delTTGATinsGTGAG	p.LeuIle629ValSer	NCTC10876_01713	ulaC_2	PTS system mannitol (cryptic)-specific transporter subunit IIA
LS483360	1679350	snp	T	A	CDS	+	missense_variant	c.287T>A	p.Met96Lys	NCTC10876_01730	skc	streptokinase
LS483360	1714385	snp	T	G	CDS	-	missense_variant	c.556A>C	p.Ile186Leu	NCTC10876_01759	emm49	Antiphagocytic M protein
LS483360	1814605	snp	G	T	CDS	-	missense_variant	c.38C>A	p.Pro13His	NCTC10876_01863		tetratricopeptide repeat family protein
LS483360	1814621	snp	G	T	CDS	-	missense_variant	c.22C>A	p.Gln8Lys	NCTC10876_01863		tetratricopeptide repeat family protein
LS483360	1814646	snp	A	T	Intergenic							
LS483360	1854714	snp	C	A	CDS	+	missense_variant	c.31C>A	p.Leu11Met	NCTC10876_01902	cadD	cadmium resistance protein
LS483360	1897438	snp	A	C	CDS	+	missense_variant	c.465A>C	p.Glu155Asp	NCTC10876_01943	yheS_2	ABC transporter ATP-binding protein

Supplementary Table S4: Metadata of 2,021 S. pyogenes isolates screened in this study

Isolate	Country	Continent	Year	Primary_disease^	*Emm*	ST (MLST)	*gacA*[#]	*gacB*	*gacC*	*gacD*	*gacE*	*gacF*	*gacG*	*gacH*	*gacI*	*gacJ*	*gacK*	*gacL*
NS2403	Australia	Oceania	2000	APSGN	emm3.22	335	9	9	8	8	8	10	9	10	5	5	9	9
NS2413	Australia	Oceania	2000	skin/soft tissue infection, NOS	emm85.0	109	10	10	4	9	9	11	10	11	7	3	10	10
NS2985	Australia	Oceania	2001	APSGN	emm105.0	954	6	6	6	6	5	12	6	12	5	4	6	11
NS4729	Australia	Oceania	2005	APSGN	emm55.0	100	11	11	7	10	10	13	11	13	8	3	11	12
NS4972	Australia	Oceania	2005	APSGN	emm55.0	100	11	11	7	10	10	13	11	13	8	3	11	12
NS81	Australia	Oceania	1992	APSGN	emm55.0	100	11	11	7	10	10	13	11	13	8	3	11	12
NS2188	Australia	Oceania	2000	APSGN	emm49.3	190	4	8	4	11	11	14	12	14	9	2	12	13
GAS253	Australia	Oceania	ND	ND	emm19.7	370	12	12	4	12	12	13	13	15	8	3	13	14
NS5268	Australia	Oceania	2005	APSGN	emm55.0	100	11	11	7	10	10	13	11	13	8	3	11	12
NS426	Australia	Oceania	1995	APSGN	emm239.0	92	13	1	1	1	1	15	14	16	5	6	14	15
NS100	Australia	Oceania	1992	APSGN	emm68.2	298	14	13	9	8	13	13	15	17	10	2	15	16
NS6902	Australia	Oceania	ND	APSGN	emm49.0	371	4	8	4	13	14	14	16	18	9	2	12	13
NS3965	Australia	Oceania	ND	skin/soft tissue infection, NOS	emm49.3	190	4	8	4	14	11	14	12	19	9	2	12	17
Dog48	Australia	Oceania	1990	ND	emm91.0	12	11	5	10	15	15	16	17	13	1	2	16	18
NS27	Australia	Oceania	1991	APSGN	emm91.0	12	11	5	10	15	15	16	17	13	1	2	16	18
NS38	Australia	Oceania	1991	APSGN	emm57.2	33	11	14	11	8	16	17	18	20	11	7	17	19
NS5658	Australia	Oceania	2005	APSGN	emm55.0	100	11	11	7	10	10	13	11	13	8	3	11	12
NS76	Australia	Oceania	1992	APSGN	emm98.1	205	15	15	10	1	10	18	19	21	12	2	6	20
NS48	Australia	Oceania	1991	APSGN	emm57.2	33	11	14	11	8	16	17	18	20	11	7	17	19
SDog	Australia	Oceania	1990	ND	emm70.0	10	16	16	3	16	17	7	20	22	1	2	18	3
NS22	Australia	Oceania	1991	APSGN	emm57.2	33	11	14	11	8	16	17	18	20	11	7	17	19
YARB5	Australia	Oceania	2010	skin/soft tissue infection, NOS	emm52.1	180	15	17	10	17	18	18	21	23	12	8	19	21
NS2283	Australia	Oceania	2000	APSGN	emm3.22	335	9	9	8	8	8	10	9	10	5	5	9	9
NS2299	Australia	Oceania	2000	APSGN	emm3.22	335	9	9	8	8	8	10	9	10	5	5	9	9
NS2361	Australia	Oceania	2000	APSGN	emm70.0	10	16	16	3	16	17	7	20	22	1	2	18	3
YARC4	Australia	Oceania	2010	skin/soft tissue infection, NOS	emm92.0	82	17	4	3	18	19	19	22	24	13	2	20	22
YARG2	Australia	Oceania	2010	skin/soft tissue infection, NOS	emm11.0	ND	1	6	12	1	20	20	13	25	8	3	21	23
YARG4	Australia	Oceania	2010	skin/soft tissue infection, NOS	emm124.0	544	18	18	4	16	21	21	23	26	8	9	21	23
NS53	Australia	Oceania	1991	skin/soft tissue infection, NOS	emm71.0	318	19	[S]	13	19	2	22	3	27	1	1	4	24
NS564	Australia	Oceania	1995	invasive, NOS	emm44.0	178	5	7	7	3	2	3	24	8	14	3	7	7
NS696	Australia	Oceania	1996	pharyngitis and/or tonsillitis	emm1.0	1014	1	1	1	1	1	1	1	1	1	1	1	1
NS730	Australia	Oceania	1996	skin/soft tissue infection, NOS	emm90.5	1083	20	20	14	19	2	23	25	28	15	3	22	25
NS80	Australia	Oceania	1992	skin/soft tissue infection, NOS	emm70.0	1063	16	16	3	[S]	17	7	20	[S]	1	2	18	3
NS803	Australia	Oceania	1996	invasive, NOS	emm97.1	216	21	21	15	8	22	24	26	30	8	10	12	26
NS83	Australia	Oceania	1992	invasive, NOS	emm232.1	288	18	18	16	16	21	25	27	26	16	3	21	23
NS931	Australia	Oceania	1996	skin/soft tissue infection, NOS	emm65.0	1015	22	22	17	21	23	26	28	31	1	1	1	27
NS3	Australia	Oceania	1990	invasive, NOS	emm98.0	1076	15	15	10	1	[S]	18	19	21	12	2	6	20
NS101	Australia	Oceania	1992	skin/soft tissue infection, NOS	emm110.0	628	14	23	18	6	25	27	29	32	1	2	23	28
NS236	Australia	Oceania	1994	skin/soft tissue infection, NOS	emm77.0	63	23	24	19	1	26	28	30	33	8	3	24	29
NS240	Australia	Oceania	1995	skin/soft tissue infection, NOS	ND	166	14	25	3	22	27	13	26	29	8	3	25	30
NS50.1	Australia	Oceania	1991	skin/soft tissue infection, NOS	emm108.0	304	11	5	10	20	15	16	31	13	1	2	16	31
NS1033	Australia	Oceania	1997	ND	emm230.0	205	1	15	10	1	24	18	19	21	12	2	6	20
NS1122	Australia	Oceania	1997	pharyngitis and/or tonsillitis	emm63.3	297	24	19	3	23	28	7	32	34	17	11	26	32
NS1210	Australia	Oceania	1997	invasive, NOS	emm75.0	632	25	26	20	24	29	29	33	35	15	3	12	33
NS13	Australia	Oceania	1991	invasive, NOS	emm53.0	299	11	5	10	20	15	16	17	13	1	2	18	3

NS192	Australia	Oceania	1994	skin/soft tissue infection, NOS	emm106.0	338	26	27	21	25	30	19	34	36	15	3	27	34
NS195	Australia	Oceania	1994	skin/soft tissue infection, NOS	ND	370	12	12	4	12	12	13	13	15	8	3	13	14
NS20	Australia	Oceania	1991	skin/soft tissue infection, NOS	emm75.1	607	27	28	22	26	31	13	35	37	18	3	28	30
NS216	Australia	Oceania	1994	invasive, NOS	emm229.0	614	28	29	23	1	32	30	36	38	8	5	29	35
NS1	Australia	Oceania	1990	skin/soft tissue infection, NOS	emm100.0	119	11	11	7	10	10	7	37	39	19	2	30	31
NS1132	Australia	Oceania	1997	skin/soft tissue infection, NOS	emm25.1	192	14	30	24	2	33	13	13	40	20	3	31	36
NS1133	Australia	Oceania	1997	invasive, NOS	emm101.0	182	15	31	10	20	15	31	38	41	1	3	32	37
NS1140	Australia	Oceania	1997	skin/soft tissue infection, NOS	emm57.0	348	29	32	7	1	34	32	39	42	1	2	23	3
NS10	Australia	Oceania	1990	APSGN	emm53.0	299	11	5	10	20	15	16	17	13	1	2	18	3
NS1036	Australia	Oceania	1997	ND	emm110.0	179	14	33	18	6	25	27	40	43	1	2	18	38
NS1045	Australia	Oceania	1997	ND	emm60.7	193	14	34	25	27	2	33	41	44	1	2	33	39
NS1053	Australia	Oceania	1997	invasive, NOS	emm60.4	193	14	34	25	27	2	33	41	44	1	2	33	39
NS1096	Australia	Oceania	1997	skin/soft tissue infection, NOS	emm25.0	191	30	35	26	28	35	34	42	45	11	2	12	40
NS1107	Australia	Oceania	1997	skin/soft tissue infection, NOS	emm76.4	631	31	36	27	1	36	35	4	46	21	1	4	41
NS1017	Australia	Oceania	1997	skin/soft tissue infection, NOS	emm90.5	184	20	20	14	19	2	23	25	28	15	3	22	25
NS1120	Australia	Oceania	1997	skin/soft tissue infection, NOS	emm77.0	1016	23	24	19	1	26	28	30	33	8	3	24	29
NS196	Australia	Oceania	1994	ARF	emm58.0	176	14	13	10	29	13	24	15	47	10	2	34	31
NS204	Australia	Oceania	1994	invasive, NOS	emm2.0	55	32	26	28	24	29	13	43	48	15	3	12	42
NS210	Australia	Oceania	1994	invasive, NOS	emm22.0	202	33	11	7	10	35	36	44	49	19	2	30	43
NS1216	Australia	Oceania	1997	invasive, NOS	emm109.1	633	8	4	29	30	19	37	45	50	15	3	35	34
NS125	Australia	Oceania	1993	invasive, NOS	emm95.0	604	11	32	30	31	37	7	46	51	1	2	6	44
NS14	Australia	Oceania	1991	skin/soft tissue infection, NOS	emm102.2	185	14	37	31	3	2	38	47	52	1	2	18	25
NS176	Australia	Oceania	1994	ND	emm207.1	332	11	38	32	16	38	18	48	53	1	2	6	20
NS178	Australia	Oceania	1994	skin/soft tissue infection, NOS	emm54.1	302	11	5	10	20	15	16	17	13	1	2	16	31
NS179	Australia	Oceania	1994	skin/soft tissue infection, NOS	emm9.0	204	34	39	33	32	2	1	49	54	5	3	11	45
NS190	Australia	Oceania	1994	invasive, NOS	emm92.0	312	35	40	34	33	39	39	22	24	13	2	20	22
NS223	Australia	Oceania	1994	skin/soft tissue infection, NOS	emm91.0	12	11	5	10	15	15	16	17	13	1	2	16	18
NS282	Australia	Oceania	1995	skin/soft tissue infection, NOS	emm207.1	332	11	38	32	16	38	18	48	55	1	2	6	20
NS297	Australia	Oceania	1995	ND	emm11.0	ND	1	6	12	1	20	20	13	25	8	3	21	23
NS32	Australia	Oceania	1991	invasive, NOS	emm101.0	182	15	31	10	20	40	31	38	41	1	3	32	37
NS225	Australia	Oceania	1994	invasive, NOS	emm99.0	141	14	41	35	1	20	40	26	56	5	4	20	46
NS226	Australia	Oceania	1994	invasive, NOS	emm4.5	1082	36	42	36	34	41	7	50	57	18	3	36	47
NS235	Australia	Oceania	1994	skin/soft tissue infection, NOS	emm24.1	582	14	43	33	1	42	13	32	58	22	8	37	48
NS239	Australia	Oceania	1995	invasive, NOS	emm207.1	610	6	6	6	6	5	6	6	7	5	4	6	6
NS244	Australia	Oceania	1995	ND	emm4.5	1017	36	42	36	34	41	7	50	57	18	3	36	47
NS25	Australia	Oceania	1991	invasive, NOS	emm55.0	1018	11	11	7	10	10	13	11	13	8	3	11	12
NS253	Australia	Oceania	1995	invasive, NOS	emm52.1	180	15	17	10	17	18	18	21	23	12	8	19	21
NS265	Australia	Oceania	1995	ND	emm56.0	115	14	44	24	2	12	41	51	22	23	1	6	49
NS488	Australia	Oceania	1995	invasive, NOS	emm12.0	1019	14	3	3	3	2	3	52	59	24	12	38	[S]
NS578	Australia	Oceania	1995	ND	emm232.0	110	18	18	16	16	21	42	27	26	25	3	21	51
NS581	Australia	Oceania	1995	invasive, NOS	emm42.1	80	37	32	37	35	43	43	44	60	26	2	39	52
NS59	Australia	Oceania	1992	ND	emm53.0	299	11	5	10	20	15	16	17	13	1	2	18	3
NS495	Australia	Oceania	1995	skin/soft tissue infection, NOS	emm90.5	184	20	20	14	19	2	23	25	28	15	3	22	25
NS50	Australia	Oceania	1991	invasive, NOS	emm218.1	292	38	45	3	16	44	44	53	61	27	2	12	3
NS506	Australia	Oceania	1995	invasive, NOS	emm14.4	118	11	5	38	8	15	16	17	13	1	2	40	53
NS516	Australia	Oceania	1995	ND	emm110.0	596	14	23	18	6	25	27	40	43	1	2	18	28
NS534	Australia	Oceania	1995	invasive, NOS	emm232.0	110	18	18	16	16	21	42	27	26		3	21	51
NS539	Australia	Oceania	1995	skin/soft tissue infection, NOS	emm76.6	291	39	46	39	36	45	45	54	62	2	2	41	54

NS567	Australia	Oceania	1995	skin/soft tissue infection, NOS	emm193.0	108	14	47	40	1	20	2	55	63	8	2	42	55
NS611	Australia	Oceania	1995	invasive, NOS	emm15.1	629	14	48	41	2	2	2	56	13	8	3	43	56
NS733	Australia	Oceania	1996	invasive, NOS	emm90.5	184	20	20	14	19	2	23	25	28	15	3	22	25
NS736	Australia	Oceania	1996	ND	emm221.0	113	11	49	2	37	2	32	57	42	1	2	23	57
NS755	Australia	Oceania	1996	invasive, NOS	emm90.5	184	20	20	14	19	2	23	25	28	15	3	22	25
NS671	Australia	Oceania	1995	invasive, NOS	emm81.3	183	8	4	4	4	3	13	4	42	21	1	4	4
NS672	Australia	Oceania	1995	invasive, NOS	emm28.0	52	3	3	3	3	2	3	3	4	1	1	44	3
NS684	Australia	Oceania	1996	skin/soft tissue infection, NOS	emm81.2	290	24	50	25	27	2	33	58	64	18	3	28	30
NS687	Australia	Oceania	1996	ND	emm58.0	176	14	13	10	29	13	13	15	47	10	2	34	31
NS691	Australia	Oceania	1996	skin/soft tissue infection, NOS	emm65.0	111	22	22	17	38	23	43	28	65	1	1	1	27
NS702	Australia	Oceania	1996	ARF	emm65.0	111	22	22	17	38	23	43	28	65	1	1	1	27
NS718	Australia	Oceania	1996	skin/soft tissue infection, NOS	emm92.0	312	35	40	34	33	39	39	22	24	13	2	20	22
NS731	Australia	Oceania	1996	invasive, NOS	emm90.5	184	20	20	14	19	2	23	25	28	15	3	22	25
NS5530	Australia	Oceania	1993	ND	emm232.1	288	18	18	16	16	21	25	27	26	16	3	21	23
NS804	Australia	Oceania	1996	invasive, NOS	emm97.1	216	21	21	15	8	46	24	26	30	8	10	12	26
NS833	Australia	Oceania	1996	ND	emm221.0	113	11	49	2	37	2	32	57	42	1	2	23	57
NS836	Australia	Oceania	1996	ND	emm221.0	113	11	49	2	37	2	32	57	42	1	2	23	57
NS930	Australia	Oceania	1996	pharyngitis and/or tonsillitis	emm65.0	111	22	22	17	38	23	43	28	65	1	1	1	27
NS5341	Australia	Oceania	1991	invasive, NOS	emm85.0	109	10	10	4	9	9	11	10	11	7	3	10	10
NS5347	Australia	Oceania	1991	skin/soft tissue infection, NOS	emm4.5	289	14	51	42	39	47	33	59	66	8	13	45	58
NS35	Australia	Oceania	1991	invasive, NOS	emm58.0	176	14	13	10	29	13	13	15	47	10	2	34	31
NS473	Australia	Oceania	1995	invasive, NOS	emm232.0	110	18	18	16	16	21	42	27	26	25	3	21	51
NS474	Australia	Oceania	1995	invasive, NOS	emm58.0	176	14	13	10	29	13	24	15	47	10	2	34	31
NS476	Australia	Oceania	1995	invasive, NOS	ND	1084	16	16	3	16	17	46	20	22	1	2	46	3
NS351	Australia	Oceania	1995	ND	emm58.0	176	14	13	10	29	13	24	15	47	10	2	34	31
NS365	Australia	Oceania	1995	invasive, NOS	emm58.0	176	14	13	10	29	13	13	15	47	10	2	34	31
NS414	Australia	Oceania	1995	skin/soft tissue infection, NOS	emm11.0	ND	1	6	12	1	20	20	13	25	8	3	21	23
NS415	Australia	Oceania	1995	skin/soft tissue infection, NOS	emm4.5	177	36	42	36	34	41	7	50	57	18	3	36	47
NS43	Australia	Oceania	1991	pharyngitis and/or tonsillitis	emm25.0	600	30	11	7	40	48	31	60	67	1	1	47	40
NS436	Australia	Oceania	1995	ND	emm11.0	ND	1	6	12	1	20	20	13	25	8	3	21	23
NS452	Australia	Oceania	1995	skin/soft tissue infection, NOS	emm25.0	191	30	35	26	28	35	34	42	45	11	2	12	40
NS455	Australia	Oceania	1995	invasive, NOS	emm52.1	180	15	17	10	17	18	18	21	23	12	8	19	21
NS6	Australia	Oceania	1990	invasive, NOS	emm123.0	325	6	6	6	6	5	6	6	7	5	4	6	6
NS8	Australia	Oceania	1990	skin/soft tissue infection, NOS	emm85.0	109	10	10	4	9	9	11	10	11	7	3	10	10
NS16	Australia	Oceania	1991	ND	emm77.0	166	14	25	3	22	27	13	26	29	8	3	25	30
NS205	Australia	Oceania	1994	invasive, NOS	emm56.1	115	14	44	24	2	12	41	51	22	23	1	6	49
NS232	Australia	Oceania	1994	pharyngitis and/or tonsillitis	emm49.3	190	4	8	4	11	11	14	12	14	9	2	12	13
NS234	Australia	Oceania	1994	pharyngitis and/or tonsillitis	emm42.0	80	37	32	37	35	43	43	44	60	26	2	39	52
NS237	Australia	Oceania	1994	ND	emm97.0	203	11	49	2	37	2	32	61	42	1	2	23	57
NS343	Australia	Oceania	1995	skin/soft tissue infection, NOS	emm225.0	48	28	33	18	6	49	6	62	68	29	2	48	59
NS501	Australia	Oceania	1995	invasive, NOS	emm14.4	118	11	5	38	8	15	16	17	13	1	2	40	53
NS514	Australia	Oceania	1995	ND	emm73.0	331	14	52	3	41	50	13	3	69	7	2	49	60
NS678	Australia	Oceania	1995	pharyngitis and/or tonsillitis	emm4.0	39	4	8	4	7	51	9	63	9	7	2	8	8
NS909	Australia	Oceania	1996	invasive, NOS	emm81.0	117	40	53	43	42	52	47	64	70	30	14	30	61
NS1043	Australia	Oceania	1997	skin/soft tissue infection, NOS	emm222.0	116	21	54	44	1	35	24	26	71	8	15	12	62
NS1051	Australia	Oceania	1997	invasive, NOS	emm114.2	189	41	28	40	43	53	40	65	72	18	3	50	63
NS1185	Australia	Oceania	1997	invasive, NOS	emm80.2	10	16	16	3	16	17	46	20	22	1	2	46	3
NS1196	Australia	Oceania	1997	skin/soft tissue infection, NOS	emm60.4	193	14	34	25	27	2	33	41	44	1	2	33	39

NS1353	Australia	Oceania	1998	skin/soft tissue infection, NOS	emm233.1	206	11	49	2	37	54	32	61	42	31	1	51	25
NS1425	Australia	Oceania	1998	skin/soft tissue infection, NOS	emm13.0	286	14	13	10	8	13	13	15	17	10	2	15	16
NS1438	Australia	Oceania	1997	skin/soft tissue infection, NOS	emm71.2	318	19	20	13	19	2	22	3	27	1	1	4	24
NS1498	Australia	Oceania	1998	skin/soft tissue infection, NOS	emm217.0	594	42	55	3	6	5	48	66	73	1	2	52	11
NS1691	Australia	Oceania	1999	skin/soft tissue infection, NOS	emm75.3	590	10	56	45	6	55	2	67	74	1	2	30	31
NS1932	Australia	Oceania	1999	skin/soft tissue infection, NOS	emm22.0	175	33	11	7	10	35	36	44	49	19	2	30	64
NS1944	Australia	Oceania	1999	skin/soft tissue infection, NOS	emm18.12	535	43	57	2	2	2	2	67	75	8	3	53	2
NS1951	Australia	Oceania	1999	pharyngitis and/or tonsillitis	emm90.2	418	14	13	10	8	13	13	15	17	10	2	15	16
NS1980	Australia	Oceania	1999	skin/soft tissue infection, NOS	emm77.0	572	14	25	3	39	56	13	68	76	32	3	54	65
NS1984	Australia	Oceania	1999	skin/soft tissue infection, NOS	emm82.0	26	14	58	46	1	6	18	69	77	1	1	1	3
NS1988	Australia	Oceania	1999	ND	emm9.0	603	44	39	33	32	2	1	49	54	5	3	11	45
NS1999	Australia	Oceania	1999	skin/soft tissue infection, NOS	emm75.3	590	10	56	45	6	55	2	67	74	1	2	30	31
NS2000	Australia	Oceania	1999	skin/soft tissue infection, NOS	emm77.0	63	23	24	19	1	26	28	30	33	8	3	24	29
NS2012	Australia	Oceania	1999	skin/soft tissue infection, NOS	emm99.5	593	11	32	47	1	57	32	61	32	1	2	55	66
NS2023	Australia	Oceania	1999	pharyngitis and/or tonsillitis	emm22.0	46	7	5	5	5	4	5	5	6	4	3	5	5
NS2128	Australia	Oceania	2000	ND	emm192.0	612	11	5	10	44	57	49	70	42	1	2	23	31
NS2231	Australia	Oceania	2000	skin/soft tissue infection, NOS	emm49.3	190	4	8	4	11	11	14	12	14	9	2	12	13
NS2416	Australia	Oceania	2000	skin/soft tissue infection, NOS	emm104.0	597	45	34	48	8	58	50	71	78	8	3	31	67
NS2509	Australia	Oceania	2000	skin/soft tissue infection, NOS	emm85.0	1020	10	59	4	9	9	51	72	11	7	3	10	68
NS2663	Australia	Oceania	2001	skin/soft tissue infection, NOS	emm53.0	363	46	32	7	1	6	7	73	79	1	16	56	31
NS2664	Australia	Oceania	2001	skin/soft tissue infection, NOS	emm89.14	812	14	49	20	16	59	52	74	80	33	11	57	25
NS2671	Australia	Oceania	2001	skin/soft tissue infection, NOS	emm102.7	287	1	60	7	45	60	19	75	79	1	2	18	69
NS2676	Australia	Oceania	2001	skin/soft tissue infection, NOS	emm232.0	185	14	37	31	3	2	38	47	81	1	2	58	25
NS2679	Australia	Oceania	1990	skin/soft tissue infection, NOS	emm74.0	120	28	13	3	41	50	53	76	82	11	4	59	70
NS2763	Australia	Oceania	2001	invasive, NOS	emm70.0	10	16	16	3	16	17	7	20	22	1	2	18	3
NS2811	Australia	Oceania	2001	skin/soft tissue infection, NOS	emm9.0	204	34	39	33	32	2	1	49	54	5	3	11	45
NS3003	Australia	Oceania	2001	skin/soft tissue infection, NOS	emm225.0	48	28	33	18	6	49	6	62	68	29	2	48	59
NS3275	Australia	Oceania	2002	skin/soft tissue infection, NOS	emm8.0	602	18	18	7	10	10	7	77	39	19	2	30	31
NS3309	Australia	Oceania	2002	invasive, NOS	emm86.1	4	47	5	10	46	15	16	17	13	1	7	60	71
NS3332	Australia	Oceania	2002	skin/soft tissue infection, NOS	emm108.0	304	48	5	10	20	15	16	31	13	1	2	16	31
NS3335	Australia	Oceania	2002	skin/soft tissue infection, NOS	emm39.4	268	1	60	49	45	61	19	13	83	34	3	61	29
NS3473	Australia	Oceania	2002	ND	emm82.0	26	14	58	46	1	6	18	69	77	1	1	1	3
NS4505	Australia	Oceania	2004	skin/soft tissue infection, NOS	emm110.0	179	14	33	18	6	25	27	40	43	1	2	18	38
NS4517	Australia	Oceania	2004	skin/soft tissue infection, NOS	emm53.0	11	49	5	10	8	15	54	17	84	1	2	6	20
NS4518	Australia	Oceania	2004	ND	emm12.1	36	14	3	3	3	62	3	52	59	24	12	38	3
NS4618	Australia	Oceania	2004	ND	emm76.6	291	39	46	39	36	45	45	54	85	2	2	62	54
NS4624	Australia	Oceania	2004	skin/soft tissue infection, NOS	emm19.4	616	50	32	10	1	6	55	78	13	1	2	6	31
NS4697	Australia	Oceania	2005	ND	emm1.0	28	1	1	1	1	1	1	1	1	1	1	1	1
NS4706	Australia	Oceania	2005	ND	emm13.0	286	14	13	10	8	13	13	15	17	10	2	15	16
NS4716	Australia	Oceania	2005	ND	emm91.1	12	11	5	10	15	15	16	17	13	1	2	16	18
NS4743	Australia	Oceania	2005	ND	emm58.8	549	51	61	50	47	63	56	79	86	35	3	35	72
NS4766	Australia	Oceania	2005	skin/soft tissue infection, NOS	emm183.2	586	11	62	3	22	27	13	26	29	8	3	25	30
NS3523	Australia	Oceania	2002	ND	emm197.0	615	11	63	32	24	64	57	80	42	15	3	23	57
NS4797	Australia	Oceania	2005	skin/soft tissue infection, NOS	emm100.0	119	52	11	7	10	10	7	37	39	19	2	30	31
NS4800	Australia	Oceania	2005	skin/soft tissue infection, NOS	emm97.1	216	21	21	15	8	46	24	26	30	8	10	12	26
NS4804	Australia	Oceania	2005	skin/soft tissue infection, NOS	emm81.2	608	11	11	7	48	65	13	81	79	21	1	4	4
NS4883	Australia	Oceania	2005	skin/soft tissue infection, NOS	emm103.0	611	14	13	3	1	66	13	82	87	1	2	49	4
NS4982	Australia	Oceania	2005	skin/soft tissue infection, NOS	emm42.0	80	37	32	37	35	43	43	44	60	26	2	39	52

NS4984	Australia	Oceania	2005	skin/soft tissue infection, NOS	emm124.0	544	18	18	4	16	21	21	23	26	8	9	21	23
NS4985	Australia	Oceania	2005	skin/soft tissue infection, NOS	emm14.4	118	11	5	38	8	15	16	17	13	1	2	40	53
NS5025	Australia	Oceania	2005	ND	emm105.0	954	6	6	6	6	5	12	6	12	5	4	6	11
NS5048	Australia	Oceania	2005	ND	emm55.0	100	11	11	7	10	10	13	11	13	8	3	11	12
NS5128	Australia	Oceania	2005	ND	emm77.0	588	14	25	51	39	56	13	68	76	32	3	54	65
NS3785	Australia	Oceania	2003	ND	emm12.0	36	14	3	3	3	2	3	52	59	24	12	38	3
NS5146	Australia	Oceania	2005	ND	emm78.3	181	11	32	52	1	67	13	83	88	1	2	63	73
NS5340	Australia	Oceania	1991	skin/soft tissue infection, NOS	emm65.4	1021	53	64	53	35	68	43	44	89	2	2	39	2
NS5378	Australia	Oceania	1991	skin/soft tissue infection, NOS	emm124.0	544	18	18	4	16	69	21	23	26	8	3	21	23
NS5427	Australia	Oceania	1991	ND	emm63.0	598	5	65	34	27	39	13	84	90	13	2	20	22
NS5434	Australia	Oceania	1991	ND	emm57.2	33	11	14	11	8	16	17	18	20	11	7	17	19
NS5496	Australia	Oceania	1992	skin/soft tissue infection, NOS	emm77.0	994	20	66	40	38	70	6	25	91	18	3	22	25
NS5539	Australia	Oceania	1993	skin/soft tissue infection, NOS	emm33.0	1077	54	11	7	10	35	36	85	79	1	2	6	20
NS5544	Australia	Oceania	1993	skin/soft tissue infection, NOS	emm76.0	617	2	67	54	49	36	9	86	92	1	2	49	4
NS5568	Australia	Oceania	1992	skin/soft tissue infection, NOS	emm77.0	572	14	25	3	39	56	13	68	76	32	3	54	65
NS5578	Australia	Oceania	1992	skin/soft tissue infection, NOS	emm123.0	610	6	6	6	6	5	6	6	7	5	4	6	6
NS3844	Australia	Oceania	2004	ND	emm25.1	192	14	30	24	2	33	13	13	40	36	3	31	36
NS5749	Australia	Oceania	1987	skin/soft tissue infection, NOS	emm238.2	372	1	1	1	1	1	15	14	93	5	6	14	15
NS5582	Australia	Oceania	2005	ND	emm18.12	535	43	57	2	2	2	2	67	75	8	3	53	2
NS5615	Australia	Oceania	2005	skin/soft tissue infection, NOS	emm33.0	3	11	11	7	10	35	36	85	94	1	2	6	20
NS5630	Australia	Oceania	2005	skin/soft tissue infection, NOS	emm98.0	182	15	31	10	20	15	31	38	41	1	3	32	37
NS5694	Australia	Oceania	2005	skin/soft tissue infection, NOS	emm230.0	205	1	15	10	1	10	18	19	21	12	2	6	20
NS5758	Australia	Oceania	2005	skin/soft tissue infection, NOS	emm58.0	176	14	13	10	8	13	13	15	47	10	2	34	31
NS5778	Australia	Oceania	2005	skin/soft tissue infection, NOS	emm98.1	205	15	15	10	1	10	18	19	21	12	2	6	20
NS5779	Australia	Oceania	2005	skin/soft tissue infection, NOS	emm108.0	304	48	5	10	20	15	16	31	13	1	2	16	31
NS3850	Australia	Oceania	2003	ND	emm11.0	ND	1	6	12	1	20	20	13	25	8	3	21	23
NS3870	Australia	Oceania	2004	ND	emm75.1	609	27	28	55	50	71	45	35	37	18	3	28	30
NS4147	Australia	Oceania	2004	ND	emm58.0	176	14	13	10	29	13	13	15	47	10	2	34	31
NS4150	Australia	Oceania	2004	ND	emm22.0	46	7	5	5	5	4	5	5	6	4	3	5	5
NS4155	Australia	Oceania	2004	ND	emm71.0	318	19	20	13	19	2	22	3	27	1	1	4	24
NS5782	Australia	Oceania	2005	skin/soft tissue infection, NOS	emm89.0	101	2	2	2	2	2	2	2	2	2	2	2	2
NS5789	Australia	Oceania	2005	skin/soft tissue infection, NOS	emm4.0	39	4	8	4	7	7	9	8	9	37	2	8	8
NS5958	Australia	Oceania	2005	skin/soft tissue infection, NOS	emm56.0	115	14	44	24	2	12	41	51	22	23	1	6	49
NS6033	Australia	Oceania	2006	ND	emm87.0	541	55	68	10	1	72	58	87	95	1	2	52	74
NS6078	Australia	Oceania	2006	skin/soft tissue infection, NOS	ND	92	1	1	1	1	1	15	14	96	5	6	14	15
NS6087	Australia	Oceania	2006	skin/soft tissue infection, NOS	emm114.2	140	11	69	7	10	73	7	88	97	15	3	64	34
NS6142	Australia	Oceania	2006	skin/soft tissue infection, NOS	emm183.2	166	14	25	3	22	27	13	26	29	8	3	25	30
NS6185	Australia	Oceania	2006	skin/soft tissue infection, NOS	emm15.1	872	14	48	41	2	2	2	56	13	8	3	43	56
NS6187	Australia	Oceania	2006	skin/soft tissue infection, NOS	emm13.0	286	14	13	10	8	13	13	15	17	10	2	15	16
NS6221	Australia	Oceania	2006	skin/soft tissue infection, NOS	emm100.5	119	11	11	7	10	10	7	37	39	19	2	30	31
NS6263	Australia	Oceania	2006	skin/soft tissue infection, NOS	emm232.0	185	14	37	31	3	2	38	47	81	1	2	58	25
NS6441	Australia	Oceania	2006	skin/soft tissue infection, NOS	emm70.0	10	16	16	3	16	17	7	20	22	1	2	18	3
NS6474	Australia	Oceania	2006	skin/soft tissue infection, NOS	emm44.0	178	5	7	7	3	2	3	24	8	14	3	7	7
NS6531	Australia	Oceania	2006	skin/soft tissue infection, NOS	emm91.0	12	11	5	10	15	15	16	17	13	1	2	16	18
NS6570	Australia	Oceania	2007	skin/soft tissue infection, NOS	emm82.1	591	56	70	4	1	20	2	89	98	38	2	65	3
NS6595	Australia	Oceania	2007	skin/soft tissue infection, NOS	emm123.0	610	6	6	6	6	5	6	6	7	5	4	6	6
NS6648	Australia	Oceania	2007	invasive, NOS	emm41.2	579	11	5	10	51	15	16	77	99	1	2	6	20
NS6749	Australia	Oceania	2007	skin/soft tissue infection, NOS	emm54.1	302	11	5	10	20	15	16	17	13	1	2	16	31

NS6770	Australia	Oceania	2007	skin/soft tissue infection, NOS	emm11.0	ND	1	6	12	1	20	20	13	25	8	3	21	23
NS6783	Australia	Oceania	2007	skin/soft tissue infection, NOS	emm65.2	585	15	15	10	1	74	7	7	8	39	3	7	75
NS6845	Australia	Oceania	2007	skin/soft tissue infection, NOS	emm178.0	592	11	71	7	41	75	32	90	42	1	2	23	76
NS6930	Australia	Oceania	2007	skin/soft tissue infection, NOS	emm99.5	593	11	32	47	1	57	32	61	32	1	2	55	66
NS6983	Australia	Oceania	2007	skin/soft tissue infection, NOS	emm74.0	120	28	13	3	41	50	53	76	82	11	4	59	70
NS6989	Australia	Oceania	2007	skin/soft tissue infection, NOS	emm101.0	182	15	31	10	20	15	31	38	41	1	3	32	37
NS7048	Australia	Oceania	2007	skin/soft tissue infection, NOS	emm24.1	582	14	43	33	1	42	13	32	58	22	8	37	48
NS7056	Australia	Oceania	2007	skin/soft tissue infection, NOS	emm116.1	227	57	72	33	1	42	13	32	100	5	4	11	77
NS7092	Australia	Oceania	1997	skin/soft tissue infection, NOS	emm78.3	181	11	32	52	1	67	13	83	88	1	2	63	73
NS7096	Australia	Oceania	1997	skin/soft tissue infection, NOS	emm53.0	11	49	5	10	8	15	54	17	84	1	2	6	20
NS7098	Australia	Oceania	1997	skin/soft tissue infection, NOS	emm86.1	4	47	5	10	46	15	16	17	13	1	7	60	71
NS7124	Australia	Oceania	1997	ND	emm124.0	199	18	18	4	16	21	21	23	101	8	3	21	23
NS7133	Australia	Oceania	1997	ND	emm165.0	112	58	73	56	1	76	59	27	102	8	3	61	29
NS7158	Australia	Oceania	1999	skin/soft tissue infection, NOS	emm99.0	141	14	41	35	1	20	40	26	56	5	4	20	46
NS7160	Australia	Oceania	1999	ND	emm92.0	599	5	65	34	27	39	39	22	103	13	2	20	22
NS7168	Australia	Oceania	1999	skin/soft tissue infection, NOS	emm4.5	606	59	74	40	43	77	60	65	72	18	2	11	78
NS7175	Australia	Oceania	1999	skin/soft tissue infection, NOS	emm192.0	612	11	5	10	44	57	49	70	42	1	2	23	31
NS7185	Australia	Oceania	1999	ND	emm238.1	1022	60	1	1	1	1	15	14	104	1	2	30	15
NS7249	Australia	Oceania	1999	ND	emm75.3	590	10	56	45	6	55	2	67	74	1	2	30	31
NS7259	Australia	Oceania	1999	ND	emm192.0	612	11	5	10	44	57	49	70	42	1	2	23	31
NS7970	Australia	Oceania	1995	skin/soft tissue infection, NOS	emm41.2	296	11	71	7	1	78	32	91	42	1	2	23	57
NS8042	Australia	Oceania	1995	ND	emm89.0	142	61	8	3	11	79	14	92	18	9	2	12	13
NS8145	Australia	Oceania	1995	ND	emm22.0	175	33	11	7	10	35	36	44	49	19	2	30	43
NS10015	Australia	Oceania	1987	skin/soft tissue infection, NOS	emm97.0	197	11	5	10	1	57	32	93	105	1	2	23	79
patientB	Australia	Oceania	2010	ND	emm1.0	28	1	1	1	1	1	1	1	1	1	1	1	1
patientC	Australia	Oceania	2010	ND	emm77.0	1023	23	24	57	1	26	28	30	33	8	3	24	29
patientG	Australia	Oceania	2010	ND	emm89.0	101	2	2	2	2	2	2	2	2	2	2	2	2
patientH	Australia	Oceania	2010	invasive, NOS	emm75.0	150	25	26	20	24	29	29	33	48	15	3	12	33
patientI	Australia	Oceania	2010	ND	emm89.0	101	2	2	2	2	2	2	2	2	2	2	2	2
GAS_dog	Australia	Oceania	1990	ND	emm70.0	10	16	16	3	16	17	7	20	22	1	2	18	3
GAS_dogM3	Australia	Oceania	1990	ND	emm70.0	10	16	16	3	16	[S]	7	20	[S]	1	2	18	3
GAS_dog48	Australia	Oceania	1990	ND	emm91.0	12	11	5	10	15	15	16	17	13	1	2	16	18
1021-1	Australia	Oceania	2004	skin/soft tissue infection, NOS	emm70.0	10	16	16	3	16	17	7	20	22	1	2	18	3
1024-1	Australia	Oceania	2004	skin/soft tissue infection, NOS	emm53.0	11	49	5	10	8	15	54	17	84	1	2	6	20
2095-1	Australia	Oceania	2004	skin/soft tissue infection, NOS	emm81.0	974	62	75	45	52	5	6	45	106	24	17	66	61
2098-1	Australia	Oceania	2004	skin/soft tissue infection, NOS	emm58.0	176	14	13	10	29	13	13	15	47	10	2	34	31
2241-1	Australia	Oceania	2004	skin/soft tissue infection, NOS	emm101.0	182	15	31	10	20	15	31	38	107	1	3	32	37
2102-1	Australia	Oceania	2004	skin/soft tissue infection, NOS	emm108.0	304	48	5	10	20	15	16	31	13	1	2	16	31
2103-1	Australia	Oceania	2004	skin/soft tissue infection, NOS	emm207.1	332	18	18	16	16	21	25	94	26	8	3	21	80
2235-1	Australia	Oceania	2004	skin/soft tissue infection, NOS	emm44.0	641	5	7	7	1	81	13	26	108	8	18	67	30
2233-1	Australia	Oceania	2004	skin/soft tissue infection, NOS	emm44.0	641	5	7	7	1	81	13	26	108	8	18	67	30
2088-1	Australia	Oceania	2004	skin/soft tissue infection, NOS	emm230.0	205	1	15	10	1	10	18	19	21	12	2	6	81
Bel007	Belgium	Europe	2004	skin/soft tissue infection, NOS	emm184.0	174	63	45	3	16	82	13	20	109	1	2	11	78
Bel006	Belgium	Europe	2004	pharyngitis and/or tonsillitis	emm19.4	1007	50	32	10	1	6	55	78	13	1	19	6	31
Bel005	Belgium	Europe	2004	pharyngitis and/or tonsillitis	emm8.0	59	14	13	3	41	50	13	3	110	7	2	49	4
Bel004	Belgium	Europe	2004	pharyngitis and/or tonsillitis	emm232.0	187	11	32	58	16	38	18	77	111	11	7	60	71
Bel003	Belgium	Europe	2004	skin/soft tissue infection, NOS	emm209.0	573	64	76	4	53	21	61	23	26	8	3	21	23
Bel002	Belgium	Europe	2004	skin/soft tissue infection, NOS	emm186.2	147	11	11	7	1	83	31	77	79	1	1	1	3

Bel001	Belgium	Europe	2004	pharyngitis and/or tonsillitis	emm75.0	150	25	26	20	24	29	29	33	112	15	3	12	33
Bra017	Brazil	South America	2011	scarlet fever	emm22.0	46	7	5	5	5	4	5	5	6	4	3	5	5
Bra005	Brazil	South America	2013	skin/soft tissue infection, NOS	emm88.2	397	14	33	59	6	25	27	40	43	1	2	18	28
Bra040	Brazil	South America	2012	invasive, NOS	emm9.0	75	34	77	60	32	2	1	95	113	5	3	11	45
Bra028	Brazil	South America	2011	scarlet fever	emm22.0	46	7	5	5	5	4	5	5	6	4	3	5	5
Bra016	Brazil	South America	2012	pharyngitis and/or tonsillitis	emm80.0	538	11	78	61	54	84	9	96	114	40	3	68	3
Bra004	Brazil	South America	2012	pharyngitis and/or tonsillitis	ND	174	14	79	3	26	82	13	20	109	1	2	11	78
Bra051	Brazil	South America	2005	scarlet fever	emm12.0	36	14	3	3	3	2	3	52	59	24	12	38	3
Bra039	Brazil	South America	2007	invasive, NOS	emm6.4	37	65	80	62	55	85	62	97	115	11	20	69	82
Bra027	Brazil	South America	2011	scarlet fever	emm22.0	46	7	5	5	5	4	5	5	6	4	3	5	5
Bra015	Brazil	South America	2007	invasive, NOS	emm4.0	38	4	8	63	11	86	9	8	9	7	2	8	8
Bra003	Brazil	South America	2009	scarlet fever	emm28.0	626	3	3	3	3	87	3	98	4	1	1	3	3
Bra050	Brazil	South America	2012	scarlet fever	emm75.0	150	25	26	20	24	29	29	33	112	15	3	12	33
Bra038	Brazil	South America	2014	skin/soft tissue infection, NOS	emm119.1	1061	28	32	10	1	88	63	78	13	1	2	6	31
Bra026	Brazil	South America	2005	skin/soft tissue infection, NOS	emm15.1	999	66	81	7	1	89	64	99	79	1	1	70	3
Bra014	Brazil	South America	2003	invasive, NOS	emm92.0	82	17	4	3	18	19	19	22	24	13	2	20	22
Bra002	Brazil	South America	2013	scarlet fever	emm87.0	62	67	15	10	1	72	58	87	95	1	2	52	74
Bra049	Brazil	South America	2004	invasive, NOS	emm83.1	5	11	5	10	8	15	65	97	115	11	2	69	83
Bra037	Brazil	South America	2013	skin/soft tissue infection, NOS	emm63.0	1005	68	10	4	37	90	66	72	116	17	11	26	3
Bra025	Brazil	South America	2003	scarlet fever	emm48.1	161	69	51	42	39	91	7	100	117	41	2	71	84
Bra013	Brazil	South America	2000	ARF	emm22.0	46	7	5	5	5	4	5	5	6	4	3	5	5
Bra001	Brazil	South America	2005	scarlet fever	emm87.0	62	67	15	10	1	72	58	87	95	1	2	52	74
Bra048	Brazil	South America	2006	scarlet fever	emm1.0	28	1	1	1	1	1	1	1	1	1	1	1	1
Bra036	Brazil	South America	2013	pharyngitis and/or tonsillitis	ND	53	70	4	3	18	19	19	101	118	42	2	72	3
Bra024	Brazil	South America	2012	pharyngitis and/or tonsillitis	emm81.0	624	5	53	43	42	52	47	64	119	30	14	30	61
Bra012	Brazil	South America	2012	skin/soft tissue infection, NOS	emm43.5	1006	11	5	10	8	15	16	77	120	1	2	6	20
Bra047	Brazil	South America	2003	scarlet fever	emm87.0	62	67	15	10	1	72	58	87	95	1	2	52	74
Bra035	Brazil	South America	2008	invasive, NOS	emm66.0	44	14	82	64	31	6	67	69	77	1	1	73	3
Bra023	Brazil	South America	2000	pharyngitis and/or tonsillitis	emm86.1	4	11	5	10	20	15	16	17	13	1	7	60	71
Bra011	Brazil	South America	2011	scarlet fever	emm12.0	36	14	3	3	3	2	3	52	59	24	12	38	3
Bra046	Brazil	South America	2004	APSGN	emm94.1	89	8	4	4	4	3	13	4	42	21	1	4	4
Bra034	Brazil	South America	2009	invasive, NOS	emm118.0	1075	14	83	3	3	92	7	25	121	36	4	74	25
Bra022	Brazil	South America	2002	pharyngitis and/or tonsillitis	emm59.0	172	71	84	24	2	93	68	102	122	18	3	75	85
Bra010	Brazil	South America	2003	pharyngitis and/or tonsillitis	emm64.3	1008	14	25	3	26	71	45	103	123	43	3	76	30
Bra045	Brazil	South America	2000	ARF	emm87.0	62	67	15	10	1	72	58	87	95	1	2	52	74
Bra033	Brazil	South America	2007	scarlet fever	emm4.0	1009	4	8	4	7	51	9	8	9	7	2	8	8
Bra021	Brazil	South America	2013	scarlet fever	emm22.0	46	7	5	5	5	4	5	5	6	4	3	5	5
Bra009	Brazil	South America	2004	scarlet fever	emm1.0	28	1	1	1	1	1	1	1	1	1	1	1	1
Bra044	Brazil	South America	2011	pharyngitis and/or tonsillitis	emm27.0	308	10	85	65	56	1	69	104	18	9	2	12	13
Bra032	Brazil	South America	2012	pharyngitis and/or tonsillitis	emm1.0	28	1	1	1	1	1	1	1	1	44	1	1	1
Bra007	Brazil	South America	2011	scarlet fever	emm101.1	11	11	86	10	20	15	16	17	13	1	2	16	31
Bra043	Brazil	South America	2003	skin/soft tissue infection, NOS	emm117.1	986	14	83	3	3	92	7	25	121	36	4	74	25
Bra031	Brazil	South America	2013	invasive, NOS	emm8.0	59	14	13	3	41	50	13	3	110	7	2	49	4
Bra019	Brazil	South America	2007	scarlet fever	emm1.0	28	1	1	1	1	1	1	1	1	44	1	1	1
Bra008	Brazil	South America	2012	ND	emm22.0	46	7	5	5	5	4	5	5	6	4	3	5	5
Bra042	Brazil	South America	2007	ARF	emm75.0	150	25	26	20	24	29	29	33	112	15	3	12	33
Bra030	Brazil	South America	2005	pharyngitis and/or tonsillitis	emm2.0	55	32	26	28	24	29	13	43	48	15	3	12	42
Bra018	Brazil	South America	2002	pharyngitis and/or tonsillitis	emm185.0	1010	18	18	66	1	10	7	105	124	1	1	77	86

Bra041	Brazil	South America	2014	skin/soft tissue infection, NOS	emm53.0	11	11	5	10	8	15	54	17	84	1	2	6	20
Bra029	Brazil	South America	2013	invasive, NOS	emm34.0	14	28	87	10	1	6	63	78	126	1	2	6	31
5448	Canada	North America	1994	necrotizing fasciitis	emm1.0	28	1	1	1	1	1	1	1	1	1	1	1	1
MGAS15252	Canada	North America	2008	ND	emm59.0	172	72	84	24	2	93	68	107	122	18	3	75	85
NGAS322	Canada	North America	ND	invasive, NOS	emm114.0	188	14	18	67	16	21	61	108	26	46	3	78	87
NGAS596	Canada	North America	ND	invasive, NOS	emm82.0	334	73	58	46	1	6	18	69	77	1	1	1	3
NGAS638	Canada	North America	ND	invasive, NOS	emm101.0	182	15	31	10	20	15	31	38	41	1	21	32	37
NGAS743	Canada	North America	ND	ND	emm87.0	62	67	15	10	1	72	58	87	95	1	2	52	74
NGAS005	Canada	North America	2012	ND	emm90.2	184	14	88	68	57	94	70	109	127	1	2	79	88
NGAS008	Canada	North America	2012	ND	emm122.2	1080	14	37	20	24	64	57	110	128	15	3	18	89
NGAS015	Canada	North America	2012	ND	emm63.3	297	24	19	3	23	28	7	32	[S]	17	11	26	32
NGAS025	Canada	North America	2012	ND	emm89.0	101	2	2	2	58	2	2	2	2	2	2	2	2
NGAS026	Canada	North America	2012	ND	emm89.0	101	2	2	2	2	2	2	2	2	2	2	2	2
NGAS035	Canada	North America	2012	ND	emm1.0	1064	1	1	1	1	1	1	1	1	1	1	1	1
NGAS056	Canada	North America	2012	ND	emm1.0	28	1	1	1	1	95	1	1	1	1	1	1	1
NGAS057	Canada	North America	2012	ND	emm88.3	971	14	89	69	35	96	2	67	74	1	2	6	31
NGAS061	Canada	North America	2012	ND	emm1.0	28	1	1	1	1	1	1	1	1	1	1	1	1
NGAS070	Canada	North America	2012	ND	emm89.0	101	2	2	2	2	97	2	2	2	2	2	2	2
NGAS071	Canada	North America	2012	ND	emm87.0	62	67	15	10	1	72	58	87	95	1	2	52	74
NGAS075	Canada	North America	2012	ND	emm9.0	75	34	77	60	32	2	1	95	113	5	3	11	45
NGAS076	Canada	North America	2012	ND	emm1.0	28	1	1	1	1	1	1	1	1	1	1	1	1
NGAS078	Canada	North America	2012	ND	emm1.0	28	1	1	1	1	1	1	1	1	1	1	1	1
NGAS083	Canada	North America	2012	ND	emm1.0	28	1	1	1	1	1	1	1	1	1	1	1	1
NGAS084	Canada	North America	2012	ND	emm28.0	52	3	3	3	3	2	3	3	4	1	1	3	3
NGAS088	Canada	North America	2012	ND	emm89.0	101	2	2	2	2	97	2	2	2	2	2	2	2
NGAS094	Canada	North America	2012	ND	emm3.1	15	15	15	10	1	10	32	111	129	1	2	6	31
NGAS096	Canada	North America	2012	ND	emm12.0	36	14	3	3	3	2	3	52	59	24	12	38	3
NGAS100	Canada	North America	2012	ND	emm3.1	15	15	15	10	1	10	32	111	129	1	2	6	31
NGAS104	Canada	North America	2012	ND	emm3.53	406	15	15	10	1	10	32	111	129	1	2	6	31
NGAS113	Canada	North America	2012	ND	emm122.0	200	11	13	3	41	50	13	112	42	31	1	51	4
NGAS123	Canada	North America	2012	ND	emm6.79	382	65	80	62	55	85	62	97	130	11	20	69	82
NGAS128	Canada	North America	2012	ND	emm14.3	84	11	5	10	8	15	16	113	13	1	2	6	20
NGAS130	Canada	North America	2012	ND	emm191.0	208	11	90	3	59	50	13	4	42	31	1	4	90
NGAS145	Canada	North America	2012	ND	emm12.0	36	14	3	3	3	2	3	52	59	24	12	38	3
NGAS148	Canada	North America	2012	ND	ND	99	14	91	70	1	17	71	114	131	47	22	80	91
NGAS151	Canada	North America	2012	ND	emm6.0	382	65	80	62	55	85	62	97	130	11	20	69	82
NGAS162	Canada	North America	2012	ND	emm6.0	382	65	80	62	55	85	62	97	130	11	20	69	82
NGAS169	Canada	North America	2012	ND	emm77.0	63	23	24	19	1	26	28	30	33	8	3	24	29
NGAS185	Canada	North America	2012	ND	emm12.8	36	74	3	3	3	2	3	52	59	24	12	38	3
NGAS197	Canada	North America	2012	ND	emm89.0	101	2	2	2	2	97	2	2	2	2	2	2	2
NGAS202	Canada	North America	2012	ND	emm1.0	28	1	1	1	1	1	1	1	1	1	1	1	1
NGAS209	Canada	North America	2012	ND	emm18.0	ND	75	92	61	5	98	72	115	132	47	2	69	5
NGAS226	Canada	North America	2012	ND	emm2.0	55	32	26	28	24	29	13	43	48	15	3	12	42
NGAS227	Canada	North America	2012	ND	emm102.2	895	14	93	71	60	99	13	116	133	13	2	81	92
NGAS228	Canada	North America	2012	ND	emm4.0	39	4	8	4	7	51	9	8	9	7	2	8	8
NGAS233	Canada	North America	2012	ND	emm89.0	101	2	2	2	2	2	2	2	2	2	2	2	2
NGAS240	Canada	North America	2012	ND	emm82.0	334	73	58	46	1	6	18	69	77	1	1	1	3
NGAS241	Canada	North America	2012	ND	emm82.0	334	73	58	46	1	6	18	69	77	1	1	1	3

NGAS245	Canada	North America	2012	ND	emm58.0	176	14	94	10	8	13	13	15	47	10	2	34	31
NGAS253	Canada	North America	2012	ND	emm83.1	853	11	5	10	8	15	73	97	115	11	2	82	83
NGAS255	Canada	North America	2012	ND	emm1.0	28	1	1	1	1	1	1	1	1	1	1	1	1
NGAS260	Canada	North America	2012	ND	emm12.8	36	74	3	3	3	2	3	52	59	24	12	38	3
NGAS266	Canada	North America	2012	ND	emm3.1	15	15	15	10	1	10	32	111	129	1	2	6	31
NGAS282	Canada	North America	2012	ND	emm89.0	101	2	2	2	2	2	2	2	2	2	2	2	2
NGAS287	Canada	North America	2012	ND	emm6.0	382	65	80	62	55	85	62	97	130	11	20	69	82
NGAS289	Canada	North America	2012	ND	emm6.0	382	65	80	62	55	85	62	97	130	11	20	69	82
NGAS290	Canada	North America	2012	ND	emm87.0	62	67	15	10	1	72	58	87	95	1	2	52	74
NGAS291	Canada	North America	2012	ND	emm87.0	62	67	15	10	1	72	58	87	95	1	2	52	74
NGAS292	Canada	North America	2012	ND	emm87.0	62	67	15	10	1	72	58	87	95	1	2	52	74
NGAS297	Canada	North America	2012	ND	emm1.0	28	1	1	1	1	1	1	1	134	1	1	1	1
NGAS298	Canada	North America	2011	invasive, NOS	emm87.0	62	67	15	10	1	72	58	87	95	1	2	52	74
NGAS299	Canada	North America	2011	invasive, NOS	emm87.0	62	67	15	10	1	72	58	87	95	1	2	52	74
NGAS300	Canada	North America	2011	invasive, NOS	emm83.1	5	11	5	10	8	15	65	97	115	11	2	69	83
NGAS302	Canada	North America	2011	invasive, NOS	emm28.0	52	3	95	3	3	2	3	117	4	1	1	3	3
NGAS304	Canada	North America	2011	invasive, NOS	emm28.0	52	3	3	3	3	2	3	3	4	1	1	3	3
NGAS305	Canada	North America	2011	invasive, NOS	emm1.0	28	1	1	1	1	1	1	1	1	1	1	1	1
NGAS308	Canada	North America	2011	invasive, NOS	emm18.0	ND	75	92	61	5	98	72	115	132	47	2	69	5
NGAS312	Canada	North America	2012	ND	emm80.0	538	11	78	61	54	84	9	96	114	40	3	68	3
NGAS320	Canada	North America	2011	invasive, NOS	emm83.1	853	11	5	10	8	15	73	97	115	11	2	82	83
NGAS323	Canada	North America	2011	ND	emm82.0	334	73	58	46	1	6	18	69	77	1	1	1	3
NGAS325	Canada	North America	2011	invasive, NOS	emm22.0	1069	76	5	5	5	4	5	5	6	4	3	5	5
NGAS328	Canada	North America	2011	ND	emm82.0	334	73	58	46	1	6	18	69	77	1	1	1	3
NGAS332	Canada	North America	2011	ND	emm82.0	334	73	58	46	1	6	18	69	77	1	1	1	3
NGAS336	Canada	North America	2011	invasive, NOS	emm1.0	28	1	1	1	1	1	1	1	134	1	1	1	1
NGAS338	Canada	North America	2011	invasive, NOS	emm82.0	334	73	58	46	1	6	18	69	77	1	1	1	3
NGAS339	Canada	North America	2011	ND	emm59.0	172	72	84	24	2	93	68	107	122	18	3	75	85
NGAS340	Canada	North America	2011	ND	emm118.0	167	11	32	52	1	67	13	118	135	1	23	83	93
NGAS344	Canada	North America	2011	invasive, NOS	emm75.0	49	25	26	20	24	29	74	33	136	15	3	12	33
NGAS346	Canada	North America	2011	ND	emm41.2	579	11	5	10	51	15	16	77	99	1	2	6	20
NGAS347	Canada	North America	2011	ND	emm82.0	334	73	58	46	1	6	18	69	77	1	1	1	3
NGAS358	Canada	North America	2012	ND	emm169.3	53	70	4	3	18	19	19	101	118	42	2	72	3
NGAS364	Canada	North America	2012	ND	emm77.0	63	23	24	57	1	26	28	30	33	8	3	24	29
NGAS367	Canada	North America	2012	ND	emm169.3	53	70	4	3	18	19	19	101	118	42	2	72	3
NGAS372	Canada	North America	2012	ND	emm92.0	82	17	4	3	18	19	19	22	24	13	2	20	22
NGAS401	Canada	North America	2012	ND	emm102.2	895	14	93	71	60	99	13	116	133	13	2	81	92
NGAS408	Canada	North America	2012	ND	emm1.0	28	1	1	1	1	1	1	1	1	1	1	1	1
NGAS413	Canada	North America	2012	ND	emm1.0	28	1	1	1	1	1	1	1	1	1	1	1	1
NGAS419	Canada	North America	2012	ND	emm3.1	15	15	15	10	1	10	32	111	129	1	2	6	31
NGAS425	Canada	North America	2012	ND	emm1.0	28	1	1	1	1	1	1	1	1	1	1	1	1
NGAS426	Canada	North America	2012	ND	emm3.1	15	15	15	10	1	10	32	111	129	1	2	6	31
NGAS428	Canada	North America	2012	ND	emm5.14	99	14	91	70	1	17	71	114	131	47	22	80	91
NGAS438	Canada	North America	2012	ND	emm73.0	331	14	96	3	41	50	13	3	137	48	2	49	94
NGAS440	Canada	North America	2012	ND	emm9.2	12	11	5	10	20	15	16	17	138	1	2	16	31
NGAS447	Canada	North America	2012	ND	emm12.0	36	14	3	3	3	2	3	52	59	24	12	38	3
NGAS450	Canada	North America	2012	ND	emm1.0	28	1	1	1	1	1	1	1	1	1	1	1	1
NGAS465	Canada	North America	2012	ND	emm29.1	12	11	5	10	20	15	16	17	138	1	2	16	31

NGAS473	Canada	North America	2012	ND	emm82.0	36	14	3	3	3	2	3	52	59	24	12	38	3
NGAS474	Canada	North America	2012	ND	emm87.0	62	67	15	10	1	72	58	87	95	1	2	52	74
NGAS495	Canada	North America	2012	ND	emm82.0	334	73	58	46	1	6	18	69	77	1	1	1	3
NGAS500	Canada	North America	2012	ND	emm73.0	331	14	96	3	41	50	13	3	137	48	2	49	60
NGAS501	Canada	North America	2012	ND	emm82.0	334	73	58	46	1	6	18	69	77	1	1	1	3
NGAS508	Canada	North America	2012	ND	emm87.0	62	67	15	10	1	72	58	87	95	1	2	52	74
NGAS510	Canada	North America	2012	ND	emm9.2	891	34	77	60	32	2	1	95	113	5	3	84	45
NGAS516	Canada	North America	2012	ND	emm89.0	101	2	2	2	2	97	2	2	2	2	2	2	2
NGAS520	Canada	North America	2012	ND	emm83.1	5	11	5	10	8	15	65	97	115	11	2	69	83
NGAS532	Canada	North America	2012	ND	emm11.0	ND	78	97	40	61	100	20	120	139	8	3	85	95
NGAS535	Canada	North America	2012	ND	ND	1081	14	49	33	32	2	1	121	140	5	4	86	96
NGAS556	Canada	North America	2012	ND	emm87.0	62	67	15	10	1	72	58	87	95	1	2	52	74
NGAS565	Canada	North America	2012	ND	emm87.0	62	67	15	10	1	72	58	87	95	1	2	52	74
NGAS592	Canada	North America	2012	ND	emm82.0	334	73	58	46	1	6	18	69	77	1	1	1	3
NGAS594	Canada	North America	2012	ND	emm82.0	334	73	58	46	1	6	18	69	77	1	1	1	3
NGAS595	Canada	North America	2012	ND	emm89.0	101	2	2	2	2	2	2	2	2	2	2	2	2
NGAS597	Canada	North America	2012	invasive, NOS	emm82.0	334	73	58	46	1	6	18	69	77	1	1	1	3
NGAS599	Canada	North America	2012	ND	emm114.1	188	14	18	67	16	21	61	108	26	46	3	78	87
NGAS601	Canada	North America	2012	ND	emm82.0	334	73	58	46	1	6	18	69	77	1	1	1	3
NGAS603	Canada	North America	2012	invasive, NOS	emm6.0	382	65	80	62	55	85	62	97	130	11	20	69	82
NGAS604	Canada	North America	2012	invasive, NOS	emm75.0	49	25	26	20	24	29	74	33	136	15	3	12	33
NGAS605	Canada	North America	2012	ND	emm82.0	334	73	58	46	1	6	18	69	77	1	1	1	3
NGAS606	Canada	North America	2012	invasive, NOS	emm80.0	538	11	78	61	54	84	9	96	114	40	3	68	3
NGAS608	Canada	North America	2012	ND	emm82.0	334	73	58	46	1	6	18	69	77	1	1	1	3
NGAS609	Canada	North America	2012	invasive, NOS	emm82.0	334	73	98	46	1	6	18	69	77	1	1	1	3
NGAS610	Canada	North America	2012	invasive, NOS	emm87.0	62	67	15	10	1	72	58	87	95	1	2	52	74
NGAS612	Canada	North America	2012	ND	emm87.0	62	67	15	10	1	72	58	87	95	1	2	52	74
NGAS613	Canada	North America	2012	invasive, NOS	emm82.0	334	73	58	46	1	6	18	69	77	1	1	1	3
NGAS615	Canada	North America	2012	ND	emm82.0	334	73	58	46	1	6	18	69	77	1	1	1	3
NGAS616	Canada	North America	2012	ND	emm22.0	1069	76	5	5	5	4	5	5	6	4	3	5	5
NGAS618	Canada	North America	2012	invasive, NOS	emm6.0	382	65	80	62	55	85	62	97	130	11	20	69	82
NGAS623	Canada	North America	2012	ND	emm1.0	28	1	1	1	1	1	1	1	1	1	1	1	1
NGAS629	Canada	North America	2012	ND	emm83.1	5	11	5	10	8	15	65	97	115	11	2	69	83
NGAS630	Canada	North America	2012	ND	emm87.0	62	67	15	10	1	72	58	87	95	1	2	52	74
NGAS633	Canada	North America	2012	ND	emm83.1	853	11	5	10	8	15	73	97	115	11	2	82	83
NGAS634	Canada	North America	2012	invasive, NOS	emm87.0	62	67	15	10	1	72	58	87	95	1	2	52	74
NGAS639	Canada	North America	2012	invasive, NOS	emm59.0	172	72	84	24	2	93	68	107	122	18	3	75	85
NGAS640	Canada	North America	2012	ND	emm11.0	ND	1	6	40	61	20	20	122	141	8	3	18	4
NGAS641	Canada	North America	2012	ND	emm114.0	188	14	18	67	16	21	61	108	26	46	3	78	87
NGAS643	Canada	North America	2012	ND	emm83.1	853	11	5	10	8	15	73	97	115	11	2	82	83
NGAS650	Canada	North America	2012	ND	emm3.6	315	15	15	10	1	10	32	111	129	1	2	6	31
NGAS653	Canada	North America	2012	ND	emm28.0	458	3	3	3	3	2	3	3	4	1	1	3	3
NGAS655	Canada	North America	2012	ND	emm83.1	853	11	5	10	8	15	73	97	115	11	2	82	83
NGAS657	Canada	North America	2012	ND	emm1.0	28	1	1	1	1	1	1	1	134	1	1	1	1
NGAS660	Canada	North America	2012	ND	emm89.0	101	2	2	2	2	97	2	2	2	2	2	2	2
NGAS662	Canada	North America	2012	ND	emm87.0	62	67	15	10	1	72	58	87	95	1	2	52	74
NGAS663	Canada	North America	2012	ND	emm89.0	101	2	2	2	2	97	2	2	2	2	2	2	2
NGAS664	Canada	North America	2012	ND	emm74.0	120	28	13	3	41	50	53	123	82	11	4	59	70

NGAS665	Canada	North America	2012	ND	emm87.0	62	67	15	10	1	72	58	87	95	1	2	52	74
NGAS668	Canada	North America	2012	ND	emm87.0	62	67	15	10	1	72	58	87	95	1	2	52	74
NGAS669	Canada	North America	2012	ND	emm87.0	62	67	15	10	1	72	58	87	95	1	2	52	74
NGAS670	Canada	North America	2012	ND	emm4.0	39	4	8	4	7	51	9	63	9	7	2	8	8
NGAS671	Canada	North America	2012	ND	emm2.0	55	32	26	28	24	29	13	43	48	15	3	12	42
NGAS673	Canada	North America	2012	ND	emm89.0	101	2	2	2	2	97	2	2	2	2	2	2	2
NGAS675	Canada	North America	2012	ND	emm4.0	39	4	8	4	7	51	9	63	9	7	2	8	8
NGAS717	Canada	North America	2012	ND	ND	82	17	4	3	18	19	19	22	24	13	2	20	22
NGAS719	Canada	North America	2012	ND	emm90.5	144	79	20	14	32	2	23	25	57	18	3	22	25
NGAS726	Canada	North America	2012	ND	emm68.3	894	25	26	20	24	29	13	124	48	49	3	12	42
NGAS729	Canada	North America	2012	ND	emm94.0	89	8	4	4	4	3	13	4	42	21	24	4	4
NGAS732	Canada	North America	2012	ND	emm94.0	89	8	4	4	4	3	13	4	42	21	24	4	4
NGAS737	Canada	North America	2012	ND	emm4.0	39	4	8	4	7	51	9	8	9	7	2	8	8
NGAS739	Canada	North America	2013	invasive, NOS	emm87.0	62	67	15	10	1	72	58	87	95	1	2	52	74
NGAS746	Canada	North America	2013	invasive, NOS	emm4.0	39	4	8	4	7	51	9	8	9	7	2	8	8
NGAS747	Canada	North America	2013	invasive, NOS	emm87.0	62	67	15	10	1	72	58	87	95	1	2	52	74
NGAS749	Canada	North America	2013	invasive, NOS	emm12.0	36	14	3	3	3	2	3	52	59	24	12	38	3
NGAS750	Canada	North America	2013	invasive, NOS	emm1.0	28	1	1	1	1	1	1	1	134	1	1	1	1
NGAS751	Canada	North America	2012	ND	emm115.0	135	77	11	72	1	15	31	119	142	1	1	1	3
NGAS755	Canada	North America	2013	ND	emm101.0	182	15	31	10	20	15	31	38	41	1	21	32	37
NGAS757	Canada	North America	2013	ND	emm82.0	334	73	58	46	1	6	18	69	77	1	1	1	3
NGAS758	Canada	North America	2013	ND	emm4.0	39	4	8	4	7	51	9	8	9	7	2	8	8
NGAS759	Canada	North America	2012	ND	emm4.0	39	4	8	4	7	51	9	8	9	7	2	8	8
NGAS760	Canada	North America	2012	ND	emm4.0	39	4	8	4	7	51	9	63	9	7	2	8	97
TJ11-001	China	Asia	2011	scarlet fever	emm12.0	36	14	3	3	3	2	3	52	59	24	12	38	3
AP1	Czech Republic	Europe	ND	bacteremia	emm1.0	28	1	1	1	1	1	1	1	1	1	1	1	1
33022V1T1	Fiji	Oceania	2006	pharyngitis and/or tonsillitis	emm82.1	320	56	70	4	1	[S]	2	89	98	38	2	65	3
33042V1T1	Fiji	Oceania	2006	pharyngitis and/or tonsillitis	emm14.4	118	11	5	38	8	15	16	17	13	1	2	40	53
33076V1T1	Fiji	Oceania	2006	pharyngitis and/or tonsillitis	emm232.1	1013	18	18	16	16	21	25	125	26	8	3	21	23
33087V1T1	Fiji	Oceania	2006	pharyngitis and/or tonsillitis	emm58.0	176	14	13	73	29	80	75	126	47	10	2	34	[S]
33100V1T1	Fiji	Oceania	2006	pharyngitis and/or tonsillitis	emm54.1	990	11	5	10	62	101	16	17	143	1	2	16	31
33101V1T1	Fiji	Oceania	2006	pharyngitis and/or tonsillitis	emm33.0	1060	11	11	7	10	35	36	85	144	1	2	6	20
33112V1T1	Fiji	Oceania	2006	pharyngitis and/or tonsillitis	emm42.0	1024	37	32	37	35	43	43	44	[S]	2	2	39	99
33124V1T1	Fiji	Oceania	2006	pharyngitis and/or tonsillitis	emm65.4	129	11	66	40	35	102	76	44	145	1	2	87	31
33129V1T1	Fiji	Oceania	2006	pharyngitis and/or tonsillitis	emm75.1	1078	27	28	22	26	31	77	35	[S]	18	3	28	30
33131V1T1	Fiji	Oceania	2006	pharyngitis and/or tonsillitis	emm232.1	1013	18	18	16	16	21	25	[S]	26	8	3	21	23
33133V1T1	Fiji	Oceania	2006	pharyngitis and/or tonsillitis	emm71.0	318	19	20	13	19	2	22	3	27	1	1	88	24
33140V1T1	Fiji	Oceania	2006	pharyngitis and/or tonsillitis	emm86.2	963	11	32	74	20	15	16	128	13	1	2	6	100
33149V1T1	Fiji	Oceania	2006	pharyngitis and/or tonsillitis	emm77.0	572	15	99	3	39	56	13	68	146	32	3	54	101
33167V1T1	Fiji	Oceania	2006	pharyngitis and/or tonsillitis	emm230.0	10	16	100	75	16	17	7	20	22	1	2	18	3
33173V1T1	Fiji	Oceania	2006	pharyngitis and/or tonsillitis	emm71.0	318	19	20	13	19	2	22	3	27	1	1	4	24
33181V1T1	Fiji	Oceania	2006	pharyngitis and/or tonsillitis	emm137.0	268	1	60	49	45	103	19	13	[S]	34	3	61	29
33185V1T1	Fiji	Oceania	2006	pharyngitis and/or tonsillitis	emm74.0	120	28	13	75	41	50	53	123	147	11	4	59	70
33193V1T1	Fiji	Oceania	2006	pharyngitis and/or tonsillitis	emm25.1	192	14	101	24	2	33	13	13	40	36	3	31	36
33202V1T1	Fiji	Oceania	2006	pharyngitis and/or tonsillitis	emm33.0	3	11	11	7	10	35	36	85	148	1	2	6	20
20004V1S1	Fiji	Oceania	2006	ND	emm105.0	954	6	6	6	6	5	6	6	12	5	4	6	11
20028V1I1	Fiji	Oceania	2006	invasive, NOS	emm81.3	995	8	4	4	4	3	13	4	42	21	1	4	4
20033V1I1	Fiji	Oceania	2006	invasive, NOS	emm81.3	995	8	4	4	4	3	13	4	42	21	1	4	4

20034V1I1	Fiji	Oceania	2006	bacteremia	emm76.4	353	45	34	48	8	58	50	71	57	18	3	22	102
20051V1I1	Fiji	Oceania	2006	invasive, NOS	emm76.4	353	45	34	48	8	58	50	71	57	18	3	22	102
20057V1I1	Fiji	Oceania	2006	pneumonia	emm105.0	954	6	6	6	6	5	6	6	12	5	4	6	11
20058V1I1	Fiji	Oceania	2006	invasive, NOS	emm18.22	535	14	102	2	2	2	78	67	75	8	3	89	2
20059V1I1	Fiji	Oceania	2006	pneumonia	emm58.0	176	14	13	73	29	13	13	126	47	10	2	34	31
20061V1I1	Fiji	Oceania	2006	invasive, NOS	emm76.4	353	45	34	48	8	58	50	71	57	18	3	22	102
20064V1I1	Fiji	Oceania	2006	bacteremia	emm57.0	1025	80	9	8	1	32	79	129	149	8	2	23	103
20065V1I1	Fiji	Oceania	2006	invasive, NOS	emm113.0	148	14	3	3	3	2	3	52	59	24	12	38	3
20006V1I1	Fiji	Oceania	2006	invasive, NOS	emm11.0	ND	1	6	76	1	20	20	13	25	8	3	21	23
20066V1I1	Fiji	Oceania	2006	arthritis	emm33.0	3	11	11	7	10	35	36	85	79	1	2	6	20
20068V1I1	Fiji	Oceania	2006	bacteremia	emm123.0	325	6	6	6	6	5	6	6	7	5	4	6	6
20071V1I1	Fiji	Oceania	2006	bacteremia	emm73.0	957	14	18	4	16	21	61	27	26	46	3	66	87
20077V1I1	Fiji	Oceania	2006	invasive, NOS	emm60.7	193	14	34	25	27	2	33	41	44	1	2	52	39
20079V1I1	Fiji	Oceania	2006	invasive, NOS	emm104.0	789	14	13	10	8	13	13	15	17	10	2	15	16
20081V1I1	Fiji	Oceania	2006	invasive, NOS	emm53.0	11	11	5	10	8	15	54	17	84	1	2	6	20
20085V1I1	Fiji	Oceania	2006	necrotizing fasciitis	emm11.0	ND	1	6	76	1	104	20	13	25	8	3	21	23
20086V1I1	Fiji	Oceania	2006	invasive, NOS	emm33.0	3	11	11	7	10	35	36	85	79	1	2	6	20
20095V1I1	Fiji	Oceania	2006	skin/soft tissue infection, NOS	emm73.0	957	14	18	4	16	21	61	27	26	46	3	66	87
20011V1S1	Fiji	Oceania	2006	skin/soft tissue infection, NOS	emm68.0	993	81	103	33	63	105	7	130	150	34	3	90	104
20097V1I1	Fiji	Oceania	2006	invasive, NOS	emm106.0	140	11	69	7	10	106	7	88	36	15	3	64	34
20098V1I1	Fiji	Oceania	2006	bacteremia	emm106.0	140	11	69	7	10	106	7	88	36	15	3	64	34
20099V1I1	Fiji	Oceania	2006	invasive, NOS	emm52.1	180	15	17	10	17	18	18	131	23	12	8	19	21
20109V1I1	Fiji	Oceania	2006	necrotizing fasciitis	emm100.0	119	11	11	7	10	10	7	37	39	19	2	30	31
20110V1I1	Fiji	Oceania	2006	bacteremia	emm76.4	353	45	34	48	8	58	50	71	57	18	3	22	102
20111V1I1	Fiji	Oceania	2006	bacteremia	emm15.1	872	14	48	41	2	2	2	56	13	8	3	43	105
20113V1I1	Fiji	Oceania	2006	invasive, NOS	emm100.0	119	11	11	7	10	10	7	37	39	19	2	30	31
20116V1I1	Fiji	Oceania	2006	bacteremia	emm100.0	119	11	11	7	10	10	7	37	39	19	2	30	31
20123V1I1	Fiji	Oceania	2006	bacteremia	emm100.0	119	11	11	7	10	10	7	37	39	19	2	30	31
20008V1I1	Fiji	Oceania	2006	arthritis	emm33.0	3	11	11	7	10	35	36	85	79	1	2	6	20
20139V1I1	Fiji	Oceania	2006	pneumonia	emm77.0	996	82	25	3	64	107	13	26	29	8	3	25	30
20148V1I1	Fiji	Oceania	2006	bacteremia	emm106.0	140	11	69	7	10	106	7	88	36	15	3	64	34
20170V1I1	Fiji	Oceania	2006	invasive, NOS	emm22.0	202	33	11	7	10	35	36	44	49	19	2	30	43
20174V1I1	Fiji	Oceania	2006	arthritis	emm82.1	320	56	70	4	1	20	2	89	98	38	2	65	3
20178V1I1	Fiji	Oceania	2006	bacteremia	emm11.0	ND	1	6	76	1	104	20	13	25	8	3	21	23
20183V1I1	Fiji	Oceania	2006	bacteremia	emm57.0	1025	80	9	8	1	32	79	129	149	8	2	23	103
20184V1I1	Fiji	Oceania	2006	necrotizing fasciitis	emm75.1	468	27	28	22	16	44	80	35	37	18	3	28	30
20232V1I1	Fiji	Oceania	2006	invasive, NOS	emm101.0	182	15	31	10	20	15	31	38	41	1	3	32	37
20237V1I1	Fiji	Oceania	2006	bacteremia	emm63.3	297	24	19	3	23	28	7	32	151	17	11	26	32
20242V1I1	Fiji	Oceania	2006	bacteremia	emm19.4	616	50	32	10	1	6	55	78	13	1	2	6	106
20012V1I1	Fiji	Oceania	2006	invasive, NOS	emm65.4	129	11	66	40	35	102	76	44	145	1	2	87	31
30083V1T1	Fiji	Oceania	2006	pharyngitis and/or tonsillitis	emm86.2	963	11	32	10	20	15	16	128	13	1	2	6	100
30085V1T1	Fiji	Oceania	2006	pharyngitis and/or tonsillitis	emm232.1	1013	18	18	16	16	21	25	125	26	8	3	21	23
30108V1T1	Fiji	Oceania	2006	pharyngitis and/or tonsillitis	emm11.0	ND	1	6	76	1	20	20	13	25	8	3	21	23
30109V1T1	Fiji	Oceania	2006	pharyngitis and/or tonsillitis	emm92.0	1026	5	65	34	27	39	39	22	152	13	2	20	22
30124V1T1	Fiji	Oceania	2006	pharyngitis and/or tonsillitis	emm92.0	1026	5	65	34	27	39	39	22	152	13	2	20	22
30164V1T1	Fiji	Oceania	2006	pharyngitis and/or tonsillitis	emm105.0	954	6	6	6	6	5	6	6	12	5	4	6	11
30178V1T1	Fiji	Oceania	2006	pharyngitis and/or tonsillitis	emm123.0	325	6	6	6	6	5	6	6	7	5	4	6	6
30227V1T1	Fiji	Oceania	2006	pharyngitis and/or tonsillitis	emm232.1	1013	18	18	16	16	21	25	125	26	8	3	21	23

20018V1I1	Fiji	Oceania	2006	necrotizing fasciitis	emm87.0	541	55	68	10	1	72	58	87	95	1	2	52	74
30237V1T1	Fiji	Oceania	2006	pharyngitis and/or tonsillitis	emm86.2	963	11	32	10	20	15	16	128	13	1	2	6	100
30239V1T1	Fiji	Oceania	2006	pharyngitis and/or tonsillitis	emm238.2	867	1	1	1	1	1	15	14	93	5	6	14	15
31002V1T1	Fiji	Oceania	2006	pharyngitis and/or tonsillitis	emm56.0	115	14	44	24	2	12	41	51	153	23	1	6	49
31138V1T1	Fiji	Oceania	2006	pharyngitis and/or tonsillitis	emm4.5	1027	14	51	42	39	47	33	132	79	8	3	91	107
31150V1T1	Fiji	Oceania	2006	pharyngitis and/or tonsillitis	emm56.0	115	14	44	24	2	12	41	51	153	23	1	6	49
31168V1T1	Fiji	Oceania	2006	pharyngitis and/or tonsillitis	emm101.0	182	15	31	10	20	15	31	38	41	1	3	32	37
20019V1I1	Fiji	Oceania	2006	bacteremia	emm60.7	193	14	34	25	27	2	33	41	44	1	2	52	39
20025V1I1	Fiji	Oceania	2006	arthritis	emm86.2	1028	11	32	10	20	15	16	128	13	1	2	6	100
31001V5S1	Fiji	Oceania	2006	skin/soft tissue infection, NOS	emm11.0	ND	1	6	76	1	104	20	13	25	8	3	21	23
31002V5S1	Fiji	Oceania	2006	skin/soft tissue infection, NOS	emm11.0	ND	1	6	76	1	20	20	13	25	8	3	21	23
31004V6S1	Fiji	Oceania	2006	skin/soft tissue infection, NOS	emm49.0	433	4	8	4	11	14	14	16	18	9	2	12	13
31005V6S1	Fiji	Oceania	2006	skin/soft tissue infection, NOS	emm39.4	268	1	60	49	45	61	19	13	83	34	3	61	29
31006V5S1	Fiji	Oceania	2006	skin/soft tissue infection, NOS	emm103.0	327	14	13	3	1	66	13	82	87	1	2	49	4
20027V1I1	Fiji	Oceania	2006	bacteremia	emm110.0	515	14	33	18	6	25	27	40	43	1	2	92	28
31009V5S1	Fiji	Oceania	2006	skin/soft tissue infection, NOS	emm49.0	433	4	8	4	11	14	14	16	18	9	2	12	13
31022V5S1	Fiji	Oceania	2006	skin/soft tissue infection, NOS	emm11.0	ND	1	6	76	1	20	20	13	25	8	3	21	23
31038V6S1	Fiji	Oceania	2006	skin/soft tissue infection, NOS	emm103.0	327	14	13	3	1	66	13	82	87	1	2	49	4
31039V5S1	Fiji	Oceania	2006	skin/soft tissue infection, NOS	emm49.0	433	4	8	4	11	14	14	16	18	9	2	12	13
31039V6S1	Fiji	Oceania	2006	skin/soft tissue infection, NOS	emm93.4	814	28	104	10	1	108	32	61	42	50	2	93	69
31042V5S1	Fiji	Oceania	2006	skin/soft tissue infection, NOS	emm56.0	115	14	44	24	2	12	41	51	153	23	1	6	49
31043V6S1	Fiji	Oceania	2006	skin/soft tissue infection, NOS	emm56.0	115	14	44	24	2	12	41	51	153	23	1	6	49
31046V5S1	Fiji	Oceania	2006	skin/soft tissue infection, NOS	emm82.1	320	56	70	4	1	20	2	89	98	38	2	65	3
31072V6S2	Fiji	Oceania	2006	skin/soft tissue infection, NOS	emm33.0	3	11	11	7	10	35	36	85	79	1	2	6	20
31073V5S1	Fiji	Oceania	2006	skin/soft tissue infection, NOS	emm76.4	353	45	34	48	8	58	50	71	57	18	3	22	102
31075V5S1	Fiji	Oceania	2006	skin/soft tissue infection, NOS	emm56.0	115	14	44	24	2	12	41	51	153	23	1	6	49
31086V5S1	Fiji	Oceania	2006	skin/soft tissue infection, NOS	emm65.4	129	11	66	40	35	68	76	44	145	1	2	87	31
31090V5S1	Fiji	Oceania	2006	skin/soft tissue infection, NOS	emm11.0	ND	1	6	76	1	104	20	13	25	8	3	21	23
31097V5S1	Fiji	Oceania	2006	skin/soft tissue infection, NOS	emm56.0	115	14	44	24	2	12	41	51	153	23	1	6	49
31107V5S1	Fiji	Oceania	2006	skin/soft tissue infection, NOS	emm65.4	129	11	66	40	35	102	76	44	145	1	2	87	31
31108V5S1	Fiji	Oceania	2006	skin/soft tissue infection, NOS	emm93.4	814	28	104	10	1	108	32	61	42	50	2	93	69
31113V5S1	Fiji	Oceania	2006	skin/soft tissue infection, NOS	emm74.0	120	28	13	75	41	50	53	123	82	11	4	59	70
31114V5S1	Fiji	Oceania	2006	skin/soft tissue infection, NOS	emm39.4	268	1	60	49	45	61	19	13	83	34	3	61	29
31049V5S1	Fiji	Oceania	2006	skin/soft tissue infection, NOS	emm65.4	129	11	66	40	35	68	76	44	145	1	2	87	31
31114V6S1	Fiji	Oceania	2006	skin/soft tissue infection, NOS	emm103.0	327	14	13	3	1	66	13	82	87	1	2	49	4
31126V5S1	Fiji	Oceania	2006	skin/soft tissue infection, NOS	emm65.4	129	11	66	40	35	68	76	44	145	1	2	87	31
31128V6S1	Fiji	Oceania	2006	skin/soft tissue infection, NOS	emm70.0	10	16	16	3	16	109	7	20	22	1	2	18	3
31134V5S1	Fiji	Oceania	2006	skin/soft tissue infection, NOS	emm11.0	ND	1	6	76	1	104	20	13	25	8	3	21	23
31136V5S1	Fiji	Oceania	2006	skin/soft tissue infection, NOS	emm56.0	115	14	44	24	2	12	41	51	153	23	1	6	49
31136V6S1	Fiji	Oceania	2006	skin/soft tissue infection, NOS	emm39.4	268	1	60	49	45	61	19	13	83	34	3	61	29
31138V6S1	Fiji	Oceania	2006	skin/soft tissue infection, NOS	emm39.4	268	1	60	49	45	61	19	13	83	34	3	61	29
31142V6S1	Fiji	Oceania	2006	skin/soft tissue infection, NOS	emm93.4	814	28	104	10	1	108	32	61	42	50	2	93	69
31144V6S1	Fiji	Oceania	2006	skin/soft tissue infection, NOS	emm105.0	954	6	6	6	6	5	6	6	12	5	4	6	11
31049V5S2	Fiji	Oceania	2006	skin/soft tissue infection, NOS	emm70.0	10	16	16	3	16	17	7	20	22	1	2	18	3
31145V5S1	Fiji	Oceania	2006	skin/soft tissue infection, NOS	emm93.4	814	28	104	10	1	108	32	61	42	50	2	93	69
31154V5S1	Fiji	Oceania	2006	skin/soft tissue infection, NOS	emm4.5	1027	14	51	42	39	47	33	132	79	8	3	91	107
31155V6S1	Fiji	Oceania	2006	skin/soft tissue infection, NOS	emm56.0	115	14	44	24	2	12	41	51	153	23	1	6	49
31158V5S1	Fiji	Oceania	2006	skin/soft tissue infection, NOS	emm49.0	433	4	8	4	11	14	14	16	18	9	2	12	13

31163V5S1	Fiji	Oceania	2006	skin/soft tissue infection, NOS	emm70.0	10	16	16	3	16	109	7	20	22	1	2	18	3
31165V6S1	Fiji	Oceania	2006	skin/soft tissue infection, NOS	emm39.4	268	1	60	49	45	61	19	13	83	34	3	61	29
31168V5S1	Fiji	Oceania	2006	skin/soft tissue infection, NOS	emm39.4	268	1	60	49	45	61	19	13	83	34	3	61	29
31170V5S1	Fiji	Oceania	2006	skin/soft tissue infection, NOS	emm93.4	814	28	104	10	1	108	32	61	42	50	2	93	69
41095V1S1	Fiji	Oceania	2006	skin/soft tissue infection, NOS	emm63.3	297	24	19	3	23	28	7	32	151	17	11	26	32
41126V1S1	Fiji	Oceania	2006	skin/soft tissue infection, NOS	emm11.0	ND	1	6	76	1	20	20	13	25	8	3	21	23
31050V6S1	Fiji	Oceania	2006	skin/soft tissue infection, NOS	emm73.0	957	14	18	4	16	21	61	27	26	46	3	66	87
42003V1S1	Fiji	Oceania	2006	skin/soft tissue infection, NOS	emm71.0	318	19	20	13	19	2	22	3	27	1	1	4	24
42023V1S1	Fiji	Oceania	2006	skin/soft tissue infection, NOS	emm55.0	100	11	11	7	10	10	13	11	13	8	3	11	12
42024V1S1	Fiji	Oceania	2006	skin/soft tissue infection, NOS	emm11.0	ND	1	6	76	1	104	20	13	25	8	3	21	23
42028V1S1	Fiji	Oceania	2006	skin/soft tissue infection, NOS	emm100.0	119	11	11	7	10	10	7	37	39	19	2	30	31
42043V1S1	Fiji	Oceania	2006	skin/soft tissue infection, NOS	emm74.0	120	28	13	75	41	50	53	123	82	11	4	59	70
42048V1S1	Fiji	Oceania	2006	skin/soft tissue infection, NOS	emm81.3	995	8	4	4	4	3	13	4	42	21	1	4	4
42056V1S1a	Fiji	Oceania	2006	skin/soft tissue infection, NOS	emm25.0	191	30	35	26	28	35	34	42	45	11	2	12	40
42058V1S1	Fiji	Oceania	2006	skin/soft tissue infection, NOS	emm74.1	468	27	28	22	26	31	77	35	37	18	3	28	30
31056V5S1	Fiji	Oceania	2006	skin/soft tissue infection, NOS	emm11.0	ND	1	6	76	1	104	20	13	25	8	3	21	23
42063V1S1	Fiji	Oceania	2006	skin/soft tissue infection, NOS	emm97.1	216	21	21	15	8	46	24	133	30	8	10	12	26
42070V1S1	Fiji	Oceania	2006	skin/soft tissue infection, NOS	emm44.0	178	5	7	7	3	2	3	24	8	14	3	7	7
43078V1S1	Fiji	Oceania	2006	skin/soft tissue infection, NOS	ND	1029	15	15	10	1	110	7	134	22	23	1	6	108
43086V1S1	Fiji	Oceania	2006	skin/soft tissue infection, NOS	emm11.0	ND	1	6	76	1	20	20	13	25	8	3	21	23
43110V1S1	Fiji	Oceania	2006	skin/soft tissue infection, NOS	emm57.0	1030	80	9	8	1	32	79	129	149	8	2	23	103
44001V1S1	Fiji	Oceania	2006	skin/soft tissue infection, NOS	emm123.0	325	6	6	6	6	5	6	6	7	5	4	6	6
44002V1S1	Fiji	Oceania	2006	skin/soft tissue infection, NOS	emm103.0	327	14	13	3	1	66	13	82	87	1	2	49	4
44004V1S1	Fiji	Oceania	2006	skin/soft tissue infection, NOS	emm82.1	591	56	70	4	1	20	2	135	98	38	2	65	3
44015V1S1	Fiji	Oceania	2006	skin/soft tissue infection, NOS	emm81.3	995	8	4	4	4	3	13	4	42	21	1	4	4
31056V6S1	Fiji	Oceania	2006	skin/soft tissue infection, NOS	emm11.0	ND	1	6	76	1	104	20	13	25	8	3	21	23
44031V1S1	Fiji	Oceania	2006	skin/soft tissue infection, NOS	emm82.1	320	56	70	4	1	20	2	89	98	38	2	65	3
44037V1S1	Fiji	Oceania	2006	skin/soft tissue infection, NOS	emm116.1	227	28	105	32	16	111	63	87	154	51	4	11	77
44052V1S1	Fiji	Oceania	2006	skin/soft tissue infection, NOS	emm44.0	178	5	7	7	3	2	3	24	8	14	3	7	7
44079V1S1	Fiji	Oceania	2006	skin/soft tissue infection, NOS	emm87.0	541	55	68	10	1	72	58	87	95	52	2	52	74
31004V2S1	Fiji	Oceania	2006	skin/soft tissue infection, NOS	emm70.0	10	16	16	3	16	17	7	20	22	1	2	18	3
31006V2S1	Fiji	Oceania	2006	skin/soft tissue infection, NOS	emm70.0	10	16	16	3	16	17	7	20	22	1	2	18	3
31012V2S1	Fiji	Oceania	2006	skin/soft tissue infection, NOS	emm73.4	814	28	104	10	1	108	32	61	42	50	2	93	69
31059V6S1	Fiji	Oceania	2006	skin/soft tissue infection, NOS	emm53.0	11	11	5	10	8	15	54	17	84	1	2	6	20
31026V2S1	Fiji	Oceania	2006	skin/soft tissue infection, NOS	emm105.0	954	6	6	6	6	5	6	6	12	5	4	6	11
31027V2S1	Fiji	Oceania	2006	skin/soft tissue infection, NOS	emm52.1	180	15	17	10	17	18	18	131	23	12	8	19	21
31028V2S1	Fiji	Oceania	2006	skin/soft tissue infection, NOS	emm73.0	957	14	18	4	16	21	61	27	26	46	3	66	87
31041V2S1	Fiji	Oceania	2006	skin/soft tissue infection, NOS	emm70.0	10	16	16	3	16	17	7	20	22	1	2	18	3
31056V2S1	Fiji	Oceania	2006	skin/soft tissue infection, NOS	emm65.4	129	11	66	40	35	68	76	44	145	1	2	87	31
31067V2S1	Fiji	Oceania	2006	skin/soft tissue infection, NOS	emm33.0	3	11	11	7	10	35	36	85	79	1	2	6	20
31069V2S1	Fiji	Oceania	2006	skin/soft tissue infection, NOS	emm82.1	320	56	70	4	1	20	2	89	98	38	2	65	3
31076V2S1	Fiji	Oceania	2006	skin/soft tissue infection, NOS	emm56.0	115	14	44	24	2	12	41	51	153	23	1	6	49
31062V6S1	Fiji	Oceania	2006	skin/soft tissue infection, NOS	emm103.0	327	14	13	3	1	66	13	82	87	1	2	49	4
31082V2S1	Fiji	Oceania	2006	skin/soft tissue infection, NOS	emm11.0	ND	1	6	76	1	104	20	13	25	8	3	21	23
31084V2S1	Fiji	Oceania	2006	skin/soft tissue infection, NOS	emm70.0	10	16	16	3	16	17	7	20	22	1	2	18	3
31072V6S1	Fiji	Oceania	2006	skin/soft tissue infection, NOS	emm11.0	ND	1	6	76	1	20	20	13	25	8	3	21	23
31011V3T1	Fiji	Oceania	2006	pharyngitis and/or tonsillitis	emm65.4	129	11	66	40	35	68	76	44	145	1	2	87	31
31034V1S1	Fiji	Oceania	2006	skin/soft tissue infection, NOS	emm105.0	954	6	6	6	6	5	6	6	12	5	4	6	11

31036V1S1	Fiji	Oceania	2006	skin/soft tissue infection, NOS	emm25.0	1032	30	35	26	28	35	34	42	45	11	2	12	40
31039V1S1	Fiji	Oceania	2006	skin/soft tissue infection, NOS	emm25.0	1032	30	35	26	28	35	34	42	45	11	2	12	40
31043V1S1	Fiji	Oceania	2006	skin/soft tissue infection, NOS	emm70.0	10	16	16	3	16	17	7	20	22	1	2	18	3
31063V1S1	Fiji	Oceania	2006	skin/soft tissue infection, NOS	emm73.0	957	14	18	4	16	21	61	27	26	46	3	66	87
31065V1S1	Fiji	Oceania	2006	skin/soft tissue infection, NOS	emm56.0	115	14	44	24	2	12	41	51	153	23	1	6	49
31068V1S1	Fiji	Oceania	2006	skin/soft tissue infection, NOS	emm73.0	957	14	18	4	16	21	61	27	26	46	3	66	87
31078V1S1	Fiji	Oceania	2006	skin/soft tissue infection, NOS	emm25.1	192	14	101	24	2	33	13	13	40	36	3	31	36
31080V1S1	Fiji	Oceania	2006	skin/soft tissue infection, NOS	emm25.1	192	14	101	24	2	33	13	13	40	36	3	31	36
31013V2T1	Fiji	Oceania	2006	pharyngitis and/or tonsillitis	emm39.4	268	1	60	49	45	61	19	13	83	34	3	61	29
31081V1S1	Fiji	Oceania	2006	skin/soft tissue infection, NOS	emm73.0	957	14	18	4	16	21	61	27	26	46	3	66	87
31082V1S1	Fiji	Oceania	2006	skin/soft tissue infection, NOS	emm73.0	957	14	18	4	16	21	61	27	26	46	3	66	87
31086V1S1	Fiji	Oceania	2006	skin/soft tissue infection, NOS	emm73.0	957	14	18	4	16	21	61	27	26	46	3	66	87
31106V1S1	Fiji	Oceania	2006	skin/soft tissue infection, NOS	emm25.0	1032	30	35	26	28	35	34	42	45	11	2	12	40
31111V1S1	Fiji	Oceania	2006	skin/soft tissue infection, NOS	emm93.4	814	28	104	10	1	108	32	61	42	50	2	93	69
31114V1S1	Fiji	Oceania	2006	skin/soft tissue infection, NOS	emm25.0	1032	30	35	26	28	35	34	42	45	11	2	12	40
31122V1S1	Fiji	Oceania	2006	skin/soft tissue infection, NOS	emm207.1	332	11	38	32	16	38	18	48	53	1	2	6	20
31129V1S2	Fiji	Oceania	2006	skin/soft tissue infection, NOS	emm73.0	957	14	18	4	16	21	61	27	26	46	3	66	87
31023V2T1	Fiji	Oceania	2006	pharyngitis and/or tonsillitis	emm56.0	115	14	44	24	2	12	41	51	153	23	1	6	49
31132V1S1	Fiji	Oceania	2006	skin/soft tissue infection, NOS	emm25.0	1032	30	35	26	28	35	34	42	45	11	2	12	40
31140V1S1	Fiji	Oceania	2006	skin/soft tissue infection, NOS	emm98.1	205	15	15	10	1	10	18	19	21	12	2	6	20
31143V1S1	Fiji	Oceania	2006	skin/soft tissue infection, NOS	emm71.0	318	19	20	13	19	2	22	3	27	1	1	4	24
31147V1S1	Fiji	Oceania	2006	skin/soft tissue infection, NOS	emm25.0	1032	30	35	26	28	35	34	42	45	11	2	12	40
31150V1S1	Fiji	Oceania	2006	skin/soft tissue infection, NOS	emm56.0	115	14	44	24	2	12	41	51	153	23	1	6	49
31158V1S1	Fiji	Oceania	2006	skin/soft tissue infection, NOS	emm25.0	1032	30	35	26	28	35	34	42	45	11	2	12	40
31165V1S1	Fiji	Oceania	2006	skin/soft tissue infection, NOS	emm25.0	1032	30	35	26	28	35	34	42	45	11	2	12	40
31167V1S1	Fiji	Oceania	2006	skin/soft tissue infection, NOS	emm56.0	115	14	44	24	2	12	41	51	153	23	1	6	49
31175V1S1	Fiji	Oceania	2006	skin/soft tissue infection, NOS	emm25.0	1032	30	35	26	28	35	34	42	45	11	2	12	40
31039V5T1	Fiji	Oceania	2006	pharyngitis and/or tonsillitis	emm74.0	120	28	13	75	41	50	53	123	82	11	4	59	70
31176V1S1	Fiji	Oceania	2006	skin/soft tissue infection, NOS	emm89.14	380	14	49	20	16	112	52	136	80	33	11	57	25
33014V1S1	Fiji	Oceania	2006	skin/soft tissue infection, NOS	emm105.0	954	6	6	6	6	5	6	6	12	5	4	6	11
33017V1S1	Fiji	Oceania	2006	skin/soft tissue infection, NOS	emm92.0	1026	5	65	34	27	39	39	22	152	13	2	20	22
33022V1S1	Fiji	Oceania	2006	skin/soft tissue infection, NOS	emm82.1	320	56	70	4	1	20	2	89	98	38	2	65	3
33041V1S1	Fiji	Oceania	2006	skin/soft tissue infection, NOS	emm33.0	3	11	11	7	10	35	36	85	79	1	2	6	20
33048V1S1	Fiji	Oceania	2006	skin/soft tissue infection, NOS	emm25.0	1032	30	35	26	28	35	34	42	45	11	2	12	40
33050V1S1	Fiji	Oceania	2006	skin/soft tissue infection, NOS	emm25.1	192	14	101	24	2	33	13	13	40	36	3	31	36
33052V1S1	Fiji	Oceania	2006	skin/soft tissue infection, NOS	emm75.1	468	27	28	22	26	31	77	35	37	18	3	28	30
33061V1S1	Fiji	Oceania	2006	skin/soft tissue infection, NOS	emm105.0	954	6	6	6	6	5	6	6	12	5	4	6	11
31097V2T1	Fiji	Oceania	2006	pharyngitis and/or tonsillitis	emm82.1	320	56	70	4	1	20	2	89	98	38	2	65	3
33080V1S1	Fiji	Oceania	2006	skin/soft tissue infection, NOS	emm92.0	1026	5	65	34	27	39	39	22	152	13	2	20	22
33082V1S1	Fiji	Oceania	2006	skin/soft tissue infection, NOS	emm71.0	318	19	20	13	19	2	22	3	27	1	1	88	24
33089V1S1	Fiji	Oceania	2006	skin/soft tissue infection, NOS	emm54.1	990	11	5	10	20	15	16	17	13	1	2	16	31
33100V1S1	Fiji	Oceania	2006	skin/soft tissue infection, NOS	emm54.1	990	11	5	10	20	15	16	17	13	1	2	16	31
33107V1S1	Fiji	Oceania	2006	skin/soft tissue infection, NOS	emm73.0	957	14	18	4	16	21	61	27	26	46	3	66	87
33117V1S1	Fiji	Oceania	2006	skin/soft tissue infection, NOS	emm33.0	3	11	11	7	10	35	36	85	79	1	2	6	20
33122V1S1	Fiji	Oceania	2006	skin/soft tissue infection, NOS	emm100.0	119	11	11	7	10	10	7	37	39	19	2	30	31
33122V1S2	Fiji	Oceania	2006	skin/soft tissue infection, NOS	emm86.2	963	11	32	10	20	15	16	128	13	1	2	6	100
33124V1S1	Fiji	Oceania	2006	skin/soft tissue infection, NOS	emm65.4	129	11	66	40	35	102	76	44	145	1	2	87	31
31001V1S1	Fiji	Oceania	2006	skin/soft tissue infection, NOS	emm25.0	1032	30	35	26	28	35	34	42	45	11	2	12	40

33134V1S1	Fiji	Oceania	2006	skin/soft tissue infection, NOS	emm58.0	176	14	13	73	29	13	13	126	47	10	2	34	31
33153V1S1	Fiji	Oceania	2006	skin/soft tissue infection, NOS	emm65.4	129	11	66	40	35	102	76	44	145	1	2	87	31
33156V1S1	Fiji	Oceania	2006	skin/soft tissue infection, NOS	emm86.2	963	11	32	10	20	15	16	128	13	1	2	6	100
33156V1S2	Fiji	Oceania	2006	skin/soft tissue infection, NOS	emm86.2	963	11	32	10	20	15	16	128	13	1	2	6	100
33187V1S1	Fiji	Oceania	2006	skin/soft tissue infection, NOS	emm25.1	192	14	101	24	2	33	13	13	40	36	3	31	36
33192V1S1	Fiji	Oceania	2006	skin/soft tissue infection, NOS	emm103.0	327	14	13	3	1	66	13	82	87	1	2	49	4
33193V1S1	Fiji	Oceania	2006	skin/soft tissue infection, NOS	emm25.1	192	14	101	24	2	33	13	13	40	36	3	31	36
33194V1S1	Fiji	Oceania	2006	skin/soft tissue infection, NOS	emm100.0	119	11	11	7	10	10	7	37	39	19	2	30	31
33201V1S1	Fiji	Oceania	2006	skin/soft tissue infection, NOS	emm92.0	1026	5	65	34	27	39	39	22	152	13	2	20	22
33052V2S1	Fiji	Oceania	2006	skin/soft tissue infection, NOS	emm25.0	1032	30	35	26	28	35	34	42	45	11	2	12	40
31010V1S1	Fiji	Oceania	2006	skin/soft tissue infection, NOS	emm25.0	1032	30	35	26	28	35	34	42	45	11	2	12	40
33094V2S1	Fiji	Oceania	2006	skin/soft tissue infection, NOS	emm25.0	1032	30	35	26	28	35	34	42	45	11	2	12	40
33120V2S1	Fiji	Oceania	2006	skin/soft tissue infection, NOS	emm33.0	3	11	11	7	10	35	36	85	79	1	2	6	20
33123V2S1	Fiji	Oceania	2006	skin/soft tissue infection, NOS	emm71.0	318	19	20	13	19	2	22	3	27	1	1	4	24
33139V2S1	Fiji	Oceania	2006	skin/soft tissue infection, NOS	emm85.0	109	10	10	4	9	9	11	72	11	7	3	10	10
33158V2S1	Fiji	Oceania	2006	skin/soft tissue infection, NOS	emm19.4	616	50	32	10	1	6	55	78	13	1	2	6	106
33169V2S1	Fiji	Oceania	2006	skin/soft tissue infection, NOS	emm19.4	616	50	32	10	1	6	55	78	13	1	2	6	106
33169V2S2	Fiji	Oceania	2006	skin/soft tissue infection, NOS	emm19.4	616	50	32	10	1	6	55	78	13	1	2	6	106
33188V2S2	Fiji	Oceania	2006	skin/soft tissue infection, NOS	emm73.0	957	14	18	4	16	21	61	27	26	46	3	66	87
33189V2S2	Fiji	Oceania	2006	skin/soft tissue infection, NOS	emm100.0	119	11	11	7	10	10	7	37	39	19	2	30	31
33190V2S1	Fiji	Oceania	2006	skin/soft tissue infection, NOS	emm33.0	3	11	11	7	10	35	36	85	79	1	2	6	20
31012V1S1	Fiji	Oceania	2006	skin/soft tissue infection, NOS	emm73.0	957	14	18	4	16	21	61	27	26	46	3	66	87
33191V2S1	Fiji	Oceania	2006	skin/soft tissue infection, NOS	emm52.1	180	15	17	10	17	18	18	131	23	12	8	19	21
33192V2S1	Fiji	Oceania	2006	skin/soft tissue infection, NOS	emm25.0	1032	30	35	26	28	35	34	42	45	11	2	12	40
33194V2S1	Fiji	Oceania	2006	skin/soft tissue infection, NOS	emm101.0	182	15	31	10	20	15	31	38	41	1	3	32	37
33011V3S1	Fiji	Oceania	2006	skin/soft tissue infection, NOS	emm44.0	178	5	7	7	3	2	3	24	8	14	3	7	7
33016V3S1	Fiji	Oceania	2006	skin/soft tissue infection, NOS	emm53.0	1033	46	32	7	1	6	7	73	79	1	16	56	31
33045V3S1	Fiji	Oceania	2006	skin/soft tissue infection, NOS	emm105.0	954	6	6	6	6	5	6	6	12	5	4	6	11
33080V3S1	Fiji	Oceania	2006	skin/soft tissue infection, NOS	emm82.1	320	56	70	4	1	20	2	89	98	38	2	65	3
33093V3S1	Fiji	Oceania	2006	skin/soft tissue infection, NOS	emm71.0	318	19	20	13	19	2	22	3	27	1	1	88	24
33101V3S1	Fiji	Oceania	2006	skin/soft tissue infection, NOS	emm65.4	129	11	66	40	35	102	76	44	145	1	2	87	31
33107V3S1	Fiji	Oceania	2006	skin/soft tissue infection, NOS	emm89.14	380	14	49	20	16	112	52	136	80	33	11	57	25
31032V1S2	Fiji	Oceania	2006	skin/soft tissue infection, NOS	emm25.0	1032	30	35	26	28	35	34	42	45	11	2	12	40
33115V3S1	Fiji	Oceania	2006	skin/soft tissue infection, NOS	emm93.4	814	28	104	10	1	108	32	61	42	50	2	93	69
33119V3S1	Fiji	Oceania	2006	skin/soft tissue infection, NOS	emm123.0	325	6	6	6	6	5	6	6	7	5	4	6	6
33126V3S1	Fiji	Oceania	2006	skin/soft tissue infection, NOS	emm73.0	957	14	18	4	16	21	61	27	26	46	3	66	87
33132V3S1	Fiji	Oceania	2006	skin/soft tissue infection, NOS	emm101.0	182	15	31	10	20	15	31	38	41	53	3	32	37
33139V3S1	Fiji	Oceania	2006	skin/soft tissue infection, NOS	emm60.7	193	14	34	25	27	2	33	41	44	1	2	52	39
33139V3S2	Fiji	Oceania	2006	skin/soft tissue infection, NOS	emm60.7	193	14	34	25	27	2	33	41	44	1	2	52	39
31158V1S1_01	Fiji	Oceania	2006	skin/soft tissue infection, NOS	emm25.0	1032	30	35	26	28	35	34	42	45	11	2	12	40
31158V1S2	Fiji	Oceania	2006	skin/soft tissue infection, NOS	emm25.0	1032	30	35	26	28	35	34	42	45	11	2	12	40
31158V3S1	Fiji	Oceania	2006	skin/soft tissue infection, NOS	emm93.4	814	28	104	10	1	108	32	61	42	50	2	93	69
31158V3S2_01	Fiji	Oceania	2006	skin/soft tissue infection, NOS	emm89.14	380	14	49	20	16	112	52	136	80	33	11	57	25
31158V4S1	Fiji	Oceania	2006	skin/soft tissue infection, NOS	emm89.14	380	14	49	20	16	112	52	136	80	33	11	57	25
31158V5S1_01	Fiji	Oceania	2006	skin/soft tissue infection, NOS	emm49.0	433	4	8	4	11	14	14	16	18	9	2	12	13
33181V1T1_01	Fiji	Oceania	2006	pharyngitis and/or tonsillitis	emm137.0	268	1	60	49	45	61	19	13	83	34	3	61	29
33181V3S1	Fiji	Oceania	2006	skin/soft tissue infection, NOS	emm218.1	292	38	45	3	16	44	44	53	61	54	2	12	3
33181V4T1	Fiji	Oceania	2006	pharyngitis and/or tonsillitis	emm205.0	182	15	31	10	20	15	31	38	41	1	3	32	37

31086V2S2	Fiji	Oceania	2006	skin/soft tissue infection, NOS	emm70.0	10	16	16	3	16	17	7	20	22	1	2	18	3
31142V2S1	Fiji	Oceania	2006	skin/soft tissue infection, NOS	emm70.0	10	16	16	3	16	17	7	20	22	1	2	18	3
31144V2S1	Fiji	Oceania	2006	skin/soft tissue infection, NOS	emm82.1	320	56	70	4	1	20	2	89	98	38	2	65	3
31152V2S1	Fiji	Oceania	2006	skin/soft tissue infection, NOS	emm93.4	814	28	104	10	1	108	32	61	42	50	2	93	69
31154V2S1	Fiji	Oceania	2006	skin/soft tissue infection, NOS	emm25.0	1032	30	35	26	28	35	34	42	45	11	2	12	40
31165V2S1	Fiji	Oceania	2006	skin/soft tissue infection, NOS	emm93.4	814	28	104	10	1	108	32	61	42	50	2	93	69
31166V2S1	Fiji	Oceania	2006	skin/soft tissue infection, NOS	emm82.1	320	56	70	4	1	20	2	89	98	38	2	65	3
31168V2S1	Fiji	Oceania	2006	skin/soft tissue infection, NOS	emm70.0	10	16	16	3	16	17	7	20	22	1	2	18	3
31170V2S1	Fiji	Oceania	2006	skin/soft tissue infection, NOS	emm82.1	320	56	70	4	1	20	2	89	98	38	2	65	3
31175V2S1	Fiji	Oceania	2006	skin/soft tissue infection, NOS	emm70.0	10	16	16	3	16	17	7	20	22	1	2	18	3
31176V2S2	Fiji	Oceania	2006	skin/soft tissue infection, NOS	emm103.0	327	14	13	3	1	66	13	82	87	1	2	49	4
31010V3S1	Fiji	Oceania	2006	skin/soft tissue infection, NOS	emm123.0	325	6	6	6	6	5	6	6	7	5	4	6	6
31013V3S1	Fiji	Oceania	2006	skin/soft tissue infection, NOS	emm82.1	320	56	70	4	1	20	2	89	98	38	2	65	3
31029V3S1	Fiji	Oceania	2006	skin/soft tissue infection, NOS	emm57.0	1025	80	9	8	1	32	79	129	149	8	2	23	103
31048V3S1	Fiji	Oceania	2006	skin/soft tissue infection, NOS	emm25.0	1032	30	35	26	28	35	34	42	45	11	2	12	40
31050V3S1	Fiji	Oceania	2006	skin/soft tissue infection, NOS	emm103.0	327	14	13	3	1	66	13	82	87	1	2	49	4
31069V3S1	Fiji	Oceania	2006	skin/soft tissue infection, NOS	emm4.5	1027	14	51	42	39	47	33	132	79	8	3	91	107
31089V2S1	Fiji	Oceania	2006	skin/soft tissue infection, NOS	emm4.5	1027	14	51	42	39	47	33	132	79	8	3	91	107
31086V3S1	Fiji	Oceania	2006	skin/soft tissue infection, NOS	emm104.0	789	14	13	10	8	13	13	15	17	10	2	15	16
31089V3S1	Fiji	Oceania	2006	skin/soft tissue infection, NOS	emm70.0	10	16	16	3	16	17	7	20	22	1	2	18	3
31094V3S1	Fiji	Oceania	2006	skin/soft tissue infection, NOS	emm82.1	320	56	70	4	1	20	2	89	98	38	2	65	3
31099V3S1	Fiji	Oceania	2006	skin/soft tissue infection, NOS	emm123.0	325	6	6	6	6	5	6	6	7	5	4	6	6
31118V3S1	Fiji	Oceania	2006	skin/soft tissue infection, NOS	emm82.1	320	56	70	4	1	20	2	89	98	38	2	65	3
31118V3S2	Fiji	Oceania	2006	skin/soft tissue infection, NOS	emm74.0	120	28	13	75	41	50	53	123	82	11	4	59	70
31128V3S1	Fiji	Oceania	2006	skin/soft tissue infection, NOS	emm42.0	80	37	32	37	35	43	43	44	60	2	2	39	52
31132V3S1	Fiji	Oceania	2006	skin/soft tissue infection, NOS	emm65.4	129	11	66	40	35	68	76	44	145	1	2	87	31
31143V3S1	Fiji	Oceania	2006	skin/soft tissue infection, NOS	emm89.14	380	14	49	20	16	112	52	136	80	33	11	57	25
31104V2S1	Fiji	Oceania	2006	skin/soft tissue infection, NOS	emm4.5	1027	14	51	42	39	47	33	132	79	8	3	91	107
31147V3S1	Fiji	Oceania	2006	skin/soft tissue infection, NOS	emm56.0	115	14	44	24	2	12	41	51	153	23	1	6	49
31158V3S1a	Fiji	Oceania	2006	skin/soft tissue infection, NOS	emm93.4	814	28	104	10	1	108	32	61	42	50	2	93	69
31158V3S1b	Fiji	Oceania	2006	skin/soft tissue infection, NOS	emm89.14	380	14	49	20	16	112	52	136	80	33	11	57	25
31163V3S1	Fiji	Oceania	2006	skin/soft tissue infection, NOS	emm97.1	216	21	21	15	8	46	24	26	30	8	10	12	26
31170V3S1	Fiji	Oceania	2006	skin/soft tissue infection, NOS	emm73.0	957	14	18	4	16	21	61	27	26	46	3	66	87
31171V3S1	Fiji	Oceania	2006	skin/soft tissue infection, NOS	emm93.4	814	28	104	10	1	108	32	61	42	50	2	93	69
31177V3S1	Fiji	Oceania	2006	skin/soft tissue infection, NOS	emm123.0	325	6	6	6	6	5	6	6	7	5	4	6	6
31004V4S1	Fiji	Oceania	2006	skin/soft tissue infection, NOS	emm93.4	814	28	104	10	1	108	32	61	42	50	2	93	69
31009V4S1	Fiji	Oceania	2006	skin/soft tissue infection, NOS	emm93.4	814	28	104	10	1	108	32	61	42	50	2	93	69
31032V4S1	Fiji	Oceania	2006	skin/soft tissue infection, NOS	emm104.0	789	14	13	10	8	13	13	15	17	10	2	15	16
31034V4S1	Fiji	Oceania	2006	skin/soft tissue infection, NOS	emm57.0	1025	80	9	8	1	32	79	129	149	8	2	23	103
31037V4S1	Fiji	Oceania	2006	skin/soft tissue infection, NOS	emm70.0	10	16	16	3	16	17	7	20	22	1	2	18	3
31044V4S1	Fiji	Oceania	2006	skin/soft tissue infection, NOS	emm39.4	268	1	60	49	45	61	19	13	83	34	3	61	29
31053V4S1	Fiji	Oceania	2006	skin/soft tissue infection, NOS	emm93.4	814	28	104	10	1	108	32	61	42	50	2	93	69
31058V4S1	Fiji	Oceania	2006	skin/soft tissue infection, NOS	emm103.0	327	14	13	3	1	66	13	82	87	1	2	49	4
31059V4S1	Fiji	Oceania	2006	skin/soft tissue infection, NOS	emm93.4	814	28	104	10	1	108	32	61	42	50	2	93	69
31063V4S1	Fiji	Oceania	2006	skin/soft tissue infection, NOS	emm70.0	10	16	16	3	16	17	7	20	22	1	2	18	3
31075V4S1	Fiji	Oceania	2006	skin/soft tissue infection, NOS	emm11.0	ND	1	6	76	1	20	20	13	25	8	3	21	23
31077V4S1	Fiji	Oceania	2006	skin/soft tissue infection, NOS	emm65.4	129	11	66	40	35	68	76	44	145	1	2	87	31
31107V2S1	Fiji	Oceania	2006	skin/soft tissue infection, NOS	emm105.0	954	6	6	6	6	5	6	6	12	5	4	6	11

31081V4S1	Fiji	Oceania	2006	skin/soft tissue infection, NOS	emm65.4	129	11	66	40	35	68	76	44	145	1	2	87	31
31088V4S1	Fiji	Oceania	2006	skin/soft tissue infection, NOS	emm39.4	268	1	60	49	45	61	19	13	83	34	3	61	29
31091V4S1	Fiji	Oceania	2006	skin/soft tissue infection, NOS	emm97.1	216	21	21	15	8	46	24	26	30	8	10	12	26
31101V4S1	Fiji	Oceania	2006	skin/soft tissue infection, NOS	emm89.14	380	14	49	20	16	112	52	136	80	33	11	57	25
31109V4S1	Fiji	Oceania	2006	skin/soft tissue infection, NOS	emm93.4	814	28	104	10	1	108	32	61	42	50	2	93	69
31114V4S1	Fiji	Oceania	2006	skin/soft tissue infection, NOS	emm93.4	814	28	104	10	1	108	32	61	42	50	2	93	69
31117V4S1	Fiji	Oceania	2006	skin/soft tissue infection, NOS	emm89.14	380	14	49	20	16	112	52	136	80	33	11	57	25
31119V4S1	Fiji	Oceania	2006	skin/soft tissue infection, NOS	emm89.14	380	14	49	20	16	112	52	136	80	33	11	57	25
31118V2S1	Fiji	Oceania	2006	skin/soft tissue infection, NOS	emm65.4	129	11	66	40	35	68	76	44	145	1	2	87	31
31121V4S1	Fiji	Oceania	2006	skin/soft tissue infection, NOS	emm39.4	268	1	60	49	45	61	19	13	83	34	3	61	29
31124V4S1	Fiji	Oceania	2006	skin/soft tissue infection, NOS	emm65.4	129	11	66	40	35	68	76	44	145	1	2	87	31
31125V4S1	Fiji	Oceania	2006	skin/soft tissue infection, NOS	emm65.4	129	11	66	40	35	68	76	44	145	1	2	87	31
31128V4S1	Fiji	Oceania	2006	skin/soft tissue infection, NOS	emm11.0	ND	1	6	76	1	20	20	13	25	8	3	21	23
31129V4S1	Fiji	Oceania	2006	skin/soft tissue infection, NOS	emm39.4	268	1	60	49	45	61	19	13	83	34	3	61	29
31134V4S1	Fiji	Oceania	2006	skin/soft tissue infection, NOS	emm39.4	268	1	60	49	45	61	19	13	83	34	3	61	29
31136V4S1	Fiji	Oceania	2006	skin/soft tissue infection, NOS	emm49.0	433	4	8	4	11	14	14	16	18	9	2	12	13
31148V4S1	Fiji	Oceania	2006	skin/soft tissue infection, NOS	emm70.0	10	16	16	3	16	17	7	20	22	1	2	18	3
31165V4S1	Fiji	Oceania	2006	skin/soft tissue infection, NOS	emm56.0	115	14	44	24	2	12	41	51	153	23	1	6	49
31167V4S1	Fiji	Oceania	2006	skin/soft tissue infection, NOS	emm11.0	ND	1	6	76	1	104	20	13	25	8	3	21	23
31176V4S1	Fiji	Oceania	2006	skin/soft tissue infection, NOS	emm76.4	988	45	34	48	8	58	50	71	57	18	3	22	102
33160V2T1	Fiji	Oceania	2006	pharyngitis and/or tonsillitis	emm92.0	1026	5	65	34	27	39	39	22	152	13	2	20	22
33157V2T1	Fiji	Oceania	2006	pharyngitis and/or tonsillitis	emm101.0	182	15	31	10	20	15	31	38	41	1	3	32	37
33141V2T1	Fiji	Oceania	2006	pharyngitis and/or tonsillitis	emm25.1	192	14	101	24	2	33	13	13	40	36	3	31	36
33098V2T1	Fiji	Oceania	2006	pharyngitis and/or tonsillitis	emm44.0	178	5	7	7	3	2	3	24	8	14	3	7	7
33181V3T1	Fiji	Oceania	2006	pharyngitis and/or tonsillitis	emm101.0	182	15	31	10	20	15	31	38	41	1	3	32	37
33133V3T1	Fiji	Oceania	2006	pharyngitis and/or tonsillitis	emm238.2	867	1	1	1	1	1	15	14	93	5	6	14	15
33092V2T1	Fiji	Oceania	2006	pharyngitis and/or tonsillitis	emm49.0	433	4	8	4	11	14	14	16	18	9	2	12	13
33141V4T1	Fiji	Oceania	2006	pharyngitis and/or tonsillitis	emm238.2	867	1	1	1	1	1	15	14	93	5	6	14	15
33138V3T1	Fiji	Oceania	2006	pharyngitis and/or tonsillitis	emm122.2	1029	15	15	10	1	110	7	134	22	23	1	6	108
31124V2S1	Fiji	Oceania	2006	skin/soft tissue infection, NOS	emm71.0	318	19	20	13	19	2	22	3	27	1	1	4	24
33124V2T1	Fiji	Oceania	2006	pharyngitis and/or tonsillitis	emm54.1	990	11	5	10	20	15	16	17	13	1	2	16	31
31027V4S1	Fiji	Oceania	2006	skin/soft tissue infection, NOS	emm57.0	1025	80	9	8	1	32	79	129	149	8	2	23	103
33181V5T1	Fiji	Oceania	2006	pharyngitis and/or tonsillitis	emm101.0	182	15	31	10	20	15	31	38	41	1	3	32	37
33181V6T1	Fiji	Oceania	2006	pharyngitis and/or tonsillitis	emm92.0	1026	5	65	34	27	39	39	22	152	13	2	20	22
M28PF1	France	Europe	ND	puerperal fever	emm28.0	52	3	3	3	3	2	3	3	4	1	1	3	3
STAB09014	France	Europe	2015	skin/soft tissue infection, NOS	emm28.0	52	3	3	3	3	2	3	3	4	1	1	3	3
STAB090229	France	Europe	2009	sepsis	emm75.0	150	25	26	20	24	29	29	33	48	15	3	12	33
STAB10015	France	Europe	ND	skin/soft tissue infection, NOS	emm28.0	52	3	3	3	3	2	3	3	4	1	1	3	3
7F7	France	Europe	2011	ND	emm83.1	5	11	5	10	8	15	65	97	115	11	2	69	83
STAB13021	France	Europe	2013	abscess	emm66.0	44	14	82	64	31	6	18	127	77	1	1	73	3
STAB14018	France	Europe	2014	sepsis	emm75.0	150	25	26	20	24	29	29	33	155	15	3	12	33
STAB901	France	Europe	2009	invasive, NOS	emm44.0	178	5	7	7	1	6	7	7	[S]	[S]	3	7	7
STAB902	France	Europe	ND	ND	emm3.2	406	15	15	10	1	10	32	111	129	1	2	6	31
STAB1102	France	Europe	ND	ND	emm83.1	5	11	5	10	8	15	65	97	115	11	2	69	83
1E1	France	Europe	2009	ND	emm44.0	178	5	7	7	1	6	7	7	8	14	3	7	7
HKU30	China [Hong Kong]	Asia	2011	scarlet fever	emm12.0	36	14	3	3	3	2	3	52	59	24	12	38	3
HKU22	China [Hong Kong]	Asia	2011	ND	emm12.0	36	14	3	3	3	2	3	52	59	24	12	38	3
HKU165	China [Hong Kong]	Asia	2005	ND	emm12.0	36	14	3	3	3	2	3	52	59	24	12	94	3

HKU306	China [Hong Kong]	Asia	2011	scarlet fever	emm12.0	36	14	3	3	3	2	3	52	59	24	12	38	3
HKU360	China [Hong Kong]	Asia	2011	scarlet fever	emm12.0	36	14	3	3	3	113	3	52	59	24	12	38	3
HKU383	China [Hong Kong]	Asia	2011	scarlet fever	emm12.0	36	14	3	3	3	2	3	52	59	24	12	38	3
HKU388	China [Hong Kong]	Asia	2011	scarlet fever	emm12.0	36	14	3	3	3	2	3	52	59	24	12	38	3
HKU364	China [Hong Kong]	Asia	2008	ND	emm12.0	36	14	3	3	3	2	3	52	59	24	12	38	3
HKU397	China [Hong Kong]	Asia	2011	scarlet fever	emm12.0	36	14	3	3	3	2	3	52	59	24	12	38	3
HKU434	China [Hong Kong]	Asia	2011	scarlet fever	emm1.0	28	1	1	1	1	1	1	1	156	1	1	1	1
HKU464	China [Hong Kong]	Asia	2012	pneumonia	emm1.0	28	1	1	1	1	1	1	1	1	1	1	1	1
HKU474	China [Hong Kong]	Asia	2012	scarlet fever	emm1.0	28	1	1	1	1	1	1	1	156	1	1	1	1
HKU484	China [Hong Kong]	Asia	2012	scarlet fever	emm1.0	28	1	1	1	1	114	1	1	1	1	1	1	1
HKU486	China [Hong Kong]	Asia	2011	ND	emm1.0	28	1	1	1	65	1	1	1	1	1	1	1	1
HKU487	China [Hong Kong]	Asia	2011	scarlet fever	emm1.0	28	1	1	1	1	1	1	1	1	1	1	1	1
HKU488	China [Hong Kong]	Asia	2012	scarlet fever	emm1.0	28	1	1	1	1	1	1	1	1	1	1	1	1
A1268	India	Asia	2009	invasive, NOS	emm1.0	28	1	1	1	1	1	1	1	1	1	1	1	1
A1287	India	Asia	2009	invasive, NOS	emm49.0	371	4	8	77	11	14	14	16	18	9	2	12	13
A1267	India	Asia	2009	invasive, NOS	emm43.3	1001	14	49	33	19	2	22	137	157	56	4	95	109
A1152	India	Asia	2009	invasive, NOS	emm12.0	36	14	3	3	3	2	3	52	59	24	12	38	3
A1151	India	Asia	2009	invasive, NOS	emm104.0	353	45	34	48	8	58	50	71	57	18	3	22	102
A1144	India	Asia	2009	pharyngitis and/or tonsillitis	emm12.40	36	14	3	3	3	2	3	52	59	24	12	38	3
A1138	India	Asia	2009	pharyngitis and/or tonsillitis	emm12.40	36	14	3	3	3	2	3	52	59	24	12	38	3
A1013	India	Asia	2009	ND	emm82.1	320	56	70	4	1	20	2	89	158	38	2	65	3
A998	India	Asia	2009	ND	emm80.0	701	16	16	3	16	17	7	20	22	1	2	18	110
A997	India	Asia	2009	ND	emm75.3	230	10	106	45	6	55	2	138	74	1	2	30	31
A996	India	Asia	2009	ND	emm44.0	987	5	7	7	1	6	7	7	8	14	3	7	7
A995	India	Asia	2009	ND	emm22.8	360	14	51	42	39	115	81	139	159	57	2	96	111
A993	India	Asia	2009	ND	emm80.0	715	16	16	3	16	17	82	20	22	1	2	18	3
A982	India	Asia	2009	ND	emm80.0	701	16	16	3	16	116	7	20	22	1	2	18	110
A981	India	Asia	2009	ND	emm102.2	349	14	37	31	3	55	38	47	81	1	2	58	25
A980	India	Asia	2009	ND	emm82.1	320	56	70	4	1	20	2	89	158	38	2	65	3
A969	India	Asia	2009	ND	emm100.6	119	11	11	7	10	10	7	37	39	19	2	30	31
A967	India	Asia	2009	ND	emm44.0	178	5	7	7	1	6	7	7	8	14	3	7	7
A960	India	Asia	2009	ND	emm168.1	358	24	19	78	23	117	83	45	160	58	3	97	112
A920	India	Asia	2009	ND	emm112.2	497	18	107	4	66	118	61	27	161	8	3	21	113
A915	India	Asia	2009	ND	emm49.4	534	14	108	79	22	27	13	26	29	8	3	25	114
A914	India	Asia	2009	ND	emm74.0	120	28	13	3	41	50	53	123	82	11	4	59	70
A906	India	Asia	2009	ND	emm104.0	353	45	34	48	8	58	50	71	57	18	3	22	102
A905	India	Asia	2009	ND	emm82.1	320	56	70	4	1	20	2	89	158	38	2	65	3
A904	India	Asia	2009	ND	emm113.0	499	18	18	4	4	119	7	25	57	18	3	35	115
A900	India	Asia	2009	ND	emm110.0	495	14	33	18	6	25	27	40	43	1	2	18	28
A896	India	Asia	2009	ND	emm18.12	535	83	57	2	2	2	2	67	75	8	3	53	2
A895	India	Asia	2009	ND	emm100.6	119	11	11	7	10	10	7	37	39	19	2	30	31
A879	India	Asia	2009	ND	emm12.21	991	14	3	3	3	2	3	52	59	24	12	38	3
A876	India	Asia	2009	ND	emm18.12	535	83	57	2	2	2	2	67	75	8	3	53	2
A873	India	Asia	2009	ND	emm3.23	15	15	15	10	1	10	32	111	129	1	2	6	31
A870	India	Asia	2009	ND	emm168.1	358	24	19	78	23	117	83	45	160	58	3	97	112
A868	India	Asia	2009	ND	emm108.0	304	11	109	10	20	15	16	17	13	59	2	16	31
A856	India	Asia	2009	ND	emm3.23	15	15	15	10	1	10	32	111	129	1	2	6	31
A855	India	Asia	2009	ND	emm108.0	304	11	109	10	20	15	16	17	13	59	2	16	31

A848	India	Asia	2009	pharyngitis and/or tonsillitis	emm63.3	297	24	19	3	23	120	7	32	34	17	11	26	32
A846	India	Asia	2009	ND	emm28.5	473	38	45	3	16	44	19	140	162	60	3	12	30
A843	India	Asia	2009	pharyngitis and/or tonsillitis	emm3.22	1002	9	9	80	8	8	10	9	163	5	5	98	9
A842	India	Asia	2009	ND	emm3.22	1002	9	9	80	8	8	10	9	163	5	5	98	9
A841	India	Asia	2009	ND	emm77.0	482	14	110	3	22	27	13	26	29	8	3	25	30
A840	India	Asia	2009	ND	emm77.0	482	14	110	3	22	27	13	26	29	8	3	25	30
A839	India	Asia	2009	pharyngitis and/or tonsillitis	emm75.1	357	14	34	55	50	71	45	35	37	18	3	28	30
A838	India	Asia	2009	ND	emm49.4	534	14	25	79	22	27	13	26	29	8	3	25	114
A837	India	Asia	2009	ND	emm82.1	320	56	70	4	1	20	2	89	158	38	2	65	3
A829	India	Asia	2009	pharyngitis and/or tonsillitis	emm28.5	473	38	45	3	16	44	19	140	162	60	3	12	30
JS12	Israel	Asia	1997	ND	emm179.0	619	21	111	15	8	46	24	26	30	8	10	12	26
MGAS23530	Italy	Europe	ND	pharyngitis and/or tonsillitis	emm89.0	101	2	2	2	2	2	2	2	2	2	2	2	2
JMUB1235	Japan	Asia	2016	ND	emm89.0	101	2	2	2	2	121	2	2	2	2	2	2	2
M1_476	Japan	Asia	1994	invasive, NOS	emm1.0	28	1	1	1	1	1	1	1	1	1	1	[S]	1
MTB313	Japan	Asia	ND	meningitis	emm1.0	28	1	1	1	1	1	1	141	[S]	[S]	1	1	1
MTB314	Japan	Asia	ND	meningitis	emm1.0	28	1	1	1	1	1	1	141	1	1	1	1	[S]
SSI-1	Japan	Asia	1994	invasive, NOS	emm3.1	15	15	15	10	1	10	32	111	129	1	2	6	31
K3534	Kenya	Africa	1998	skin/soft tissue infection, NOS	emm65.0	716	14	34	81	27	122	13	142	164	15	3	100	117
K5499	Kenya	Africa	2000	skin/soft tissue infection, NOS	emm65.5	215	1	112	24	1	2	13	143	165	8	3	101	118
K5721	Kenya	Africa	2000	skin/soft tissue infection, NOS	emm26.3	745	14	6	82	67	123	84	144	166	8	3	102	119
K5911	Kenya	Africa	2000	invasive, NOS	emm217.0	728	14	37	20	24	64	57	145	167	15	3	18	89
K6239	Kenya	Africa	2000	pneumonia	emm9.0	740	45	34	40	8	124	19	45	168	62	3	12	120
K6429	Kenya	Africa	2000	skin/soft tissue infection, NOS	emm38.1	757	14	37	20	24	125	13	146	23	5	25	103	117
K6821	Kenya	Africa	2001	skin/soft tissue infection, NOS	emm177.0	758	84	113	21	23	126	13	147	169	1	2	104	121
K7367	Kenya	Africa	2001	skin/soft tissue infection, NOS	emm55.0	248	11	11	83	10	10	13	148	13	8	3	11	12
K9340	Kenya	Africa	2002	skin/soft tissue infection, NOS	emm18.21	221	11	114	84	16	82	13	20	170	63	3	13	30
K9404	Kenya	Africa	2002	invasive, NOS	emm90.5	708	14	115	85	2	127	13	149	171	8	3	12	122
K9429	Kenya	Africa	2002	pneumonia	emm95.0	712	85	6	6	6	5	6	6	172	5	4	6	11
K9466	Kenya	Africa	2002	pneumonia	emm78.5	255	86	34	40	8	128	19	45	173	62	3	12	120
K12363	Kenya	Africa	2004	skin/soft tissue infection, NOS	emm79.5	714	14	25	3	22	27	13	26	29	8	3	25	30
K12452	Kenya	Africa	2004	skin/soft tissue infection, NOS	emm183.2	761	14	25	3	22	27	13	26	174	8	3	25	30
K13569	Kenya	Africa	2004	invasive, NOS	emm63.5	764	14	116	86	27	129	9	150	175	64	2	12	123
K16587	Kenya	Africa	2005	skin/soft tissue infection, NOS	ND	450	87	117	3	1	26	13	20	176	65	3	105	124
K16849	Kenya	Africa	2005	invasive, NOS	emm83.12	393	88	45	3	16	130	85	151	177	60	3	12	30
K19464	Kenya	Africa	2006	skin/soft tissue infection, NOS	ND	254	6	118	71	1	131	13	152	178	8	3	106	125
K24357	Kenya	Africa	2007	skin/soft tissue infection, NOS	emm209.0	260	14	37	3	27	132	13	153	179	34	2	107	126
K39244	Kenya	Africa	2009	pneumonia	emm80.0	715	16	16	3	16	133	7	20	22	1	2	18	3
K47483	Kenya	Africa	2011	skin/soft tissue infection, NOS	emm225.0	262	14	44	24	2	12	41	154	180	8	3	101	127
K51725	Kenya	Africa	2011	skin/soft tissue infection, NOS	emm18.21	402	11	13	3	41	50	13	112	42	31	1	51	4
K9374	Kenya	Africa	2002	skin/soft tissue infection, NOS	emm18.21	221	11	114	84	16	82	13	20	170	63	3	13	30
K9400	Kenya	Africa	2002	skin/soft tissue infection, NOS	emm90.5	708	14	115	85	2	127	13	149	171	8	3	12	122
K9887	Kenya	Africa	2003	skin/soft tissue infection, NOS	emm119.2	239	38	45	3	16	44	44	53	61	54	2	12	3
K9927	Kenya	Africa	2003	skin/soft tissue infection, NOS	emm223.0	613	85	6	6	6	5	6	6	12	5	4	6	11
K10234	Kenya	Africa	2003	skin/soft tissue infection, NOS	emm65.5	215	1	112	24	1	2	13	143	165	8	3	101	118
K10246	Kenya	Africa	2003	pneumonia	emm162.1	412	14	119	24	1	134	1	121	140	5	4	95	128
K13254	Kenya	Africa	2004	skin/soft tissue infection, NOS	emm63.5	764	14	116	86	27	129	9	150	175	64	2	12	123
K17011	Kenya	Africa	2005	skin/soft tissue infection, NOS	emm79.5	714	14	25	3	22	27	13	26	29	8	3	25	30
K17074	Kenya	Africa	2005	skin/soft tissue infection, NOS	emm218.1	292	38	45	3	16	44	44	53	61	54	2	12	3

K19188	Kenya	Africa	2006	skin/soft tissue infection, NOS	emm8.3	505	89	120	3	1	135	45	103	181	66	3	12	30
K21771	Kenya	Africa	2007	invasive, NOS	emm60.7	700	90	12	87	68	132	13	153	179	34	3	108	129
K36563	Kenya	Africa	2009	skin/soft tissue infection, NOS	emm229.0	703	91	9	8	8	136	13	129	182	8	3	106	130
K38470	Kenya	Africa	2009	skin/soft tissue infection, NOS	emm18.21	402	11	13	3	41	50	13	112	42	31	1	51	4
K42952	Kenya	Africa	2010	skin/soft tissue infection, NOS	emm111.2	737	14	37	88	16	12	13	26	29	67	3	109	89
K43037	Kenya	Africa	2010	pneumonia	emm44.0	178	5	7	7	1	6	7	7	8	14	3	7	7
K50977	Kenya	Africa	2011	invasive, NOS	emm179.0	619	21	111	15	8	46	24	26	30	8	10	12	26
K6847	Kenya	Africa	2001	skin/soft tissue infection, NOS	emm65.5	215	1	112	24	1	2	13	143	165	8	3	101	118
K6932	Kenya	Africa	2001	skin/soft tissue infection, NOS	emm217.0	728	14	37	20	24	64	57	145	167	15	3	18	89
K7275	Kenya	Africa	2001	skin/soft tissue infection, NOS	ND	450	14	121	3	1	26	13	20	176	65	3	105	124
K7478	Kenya	Africa	2001	skin/soft tissue infection, NOS	emm65.5	215	1	112	24	1	2	13	143	165	8	3	101	118
K7928	Kenya	Africa	2001	pneumonia	emm4.5	771	5	65	34	50	26	13	155	183	18	3	50	129
K37990	Kenya	Africa	2009	meningitis	emm64.3	223	14	122	89	24	64	57	156	128	15	3	110	131
K42771	Kenya	Africa	2010	pneumonia	emm124.2	231	18	123	84	16	82	13	157	184	8	3	111	132
K48186	Kenya	Africa	2011	skin/soft tissue infection, NOS	ND	731	92	16	3	16	137	24	26	185	60	3	112	133
K48650	Kenya	Africa	2011	skin/soft tissue infection, NOS	emm44.0	178	5	7	7	1	6	7	7	8	14	3	7	7
K47581	Kenya	Africa	2011	skin/soft tissue infection, NOS	emm80.0	729	16	16	3	16	17	7	20	22	1	2	18	110
K16727	Kenya	Africa	2005	skin/soft tissue infection, NOS	emm65.0	778	5	65	34	27	39	13	158	186	15	3	35	2
K48083	Kenya	Africa	2011	skin/soft tissue infection, NOS	emm179.0	619	21	111	15	8	46	24	26	187	8	10	12	26
K47118	Kenya	Africa	2011	skin/soft tissue infection, NOS	emm217.0	728	14	37	20	69	64	57	159	167	15	3	18	89
K46187	Kenya	Africa	2010	skin/soft tissue infection, NOS	emm68.8	726	1	124	4	70	138	41	160	188	68	3	12	134
K47020	Kenya	Africa	2011	ND	emm80.0	701	16	16	3	16	17	7	20	22	1	2	18	110
K37698	Kenya	Africa	2009	meningitis	emm85.1	709	14	125	90	71	139	9	161	189	11	3	113	25
K30465	Kenya	Africa	2008	skin/soft tissue infection, NOS	emm8.3	505	89	120	3	1	135	45	103	181	66	3	12	30
K23653	Kenya	Africa	2007	skin/soft tissue infection, NOS	emm8.3	241	89	120	3	1	135	45	103	181	66	3	12	30
K36535	Kenya	Africa	2009	skin/soft tissue infection, NOS	emm100.2	773	93	25	3	26	71	45	162	190	60	26	114	135
K37741	Kenya	Africa	2009	invasive, NOS	emm239.1	776	63	79	91	1	26	13	20	176	65	3	105	124
K6102	Kenya	Africa	2000	skin/soft tissue infection, NOS	emm109.1	718	94	126	3	27	19	37	163	191	15	3	35	34
K45527	Kenya	Africa	2010	invasive, NOS	emm192.0	724	14	44	24	2	12	41	154	[S]	8	3	112	3
K45900	Kenya	Africa	2010	skin/soft tissue infection, NOS	emm118.2	725	1	124	4	72	138	41	164	188	68	3	12	134
K44869	Kenya	Africa	2010	skin/soft tissue infection, NOS	ND	450	87	117	3	1	26	13	20	176	65	3	83	136
K21345	Kenya	Africa	2007	skin/soft tissue infection, NOS	ND	265	87	117	3	1	26	13	20	176	65	3	105	124
K44896	Kenya	Africa	2010	invasive, NOS	emm99.5	781	14	41	92	1	20	40	26	56	5	4	20	46
K44098	Kenya	Africa	2010	invasive, NOS	emm18.21	402	11	13	3	41	140	13	165	193	31	1	51	4
K44582	Kenya	Africa	2010	skin/soft tissue infection, NOS	emm57.0	723	21	54	44	1	35	24	166	194	8	10	12	137
K43101	Kenya	Africa	2010	ND	emm55.0	248	11	11	83	10	10	13	148	13	8	3	11	12
K43304	Kenya	Africa	2010	ND	emm18.21	402	11	13	3	41	50	13	165	42	31	1	51	4
K48877	Kenya	Africa	2011	ND	emm74.0	732	11	13	3	41	50	13	112	195	5	4	95	138
K49285	Kenya	Africa	2011	skin/soft tissue infection, NOS	emm103.0	733	63	79	91	1	26	24	26	196	65	3	105	124
K48807	Kenya	Africa	2011	invasive, NOS	emm75.1	704	14	33	18	6	25	27	40	37	18	3	28	30
K48817	Kenya	Africa	2011	invasive, NOS	emm74.0	120	11	13	3	41	50	13	112	195	5	4	95	138
K12434	Kenya	Africa	2004	skin/soft tissue infection, NOS	emm44.0	178	5	7	7	1	6	7	7	8	14	3	7	7
K25147	Kenya	Africa	2007	pneumonia	emm90.5	708	14	115	85	2	127	13	149	171	8	3	12	122
K11898	Kenya	Africa	2004	skin/soft tissue infection, NOS	emm111.2	737	14	37	88	16	12	13	26	29	69	3	109	89
K16781	Kenya	Africa	2005	skin/soft tissue infection, NOS	ND	450	87	117	3	1	26	13	20	176	65	3	105	124
K16837	Kenya	Africa	2005	skin/soft tissue infection, NOS	emm83.12	393	88	45	3	16	130	85	151	177	60	3	12	30
K13994	Kenya	Africa	2005	invasive, NOS	emm77.0	747	95	127	93	73	2	86	167	197	8	3	20	139
K16544	Kenya	Africa	2005	skin/soft tissue infection, NOS	emm11.0	250	92	128	3	26	141	24	168	198	70	3	83	28

K22338	Kenya	Africa	2006	skin/soft tissue infection, NOS	ND	450	87	117	3	1	26	13	20	176	65	3	105	124
K22633	Kenya	Africa	2007	skin/soft tissue infection, NOS	emm89.8	772	14	49	33	8	138	50	71	199	18	3	83	126
K22757	Kenya	Africa	2007	skin/soft tissue infection, NOS	emm83.12	393	88	45	3	16	130	85	151	177	60	3	12	30
K23799	Kenya	Africa	2007	skin/soft tissue infection, NOS	emm124.2	280	18	129	94	16	64	50	71	184	8	3	111	132
K22813	Kenya	Africa	2007	skin/soft tissue infection, NOS	emm121.0	262	14	44	24	2	12	41	154	200	8	3	101	118
K23180	Kenya	Africa	2007	skin/soft tissue infection, NOS	emm44.0	178	5	7	7	1	6	7	7	8	14	3	7	7
K23323	Kenya	Africa	2007	skin/soft tissue infection, NOS	emm84.1	259	14	130	95	1	142	13	169	201	60	3	115	30
K21710	Kenya	Africa	2007	skin/soft tissue infection, NOS	emm109.1	718	94	126	3	27	19	37	45	191	15	3	35	34
K19083	Kenya	Africa	2006	skin/soft tissue infection, NOS	emm56.0	115	14	44	24	2	12	41	154	180	8	3	112	3
K49882	Kenya	Africa	2011	skin/soft tissue infection, NOS	emm111.2	737	14	37	88	16	12	13	26	29	67	3	109	89
K5698	Kenya	Africa	2000	skin/soft tissue infection, NOS	emm109.1	718	94	126	3	27	19	37	45	191	15	3	35	34
K49294	Kenya	Africa	2011	skin/soft tissue infection, NOS	emm90.5	734	14	115	85	2	127	13	149	171	8	3	12	122
K49551	Kenya	Africa	2011	pneumonia	emm44.0	178	5	7	7	1	6	7	7	8	14	3	7	7
K38181	Kenya	Africa	2009	skin/soft tissue infection, NOS	emm56.0	115	14	44	24	2	12	41	154	180	8	3	112	3
K25325	Kenya	Africa	2007	pneumonia	emm90.5	708	14	115	85	2	127	13	67	171	8	3	12	122
K40810	Kenya	Africa	2010	skin/soft tissue infection, NOS	emm93.0	10	16	16	96	16	17	7	20	22	1	2	18	3
K41947	Kenya	Africa	2010	skin/soft tissue infection, NOS	emm109.1	718	94	126	3	27	19	37	45	191	15	3	35	34
K23617	Kenya	Africa	2007	skin/soft tissue infection, NOS	emm48.0	278	24	50	97	50	71	45	35	183	18	3	28	30
K6108	Kenya	Africa	2000	skin/soft tissue infection, NOS	emm25.1	323	14	7	98	2	2	13	26	202	8	3	116	140
K3637	Kenya	Africa	1998	skin/soft tissue infection, NOS	emm179.0	619	21	111	15	8	46	24	26	30	8	10	12	26
K4254	Kenya	Africa	1999	invasive, NOS	emm85.1	774	14	125	90	71	139	9	161	189	11	3	113	25
K40818	Kenya	Africa	2010	invasive, NOS	emm112.5	777	96	131	3	71	143	13	11	13	8	3	11	12
K6635	Kenya	Africa	2000	skin/soft tissue infection, NOS	emm177.0	758	84	113	21	23	126	13	147	169	1	2	104	121
K12473	Kenya	Africa	2004	invasive, NOS	emm55.0	232	11	11	83	10	10	13	148	13	8	3	11	12
K5248	Kenya	Africa	1999	skin/soft tissue infection, NOS	emm43.1	770	16	16	3	16	17	7	170	22	1	2	18	3
K16898	Kenya	Africa	2005	invasive, NOS	emm112.5	246	89	131	3	71	143	13	11	13	8	3	11	12
K7024	Kenya	Africa	2001	skin/soft tissue infection, NOS	emm18.21	402	11	13	3	41	50	13	112	42	31	1	51	4
K7087	Kenya	Africa	2001	skin/soft tissue infection, NOS	emm11.0	742	92	128	3	26	141	24	168	198	70	3	83	28
K7113	Kenya	Africa	2001	skin/soft tissue infection, NOS	emm11.0	742	92	128	3	26	141	24	168	198	70	3	83	28
K38591	Kenya	Africa	2009	skin/soft tissue infection, NOS	emm124.2	231	18	123	84	16	82	13	157	184	8	3	111	132
K41948	Kenya	Africa	2010	ND	emm208.0	719	6	132	33	1	144	19	171	203	1	3	18	141
K42600	Kenya	Africa	2010	skin/soft tissue infection, NOS	emm42.3	721	63	45	3	16	82	13	172	204	1	2	11	142
K37164	Kenya	Africa	2009	skin/soft tissue infection, NOS	emm224.1	707	14	49	33	1	42	13	173	205	1	3	18	3
K37287	Kenya	Africa	2009	skin/soft tissue infection, NOS	emm55.0	248	11	11	83	10	10	13	148	13	8	3	11	12
K36294	Kenya	Africa	2009	skin/soft tissue infection, NOS	emm49.9	705	14	133	3	1	1	13	174	206	71	3	21	143
K36347	Kenya	Africa	2009	skin/soft tissue infection, NOS	emm65.0	716	14	34	81	27	122	13	142	164	15	3	100	117
K11464	Kenya	Africa	2003	skin/soft tissue infection, NOS	emm99.5	781	14	41	92	1	20	40	26	56	5	4	20	46
K11814	Kenya	Africa	2004	skin/soft tissue infection, NOS	emm169.1	226	5	37	99	1	145	87	175	207	72	2	117	144
K17716	Kenya	Africa	2005	skin/soft tissue infection, NOS	emm19.10	769	12	134	100	12	146	13	13	208	8	3	13	14
K17300	Kenya	Africa	2005	skin/soft tissue infection, NOS	ND	450	87	117	3	1	26	13	20	176	65	3	105	124
K19219	Kenya	Africa	2006	skin/soft tissue infection, NOS	emm124.2	231	18	123	84	16	82	13	157	184	8	3	111	145
K50658	Kenya	Africa	2011	ND	emm9.0	740	45	34	40	8	124	19	45	168	62	3	12	120
K24601	Kenya	Africa	2007	skin/soft tissue infection, NOS	emm89.8	772	14	49	33	8	138	50	71	199	18	3	83	126
K24635	Kenya	Africa	2007	skin/soft tissue infection, NOS	emm44.0	178	5	7	7	1	6	7	7	8	14	3	7	7
K25713	Kenya	Africa	2007	skin/soft tissue infection, NOS	emm124.2	280	18	129	101	8	64	50	71	184	8	3	111	132
K26504	Kenya	Africa	2007	skin/soft tissue infection, NOS	emm44.0	178	5	7	7	74	6	7	7	8	14	3	7	7
K28162	Kenya	Africa	2007	skin/soft tissue infection, NOS	emm83.12	393	88	45	3	16	130	85	151	177	60	3	12	30
K29166	Kenya	Africa	2008	invasive, NOS	emm162.1	412	14	119	102	1	134	1	121	140	5	4	95	128

K29527	Kenya	Africa	2008	skin/soft tissue infection, NOS	emm4.5	771	5	65	34	27	39	13	176	192	15	3	35	2
K29655	Kenya	Africa	2008	skin/soft tissue infection, NOS	emm53.4	460	14	135	24	1	147	13	177	23	8	4	95	146
K29743	Kenya	Africa	2008	pneumonia	emm114.5	220	18	136	103	43	148	40	26	56	5	27	83	147
K30067	Kenya	Africa	2008	skin/soft tissue infection, NOS	emm73.0	469	92	137	104	27	149	50	71	209	18	3	83	25
K31063	Kenya	Africa	2008	invasive, NOS	emm209.0	260	14	37	3	27	132	13	153	179	34	2	107	126
K33937	Kenya	Africa	2008	skin/soft tissue infection, NOS	emm60.7	700	90	12	87	68	132	13	153	179	34	3	108	129
K31539	Kenya	Africa	2008	invasive, NOS	emm238.1	713	6	117	3	43	150	57	178	210	8	10	12	28
K31611	Kenya	Africa	2008	skin/soft tissue infection, NOS	emm93.6	613	85	6	6	6	5	6	6	12	5	4	6	11
K32502	Kenya	Africa	2008	skin/soft tissue infection, NOS	emm165.0	768	58	7	56	1	76	88	27	102	8	3	61	29
K33560	Kenya	Africa	2008	meningitis	emm179.0	619	21	111	15	8	46	24	26	30	8	10	12	26
K20641	Kenya	Africa	2006	skin/soft tissue infection, NOS	emm80.0	701	16	16	3	16	17	89	20	22	1	2	18	110
K20653	Kenya	Africa	2007	skin/soft tissue infection, NOS	emm209.0	260	14	37	3	27	132	13	153	179	34	2	107	126
K20746	Kenya	Africa	2007	skin/soft tissue infection, NOS	emm97.1	283	38	45	3	16	44	44	53	61	54	2	12	3
K20747	Kenya	Africa	2007	skin/soft tissue infection, NOS	emm84.1	259	14	130	105	1	142	13	169	201	60	3	115	30
K33951	Kenya	Africa	2008	skin/soft tissue infection, NOS	emm80.0	701	16	16	3	16	17	7	20	22	1	2	18	110
K20882	Kenya	Africa	2007	skin/soft tissue infection, NOS	emm209.0	260	14	37	3	27	132	13	153	179	34	2	107	126
K20910	Kenya	Africa	2007	skin/soft tissue infection, NOS	emm9.0	447	96	65	34	27	39	13	179	183	18	3	50	148
K21246	Kenya	Africa	2007	skin/soft tissue infection, NOS	emm77.0	747	95	127	93	73	2	86	167	197	8	3	20	139
K10332	Kenya	Africa	2003	skin/soft tissue infection, NOS	emm183.2	761	14	25	3	22	27	13	26	174	8	3	25	30
K10474	Kenya	Africa	2003	skin/soft tissue infection, NOS	emm118.2	752	1	124	4	72	138	41	164	188	68	3	12	134
K10586	Kenya	Africa	2003	skin/soft tissue infection, NOS	emm50.3	217	111	138	33	1	151	13	32	211	42	2	118	149
K10722	Kenya	Africa	2003	skin/soft tissue infection, NOS	emm22.5	213	112	37	20	24	64	57	110	128	15	3	18	150
K11116	Kenya	Africa	2003	skin/soft tissue infection, NOS	emm18.21	402	11	13	3	41	50	13	112	42	31	1	51	4
K11239	Kenya	Africa	2003	skin/soft tissue infection, NOS	emm169.1	238	5	37	99	1	152	87	175	207	72	2	117	144
K11243	Kenya	Africa	2003	skin/soft tissue infection, NOS	emm114.5	220	18	136	103	43	148	40	26	56	5	27	83	147
K33983	Kenya	Africa	2008	skin/soft tissue infection, NOS	emm30.15	537	21	21	15	8	46	24	26	30	8	10	12	26
K11319	Kenya	Africa	2003	skin/soft tissue infection, NOS	emm64.3	223	14	122	20	24	64	57	156	128	15	28	110	131
K13065	Kenya	Africa	2004	skin/soft tissue infection, NOS	emm75.1	578	59	74	97	50	71	45	35	183	18	3	18	131
K13107	Kenya	Africa	2004	skin/soft tissue infection, NOS	emm183.2	219	14	25	3	22	27	13	26	29	8	3	25	30
K13190	Kenya	Africa	2004	skin/soft tissue infection, NOS	emm39.4	236	14	139	4	12	12	13	13	212	8	3	13	14
K13372	Kenya	Africa	2004	skin/soft tissue infection, NOS	emm81.2	766	113	140	106	1	26	13	180	213	66	3	12	30
K17276	Kenya	Africa	2005	skin/soft tissue infection, NOS	emm92.0	674	114	65	34	27	39	13	22	152	13	2	20	22
K23685	Kenya	Africa	2007	skin/soft tissue infection, NOS	emm79.5	714	14	25	3	22	27	13	26	29	8	3	25	30
K23745	Kenya	Africa	2007	skin/soft tissue infection, NOS	emm60.7	279	59	74	107	50	71	57	181	214	15	3	18	89
K23866	Kenya	Africa	2007	skin/soft tissue infection, NOS	emm82.5	257	14	141	7	8	2	1	121	23	15	3	18	151
K35870	Kenya	Africa	2008	skin/soft tissue infection, NOS	emm229.0	703	91	9	8	8	136	13	129	182	8	3	106	130
K23890	Kenya	Africa	2007	skin/soft tissue infection, NOS	emm97.1	283	38	45	3	16	44	44	53	61	54	2	12	3
K24146	Kenya	Africa	2007	skin/soft tissue infection, NOS	emm165.0	768	58	7	56	1	76	88	27	102	8	3	61	29
K24190	Kenya	Africa	2007	skin/soft tissue infection, NOS	emm28.0	763	96	65	34	27	39	13	182	215	13	2	20	22
K24525	Kenya	Africa	2007	skin/soft tissue infection, NOS	emm11.0	ND	24	19	78	23	117	83	183	216	8	3	18	4
K19347	Kenya	Africa	2006	skin/soft tissue infection, NOS	ND	258	92	16	3	16	17	24	26	185	60	3	112	133
K19376	Kenya	Africa	2006	skin/soft tissue infection, NOS	emm75.1	578	59	74	97	50	71	45	35	183	18	3	18	131
K19669	Kenya	Africa	2006	skin/soft tissue infection, NOS	ND	258	92	16	3	16	17	24	26	217	73	3	112	133
K35909	Kenya	Africa	2009	skin/soft tissue infection, NOS	emm75.1	704	14	33	18	6	25	27	40	37	18	3	28	30
K50593	Kenya	Africa	2011	pneumonia	emm9.0	740	45	34	40	8	124	19	45	168	62	3	12	120
K19875	Kenya	Africa	2006	skin/soft tissue infection, NOS	emm111.2	256	14	37	88	16	12	90	26	29	69	3	109	89
K19912	Kenya	Africa	2006	skin/soft tissue infection, NOS	emm92.0	674	114	65	34	27	39	13	22	152	13	2	20	22
K19952	Kenya	Africa	2006	skin/soft tissue infection, NOS	emm92.0	674	114	65	34	27	39	13	22	152	13	2	20	22

K20201	Kenya	Africa	2006	skin/soft tissue infection, NOS	emm44.0	178	5	7	7	1	6	7	7	8	14	3	7	7
K36395	Kenya	Africa	2009	skin/soft tissue infection, NOS	emm81.2	766	113	140	106	1	26	13	180	213	66	3	12	30
K35215	Kenya	Africa	2008	skin/soft tissue infection, NOS	ND	450	87	117	3	1	26	13	20	176	65	3	105	124
K36067	Kenya	Africa	2009	ND	emm85.1	774	14	125	90	71	139	9	161	189	11	3	113	25
K50105	Kenya	Africa	2011	skin/soft tissue infection, NOS	emm22.5	213	112	37	20	24	64	57	110	128	15	3	18	150
K31028	Kenya	Africa	2008	skin/soft tissue infection, NOS	emm93.6	613	85	6	6	6	5	6	6	12	5	4	6	11
K35129	Kenya	Africa	2008	skin/soft tissue infection, NOS	ND	258	92	16	3	16	17	24	26	218	60	3	112	133
K27345	Kenya	Africa	2007	skin/soft tissue infection, NOS	ND	450	87	117	3	1	26	13	20	176	65	3	105	124
K28044	Kenya	Africa	2007	pneumonia	emm25.1	323	14	7	98	2	2	13	26	202	8	3	116	140
K19417	Kenya	Africa	2006	skin/soft tissue infection, NOS	emm122.0	200	11	13	3	41	50	13	112	42	31	1	51	4
K50316	Kenya	Africa	2011	skin/soft tissue infection, NOS	emm64.3	223	14	122	20	24	64	57	156	128	15	3	110	131
K7541	Kenya	Africa	2001	skin/soft tissue infection, NOS	emm81.2	766	113	140	106	1	26	13	180	213	66	3	12	30
K7114	Kenya	Africa	2001	skin/soft tissue infection, NOS	ND	450	87	117	3	1	26	13	20	176	65	3	105	124
K7151	Kenya	Africa	2001	skin/soft tissue infection, NOS	emm183.2	761	14	25	3	22	27	13	26	174	8	3	25	30
K8955	Kenya	Africa	2002	skin/soft tissue infection, NOS	emm65.0	778	5	65	34	27	39	[S]	158	186	15	3	35	2
K9333	Kenya	Africa	2002	skin/soft tissue infection, NOS	emm90.5	708	14	115	85	2	127	13	149	171	8	3	12	122
K10016	Kenya	Africa	2003	skin/soft tissue infection, NOS	emm103.0	233	115	79	91	1	26	24	26	196	65	3	105	124
K10021	Kenya	Africa	2003	skin/soft tissue infection, NOS	emm103.0	233	63	79	108	1	26	24	26	219	65	3	105	124
K9408	Kenya	Africa	2002	skin/soft tissue infection, NOS	emm65.0	778	5	65	34	27	39	13	158	186	15	3	35	2
K10040	Kenya	Africa	2003	skin/soft tissue infection, NOS	emm22.5	213	112	37	20	24	64	57	110	128	15	3	18	150
K7829	Kenya	Africa	2001	skin/soft tissue infection, NOS	emm186.1	262	14	44	24	2	12	41	154	180	74	3	101	118
K10105	Kenya	Africa	2003	skin/soft tissue infection, NOS	emm22.5	213	112	37	20	24	64	57	110	128	15	3	18	150
K10712	Kenya	Africa	2003	invasive, NOS	emm183.2	219	14	25	3	22	27	13	26	29	8	3	25	30
K5086	Kenya	Africa	1999	invasive, NOS	emm238.1	713	6	117	3	43	150	57	178	210	8	10	12	28
K10987	Kenya	Africa	2003	pneumonia	emm230.1	755	14	142	24	1	153	13	177	23	5	4	95	146
K10238	Kenya	Africa	2003	APSGN	emm218.1	292	38	45	3	16	44	44	53	61	54	2	12	3
K9440	Kenya	Africa	2002	invasive, NOS	emm99.5	779	14	41	92	1	20	40	26	56	5	4	20	46
K19639	Kenya	Africa	2006	invasive, NOS	emm111.1	496	11	11	109	10	10	7	77	39	19	2	30	152
K17494	Kenya	Africa	2005	invasive, NOS	emm55.0	248	11	11	83	10	10	13	148	13	8	3	11	12
K7908	Kenya	Africa	2001	skin/soft tissue infection, NOS	emm65.4	1031	11	66	40	35	68	76	44	145	1	2	87	31
K7175	Kenya	Africa	2001	invasive, NOS	emm165.0	762	59	74	97	50	71	45	35	183	5	4	20	46
K18724	Kenya	Africa	2006	pneumonia	emm11.0	250	92	128	3	26	141	24	168	198	70	3	83	28
K7554	Kenya	Africa	2001	invasive, NOS	emm90.5	708	14	143	85	2	127	13	67	171	8	3	12	122
K10697	Kenya	Africa	2003	invasive, NOS	emm18.21	221	11	114	84	16	82	13	20	170	63	3	13	30
K5192	Kenya	Africa	1999	invasive, NOS	emm147.0	741	88	45	3	16	130	85	151	177	60	3	12	30
K4761	Kenya	Africa	1999	pneumonia	emm8.3	730	89	120	3	1	135	45	103	181	66	3	12	30
K16772	Kenya	Africa	2005	invasive, NOS	emm89.8	245	14	49	33	8	138	50	71	199	18	3	83	126
K3573	Kenya	Africa	1998	invasive, NOS	emm116.2	702	14	119	24	1	153	13	177	23	75	4	95	146
K16738	Kenya	Africa	2005	invasive, NOS	emm64.3	223	14	122	20	24	64	57	156	128	76	28	110	131
K7559	Kenya	Africa	2001	invasive, NOS	emm110.0	767	84	144	110	27	154	33	144	220	1	3	119	28
K3525	Kenya	Africa	1998	invasive, NOS	emm55.0	100	11	11	83	10	10	13	[S]	13	8	3	11	12
K3879	Kenya	Africa	1998	invasive, NOS	emm183.2	714	14	25	3	22	27	13	26	29	8	3	25	30
K19873	Kenya	Africa	2006	meningitis	emm78.5	255	86	34	40	8	128	19	45	173	62	3	12	120
K4973	Kenya	Africa	1999	invasive, NOS	emm25.4	735	116	145	111	23	155	92	185	221	77	2	120	3
K8861	Kenya	Africa	2002	invasive, NOS	emm44.0	178	5	7	7	1	6	7	7	8	14	3	7	7
K13045	Kenya	Africa	2004	pneumonia	emm192.0	261	14	44	24	2	12	41	154	180	8	3	112	3
K12183	Kenya	Africa	2004	invasive, NOS	emm124.2	231	18	123	84	16	82	13	157	184	8	3	111	132
K37914	Kenya	Africa	2009	ND	emm119.2	239	38	45	3	16	44	44	53	61	54	2	12	3

K4728	Kenya	Africa	1999	pneumonia	emm44.0	178	5	7	7	1	6	7	7	8	14	3	7	7
K9679	Kenya	Africa	2002	pneumonia	emm112.5	777	96	131	3	71	143	13	11	13	8	3	11	153
K17786	Kenya	Africa	2006	meningitis	emm11.0	251	92	128	3	26	141	24	168	198	[S]	3	83	28
K3730	Kenya	Africa	1998	invasive, NOS	emm90.5	708	14	115	85	2	127	13	67	171	8	3	12	122
K10378	Kenya	Africa	2003	invasive, NOS	emm50.3	217	111	138	33	1	151	13	32	211	42	2	118	149
K8460	Kenya	Africa	2002	invasive, NOS	emm82.5	257	14	141	7	8	2	1	121	23	15	3	18	151
K12669	Kenya	Africa	2004	pneumonia	emm183.2	234	19	25	3	22	27	13	26	29	8	3	25	30
K5851	Kenya	Africa	2000	invasive, NOS	emm230.1	755	14	142	24	1	156	13	177	23	[S]	4	95	146
K7792	Kenya	Africa	2001	skin/soft tissue infection, NOS	emm43.1	770	16	16	3	16	17	7	20	222	1	2	18	3
K9454	Kenya	Africa	2002	invasive, NOS	emm50.3	217	111	138	33	1	151	13	32	211	42	2	118	149
K9612	Kenya	Africa	2002	pneumonia	emm99.5	781	14	41	92	1	20	40	26	56	5	4	20	46
K21633	Kenya	Africa	2007	invasive, NOS	emm15.1	266	117	146	20	24	64	2	67	223	66	3	12	174
K7219	Kenya	Africa	2001	invasive, NOS	emm28.0	763	96	65	34	27	39	13	182	215	13	2	20	22
K10676	Kenya	Africa	2003	invasive, NOS	emm103.0	233	63	79	91	1	26	24	26	196	65	3	105	124
K10167	Kenya	Africa	2003	pneumonia	emm98.3	136	118	44	24	75	12	41	154	180	8	3	101	118
K8015	Kenya	Africa	2001	skin/soft tissue infection, NOS	emm99.5	781	14	41	92	1	20	40	26	56	5	4	20	175
K9521	Kenya	Africa	2002	pneumonia	emm84.1	780	14	130	112	1	142	13	186	75	80	2	121	2
K5679	Kenya	Africa	2000	pneumonia	emm177.0	743	84	113	21	23	138	13	147	169	1	2	104	121
K7393	Kenya	Africa	2001	invasive, NOS	emm63.5	764	14	116	86	27	129	9	150	175	64	2	12	123
K6222	Kenya	Africa	2000	invasive, NOS	emm70.0	754	14	147	3	26	157	50	187	224	60	3	115	30
K17097	Kenya	Africa	2005	pneumonia	emm223.0	536	85	6	6	6	5	6	6	12	5	4	6	11
K9215	Kenya	Africa	2002	meningitis	emm112.5	777	96	131	3	71	143	13	11	13	8	3	11	153
K12554	Kenya	Africa	2004	invasive, NOS	emm44.0	178	5	7	7	1	6	7	7	8	14	3	7	7
K13484	Kenya	Africa	2004	pneumonia	emm119.2	239	38	45	3	16	44	44	53	61	54	2	12	3
K3944	Kenya	Africa	1998	invasive, NOS	emm122.0	200	11	13	3	41	50	13	112	42	31	1	51	4
K10311	Kenya	Africa	2003	invasive, NOS	emm50.3	217	111	138	33	1	151	13	32	211	42	2	118	149
K10812	Kenya	Africa	2003	pneumonia	emm82.5	257	14	141	7	8	2	1	121	23	15	3	18	151
K7846	Kenya	Africa	2001	invasive, NOS	emm74.0	120	11	13	3	41	50	13	112	195	5	4	95	138
K3589	Kenya	Africa	1998	meningitis	emm8.3	241	89	120	3	1	135	45	103	181	66	3	12	30
K8543	Kenya	Africa	2002	invasive, NOS	emm85.1	774	14	125	90	71	139	9	161	189	11	3	113	25
K8057	Kenya	Africa	2001	pneumonia	emm179.0	619	21	111	15	8	158	24	26	225	8	10	12	26
K11254	Kenya	Africa	2003	invasive, NOS	emm18.21	221	11	114	84	16	82	13	20	170	60	3	13	30
K11271	Kenya	Africa	2003	pneumonia	emm25.4	222	116	145	111	23	155	92	185	221	77	2	120	3
K9037	Kenya	Africa	2002	invasive, NOS	emm239.1	776	63	79	91	1	26	13	20	176	65	3	105	124
K4350	Kenya	Africa	1999	invasive, NOS	emm22.5	213	112	37	20	24	64	57	110	128	15	3	18	150
K7736	Kenya	Africa	2001	APSGN	emm19.10	769	12	134	100	12	146	13	13	208	8	3	13	14
K7731	Kenya	Africa	2001	invasive, NOS	emm100.2	773	93	25	3	26	71	45	162	190	60	26	114	135
K14810	Kenya	Africa	2005	invasive, NOS	emm18.21	402	11	13	3	41	50	13	112	42	31	1	51	4
K10213	Kenya	Africa	2003	skin/soft tissue infection, NOS	emm169.1	238	5	37	99	1	145	87	175	207	72	2	117	144
K3808	Kenya	Africa	1998	skin/soft tissue infection, NOS	emm95.0	712	85	6	6	6	5	6	6	226	5	4	6	176
K20001	Kenya	Africa	2006	invasive, NOS	emm77.0	747	95	127	93	73	2	86	167	197	8	3	20	139
K3828	Kenya	Africa	1998	skin/soft tissue infection, NOS	emm238.1	713	6	117	3	43	150	57	178	210	8	10	12	28
K3964	Kenya	Africa	1999	skin/soft tissue infection, NOS	emm65.0	716	14	34	81	27	122	13	142	164	15	3	100	117
K3997	Kenya	Africa	1999	skin/soft tissue infection, NOS	emm65.0	716	14	34	81	27	122	13	142	164	15	3	100	117
K4134	Kenya	Africa	1999	invasive, NOS	emm209.1	717	1	148	115	1	159	13	188	227	15	2	122	177
K4240	Kenya	Africa	1999	skin/soft tissue infection, NOS	emm74.0	720	11	13	3	41	50	13	112	195	5	4	95	138
K4263	Kenya	Africa	1999	skin/soft tissue infection, NOS	emm230.1	755	14	142	24	1	153	13	177	23	5	4	95	146
K4656	Kenya	Africa	1999	skin/soft tissue infection, NOS	emm92.1	727	5	65	34	27	150	57	189	[S]	18	3	18	178

K4819	Kenya	Africa	1999	skin/soft tissue infection, NOS	emm230.1	755	14	142	24	1	153	13	177	23	5	4	95	146
K4982	Kenya	Africa	1999	skin/soft tissue infection, NOS	emm124.2	736	18	129	94	16	64	50	71	184	8	3	111	132
K5460	Kenya	Africa	2000	skin/soft tissue infection, NOS	emm11.0	742	92	128	3	26	141	24	168	198	70	3	83	28
K8396	Kenya	Africa	2002	invasive, NOS	emm89.8	772	14	49	33	8	138	50	71	199	18	3	83	126
K5660	Kenya	Africa	2000	skin/soft tissue infection, NOS	ND	1085	87	117	3	1	26	13	20	176	65	3	105	124
K5690	Kenya	Africa	2000	skin/soft tissue infection, NOS	emm81.11	744	8	4	4	4	160	13	4	228	31	29	4	4
K5727	Kenya	Africa	2000	skin/soft tissue infection, NOS	emm25.1	746	14	7	117	76	55	13	13	40	36	3	83	179
K8492	Kenya	Africa	2002	skin/soft tissue infection, NOS	emm100.2	773	93	25	3	26	71	45	162	190	60	26	114	135
K8744	Kenya	Africa	2002	skin/soft tissue infection, NOS	emm8.3	241	89	120	3	1	135	45	190	181	66	3	12	30
K5797	Kenya	Africa	2000	skin/soft tissue infection, NOS	emm77.0	747	95	127	93	73	2	86	167	197	8	3	20	139
K5898	Kenya	Africa	2000	skin/soft tissue infection, NOS	emm209.1	749	1	148	115	1	159	13	188	227	15	2	122	177
K5910	Kenya	Africa	2000	skin/soft tissue infection, NOS	emm92.1	750	5	65	34	27	150	57	189	229	18	3	18	178
K5965	Kenya	Africa	2000	skin/soft tissue infection, NOS	emm147.0	753	88	45	3	16	130	85	151	177	60	3	12	30
K6008	Kenya	Africa	2000	skin/soft tissue infection, NOS	emm75.1	751	119	37	3	43	161	19	107	230	9	11	57	120
K6038	Kenya	Africa	2000	skin/soft tissue infection, NOS	emm118.2	752	1	124	4	72	138	41	164	188	68	3	12	134
K6048	Kenya	Africa	2000	skin/soft tissue infection, NOS	emm147.0	753	88	45	3	16	130	85	151	177	60	3	12	30
K6085	Kenya	Africa	2000	skin/soft tissue infection, NOS	emm8.3	241	89	120	3	1	135	45	191	181	66	3	12	30
K6099	Kenya	Africa	2000	skin/soft tissue infection, NOS	emm77.0	482	14	110	3	22	27	13	26	29	8	3	25	30
K6363	Kenya	Africa	2000	skin/soft tissue infection, NOS	emm230.1	755	14	142	24	77	153	13	177	23	5	4	95	146
K6428	Kenya	Africa	2000	skin/soft tissue infection, NOS	emm97.1	756	21	21	15	8	46	24	26	231	8	10	12	26
K7498	Kenya	Africa	2001	invasive, NOS	emm44.0	178	5	7	7	1	6	7	7	8	14	3	7	7
K6917	Kenya	Africa	2001	skin/soft tissue infection, NOS	emm65.5	215	1	112	24	1	2	13	143	165	8	3	101	118
K4828	Kenya	Africa	1999	invasive, NOS	emm179.0	619	21	111	15	8	162	24	26	30	8	10	12	26
K8728	Kenya	Africa	2002	invasive, NOS	emm64.3	223	14	122	20	24	64	57	156	128	15	3	110	131
K13179	Kenya	Africa	2004	invasive, NOS	ND	235	6	118	71	1	163	13	152	232	8	3	106	180
K12537	Kenya	Africa	2004	meningitis	emm103.0	233	63	79	91	1	26	24	26	196	65	3	105	124
K23182	Kenya	Africa	2007	pneumonia	emm63.5	274	59	74	97	50	71	45	35	183	81	3	18	154
K19961	Kenya	Africa	2006	pneumonia	emm82.5	257	14	141	7	8	2	1	121	23	15	3	18	151
K5017	Kenya	Africa	1999	meningitis	emm44.0	178	5	7	7	1	6	7	7	8	14	3	7	7
K10514	Kenya	Africa	2003	invasive, NOS	emm77.0	218	14	110	3	22	27	13	26	29	8	3	25	30
K12614	Kenya	Africa	2004	invasive, NOS	emm179.0	619	21	111	15	8	46	24	26	30	8	10	12	26
K9361	Kenya	Africa	2002	pneumonia	emm44.0	178	5	7	7	1	6	7	7	8	14	3	7	7
K13389	Kenya	Africa	2004	invasive, NOS	emm169.1	238	5	37	99	1	145	87	175	207	72	2	117	144
K9189	Kenya	Africa	2002	pneumonia	emm182.1	229	14	41	120	1	164	40	26	56	5	4	123	46
K6613	Kenya	Africa	2000	invasive, NOS	emm218.1	292	38	45	3	16	44	44	53	61	54	2	12	3
SP10LAU	Lebanon	Asia	2011	pharyngitis and/or tonsillitis	emm118.0	167	11	32	52	1	67	13	118	135	1	2	83	93
SP1LAU	Lebanon	Asia	2010	pharyngitis and/or tonsillitis	emm12.0	36	14	3	3	3	2	3	52	59	24	12	38	3
SP2LAU	Lebanon	Asia	2010	skin/soft tissue infection, NOS	emm108.0	304	11	109	10	20	15	16	17	13	59	2	16	31
SP3LAU	Lebanon	Asia	2010	pharyngitis and/or tonsillitis	emm89.0	101	2	2	2	2	2	2	2	2	2	2	2	2
SP4LAU	Lebanon	Asia	2010	pharyngitis and/or tonsillitis	emm28.0	52	3	3	3	3	2	3	3	4	1	1	3	3
SP5LAU	Lebanon	Asia	2011	skin/soft tissue infection, NOS	emm1.0	28	1	1	1	1	1	1	1	1	1	1	1	1
SP6LAU	Lebanon	Asia	2011	pharyngitis and/or tonsillitis	emm89.0	101	2	2	2	2	2	2	2	2	2	2	2	2
SP7LAU	Lebanon	Asia	2011	pharyngitis and/or tonsillitis	emm22.0	46	7	149	5	5	4	5	5	233	4	3	5	5
SP8LAU	Lebanon	Asia	2011	pharyngitis and/or tonsillitis	emm85.0	109	10	10	4	9	9	11	72	11	7	3	10	10
HSC5	Unknown		ND	ND	emm14.3	84	11	5	10	8	15	16	184	13	1	2	6	20
M23ND	Unknown		1965	invasive, NOS	emm23.0	160	120	150	121	54	106	62	192	234	11	4	35	155
GAS13507	New Zealand	Oceania	2013	ARF	emm25.0	191	30	35	26	28	35	34	42	45	11	2	12	40
15GA0017	New Zealand	Oceania	2015	ARF	emm41.2	579	11	5	10	51	15	16	77	99	1	2	6	20

14GA0838	New Zealand	Oceania	2014	skin/soft tissue infection, NOS	emm81.0	330	62	75	45	52	5	6	45	106	24	17	66	61
GAS14243	New Zealand	Oceania	2014	ARF	emm12.0	36	14	3	3	3	2	3	52	59	24	12	38	3
GAS131474	New Zealand	Oceania	2013	pharyngitis and/or tonsillitis	emm53.0	11	121	5	10	80	15	54	17	84	1	2	6	20
GAS131267	New Zealand	Oceania	2013	pharyngitis and/or tonsillitis	emm74.0	120	28	13	3	41	50	53	123	82	11	4	59	70
GAS131159	New Zealand	Oceania	2013	ARF	emm70.0	1003	16	16	3	16	17	7	193	22	1	2	18	3
GAS13502	New Zealand	Oceania	2013	invasive, NOS	emm116.1	227	28	105	32	16	111	63	87	235	51	4	11	77
15GA0011	New Zealand	Oceania	2015	invasive, NOS	emm41.2	579	11	5	10	51	15	16	77	99	1	2	6	20
14GA0832	New Zealand	Oceania	2014	invasive, NOS	emm81.0	330	62	75	45	52	5	6	45	106	24	17	66	61
GAS14211	New Zealand	Oceania	2014	invasive, NOS	emm116.1	227	28	105	32	16	111	63	87	154	51	4	11	77
GAS131472	New Zealand	Oceania	2013	pharyngitis and/or tonsillitis	emm100.0	119	11	11	7	10	10	7	37	39	19	2	30	31
GAS131255	New Zealand	Oceania	2013	pharyngitis and/or tonsillitis	emm58.2	176	14	13	10	8	13	13	194	47	10	2	34	31
GAS131153	New Zealand	Oceania	2013	pharyngitis and/or tonsillitis	emm92.0	82	17	4	3	18	19	19	22	24	13	2	20	22
GAS13480	New Zealand	Oceania	2013	ARF	emm116.1	227	28	105	32	16	111	63	87	154	51	4	11	77
14GA0830	New Zealand	Oceania	2014	ND	emm81.0	330	62	75	45	52	5	6	45	106	24	17	66	61
14GA1137	New Zealand	Oceania	2014	ARF	emm41.2	12	11	5	10	15	15	16	17	13	1	2	16	18
GAS131465	New Zealand	Oceania	2013	ARF	emm53.0	11	121	5	10	8	15	54	17	84	1	2	6	20
GAS131252	New Zealand	Oceania	2013	ARF	emm74.0	120	28	13	3	41	50	53	123	82	11	4	59	70
GAS131142	New Zealand	Oceania	2013	pharyngitis and/or tonsillitis	emm76.0	378	122	3	3	3	2	3	52	59	24	12	38	3
GAS13476	New Zealand	Oceania	2013	ARF	emm19.4	616	50	32	10	1	6	55	78	13	1	2	6	106
14GA0829	New Zealand	Oceania	2014	ND	emm81.0	330	62	75	45	52	5	6	45	106	24	17	66	61
14GA1136	New Zealand	Oceania	2014	ARF	emm41.2	579	11	5	10	51	15	16	77	99	1	2	6	20
GAS131460	New Zealand	Oceania	2013	ARF	emm76.0	378	122	3	3	3	2	3	52	59	24	12	38	3
GAS131245	New Zealand	Oceania	2013	pharyngitis and/or tonsillitis	emm89.0	101	123	2	2	58	2	2	2	2	2	2	2	2
GAS131141	New Zealand	Oceania	2013	pharyngitis and/or tonsillitis	emm25.0	191	30	35	26	28	35	34	42	45	11	2	12	40
GAS13475	New Zealand	Oceania	2013	ARF	emm197.0	998	11	151	10	8	165	16	17	236	1	2	6	20
14GA0828	New Zealand	Oceania	2014	skin/soft tissue infection, NOS	emm81.0	330	62	75	45	52	5	6	45	106	24	17	66	61
14GA0305	New Zealand	Oceania	2014	invasive, NOS	emm103.0	876	82	25	3	64	107	13	26	29	8	3	25	30
14GA1101	New Zealand	Oceania	2014	invasive, NOS	emm118.0	997	11	32	52	1	67	13	118	237	1	2	83	93
GAS131421	New Zealand	Oceania	2013	invasive, NOS	emm74.0	120	28	13	75	41	50	53	123	82	11	4	59	70
GAS131241	New Zealand	Oceania	2013	ARF	emm58.2	176	14	13	10	8	13	13	194	47	10	2	34	31
GAS131138	New Zealand	Oceania	2013	invasive, NOS	emm74.0	120	28	13	3	41	50	53	123	82	11	4	59	70
GAS13474	New Zealand	Oceania	2013	ARF	emm95.0	604	11	32	30	31	37	7	46	51	1	2	6	44
14GA0826	New Zealand	Oceania	2014	invasive, NOS	emm81.0	330	62	75	45	52	5	6	45	106	24	17	66	61
14GA0287	New Zealand	Oceania	2014	ARF	emm74.0	120	28	13	3	41	50	53	123	238	11	4	59	70
GAS1464	New Zealand	Oceania	2014	pharyngitis and/or tonsillitis	emm58.7	1004	14	13	10	8	13	13	195	239	10	2	34	31
GAS131413	New Zealand	Oceania	2013	pharyngitis and/or tonsillitis	emm76.0	378	122	3	3	3	2	3	52	59	24	12	38	3
GAS131223	New Zealand	Oceania	2013	ARF	emm74.0	120	28	13	3	41	50	53	123	82	11	4	59	70
GAS131111	New Zealand	Oceania	2013	pharyngitis and/or tonsillitis	emm74.0	120	28	13	3	41	50	53	123	82	11	4	59	70
GAS13472	New Zealand	Oceania	2013	invasive, NOS	emm70.0	1003	16	16	3	16	17	7	193	22	1	2	18	3
14GA0809	New Zealand	Oceania	2014	ARF	emm59.1	172	71	84	24	2	93	68	107	122	18	3	75	85
14GA0286	New Zealand	Oceania	2014	ARF	emm103.0	876	82	25	3	64	107	13	26	29	8	3	25	30
14GA1044	New Zealand	Oceania	2014	invasive, NOS	emm86.2	963	11	32	10	20	15	16	128	13	1	2	6	100
GAS131348	New Zealand	Oceania	2013	invasive, NOS	emm53.0	11	49	5	10	8	15	54	17	84	1	2	6	20
GAS131222	New Zealand	Oceania	2013	pharyngitis and/or tonsillitis	emm81.0	330	62	75	45	52	5	6	45	106	24	17	66	61
GAS131103	New Zealand	Oceania	2013	invasive, NOS	emm65.0	111	22	22	17	38	166	43	28	65	1	1	1	27
GAS13450	New Zealand	Oceania	2013	invasive, NOS	emm19.4	616	50	32	10	1	6	55	78	13	1	2	6	106
14GA0958	New Zealand	Oceania	2014	invasive, NOS	emm90.5	184	20	20	14	19	2	23	25	28	15	3	22	25
14GA0794	New Zealand	Oceania	2014	pharyngitis and/or tonsillitis	emm74.0	120	28	13	3	41	50	53	123	82	11	4	59	70

GAS14274	New Zealand	Oceania	2014	invasive, NOS	emm95.0	604	11	32	30	31	37	7	46	51	1	2	6	44
GAS1441	New Zealand	Oceania	2014	invasive, NOS	emm12.0	36	14	3	3	3	2	3	52	59	24	12	38	3
GAS131322	New Zealand	Oceania	2013	invasive, NOS	emm108.1	14	28	87	10	1	6	63	78	240	1	2	6	31
GAS131204	New Zealand	Oceania	2013	invasive, NOS	emm81.0	330	62	75	45	52	5	6	45	106	24	17	66	61
GAS131015	New Zealand	Oceania	2013	pharyngitis and/or tonsillitis	emm65.0	111	22	22	17	38	23	43	28	65	1	1	1	27
GAS13432	New Zealand	Oceania	2013	pharyngitis and/or tonsillitis	ND	616	50	32	10	1	6	55	78	13	1	2	6	106
GAS13426	New Zealand	Oceania	2013	pharyngitis and/or tonsillitis	emm116.1	227	28	105	32	16	111	63	87	154	51	4	11	77
GAS13118	New Zealand	Oceania	2013	pharyngitis and/or tonsillitis	emm74.0	120	28	13	3	41	50	53	123	82	11	4	59	70
GAS12394	New Zealand	Oceania	2012	invasive, NOS	emm41.2	579	11	5	10	51	15	16	77	99	1	2	6	20
GAS12235	New Zealand	Oceania	2012	invasive, NOS	emm89.0	101	2	2	2	58	2	2	2	2	2	2	2	2
GAS12118	New Zealand	Oceania	2012	pharyngitis and/or tonsillitis	emm89.0	101	2	2	2	2	2	2	2	2	2	2	2	2
GAS11549	New Zealand	Oceania	2011	ARF	emm71.0	981	19	20	13	19	2	22	3	27	1	1	4	24
GAS11291	New Zealand	Oceania	2011	ARF	emm11.0	ND	1	6	76	1	104	20	13	25	8	3	21	23
GAS11117	New Zealand	Oceania	2011	ARF	emm68.0	993	81	103	33	63	105	7	130	150	34	3	90	104
GAS13409	New Zealand	Oceania	2013	ARF	emm19.4	616	50	32	10	1	6	55	78	13	1	2	6	106
GAS1396	New Zealand	Oceania	2013	pharyngitis and/or tonsillitis	emm116.1	227	28	105	32	16	111	63	87	241	51	4	11	77
GAS12392	New Zealand	Oceania	2012	pharyngitis and/or tonsillitis	emm59.1	172	71	84	24	2	93	68	107	122	18	3	75	85
GAS12233	New Zealand	Oceania	2012	invasive, NOS	emm1.0	28	1	1	1	1	1	1	1	3	1	1	1	1
GAS12107	New Zealand	Oceania	2012	ARF	emm218.1	292	38	45	3	16	44	93	53	61	54	2	12	3
14GA0862	New Zealand	Oceania	2014	invasive, NOS	emm85.0	109	10	10	4	9	9	11	72	11	7	3	10	10
14GA0793	New Zealand	Oceania	2014	pharyngitis and/or tonsillitis	emm59.1	172	71	84	24	2	93	68	107	122	18	3	75	85
GAS14267	New Zealand	Oceania	2014	invasive, NOS	emm54.1	990	11	5	10	20	15	16	17	13	1	2	16	31
GAS1414	New Zealand	Oceania	2014	pharyngitis and/or tonsillitis	emm90.5	184	20	20	14	19	2	23	25	28	15	3	22	25
GAS131318	New Zealand	Oceania	2013	ARF	emm108.1	14	28	87	10	1	6	63	78	240	1	2	6	31
GAS131202	New Zealand	Oceania	2013	ARF	emm232.0	902	14	37	31	3	2	38	47	242	1	30	58	25
GAS13637	New Zealand	Oceania	2013	ARF	emm65.4	129	11	66	40	35	102	76	44	145	1	2	87	31
14GA0857	New Zealand	Oceania	2014	invasive, NOS	emm81.0	330	62	75	45	52	5	6	45	106	24	17	66	61
14GA0788	New Zealand	Oceania	2014	ARF	emm85.0	109	10	10	4	9	9	11	72	11	7	3	10	10
GAS14266	New Zealand	Oceania	2014	pharyngitis and/or tonsillitis	emm118.0	997	11	32	52	1	67	13	118	237	1	2	83	93
GAS146	New Zealand	Oceania	2014	pharyngitis and/or tonsillitis	emm12.0	36	14	3	3	3	2	3	52	243	24	12	38	156
GAS131286	New Zealand	Oceania	2013	invasive, NOS	emm89.0	101	2	2	2	2	2	2	2	2	2	2	2	2
GAS131175	New Zealand	Oceania	2013	invasive, NOS	emm74.0	120	28	13	3	41	50	53	123	82	11	4	59	70
GAS13599	New Zealand	Oceania	2013	invasive, NOS	emm41.2	579	11	5	10	51	15	16	77	99	1	2	6	20
15GAS0097	New Zealand	Oceania	2015	ND	emm95.0	604	11	32	30	31	37	7	46	51	1	2	6	44
14GA0844	New Zealand	Oceania	2014	pharyngitis and/or tonsillitis	emm91.0	12	11	5	10	15	15	16	17	13	1	2	16	18
GAS14265	New Zealand	Oceania	2014	ARF	emm86.2	963	11	32	10	20	15	16	128	13	1	2	6	100
14GA1005	New Zealand	Oceania	2014	pharyngitis and/or tonsillitis	emm1.0	28	1	1	1	1	1	1	1	3	1	1	1	1
GAS131282	New Zealand	Oceania	2013	ARF	emm89.0	101	2	2	2	2	2	2	2	2	2	2	2	2
GAS131172	New Zealand	Oceania	2013	ARF	emm74.0	120	28	13	3	41	50	53	123	82	11	4	59	70
GAS13589	New Zealand	Oceania	2013	ARF	emm41.2	579	11	5	10	51	15	16	77	99	1	2	6	20
15GAS0096	New Zealand	Oceania	2015	ND	emm90.5	184	20	20	14	19	2	23	25	28	15	3	22	25
14GA0839	New Zealand	Oceania	2014	ND	emm85.0	109	10	10	4	9	9	11	72	244	7	3	10	10
GAS14246	New Zealand	Oceania	2014	invasive, NOS	emm86.2	963	11	32	10	20	15	16	128	13	1	2	6	100
GAS131487	New Zealand	Oceania	2013	ARF	emm74.0	1011	11	13	122	81	167	13	196	42	31	1	51	4
GAS131275	New Zealand	Oceania	2013	pharyngitis and/or tonsillitis	emm77.0	63	23	24	19	1	26	28	30	33	8	3	24	29
GAS131166	New Zealand	Oceania	2013	invasive, NOS	emm58.2	176	14	13	10	8	13	13	194	47	10	2	34	31
GAS11493	New Zealand	Oceania	2011	invasive, NOS	emm19.4	616	50	32	10	1	6	55	78	13	1	2	6	106
GAS11282	New Zealand	Oceania	2011	invasive, NOS	emm100.5	119	11	11	7	10	10	7	37	39	19	2	30	31

GAS10411	New Zealand	Oceania	2010	pharyngitis and/or tonsillitis	emm91.0	12	11	5	10	15	15	16	17	13	1	2	16	18
GAS10213	New Zealand	Oceania	2010	ARF	emm36.2	748	15	15	10	1	10	32	197	21	12	8	37	31
GAS1073	New Zealand	Oceania	2010	invasive, NOS	emm36.2	748	15	15	10	1	10	32	197	21	12	8	37	31
GAS09398	New Zealand	Oceania	2009	pharyngitis and/or tonsillitis	emm12.0	36	14	3	3	3	2	3	52	245	24	12	38	3
GAS09190	New Zealand	Oceania	2009	pharyngitis and/or tonsillitis	emm87.0	62	67	15	10	1	72	58	87	95	1	2	52	74
GAS07167	New Zealand	Oceania	2007	ARF	emm6.0	411	65	80	62	55	85	62	97	130	11	20	69	82
GAS05161	New Zealand	Oceania	2005	ARF	emm98.1	205	15	15	10	1	10	94	19	246	12	2	6	20
GAS0387	New Zealand	Oceania	2003	invasive, NOS	emm108.1	14	28	87	10	1	6	63	78	240	1	2	6	31
GAS10367	New Zealand	Oceania	2010	pharyngitis and/or tonsillitis	emm22.0	46	7	5	5	5	4	5	5	6	4	3	5	5
GAS10210	New Zealand	Oceania	2010	invasive, NOS	emm87.0	62	67	15	10	1	72	58	87	247	23	2	52	74
GAS1186	New Zealand	Oceania	2011	invasive, NOS	emm65.4	129	11	66	40	35	102	76	44	145	1	2	87	31
GAS1050	New Zealand	Oceania	2010	invasive, NOS	emm68.0	993	81	103	33	63	105	7	130	150	34	3	90	104
GAS09379	New Zealand	Oceania	2009	ARF	emm36.2	748	15	15	10	1	10	32	197	21	12	8	37	31
GAS09155	New Zealand	Oceania	2009	ARF	emm65.4	129	11	66	40	35	102	76	44	145	1	2	87	31
GAS07147	New Zealand	Oceania	2007	pharyngitis and/or tonsillitis	emm44.0	178	5	7	7	3	2	3	24	8	14	3	7	7
GAS05154	New Zealand	Oceania	2005	invasive, NOS	emm53.0	11	49	5	10	8	15	54	17	84	1	2	6	20
GAS0371	New Zealand	Oceania	2003	pharyngitis and/or tonsillitis	emm82.0	334	73	58	46	1	6	18	69	77	1	1	1	3
GAS10358	New Zealand	Oceania	2010	ARF	emm53.0	11	49	5	10	8	15	54	17	84	1	2	6	20
GAS10197	New Zealand	Oceania	2010	ARF	emm113.0	148	14	3	3	3	2	3	52	59	24	12	38	3
GAS1047	New Zealand	Oceania	2010	ARF	emm36.2	748	15	15	10	1	10	32	197	21	12	8	37	31
GAS09344	New Zealand	Oceania	2009	ARF	emm106.0	1012	14	152	21	25	106	19	34	36	15	3	124	34
GAS13376	New Zealand	Oceania	2013	invasive, NOS	emm49.0	433	4	8	4	11	14	14	16	18	9	2	12	13
GAS0945	New Zealand	Oceania	2009	pharyngitis and/or tonsillitis	emm6.0	382	65	80	62	55	85	62	97	130	11	20	69	82
GAS06308	New Zealand	Oceania	2006	ARF	emm54.1	990	11	5	10	20	15	16	17	13	1	2	16	31
GAS05151	New Zealand	Oceania	2005	ARF	emm89.0	142	4	8	3	11	14	14	16	18	9	2	12	13
GAS0370	New Zealand	Oceania	2003	invasive, NOS	emm82.0	334	73	58	46	1	6	18	69	77	1	1	1	3
GAS10346	New Zealand	Oceania	2010	invasive, NOS	emm71.0	318	19	20	13	19	2	22	3	27	1	1	4	24
GAS10179	New Zealand	Oceania	2010	ARF	emm91.0	12	11	5	10	15	15	16	17	13	1	2	16	18
GAS1041	New Zealand	Oceania	2010	invasive, NOS	emm91.0	12	11	5	10	15	15	16	17	13	1	2	16	18
GAS09330	New Zealand	Oceania	2009	ARF	emm49.0	433	4	8	4	11	14	14	16	18	9	2	12	13
GAS0944	New Zealand	Oceania	2009	ARF	ND	382	65	80	62	55	85	62	97	130	11	20	69	82
GAS06262	New Zealand	Oceania	2006	invasive, NOS	emm54.1	990	11	5	10	20	15	16	17	13	1	2	16	31
GAS1376	New Zealand	Oceania	2013	pharyngitis and/or tonsillitis	emm70.0	1003	16	16	3	16	17	7	193	22	1	2	18	3
GAS05150	New Zealand	Oceania	2005	ARF	emm53.0	11	49	5	10	8	15	54	17	84	1	2	6	20
GAS0361	New Zealand	Oceania	2003	ARF	emm53.0	11	49	5	10	8	15	54	17	84	1	2	6	20
GAS10339	New Zealand	Oceania	2010	invasive, NOS	emm53.0	11	121	5	10	8	15	54	17	84	1	2	6	20
GAS10157	New Zealand	Oceania	2010	invasive, NOS	emm232.0	902	14	37	31	3	2	38	47	242	1	2	58	25
GAS1023	New Zealand	Oceania	2010	invasive, NOS	emm113.0	148	14	3	3	3	2	3	52	59	24	12	38	3
GAS09267	New Zealand	Oceania	2009	ARF	emm92.0	82	17	4	3	18	19	19	22	24	13	2	20	22
GAS06220	New Zealand	Oceania	2006	invasive, NOS	emm1.0	28	1	1	1	1	1	1	1	1	1	1	1	1
GAS05134	New Zealand	Oceania	2005	ARF	emm1.0	28	1	1	1	1	1	1	1	1	1	1	1	1
GAS0359	New Zealand	Oceania	2003	ARF	emm82.0	334	73	58	46	1	6	18	69	77	1	1	1	3
GAS12332	New Zealand	Oceania	2012	pharyngitis and/or tonsillitis	emm33.0	3	11	11	7	10	35	36	85	79	1	2	6	20
GAS10325	New Zealand	Oceania	2010	ARF	emm105.0	151	6	6	6	6	5	6	6	12	5	4	6	11
GAS10144	New Zealand	Oceania	2010	invasive, NOS	emm6.0	411	65	80	62	55	85	62	97	130	11	20	69	82
GAS1019	New Zealand	Oceania	2010	ARF	emm22.0	202	33	11	7	10	35	36	44	49	19	2	30	43
GAS09257	New Zealand	Oceania	2009	ARF	emm39.4	268	1	60	49	45	61	19	13	83	34	3	61	29
GAS0925	New Zealand	Oceania	2009	ARF	emm74.0	120	28	13	3	41	50	53	123	82	11	4	59	70

GAS06216	New Zealand	Oceania	2006	pharyngitis and/or tonsillitis	emm1.0	28	1	1	1	1	1	1	1	1	1	1	1	1
GAS0592	New Zealand	Oceania	2005	invasive, NOS	emm44.0	178	5	7	7	3	2	3	24	8	14	3	7	7
GAS0354	New Zealand	Oceania	2003	invasive, NOS	emm101.0	182	15	31	10	20	15	31	38	41	1	3	32	37
GAS10275	New Zealand	Oceania	2010	pharyngitis and/or tonsillitis	emm74.0	120	28	13	3	41	50	53	123	82	11	4	59	70
GAS10141	New Zealand	Oceania	2010	ARF	emm232.0	967	14	37	31	3	2	38	47	242	1	2	58	25
GAS12183	New Zealand	Oceania	2012	ARF	emm89.14	380	14	49	20	16	112	52	136	248	33	11	57	25
GAS09441	New Zealand	Oceania	2009	pharyngitis and/or tonsillitis	emm113.0	148	14	3	3	3	2	3	52	59	24	12	38	3
GAS09227	New Zealand	Oceania	2009	invasive, NOS	emm92.0	82	17	4	3	18	19	19	22	24	13	2	20	22
GAS0919	New Zealand	Oceania	2009	invasive, NOS	ND	382	65	80	62	55	85	62	97	130	11	20	69	82
GAS06166	New Zealand	Oceania	2006	pharyngitis and/or tonsillitis	emm12.0	36	14	3	3	3	2	3	52	59	24	12	38	3
GAS03202	New Zealand	Oceania	2003	pharyngitis and/or tonsillitis	emm53.0	11	49	5	10	8	15	54	17	84	1	2	6	20
GAS0327	New Zealand	Oceania	2003	invasive, NOS	emm3.1	15	15	15	10	1	10	32	111	129	1	2	6	31
GAS10262	New Zealand	Oceania	2010	invasive, NOS	emm22.0	992	11	11	7	10	35	95	198	49	28	1	30	43
GAS10113	New Zealand	Oceania	2010	pharyngitis and/or tonsillitis	emm74.0	120	28	13	75	41	50	53	123	82	11	4	59	70
GAS09438	New Zealand	Oceania	2009	invasive, NOS	emm36.2	748	15	15	10	1	10	32	197	21	12	8	37	31
GAS09226	New Zealand	Oceania	2009	ARF	emm59.1	172	71	84	24	2	93	68	107	122	18	3	75	85
GAS1291	New Zealand	Oceania	2012	ARF	emm77.0	572	15	99	3	39	56	13	68	146	32	3	54	157
GAS0917	New Zealand	Oceania	2009	pharyngitis and/or tonsillitis	emm6.0	382	65	80	62	55	85	62	97	130	11	20	69	82
GAS0656	New Zealand	Oceania	2006	invasive, NOS	emm65.0	111	22	22	17	38	43	43	28	65	1	1	1	27
GAS03188	New Zealand	Oceania	2003	pharyngitis and/or tonsillitis	emm89.0	142	4	8	3	11	14	14	16	18	9	2	12	13
GAS0320	New Zealand	Oceania	2003	ARF	emm3.1	15	15	15	10	1	10	32	111	129	1	2	6	31
GAS10250	New Zealand	Oceania	2010	ARF	emm87.0	541	55	68	10	1	72	58	87	95	1	2	52	74
GAS10112	New Zealand	Oceania	2010	ARF	emm71.0	318	19	20	13	19	2	22	3	27	1	1	4	24
GAS09437	New Zealand	Oceania	2009	ARF	emm12.0	36	14	3	3	3	2	3	52	59	24	12	38	3
GAS09219	New Zealand	Oceania	2009	invasive, NOS	emm59.1	172	71	84	24	2	93	68	107	122	18	3	75	85
GAS08308	New Zealand	Oceania	2008	pharyngitis and/or tonsillitis	emm6.0	382	65	80	62	55	85	62	97	130	11	20	69	82
GAS0617	New Zealand	Oceania	2006	invasive, NOS	emm12.0	36	14	3	3	3	62	3	52	59	24	12	38	3
GAS11453	New Zealand	Oceania	2011	invasive, NOS	emm36.2	748	15	15	10	1	10	32	197	21	12	8	37	31
GAS0317	New Zealand	Oceania	2003	pharyngitis and/or tonsillitis	emm3.1	15	15	15	10	1	10	32	111	129	1	2	6	31
GAS10245	New Zealand	Oceania	2010	invasive, NOS	emm90.5	184	20	20	14	19	2	23	25	28	15	3	22	25
GAS10104	New Zealand	Oceania	2010	pharyngitis and/or tonsillitis	emm90.5	184	20	20	14	19	2	23	25	28	15	3	22	25
GAS09426	New Zealand	Oceania	2009	ARF	emm101.0	182	15	31	10	20	15	31	38	41	1	3	32	37
GAS09205	New Zealand	Oceania	2009	ARF	emm108.1	14	28	87	10	1	6	63	78	240	1	2	6	31
GAS08119	New Zealand	Oceania	2008	pharyngitis and/or tonsillitis	emm53.0	11	49	5	10	8	15	54	17	84	1	2	6	20
GAS0615	New Zealand	Oceania	2006	pharyngitis and/or tonsillitis	emm53.0	11	121	5	10	8	15	54	17	84	1	2	6	20
GAS03109	New Zealand	Oceania	2003	ARF	emm81.3	995	8	4	4	4	3	13	4	42	21	1	4	4
GAS02198	New Zealand	Oceania	2002	ARF	emm78.3	1000	11	32	52	1	67	13	199	249	23	2	63	73
GAS11262	New Zealand	Oceania	2011	ARF	emm74.0	120	28	13	3	41	50	53	123	82	11	4	59	70
GAS1161	New Zealand	Oceania	2011	invasive, NOS	emm71.0	318	19	20	13	19	2	22	3	27	1	1	4	24
GAS11290	New Zealand	Oceania	2011	ARF	emm19.4	616	50	32	10	1	6	55	78	13	1	2	6	106
GAS13362	New Zealand	Oceania	2013	invasive, NOS	emm19.4	616	50	32	10	1	6	55	78	13	1	2	6	106
GAS1341	New Zealand	Oceania	2013	invasive, NOS	emm197.0	998	11	151	10	8	165	16	17	236	1	2	6	20
GAS12316	New Zealand	Oceania	2012	ARF	emm74.0	120	28	13	3	41	50	53	123	82	11	4	59	70
GAS12180	New Zealand	Oceania	2012	invasive, NOS	emm81.0	330	62	75	45	52	5	6	45	106	24	17	66	61
GAS1282	New Zealand	Oceania	2012	ARF	emm81.0	330	62	75	45	52	5	6	45	106	24	17	66	61
GAS11441	New Zealand	Oceania	2011	ARF	emm19.4	616	50	32	10	1	6	55	78	13	1	2	6	106
GAS11237	New Zealand	Oceania	2011	invasive, NOS	emm44.0	178	5	7	7	3	2	3	24	8	14	3	7	7
GAS1156	New Zealand	Oceania	2011	pharyngitis and/or tonsillitis	emm65.0	111	22	22	17	38	23	43	28	65	1	1	1	27

GAS13358	New Zealand	Oceania	2013	invasive, NOS	emm197.0	998	11	151	10	8	165	16	17	236	1	2	6	20
GAS1328	New Zealand	Oceania	2013	invasive, NOS	emm74.0	120	28	13	3	41	50	53	123	82	11	4	59	70
GAS1187	New Zealand	Oceania	2011	ARF	emm71.0	318	19	20	13	19	2	22	3	27	1	1	4	24
GAS12314	New Zealand	Oceania	2012	pharyngitis and/or tonsillitis	emm53.0	11	49	5	10	8	15	54	17	84	1	2	6	20
GAS12172	New Zealand	Oceania	2012	pharyngitis and/or tonsillitis	emm3.1	15	15	15	10	1	10	32	111	129	1	2	6	31
GAS1262	New Zealand	Oceania	2012	invasive, NOS	emm104.0	789	14	13	10	8	13	13	15	17	10	2	15	16
GAS11426	New Zealand	Oceania	2011	pharyngitis and/or tonsillitis	emm53.0	11	49	5	10	8	15	54	17	84	1	2	6	20
GAS11212	New Zealand	Oceania	2011	invasive, NOS	emm74.0	120	28	13	3	41	50	53	123	82	11	4	59	70
GAS1142	New Zealand	Oceania	2011	ARF	emm65.4	129	11	66	40	35	102	76	44	145	1	2	87	31
GAS13350	New Zealand	Oceania	2013	pharyngitis and/or tonsillitis	emm19.4	616	50	32	10	1	6	55	78	13	1	2	6	106
GAS1318	New Zealand	Oceania	2013	invasive, NOS	emm232.0	902	14	37	31	3	2	38	47	242	1	2	58	25
GAS12274	New Zealand	Oceania	2012	pharyngitis and/or tonsillitis	emm218.1	292	38	45	3	16	44	44	53	61	54	2	12	3
GAS12171	New Zealand	Oceania	2012	ARF	emm3.33	15	15	15	10	1	10	32	111	129	1	2	6	31
GAS13399	New Zealand	Oceania	2013	ARF	emm100.5	119	11	11	7	10	10	7	37	39	19	31	30	31
GAS1224	New Zealand	Oceania	2012	ARF	emm238.2	868	1	20	123	1	1	15	14	16	5	6	14	15
GAS11423	New Zealand	Oceania	2011	ARF	emm53.0	11	49	5	10	8	15	54	17	84	1	2	6	20
GAS11195	New Zealand	Oceania	2011	invasive, NOS	emm59.1	172	71	84	24	2	93	68	107	122	18	3	75	85
GAS1121	New Zealand	Oceania	2011	ARF	emm41.2	579	11	5	10	51	15	16	77	99	1	2	6	20
GAS13257	New Zealand	Oceania	2013	pharyngitis and/or tonsillitis	emm95.0	604	11	32	30	31	37	7	46	51	1	2	6	44
GAS1315	New Zealand	Oceania	2013	invasive, NOS	emm76.0	378	122	3	3	3	2	3	52	59	24	12	38	3
GAS12271	New Zealand	Oceania	2012	invasive, NOS	emm1.0	28	1	1	1	1	1	1	1	1	1	1	1	1
GAS12163	New Zealand	Oceania	2012	ARF	emm108.1	14	28	87	10	1	6	63	78	240	1	2	6	31
GAS1222	New Zealand	Oceania	2012	pharyngitis and/or tonsillitis	emm71.0	981	19	20	13	19	2	22	3	27	1	1	4	24
GAS11371	New Zealand	Oceania	2011	pharyngitis and/or tonsillitis	emm11.0	ND	1	6	76	1	104	20	13	25	8	3	21	23
GAS1377	New Zealand	Oceania	2013	pharyngitis and/or tonsillitis	emm59.1	172	71	84	24	2	93	68	107	122	18	3	75	85
GAS11186	New Zealand	Oceania	2011	pharyngitis and/or tonsillitis	emm19.4	616	50	32	10	1	6	55	78	13	1	2	6	106
GAS10495	New Zealand	Oceania	2010	pharyngitis and/or tonsillitis	emm19.4	616	50	32	10	1	6	55	78	13	1	2	6	106
GAS13208	New Zealand	Oceania	2013	pharyngitis and/or tonsillitis	emm74.0	120	28	13	3	41	50	53	123	82	11	4	59	70
GAS12484	New Zealand	Oceania	2012	invasive, NOS	emm77.0	572	15	99	3	39	56	13	68	146	32	3	54	101
GAS12260	New Zealand	Oceania	2012	ARF	emm104.0	789	14	13	10	8	13	13	15	17	10	2	15	16
GAS12150	New Zealand	Oceania	2012	ARF	emm232.0	902	14	37	31	3	2	38	47	242	1	2	58	25
GAS1219	New Zealand	Oceania	2012	invasive, NOS	emm108.1	14	28	87	10	1	6	63	78	240	1	2	6	31
GAS11364	New Zealand	Oceania	2011	ARF	emm74.0	120	28	13	3	41	50	53	123	82	11	4	59	70
GAS11154	New Zealand	Oceania	2011	pharyngitis and/or tonsillitis	emm41.2	579	11	5	10	51	15	16	77	99	1	2	6	20
GAS10481	New Zealand	Oceania	2010	ARF	emm74.0	120	28	13	3	41	50	53	123	82	11	4	59	70
GAS12357	New Zealand	Oceania	2012	ARF	emm41.2	579	11	5	10	51	15	16	77	99	1	2	6	20
GAS13169	New Zealand	Oceania	2013	pharyngitis and/or tonsillitis	emm232.0	902	14	37	31	3	2	38	47	242	1	2	58	25
GAS12463	New Zealand	Oceania	2012	pharyngitis and/or tonsillitis	emm41.2	579	11	5	10	51	15	16	77	99	1	2	6	20
GAS12244	New Zealand	Oceania	2012	pharyngitis and/or tonsillitis	emm71.0	318	19	20	13	19	2	22	3	27	1	1	4	24
GAS12144	New Zealand	Oceania	2012	invasive, NOS	emm108.1	14	28	87	10	1	6	63	78	240	1	2	6	31
GAS1218	New Zealand	Oceania	2012	invasive, NOS	emm3.1	15	15	15	10	1	10	32	111	129	1	2	6	31
GAS11353	New Zealand	Oceania	2011	ARF	emm92.0	82	17	4	3	18	19	19	22	24	13	2	20	22
GAS11146	New Zealand	Oceania	2011	ARF	emm44.0	178	5	7	7	3	2	3	24	8	14	3	7	7
GAS10476	New Zealand	Oceania	2010	invasive, NOS	emm33.0	3	11	11	7	10	35	36	85	79	1	2	6	20
GAS13137	New Zealand	Oceania	2013	pharyngitis and/or tonsillitis	emm98.1	205	15	15	10	1	10	96	19	21	12	2	6	20
GAS12446	New Zealand	Oceania	2012	pharyngitis and/or tonsillitis	emm74.0	120	28	13	3	41	50	53	123	82	11	4	59	70
GAS12229	New Zealand	Oceania	2012	ARF	emm238.2	1074	1	1	1	1	1	15	14	93	5	6	14	15
GAS12252	New Zealand	Oceania	2012	invasive, NOS	emm1.0	28	1	1	1	1	1	1	1	3	1	1	1	1

GAS12140	New Zealand	Oceania	2012	invasive, NOS	emm232.0	902	14	37	31	3	2	38	47	242	1	2	58	25
GAS1213	New Zealand	Oceania	2012	invasive, NOS	emm232.0	902	14	37	31	3	2	38	47	242	1	2	58	25
GAS11340	New Zealand	Oceania	2011	invasive, NOS	emm19.4	616	50	32	10	1	6	55	78	13	1	2	6	106
GAS11141	New Zealand	Oceania	2011	ARF	emm59.1	172	71	84	24	2	93	68	107	122	18	3	75	85
GAS10470	New Zealand	Oceania	2010	invasive, NOS	emm74.0	120	28	13	3	41	50	53	123	82	11	4	59	70
GAS13132	New Zealand	Oceania	2013	pharyngitis and/or tonsillitis	emm108.1	14	28	87	10	1	6	63	78	240	1	2	6	31
GAS12411	New Zealand	Oceania	2012	invasive, NOS	emm218.1	292	38	45	3	16	44	93	53	61	54	2	12	3
GAS12243	New Zealand	Oceania	2012	pharyngitis and/or tonsillitis	emm1.0	28	1	1	1	1	1	1	1	3	1	1	1	1
GAS12139	New Zealand	Oceania	2012	invasive, NOS	emm104.0	789	14	13	10	8	13	13	15	17	10	2	15	16
GAS12106	New Zealand	Oceania	2012	pharyngitis and/or tonsillitis	emm81.0	330	62	75	45	52	5	6	45	106	24	17	66	61
GAS11551	New Zealand	Oceania	2011	pharyngitis and/or tonsillitis	emm71.0	318	19	20	13	19	2	22	3	27	1	1	4	24
GAS11334	New Zealand	Oceania	2011	invasive, NOS	emm92.0	82	17	4	3	82	19	19	22	24	13	2	20	22
GAS11129	New Zealand	Oceania	2011	invasive, NOS	emm41.2	579	11	5	10	51	15	16	77	99	1	2	6	20
GAS10463	New Zealand	Oceania	2010	invasive, NOS	emm232.0	967	14	37	31	3	2	38	47	242	1	2	58	25
GAS10458	New Zealand	Oceania	2010	invasive, NOS	emm36.2	748	15	15	10	1	10	32	197	21	12	8	37	31
GAS10243	New Zealand	Oceania	2010	invasive, NOS	emm105.0	954	6	6	6	6	5	6	6	12	5	4	6	11
GAS1084	New Zealand	Oceania	2010	ARF	emm232.0	902	14	37	31	3	2	38	47	242	1	2	58	25
GAS09422	New Zealand	Oceania	2009	invasive, NOS	emm12.0	36	14	3	3	3	2	3	52	243	24	12	38	156
GAS09204	New Zealand	Oceania	2009	ARF	emm93.4	814	28	104	10	1	108	32	61	42	50	2	93	69
GAS0830	New Zealand	Oceania	2008	ARF	emm33.0	3	11	11	7	10	35	36	85	79	1	2	6	20
GAS11456	New Zealand	Oceania	2011	invasive, NOS	emm36.2	748	15	15	10	1	10	32	197	21	12	8	37	31
GAS066	New Zealand	Oceania	2006	ARF	emm65.0	111	22	22	17	38	43	43	28	65	1	1	1	27
GAS03101	New Zealand	Oceania	2003	ARF	emm101.0	182	15	31	10	20	15	31	38	41	1	3	32	37
GAS10417	New Zealand	Oceania	2010	invasive, NOS	emm232.0	902	14	37	124	3	2	38	47	242	1	2	58	25
GAS10219	New Zealand	Oceania	2010	invasive, NOS	emm232.0	902	14	37	31	3	2	38	47	242	1	2	58	25
GAS1074	New Zealand	Oceania	2010	invasive, NOS	emm71.0	318	19	20	13	19	2	22	3	27	1	1	4	24
GAS09418	New Zealand	Oceania	2009	pharyngitis and/or tonsillitis	emm105.0	954	6	6	6	6	5	6	6	12	5	4	6	11
GAS09194	New Zealand	Oceania	2009	invasive, NOS	emm65.0	111	22	22	17	38	23	43	28	65	1	1	1	27
GAS07174	New Zealand	Oceania	2007	ARF	emm232.1	1013	18	18	16	16	21	25	125	250	8	3	21	23
GAS05207	New Zealand	Oceania	2005	pharyngitis and/or tonsillitis	emm93.4	814	28	104	10	1	108	32	61	42	50	2	93	69
GAS0391	New Zealand	Oceania	2003	invasive, NOS	emm78.3	1000	11	32	52	1	67	13	199	249	23	2	63	73
NZ131	New Zealand	Oceania	1991	APSGN	emm49.1	1073	4	153	4	11	168	97	200	251	9	2	99	158
GUR	Russia	Asia	2017	ND	emm111.2	1065	14	154	75	8	169	19	187	109	1	2	125	159
GURSA1	Russia	Asia	2017	ND	emm111.2	1065	14	154	75	8	169	19	187	109	1	2	125	159
A20	Taiwan	Asia	ND	invasive, NOS	emm1.0	28	1	1	1	1	1	1	1	1	1	1	1	160
MGAS2096	Trinidad and Tobago	North America	1960	APSGN	emm12.0	36	14	3	3	3	2	3	52	59	24	12	38	3
PHE_12088	UK	Europe	2014	scarlet fever	emm3.56	15	15	15	10	1	10	32	111	129	1	2	6	31
PHE_12036	UK	Europe	2014	invasive, NOS	emm131.0	785	1	1	1	1	1	1	1	1	1	1	1	1
PHE_12783	UK	Europe	2014	invasive, NOS	emm90.5	184	20	20	14	83	170	23	25	28	15	3	22	25
PHE_33063	UK	Europe	2014	scarlet fever	emm12.0	1068	14	3	3	3	2	3	52	59	24	12	38	3
PHE_32280	UK	Europe	2014	invasive, NOS	emm12.37	242	14	3	3	3	2	3	52	252	24	12	38	3
PHE_24974	UK	Europe	2014	invasive, NOS	emm73.0	331	14	96	3	41	50	13	3	137	48	2	49	60
PHE_55087	UK	Europe	2014	scarlet fever	emm2.0	55	32	26	28	24	29	13	43	48	15	3	12	42
PHE_33054	UK	Europe	2014	invasive, NOS	emm53.0	363	46	32	7	1	171	7	201	79	1	16	56	31
PHE_107859	UK	Europe	2014	scarlet fever	emm22.0	46	7	149	5	5	4	5	5	253	4	3	5	5
PHE_12749	UK	Europe	2014	scarlet fever	emm12.0	36	14	3	3	3	2	3	52	59	24	12	38	3
PHE_39584	UK	Europe	2014	scarlet fever	emm58.0	176	14	94	10	8	13	13	15	47	10	2	34	31
PHE_27636	UK	Europe	2014	scarlet fever	emm77.0	399	82	25	3	22	27	13	26	29	8	3	25	30

PHE_39586	UK	Europe	2014	scarlet fever	emm28.0	458	3	3	3	3	2	3	202	4	1	1	3	3
PHE_18921	UK	Europe	2014	scarlet fever	emm131.0	382	65	80	62	55	85	62	97	130	11	20	69	82
PHE_27701	UK	Europe	2014	scarlet fever	emm3.1	406	15	155	10	1	10	32	111	129	1	2	6	31
PHE_24945	UK	Europe	2014	invasive, NOS	emm5.23	99	14	91	70	1	17	71	114	254	47	22	80	91
PHE_12750	UK	Europe	2014	scarlet fever	emm12.0	36	14	3	3	3	2	3	52	59	24	12	38	3
PHE_12875	UK	Europe	2014	scarlet fever	emm6.0	382	65	80	62	55	85	62	97	130	11	20	69	82
PHE_25160	UK	Europe	2014	invasive, NOS	emm76.0	50	14	3	3	3	2	3	52	59	24	12	38	3
PHE_33034	UK	Europe	2014	scarlet fever	emm4.0	1066	124	8	4	7	7	9	8	9	7	2	8	8
PHE_12906	UK	Europe	2014	scarlet fever	emm75.0	150	25	26	20	24	29	29	33	155	15	3	12	33
PHE_26043	UK	Europe	2014	scarlet fever	emm28.0	52	3	3	3	3	2	3	3	4	1	1	126	161
PHE_26023	UK	Europe	2014	scarlet fever	emm6.0	382	65	80	62	55	85	62	97	130	11	20	69	82
PHE_12907	UK	Europe	2014	scarlet fever	emm3.93	315	15	15	10	1	10	32	111	129	1	2	6	31
PHE_30640	UK	Europe	2014	invasive, NOS	emm219.0	25	125	58	125	1	6	18	69	255	1	1	127	162
PHE_30600	UK	Europe	2014	invasive, NOS	emm6.0	382	65	80	62	55	85	62	97	130	11	20	69	82
PHE_33016	UK	Europe	2014	invasive, NOS	emm81.0	624	5	53	43	42	172	47	64	119	30	14	30	61
PHE_32292	UK	Europe	2014	invasive, NOS	emm90.2	184	14	88	68	57	94	70	109	127	1	2	79	88
PHE_12026	UK	Europe	2014	invasive, NOS	emm3.56	15	15	15	10	1	10	32	111	129	1	2	6	31
PHE_12861	UK	Europe	2014	scarlet fever	emm3.1	315	15	15	10	1	10	32	111	129	1	2	6	31
PHE_18945	UK	Europe	2014	invasive, NOS	emm81.0	624	5	53	43	42	52	47	64	119	30	14	30	61
PHE_25152	UK	Europe	2014	scarlet fever	emm219.0	25	14	58	125	1	6	18	203	255	1	1	127	162
PHE_45816	UK	Europe	2014	scarlet fever	emm28.0	52	3	3	3	3	2	3	3	4	1	1	3	3
PHE_12832	UK	Europe	2014	scarlet fever	emm4.0	39	4	8	4	7	7	9	8	9	7	2	8	8
PHE_12841	UK	Europe	2014	scarlet fever	emm87.0	62	67	15	10	1	72	58	87	95	1	2	52	74
PHE_12764	UK	Europe	2014	scarlet fever	emm28.0	52	3	3	3	84	2	3	204	4	1	1	3	163
PHE_12067	UK	Europe	2014	invasive, NOS	emm81.0	624	5	53	43	42	52	47	64	119	30	14	30	61
PHE_45811	UK	Europe	2014	scarlet fever	emm75.0	150	25	26	20	24	29	29	33	155	15	3	12	33
PHE_12021	UK	Europe	2014	scarlet fever	emm12.60	36	14	3	3	3	2	3	52	59	24	12	38	3
PHE_18939	UK	Europe	2014	invasive, NOS	emm94.0	89	8	4	4	4	3	13	4	42	21	1	4	4
PHE_25158	UK	Europe	2014	invasive, NOS	emm82.0	314	14	58	46	85	6	18	69	77	1	1	1	3
PHE_12046	UK	Europe	2014	scarlet fever	emm82.0	36	14	3	3	3	2	3	52	59	24	12	38	3
PHE_30630	UK	Europe	2014	invasive, NOS	emm1.25	28	1	1	1	1	1	1	1	134	1	1	1	1
PHE_24940	UK	Europe	2014	invasive, NOS	emm90.2	184	14	88	68	57	94	70	109	127	1	2	79	88
PHE_12912	UK	Europe	2014	scarlet fever	emm3.93	315	15	15	10	1	10	32	111	129	1	2	6	31
PHE_12855	UK	Europe	2014	scarlet fever	emm89.0	101	2	2	2	2	2	2	2	2	2	2	2	2
PHE_45825	UK	Europe	2014	scarlet fever	emm12.37	242	14	3	3	3	2	3	52	252	24	12	38	3
PHE_104230	UK	Europe	2014	invasive, NOS	emm44.0	1086	125	58	125	1	6	18	69	255	1	1	127	162
PHE_32243	UK	Europe	2014	invasive, NOS	emm94.0	89	8	4	4	4	3	13	4	42	21	1	4	4
PHE_30599	UK	Europe	2014	invasive, NOS	emm58.8	985	20	20	14	19	2	23	25	57	18	3	22	25
PHE_12810	UK	Europe	2014	scarlet fever	emm6.0	382	65	80	62	55	85	62	205	130	11	20	69	82
PHE_26033	UK	Europe	2014	scarlet fever	emm4.0	38	4	8	4	11	51	9	8	9	7	2	8	8
PHE_45819	UK	Europe	2014	scarlet fever	emm4.0	39	4	8	4	7	51	9	8	9	7	2	8	8
PHE_39598	UK	Europe	2014	scarlet fever	emm6.0	382	65	80	126	55	85	62	97	130	11	20	69	82
PHE_30647	UK	Europe	2014	scarlet fever	emm4.0	38	4	8	4	11	173	9	8	9	7	2	8	8
PHE_45826	UK	Europe	2014	scarlet fever	emm3.103	406	15	15	10	1	10	32	111	129	1	2	6	31
PHE_26068	UK	Europe	2014	invasive, NOS	emm22.0	46	7	5	5	5	4	5	5	6	4	3	5	5
PHE_12780	UK	Europe	2014	invasive, NOS	emm90.2	184	11	32	52	1	67	13	118	256	1	2	83	93
PHE_30624	UK	Europe	2014	invasive, NOS	emm58.8	985	20	20	14	19	2	23	25	57	18	3	22	25
PHE_104235	UK	Europe	2014	invasive, NOS	emm73.0	331	14	96	3	41	50	13	3	137	48	2	49	60

PHE_26041	UK	Europe	2014	invasive, NOS	emm3.5	15	15	15	10	1	10	32	111	129	1	2	6	31
PHE_12853	UK	Europe	2014	scarlet fever	emm4.0	39	4	8	4	7	7	9	8	9	7	2	8	8
PHE_45820	UK	Europe	2014	scarlet fever	emm87.0	62	67	15	10	1	72	58	87	95	1	2	52	74
PHE_12908	UK	Europe	2014	scarlet fever	emm3.1	315	15	15	10	1	10	32	111	129	1	2	6	31
PHE_18896	UK	Europe	2014	scarlet fever	emm131.0	382	65	80	62	55	85	62	97	130	11	20	69	82
PHE_12833	UK	Europe	2014	invasive, NOS	emm3.56	15	15	15	10	1	10	32	111	129	1	2	6	31
PHE_12076	UK	Europe	2014	invasive, NOS	emm94.0	89	8	4	4	4	3	13	4	42	21	1	4	4
PHE_18919	UK	Europe	2014	invasive, NOS	emm103.0	327	14	156	3	1	66	13	82	87	1	2	49	4
PHE_55078	UK	Europe	2014	scarlet fever	emm75.0	150	25	26	20	24	29	29	33	155	15	3	12	33
PHE_104231	UK	Europe	2014	invasive, NOS	emm18.0	42	75	92	61	5	98	72	115	132	47	2	69	5
PHE_12894	UK	Europe	2014	scarlet fever	emm3.1	315	15	15	10	1	10	32	111	129	1	2	6	31
PHE_33069	UK	Europe	2014	scarlet fever	emm12.0	1068	14	3	3	3	2	3	52	59	24	12	38	3
PHE_33033	UK	Europe	2014	invasive, NOS	emm1.0	785	1	1	1	1	1	1	1	1	1	1	1	1
PHE_12792	UK	Europe	2014	scarlet fever	emm3.1	15	15	15	10	1	10	32	111	129	1	2	6	31
PHE_104228	UK	Europe	2014	scarlet fever	emm2.0	55	32	26	28	24	29	13	43	257	15	3	12	42
PHE_12903	UK	Europe	2014	scarlet fever	emm131.0	382	65	80	62	55	85	62	97	130	11	20	69	82
PHE_25109	UK	Europe	2014	scarlet fever	emm12.0	36	14	3	3	3	2	3	52	59	24	12	38	3
PHE_12757	UK	Europe	2014	invasive, NOS	emm81.0	624	5	53	43	42	52	47	64	119	30	14	30	61
PHE_24950	UK	Europe	2014	invasive, NOS	emm76.0	50	14	3	3	3	2	3	52	59	24	12	38	3
PHE_39585	UK	Europe	2014	scarlet fever	emm3.1	406	15	155	10	1	10	32	111	129	1	2	6	31
PHE_12854	UK	Europe	2014	scarlet fever	emm89.0	101	2	2	2	2	2	2	2	2	55	2	2	2
PHE_12066	UK	Europe	2014	invasive, NOS	emm58.0	176	14	94	10	8	13	13	15	47	10	2	34	31
PHE_39599	UK	Europe	2014	scarlet fever	emm6.0	382	65	80	62	55	85	62	97	130	11	20	69	82
PHE_12062	UK	Europe	2014	scarlet fever	emm3.1	406	15	155	10	1	10	32	111	129	1	2	6	31
PHE_104218	UK	Europe	2014	scarlet fever	emm89.0	101	2	2	2	2	2	2	2	2	2	2	2	2
PHE_45817	UK	Europe	2014	scarlet fever	emm3.93	315	15	15	10	1	10	32	111	129	1	2	6	31
PHE_18915	UK	Europe	2014	scarlet fever	emm2.0	55	32	26	28	24	29	13	43	48	15	3	12	42
PHE_32271	UK	Europe	2014	invasive, NOS	emm22.0	46	7	149	5	5	4	5	5	6	4	3	5	5
PHE_12031	UK	Europe	2014	invasive, NOS	emm5.3	99	14	91	70	1	17	71	114	131	47	22	80	91
PHE_18938	UK	Europe	2014	invasive, NOS	emm171.1	1062	88	157	3	16	44	85	151	177	54	2	128	164
PHE_30646	UK	Europe	2014	scarlet fever	emm28.0	1067	3	3	3	3	2	3	3	4	1	1	3	3
PHE_39608	UK	Europe	2014	scarlet fever	emm9.0	75	34	77	60	32	2	1	95	113	5	3	11	45
PHE_18958	UK	Europe	2014	scarlet fever	emm3.23	15	15	15	10	1	10	32	111	129	1	2	6	31
PHE_26066	UK	Europe	2014	invasive, NOS	emm4.0	38	4	8	4	11	51	9	8	9	61	2	8	8
PHE_12888	UK	Europe	2014	scarlet fever	emm3.5	15	127	15	10	1	10	32	111	129	1	2	6	31
PHE_12890	UK	Europe	2014	scarlet fever	emm3.1	15	15	15	10	1	10	32	111	129	1	2	6	31
PHE_27648	UK	Europe	2014	invasive, NOS	emm9.0	75	34	77	60	32	2	1	95	113	5	3	11	45
PHE_26062	UK	Europe	2014	invasive, NOS	emm58.0	176	14	94	10	8	13	13	15	47	10	2	34	31
PHE_33053	UK	Europe	2014	invasive, NOS	emm9.0	75	34	77	60	32	2	1	95	113	5	3	11	45
PHE_37685	UK	Europe	2014	invasive, NOS	emm82.0	36	14	3	3	3	2	3	52	59	24	12	38	3
PHE_12826	UK	Europe	2014	scarlet fever	emm3.1	15	15	15	10	1	10	32	111	129	1	2	6	31
PHE_12806	UK	Europe	2014	scarlet fever	emm1.0	28	1	1	1	1	1	1	1	3	78	1	1	1
PHE_32261	UK	Europe	2014	invasive, NOS	emm3.122	406	15	15	10	1	10	32	111	129	1	2	6	31
PHE_55095	UK	Europe	2014	scarlet fever	emm6.0	382	65	80	62	55	85	62	97	130	11	20	69	82
PHE_30587	UK	Europe	2014	scarlet fever	emm3.111	315	15	15	10	1	10	32	111	129	1	2	6	31
PHE_33064	UK	Europe	2014	scarlet fever	emm12.0	1068	14	3	3	3	2	3	52	59	24	12	38	3
PHE_12805	UK	Europe	2014	scarlet fever	emm3.106	315	15	15	10	1	10	32	111	129	1	2	6	31
PHE_12804	UK	Europe	2014	invasive, NOS	emm77.0	63	23	24	57	1	26	28	30	33	8	3	24	29

PHE_27706	UK	Europe	2014	invasive, NOS	emm77.0	63	23	24	57	1	26	28	30	33	8	3	24	29
PHE_12864	UK	Europe	2014	scarlet fever	emm87.0	62	67	15	10	1	72	58	87	95	1	2	52	74
PHE_25153	UK	Europe	2014	scarlet fever	emm28.0	1079	3	3	3	3	2	3	3	4	1	1	3	3
GASEMM1195	UK	Europe	2015	ND	emm11.0	ND	128	19	78	23	117	83	206	216	8	3	18	4
GASEMM1871	UK	Europe	2015	ND	emm100.2	773	93	25	3	26	71	45	162	190	60	26	114	135
GASEMM2706	UK	Europe	2014	ND	emm92.0	674	114	158	34	27	39	13	22	152	13	2	20	22
GASEMM2087	UK	Europe	2015	ND	emm18.38	42	75	92	61	5	98	72	115	132	47	2	69	165
GASEMM2594	UK	Europe	2015	ND	emm66.0	44	14	82	64	31	6	18	127	77	1	1	73	3
GASEMM0115	UK	Europe	2015	ND	emm168.1	819	14	25	3	22	27	13	26	258	8	3	83	28
GASEMM1220	UK	Europe	2015	ND	ND	984	28	29	7	1	32	30	36	38	1	32	129	50
GASEMM2296	UK	Europe	2015	ND	emm44.0	367	14	58	46	1	6	18	69	77	1	1	1	3
GASEMM2093	UK	Europe	2015	ND	emm12.0	242	14	3	3	3	2	3	52	252	24	12	38	3
GASEMM1829	UK	Europe	2015	ND	emm9.0	204	34	39	33	32	174	1	49	54	5	3	11	45
GASEMM1426	UK	Europe	2015	ND	emm74.0	120	28	13	3	41	50	53	123	82	11	4	59	70
GASEMM2806	UK	Europe	2015	ND	emm19.4	616	50	32	10	1	6	55	78	13	1	2	6	106
GASEMM2368	UK	Europe	2015	ND	emm5.120	99	14	91	70	1	17	71	114	131	47	22	80	91
GASEMM0105	UK	Europe	2015	ND	emm168.1	819	14	25	3	22	27	13	26	258	8	3	83	28
GASEMM0070	UK	Europe	2015	ND	emm49.0	371	4	8	4	11	14	14	16	18	9	2	12	13
GASEMM2885	UK	Europe	2015	ND	emm113.0	677	14	3	3	3	2	3	207	59	24	12	38	3
GASEMM0015	UK	Europe	2015	ND	emm75.0	150	25	26	20	24	29	29	33	155	79	3	12	33
GASEMM0145	UK	Europe	2015	ND	emm88.4	919	14	89	69	1	20	2	67	74	82	2	6	31
GASEMM2743	UK	Europe	2015	ND	emm92.0	674	114	158	34	27	39	13	22	152	13	2	20	22
GASEMM0052	UK	Europe	2015	ND	emm75.0	788	25	26	20	24	29	29	33	155	15	3	12	33
GASEMM0248	UK	Europe	2015	ND	emm28.0	52	3	3	3	84	2	3	3	4	1	1	3	3
GASEMM2187	UK	Europe	2015	ND	emm76.0	875	14	13	10	8	13	13	15	17	10	2	15	16
GASEMM1345	UK	Europe	2015	ND	emm89.0	966	2	2	2	2	2	2	2	2	2	2	2	2
GASEMM2863	UK	Europe	2015	ND	emm5.23	99	14	91	70	1	17	71	114	131	47	22	80	91
GASEMM2626	UK	Europe	2015	ND	emm76.0	875	14	13	10	8	13	13	15	17	10	2	15	16
GASEMM2825	UK	Europe	2015	ND	emm6.4	37	65	80	62	55	85	62	97	115	11	20	69	82
GASEMM0087	UK	Europe	2015	ND	emm81.0	117	40	53	43	42	52	47	64	70	30	14	30	61
GASEMM1403	UK	Europe	2015	ND	emm3.108	406	15	155	10	1	10	32	111	129	1	2	6	31
GASEMM1445	UK	Europe	2015	ND	emm12.19	36	14	3	3	3	2	3	52	59	24	12	38	3
GASEMM1730	UK	Europe	2015	ND	emm99.3	229	14	41	120	1	20	13	208	56	5	4	20	98
GASEMM0136	UK	Europe	2015	ND	emm1.53	28	1	1	1	1	1	1	1	259	1	1	1	1
GASEMM1699	UK	Europe	2015	ND	emm11.0	ND	1	6	40	61	20	20	13	139	8	3	18	4
GASEMM2100	UK	Europe	2015	ND	emm12.0	242	14	3	3	3	2	3	52	252	24	12	38	3
GASEMM1826	UK	Europe	2015	ND	emm76.0	378	14	3	3	3	2	3	52	59	24	12	38	3
GASEMM2311	UK	Europe	2015	ND	emm12.84	36	14	3	3	3	2	3	52	59	24	12	38	3
GASEMM1994	UK	Europe	2015	ND	emm32.4	386	129	159	7	1	89	64	209	260	1	1	70	3
GASEMM1462	UK	Europe	2015	ND	emm57.0	348	29	32	7	1	34	32	39	42	1	2	23	3
GASEMM1752	UK	Europe	2015	ND	emm18.21	402	11	13	3	41	50	13	112	42	31	1	51	4
GASEMM2438	UK	Europe	2015	ND	emm3.7	15	15	15	10	1	10	32	111	129	1	2	6	31
GASEMM1318	UK	Europe	2015	ND	emm11.24	ND	1	97	40	61	20	20	120	139	8	3	85	95
GASEMM1471	UK	Europe	2015	ND	emm3.88	15	127	15	10	1	10	32	111	129	1	2	6	31
GASEMM1536	UK	Europe	2015	ND	emm232.1	1013	18	18	16	16	21	25	125	26	8	3	21	23
GASEMM0963	UK	Europe	2015	ND	emm3.86	406	15	15	10	1	10	32	111	129	1	2	6	31
GASEMM0954	UK	Europe	2015	ND	emm3.94	15	15	15	10	1	10	32	111	129	1	2	6	31
GASEMM0568	UK	Europe	2015	ND	emm12.37	242	14	3	3	3	2	3	52	252	24	12	38	3

GASEMM2733	UK	Europe	2015	ND	emm5.119	99	14	91	70	1	17	71	114	131	47	22	80	91
GASEMM2842	UK	Europe	2015	ND	emm76.0	378	14	3	3	3	2	3	52	59	24	12	38	3
GASEMM1778	UK	Europe	2015	ND	emm131.0	382	65	80	62	55	85	62	97	261	11	20	69	82
GASEMM1780	UK	Europe	2015	ND	emm131.0	411	65	80	62	55	85	62	97	130	11	20	69	82
GASEMM0053	UK	Europe	2015	ND	emm75.0	788	25	26	20	24	29	29	33	155	15	3	12	33
GASEMM0090	UK	Europe	2015	ND	emm81.0	117	40	53	43	42	52	47	64	70	30	14	30	61
GASEMM2575	UK	Europe	2015	ND	emm218.1	224	38	45	3	86	44	44	53	61	54	2	12	3
GASEMM1282	UK	Europe	2015	ND	emm28.0	52	3	3	3	84	2	3	3	4	1	1	3	3
GASEMM2682	UK	Europe	2015	ND	emm3.41	15	15	15	10	1	10	32	111	129	1	2	6	31
GASEMM0832	UK	Europe	2015	ND	emm1.0	785	1	1	1	1	1	1	1	1	1	1	1	1
GASEMM1031	UK	Europe	2015	ND	emm218.1	292	38	45	3	16	44	44	53	61	8	3	18	4
GASEMM1603	UK	Europe	2015	ND	emm217.0	594	42	55	3	6	5	48	211	73	1	2	52	11
GASEMM2962	UK	Europe	2015	ND	emm49.4	228	14	25	79	22	27	13	26	29	8	3	25	114
GASEMM2653	UK	Europe	2015	ND	emm126.1	101	2	2	2	2	2	2	2	2	2	2	2	116
GASEMM1338	UK	Europe	2015	ND	emm123.0	123	11	13	3	41	50	99	4	42	3	1	51	4
GASEMM0065	UK	Europe	2015	ND	emm49.0	371	4	8	4	11	14	14	16	18	9	2	12	13
GASEMM2308	UK	Europe	2015	ND	emm169.3	53	70	4	3	87	19	19	101	118	42	2	72	3
GASEMM0141	UK	Europe	2015	ND	emm88.4	919	14	89	69	1	20	2	67	74	82	2	6	31
GASEMM2656	UK	Europe	2015	ND	emm80.0	701	16	16	3	16	116	7	20	22	1	2	18	110
GASEMM1755	UK	Europe	2015	ND	emm102.2	60	130	18	4	4	167	7	25	262	1	3	130	3
GASEMM2260	UK	Europe	2015	ND	emm12.84	36	14	3	3	3	2	3	52	59	24	12	38	3
GASEMM2454	UK	Europe	2015	ND	emm5.121	99	14	91	70	1	17	71	114	263	47	22	80	91
GASEMM1319	UK	Europe	2015	ND	emm11.12	ND	1	97	40	61	20	20	120	139	8	3	85	95
GASEMM0274	UK	Europe	2015	ND	emm12.37	242	14	3	3	3	2	3	52	252	24	12	38	3
GASEMM1880	UK	Europe	2015	ND	emm169.3	53	70	4	3	87	19	19	101	118	42	2	72	3
GASEMM1189	UK	Europe	2015	ND	emm3.116	15	15	15	10	1	10	32	111	129	1	2	6	31
GASEMM2898	UK	Europe	2015	ND	emm12.85	36	14	3	3	3	2	3	52	59	24	12	38	3
GASEMM2106	UK	Europe	2015	ND	emm3.19	15	15	15	10	1	10	32	111	129	1	2	6	31
GASEMM2778	UK	Europe	2015	ND	emm11.23	ND	1	97	40	61	20	20	120	139	8	3	85	95
GASEMM2115	UK	Europe	2015	ND	emm12.40	36	14	3	3	3	2	3	212	59	24	12	38	3
GASEMM1161	UK	Europe	2015	ND	emm78.3	253	2	160	7	88	176	43	213	264	2	34	2	166
GASEMM2204	UK	Europe	2015	ND	emm95.0	240	85	6	6	6	5	6	214	172	5	4	6	11
GASEMM2512	UK	Europe	2015	ND	emm5.16	99	14	91	70	1	17	71	114	254	47	22	80	91
GASEMM2268	UK	Europe	2015	ND	emm66.0	44	14	82	64	31	6	18	127	77	1	1	73	3
GASEMM2718	UK	Europe	2015	ND	emm6.49	382	65	80	62	55	85	62	97	130	11	20	69	82
GASEMM1801	UK	Europe	2015	ND	emm23.0	160	120	150	121	54	106	62	192	234	11	4	35	155
GASEMM0851	UK	Europe	2015	ND	emm6.0	839	65	80	62	55	85	62	97	130	11	20	69	82
GASEMM0022	UK	Europe	2015	ND	emm75.0	150	25	26	20	24	29	29	33	155	79	3	12	33
GASEMM2280	UK	Europe	2015	ND	emm109.1	718	94	126	3	27	177	37	45	191	15	3	35	34
GASEMM1932	UK	Europe	2015	ND	emm5.113	99	14	91	70	1	17	71	114	263	47	22	80	91
GASEMM0669	UK	Europe	2015	ND	emm32.2	386	129	159	7	1	89	64	209	260	1	1	70	3
GASEMM0619	UK	Europe	2015	ND	emm12.1	36	14	3	3	3	2	3	52	59	24	12	38	3
GASEMM1383	UK	Europe	2015	ND	emm5.86	99	14	91	70	1	17	71	114	131	47	22	80	91
GASEMM3004	UK	Europe	2015	ND	emm89.0	791	2	2	2	2	2	2	2	2	2	2	2	2
GASEMM1714	UK	Europe	2015	ND	emm165.0	277	131	161	113	89	106	100	215	265	77	2	131	25
GASEMM0726	UK	Europe	2015	ND	emm12.82	36	14	162	3	3	62	3	52	59	24	12	38	3
GASEMM1431	UK	Europe	2015	ND	emm58.7	176	14	13	10	8	13	13	195	47	10	2	34	31
GASEMM1947	UK	Europe	2015	ND	emm103.0	311	14	163	114	90	178	13	216	266	8	3	12	167

GASEMM1878	UK	Europe	2015	ND	emm1.19	28	1	1	1	1	1	1	1	3	1	1	1	1
GASEMM2496	UK	Europe	2015	ND	emm112.2	497	132	107	4	66	21	61	27	161	8	3	21	113
GASEMM1434	UK	Europe	2015	ND	emm12.37	36	14	3	3	3	2	3	52	59	24	12	38	3
GASEMM2044	UK	Europe	2015	ND	emm5.120	99	14	91	70	1	17	71	114	131	47	22	80	91
GASEMM0500	UK	Europe	2015	ND	emm131.0	785	1	1	1	1	1	1	1	1	1	1	1	1
GASEMM1541	UK	Europe	2015	ND	emm90.2	184	14	88	68	57	94	70	109	127	1	2	79	88
GASEMM0295	UK	Europe	2015	ND	emm5.86	99	14	91	70	1	17	71	114	131	47	22	80	91
GASEMM0584	UK	Europe	2015	ND	emm3.91	15	127	15	10	1	10	32	111	129	1	2	6	31
GASEMM1933	UK	Europe	2015	ND	emm58.8	549	51	61	50	27	63	56	217	267	35	3	35	72
GASEMM0956	UK	Europe	2015	ND	emm131.0	99	14	91	70	1	17	71	114	263	47	22	80	91
GASEMM2201	UK	Europe	2015	ND	emm100.0	119	11	11	116	10	10	7	37	39	83	2	132	31
H293	UK	Europe	ND	invasive, NOS	emm89.0	101	2	2	2	2	2	2	2	2	2	2	2	2
Alab49	USA	North America	1986	ND	emm53.0	11	11	164	10	8	15	54	17	84	1	2	6	20
D471	USA	North America	ND	ARF	emm6.52	37	65	80	62	55	85	62	97	268	11	20	69	82
FDAARGOS_149	USA	North America	2014	invasive, NOS	emm1.0	28	1	1	1	1	1	1	1	134	1	1	1	1
HarveyGAS	USA	North America	2017	necrotizing fasciitis	emm28.0	52	3	3	3	3	2	3	3	4	1	1	3	3
Manfredo	USA	North America	1952	ARF	emm5.0	99	[S]	91	70	1	17	71	114	131	47	22	80	91
MEW123	USA	North America	ND	pharyngitis and/or tonsillitis	emm28.0	52	3	3	3	3	2	3	117	4	1	1	3	3
MEW427	USA	North America	ND	ND	emm4.0	39	4	8	[S]	7	51	9	8	9	7	2	8	8
MGAS10270	USA	North America	2002	pharyngitis and/or tonsillitis	emm2.0	55	32	26	28	24	29	13	43	48	15	3	12	42
MGAS10394	USA	North America	1998	pharyngitis and/or tonsillitis	emm6.4	382	65	80	62	55	85	62	218	130	11	20	69	82
MGAS10750	USA	North America	2001	pharyngitis and/or tonsillitis	emm4.0	1072	4	8	4	7	7	9	8	9	7	2	8	8
MGAS11027	USA	North America	ND	pharyngitis and/or tonsillitis	emm89.0	407	2	2	2	2	2	2	2	269	2	2	2	2
MGAS1882	USA	North America	ND	ND	emm59.0	172	71	84	24	2	93	68	107	122	18	3	75	85
MGAS27061	USA	North America	ND	invasive, NOS	emm89.0	101	2	2	119	58	2	2	2	2	2	2	2	2
MGAS315	USA	North America	ND	invasive, NOS	emm3.1	15	15	15	10	1	10	32	111	129	1	2	6	31
MGAS5005	USA	North America	1996	invasive, NOS	emm1.0	28	1	1	1	1	1	1	1	1	1	1	1	1
MGAS6180	USA	North America	1998	invasive, NOS	emm28.4	52	3	3	3	3	2	3	3	4	1	1	3	3
MGAS8232	USA	North America	1987	ARF	emm18.19	42	75	92	61	5	98	72	115	132	47	2	69	5
MGAS9429	USA	North America	2001	pharyngitis and/or tonsillitis	emm12.0	36	14	3	3	3	2	3	52	59	24	12	38	3
SF370	USA	North America	1985	ND	emm1.6	28	1	1	1	1	1	1	1	270	1	1	1	1
20161543	USA	North America	2015	invasive, NOS	emm106.0	900	1	124	4	70	138	41	154	188	68	3	12	134
20163526	USA	North America	2015	invasive, NOS	emm22.0	46	7	5	127	5	4	5	5	6	4	3	5	5
20162416	USA	North America	2015	invasive, NOS	emm101.0	182	15	31	10	20	15	31	38	41	1	21	32	37
20162417	USA	North America	2015	invasive, NOS	emm41.2	579	11	5	10	51	15	16	77	99	1	2	6	20
20165275	USA	North America	2015	invasive, NOS	emm151.1	433	4	8	4	11	14	14	16	18	84	2	12	13
20162136	USA	North America	2015	invasive, NOS	emm15.1	872	14	48	41	2	2	2	56	13	8	3	43	105
20162134	USA	North America	2015	invasive, NOS	emm151.1	433	4	8	4	11	14	14	16	18	84	2	12	13
20162139	USA	North America	2015	invasive, NOS	emm60.7	53	70	4	3	18	19	19	101	118	42	2	72	3
20160937	USA	North America	2015	invasive, NOS	emm22.0	46	134	5	5	5	4	5	5	6	4	3	5	5
20170001	USA	North America	2015	invasive, NOS	emm33.0	3	11	165	128	8	15	16	219	79	1	2	6	20
20162669	USA	North America	2015	invasive, NOS	emm90.5	184	20	20	14	19	2	23	25	28	15	3	22	25
20161833	USA	North America	2015	invasive, NOS	emm90.2	184	20	20	14	19	2	23	25	28	15	3	22	25
20162642	USA	North America	2015	invasive, NOS	emm1.99	28	1	1	1	1	1	1	1	134	1	1	1	1
20162638	USA	North America	2015	invasive, NOS	emm87.3	62	67	15	10	1	72	58	87	95	1	2	52	74
20162634	USA	North America	2015	invasive, NOS	emm76.4	631	130	36	27	1	36	35	4	46	21	1	4	4
20162633	USA	North America	2015	invasive, NOS	emm11.10	ND	1	97	40	61	20	20	13	139	8	3	18	4
20160980	USA	North America	2015	invasive, NOS	emm49.1	29	4	8	4	11	11	14	12	271	9	2	12	13

20160989	USA	North America	2015	invasive, NOS	emm73.0	331	14	96	3	41	50	13	3	137	48	2	49	94
20161725	USA	North America	2015	invasive, NOS	emm68.1	247	25	26	129	24	29	13	124	48	15	3	12	42
20161207	USA	North America	2015	invasive, NOS	emm25.1	192	14	7	24	2	33	13	13	40	36	3	31	36
20161237	USA	North America	2015	invasive, NOS	emm82.0	896	14	147	3	16	6	18	220	77	1	1	1	3
20162629	USA	North America	2015	invasive, NOS	emm1.0	858	1	1	1	1	1	1	1	134	1	1	1	1
20162594	USA	North America	2015	invasive, NOS	emm80.1	8	16	16	3	16	17	7	20	22	1	2	18	3
20160981	USA	North America	2015	invasive, NOS	emm12.75	36	14	3	3	3	2	3	52	59	24	12	38	3
20162407	USA	North America	2015	invasive, NOS	emm9.0	891	34	77	60	32	2	1	95	113	5	3	84	45
20162410	USA	North America	2015	invasive, NOS	emm87.0	890	67	15	10	1	72	58	87	95	1	2	52	74
20161074	USA	North America	2015	invasive, NOS	emm81.2	901	96	65	130	27	39	19	215	152	85	2	20	22
20160749	USA	North America	2015	invasive, NOS	emm89.0	407	2	2	2	2	2	2	2	89	2	2	2	2
20160756	USA	North America	2015	invasive, NOS	emm29.14	12	11	5	10	20	15	16	17	138	1	2	16	31
20161447	USA	North America	2015	invasive, NOS	emm1.3	28	1	1	1	1	1	1	1	134	1	1	1	1
20161448	USA	North America	2015	invasive, NOS	emm41.2	579	11	5	10	51	15	16	77	99	1	2	6	20
20161432	USA	North America	2015	invasive, NOS	emm11.17	ND	1	97	40	61	20	20	120	139	8	3	85	95
20161430	USA	North America	2015	invasive, NOS	emm68.4	894	25	26	20	24	29	13	124	48	49	3	12	42
20160762	USA	North America	2015	invasive, NOS	emm219.0	25	14	58	125	1	6	18	69	255	1	1	127	162
20164926	USA	North America	2015	invasive, NOS	emm103.0	327	14	13	3	1	66	13	82	87	1	2	49	4
20160970	USA	North America	2015	invasive, NOS	emm9.2	891	34	77	60	32	2	1	95	113	5	3	84	45
20161736	USA	North America	2015	invasive, NOS	emm11.16	ND	1	97	40	61	20	20	120	139	8	3	85	95
20161731	USA	North America	2015	invasive, NOS	emm63.3	385	24	19	7	1	15	101	32	272	17	11	133	3
20161729	USA	North America	2015	invasive, NOS	emm8.0	59	14	13	3	41	50	13	3	273	7	2	49	4
20161730	USA	North America	2015	invasive, NOS	emm63.3	385	24	19	7	1	15	101	32	272	17	11	133	3
20161737	USA	North America	2015	invasive, NOS	emm63.3	385	24	19	7	1	15	101	32	272	17	11	133	3
20161863	USA	North America	2015	invasive, NOS	emm77.0	133	135	37	71	1	179	102	221	274	8	3	134	168
20162158	USA	North America	2015	invasive, NOS	emm151.1	433	4	8	4	11	14	14	16	18	84	2	12	13
20161065	USA	North America	2015	invasive, NOS	emm1.0	858	1	1	1	1	1	1	1	134	1	1	1	1
20161097	USA	North America	2015	invasive, NOS	emm42.0	80	37	32	37	35	43	43	44	60	2	2	39	52
20161201	USA	North America	2015	invasive, NOS	emm83.1	853	11	5	10	8	15	73	97	115	11	2	82	83
20161200	USA	North America	2015	invasive, NOS	emm91.0	12	11	5	10	15	15	16	17	13	1	2	16	18
20161199	USA	North America	2015	invasive, NOS	emm81.0	624	5	53	43	42	52	47	64	119	30	14	30	61
20161203	USA	North America	2015	invasive, NOS	emm82.0	866	73	58	46	1	6	18	69	77	1	1	1	3
20160332	USA	North America	2015	invasive, NOS	emm68.4	894	25	26	20	24	29	13	124	48	49	3	12	42
20160333	USA	North America	2015	invasive, NOS	emm111.2	214	11	11	109	10	180	7	77	275	19	2	30	31
20162430	USA	North America	2015	invasive, NOS	emm2.0	55	32	26	28	24	29	13	43	48	15	3	12	42
20162423	USA	North America	2015	invasive, NOS	emm81.0	909	5	53	43	42	52	47	64	276	24	17	66	61
20162105	USA	North America	2015	invasive, NOS	emm60.7	53	70	4	3	18	19	19	101	118	42	2	72	3
20162060	USA	North America	2015	invasive, NOS	emm73.0	331	14	96	3	41	50	13	3	137	48	2	49	60
20161425	USA	North America	2015	invasive, NOS	emm90.2	184	14	88	68	57	94	70	109	127	1	2	79	88
20160944	USA	North America	2015	invasive, NOS	emm89.0	892	2	2	2	2	2	8	2	2	2	2	2	2
20160946	USA	North America	2015	invasive, NOS	emm24.8	70	11	32	32	16	38	18	77	277	11	7	60	71
20160945	USA	North America	2015	invasive, NOS	emm12.8	36	74	3	3	3	2	3	52	59	24	12	38	3
20161741	USA	North America	2015	invasive, NOS	emm9.0	75	34	77	60	32	2	1	95	113	5	3	11	45
20161832	USA	North America	2015	invasive, NOS	emm60.7	53	70	4	3	18	19	19	101	118	42	2	72	3
20162146	USA	North America	2015	invasive, NOS	emm89.0	101	2	2	2	2	2	2	2	[S]	2	2	2	2
20164918	USA	North America	2015	invasive, NOS	emm4.0	911	4	8	4	7	51	9	8	9	7	2	8	8
20164915	USA	North America	2015	invasive, NOS	emm82.7	36	14	3	3	3	2	3	52	59	24	12	38	3
20161091	USA	North America	2015	invasive, NOS	emm9.0	75	34	77	60	32	2	1	95	113	5	3	11	45

20162124	USA	North America	2015	invasive, NOS	emm28.0	456	3	3	3	3	2	3	3	4	1	1	3	3
20160491	USA	North America	2015	invasive, NOS	emm108.1	14	28	87	10	1	6	63	78	240	1	2	6	31
20162118	USA	North America	2015	invasive, NOS	emm81.3	903	8	166	4	4	181	13	222	42	31	1	135	4
20162110	USA	North America	2015	invasive, NOS	emm90.5	184	20	20	14	19	2	23	25	28	15	3	22	25
20162109	USA	North America	2015	invasive, NOS	emm227.1	28	1	1	1	1	1	1	1	134	1	1	1	1
20162115	USA	North America	2015	invasive, NOS	emm28.0	456	3	3	3	3	2	3	3	4	1	1	3	3
20161333	USA	North America	2015	invasive, NOS	emm2.0	55	32	26	28	24	29	13	43	48	15	3	12	42
20163031	USA	North America	2015	invasive, NOS	emm27.0	308	10	85	65	56	1	69	104	18	9	2	12	13
20160948	USA	North America	2015	invasive, NOS	emm89.0	101	2	2	2	2	2	2	2	2	2	2	2	2
20160953	USA	North America	2015	invasive, NOS	emm77.0	904	14	110	3	22	27	13	26	29	8	3	25	30
20160951	USA	North America	2015	invasive, NOS	emm73.0	331	14	96	3	41	50	13	3	137	48	2	49	60
20160955	USA	North America	2015	invasive, NOS	emm91.0	12	11	5	10	15	15	16	17	13	1	2	16	18
20155415	USA	North America	2015	invasive, NOS	emm11.1	ND	1	97	40	61	20	20	13	139	8	3	18	4
20160028	USA	North America	2015	invasive, NOS	emm63.0	210	5	167	34	27	39	13	84	279	13	2	20	22
20156364	USA	North America	2015	invasive, NOS	emm77.0	399	82	25	3	22	27	13	26	29	8	3	25	30
20155408	USA	North America	2015	invasive, NOS	emm165.0	768	58	7	131	1	76	103	27	102	8	3	61	29
20156321	USA	North America	2015	invasive, NOS	emm75.0	150	25	26	20	24	29	29	33	48	15	3	12	33
20156324	USA	North America	2015	invasive, NOS	emm77.0	399	82	25	3	22	27	13	26	29	8	3	25	30
20156359	USA	North America	2015	invasive, NOS	emm58.2	176	14	13	10	8	13	13	194	47	10	2	34	31
20156362	USA	North America	2015	invasive, NOS	emm22.0	46	7	5	127	5	4	5	5	6	4	3	5	5
20155617	USA	North America	2015	invasive, NOS	emm75.0	49	25	26	20	24	29	74	33	136	15	3	12	33
20156430	USA	North America	2015	invasive, NOS	emm81.0	837	5	53	43	42	52	47	64	119	30	14	30	61
20156432	USA	North America	2015	invasive, NOS	emm238.2	867	1	1	1	1	1	15	14	93	5	6	14	15
20156319	USA	North America	2015	invasive, NOS	emm68.3	247	25	26	4	24	29	13	124	48	15	3	12	42
20155197	USA	North America	2015	invasive, NOS	emm81.0	624	5	53	43	42	52	47	64	119	86	14	30	61
20155204	USA	North America	2015	invasive, NOS	emm9.2	891	34	77	60	32	2	1	95	113	5	3	84	45
20155202	USA	North America	2015	invasive, NOS	emm87.0	62	67	15	10	1	72	58	87	95	23	2	52	74
20155579	USA	North America	2015	invasive, NOS	emm77.0	133	135	37	71	1	179	102	221	274	8	3	134	168
20155417	USA	North America	2015	invasive, NOS	emm44.0	641	5	7	7	1	81	13	26	280	8	18	25	30
20155580	USA	North America	2015	invasive, NOS	emm77.0	133	135	37	71	1	179	102	221	274	8	3	134	168
20155581	USA	North America	2015	invasive, NOS	emm89.0	407	2	2	2	2	2	2	2	281	2	2	2	2
20155208	USA	North America	2015	invasive, NOS	emm4.0	39	4	8	4	7	51	9	8	9	7	2	8	8
20155209	USA	North America	2015	invasive, NOS	emm76.0	50	136	3	3	3	2	3	52	59	24	12	38	3
20155267	USA	North America	2015	invasive, NOS	emm49.0	433	4	8	4	11	14	14	16	18	84	2	12	13
20155269	USA	North America	2015	invasive, NOS	emm87.0	62	67	15	10	1	72	58	87	95	1	2	52	74
20160177	USA	North America	2015	invasive, NOS	emm28.0	458	3	3	3	3	2	3	3	4	1	1	3	3
20156635	USA	North America	2015	invasive, NOS	emm28.0	859	3	3	3	3	2	3	117	4	1	1	3	3
20156154	USA	North America	2015	invasive, NOS	emm81.0	837	5	53	43	42	52	47	64	119	30	14	30	61
20160329	USA	North America	2015	invasive, NOS	emm101.0	182	15	31	10	20	15	31	38	41	1	21	32	169
20156401	USA	North America	2015	invasive, NOS	emm83.1	5	11	5	10	8	15	65	97	282	11	2	69	83
20156400	USA	North America	2015	invasive, NOS	emm104.0	137	8	168	71	1	1	64	70	1	1	1	1	3
20160016	USA	North America	2015	invasive, NOS	emm28.0	458	3	3	3	3	2	3	3	4	1	1	3	3
20156270	USA	North America	2015	invasive, NOS	emm82.0	866	73	58	46	1	6	18	69	77	1	1	1	3
20156271	USA	North America	2015	invasive, NOS	emm75.0	150	25	26	20	24	29	29	33	155	15	3	12	33
20155380	USA	North America	2015	invasive, NOS	emm58.0	176	14	94	10	8	13	13	15	47	10	2	34	31
20155381	USA	North America	2015	invasive, NOS	emm81.0	837	5	53	43	42	52	47	64	119	30	14	30	61
20160140	USA	North America	2015	invasive, NOS	emm12.91	36	14	3	3	3	2	3	52	59	24	12	38	3
20156447	USA	North America	2015	invasive, NOS	emm12.32	36	14	3	3	3	2	3	52	59	24	12	38	3

20156102	USA	North America	2015	invasive, NOS	emm118.0	863	11	32	52	1	67	13	118	135	1	23	83	93
20156448	USA	North America	2015	invasive, NOS	emm104.0	137	8	168	71	1	1	64	70	1	1	1	1	3
20156019	USA	North America	2015	invasive, NOS	emm6.80	382	65	80	62	55	85	62	97	130	11	20	69	82
20155585	USA	North America	2015	invasive, NOS	emm77.0	133	135	37	71	1	179	102	221	274	8	3	134	168
20155589	USA	North America	2015	invasive, NOS	emm75.0	861	25	26	20	24	29	74	33	283	15	3	12	33
20155588	USA	North America	2015	invasive, NOS	emm75.0	150	25	26	20	24	29	29	33	48	15	3	12	33
20160010	USA	North America	2015	invasive, NOS	emm89.0	101	2	2	2	58	2	2	2	2	2	2	2	2
20156107	USA	North America	2015	invasive, NOS	emm76.0	50	14	3	3	3	2	3	52	59	24	12	38	3
20156105	USA	North America	2015	invasive, NOS	emm59.0	172	72	84	24	2	93	68	107	122	18	3	75	85
20156103	USA	North America	2015	invasive, NOS	emm58.0	176	14	94	10	8	13	13	15	47	10	2	34	31
20155639	USA	North America	2015	invasive, NOS	emm76.0	50	14	3	3	3	2	3	52	59	24	12	38	3
20160313	USA	North America	2015	invasive, NOS	emm11.18	ND	1	97	40	61	20	20	13	139	8	3	18	4
20156730	USA	North America	2015	invasive, NOS	emm68.3	247	25	26	4	24	29	13	124	48	15	3	12	42
20156725	USA	North America	2015	invasive, NOS	emm102.2	895	14	93	71	60	99	13	116	133	13	2	81	92
20156726	USA	North America	2015	invasive, NOS	emm58.0	176	14	94	10	8	13	13	15	47	10	2	34	31
20156185	USA	North America	2015	invasive, NOS	emm114.6	220	18	136	103	43	148	40	26	56	5	27	83	147
20156188	USA	North America	2015	invasive, NOS	emm25.1	192	14	7	24	2	33	13	13	40	36	3	31	36
20155373	USA	North America	2015	invasive, NOS	emm9.0	75	34	77	60	32	2	1	95	113	5	3	11	45
20156592	USA	North America	2015	invasive, NOS	emm83.1	853	11	5	10	8	15	73	97	115	11	2	82	83
20160031	USA	North America	2015	invasive, NOS	emm232.0	902	14	37	31	3	2	38	47	242	1	2	58	25
20156457	USA	North America	2015	invasive, NOS	emm92.2	82	17	4	3	18	19	19	22	24	13	2	20	22
20155603	USA	North America	2015	invasive, NOS	emm9.2	891	34	77	60	32	2	1	95	113	5	3	84	45
20156711	USA	North America	2015	invasive, NOS	emm18.7	ND	75	92	61	5	98	72	115	132	47	2	69	5
20155386	USA	North America	2015	invasive, NOS	emm83.1	853	11	5	10	8	15	73	97	115	11	2	82	83
20155657	USA	North America	2015	invasive, NOS	emm49.0	862	4	8	4	11	14	14	16	18	9	2	12	13
20155393	USA	North America	2015	invasive, NOS	emm87.0	62	67	15	10	1	72	58	87	95	1	2	52	74
20156790	USA	North America	2015	invasive, NOS	emm28.0	458	3	3	3	3	2	3	3	4	1	1	3	3
20156794	USA	North America	2015	invasive, NOS	emm28.0	458	3	3	3	3	2	3	3	4	1	1	3	3
20156713	USA	North America	2015	invasive, NOS	emm9.0	75	34	77	60	32	2	1	95	113	5	3	11	45
20156717	USA	North America	2015	invasive, NOS	emm11.16	ND	1	97	40	61	20	20	120	139	8	3	85	95
20156781	USA	North America	2015	invasive, NOS	emm8.0	59	14	13	3	41	50	13	3	273	7	2	49	4
20155615	USA	North America	2015	invasive, NOS	emm27.0	308	10	85	65	56	1	69	104	[S]	9	2	12	13
20155609	USA	North America	2015	invasive, NOS	emm75.0	49	25	26	20	24	29	74	33	136	15	3	12	33
20155360	USA	North America	2015	invasive, NOS	emm89.0	407	2	2	2	2	2	2	2	89	2	2	2	170
20155356	USA	North America	2015	invasive, NOS	emm81.0	837	5	53	43	42	52	47	64	119	30	14	30	61
20156595	USA	North America	2015	invasive, NOS	emm8.0	869	18	169	4	8	64	50	223	285	18	3	83	171
20160246	USA	North America	2015	invasive, NOS	emm82.0	866	73	58	46	1	6	18	69	77	1	1	1	3
20156593	USA	North America	2015	invasive, NOS	emm238.1	868	1	20	123	1	1	15	14	16	5	6	14	15
20156622	USA	North America	2015	invasive, NOS	emm73.0	331	14	96	3	41	50	13	3	286	48	2	49	60
20156597	USA	North America	2015	invasive, NOS	emm91.0	12	11	5	10	15	15	16	17	13	1	2	16	18
20156169	USA	North America	2015	invasive, NOS	emm63.3	385	24	19	7	1	15	101	32	272	17	11	133	3
20156012	USA	North America	2015	invasive, NOS	emm77.0	399	82	25	3	22	27	13	26	29	8	3	25	30
20156165	USA	North America	2015	invasive, NOS	emm101.0	182	15	31	10	20	15	31	38	41	1	21	32	37
20155387	USA	North America	2015	invasive, NOS	emm89.0	860	2	2	2	58	2	2	2	2	2	2	2	2
20155047	USA	North America	2015	invasive, NOS	emm5.128	99	14	91	70	1	17	71	114	131	47	22	80	91
20155046	USA	North America	2015	invasive, NOS	emm5.18	99	14	91	70	1	17	71	114	131	47	22	80	91
20155042	USA	North America	2015	invasive, NOS	emm101.0	182	15	31	10	20	15	31	38	41	1	21	32	37
20156410	USA	North America	2015	invasive, NOS	emm12.75	36	14	170	3	3	2	3	52	59	24	12	38	3

20156414	USA	North America	2015	invasive, NOS	emm1.0	897	1	1	1	1	1	1	224	134	1	1	1	1
20156413	USA	North America	2015	invasive, NOS	emm49.0	433	4	8	4	11	14	14	16	18	84	2	12	13
20154297	USA	North America	2015	invasive, NOS	emm59.2	864	72	84	24	2	93	68	107	122	18	3	75	85
20154375	USA	North America	2015	invasive, NOS	emm11.0	ND	1	6	40	78	20	20	13	139	8	3	18	4
20154055	USA	North America	2015	invasive, NOS	emm92.0	855	17	4	3	18	19	19	22	24	13	2	20	22
20154137	USA	North America	2015	invasive, NOS	emm12.7	36	14	3	3	3	2	3	52	59	24	12	38	3
20154144	USA	North America	2015	invasive, NOS	emm41.2	579	11	5	10	51	15	16	77	99	1	2	6	20
20154142	USA	North America	2015	invasive, NOS	emm118.0	167	11	32	52	1	67	13	118	135	1	23	83	93
20154591	USA	North America	2015	invasive, NOS	emm89.0	801	137	2	2	58	2	2	2	2	2	2	2	2
20154590	USA	North America	2015	invasive, NOS	emm219.0	25	14	58	125	1	6	18	69	255	1	1	127	162
20155018	USA	North America	2015	invasive, NOS	emm77.0	63	23	24	19	1	26	28	30	33	8	3	24	172
20155015	USA	North America	2015	invasive, NOS	emm83.1	5	11	5	10	8	15	65	97	282	11	2	69	83
20154949	USA	North America	2015	invasive, NOS	emm83.1	853	11	5	10	8	15	73	97	115	11	2	82	83
20154031	USA	North America	2015	invasive, NOS	emm12.0	36	14	3	3	3	2	3	52	287	24	12	38	3
20154030	USA	North America	2015	invasive, NOS	emm76.0	50	14	3	3	3	2	3	52	59	24	12	38	3
20154029	USA	North America	2015	invasive, NOS	emm82.0	334	138	58	46	1	6	18	69	288	1	1	1	3
20154028	USA	North America	2015	invasive, NOS	emm60.7	53	70	4	3	18	19	19	101	118	42	2	72	3
20154026	USA	North America	2015	invasive, NOS	emm89.0	101	2	2	2	58	2	2	2	2	2	2	2	2
20154024	USA	North America	2015	invasive, NOS	emm28.0	52	3	3	3	3	2	3	3	4	1	1	3	3
20154025	USA	North America	2015	invasive, NOS	emm11.0	ND	1	97	40	61	20	20	120	139	8	3	85	95
20154805	USA	North America	2015	invasive, NOS	emm81.0	624	5	53	43	42	52	47	64	119	30	14	30	61
20154799	USA	North America	2015	invasive, NOS	emm3.1	15	15	15	10	1	10	32	111	129	1	2	6	31
20154797	USA	North America	2015	invasive, NOS	emm6.101	382	65	80	62	55	182	62	97	130	11	20	69	82
20154803	USA	North America	2015	invasive, NOS	emm1.0	899	1	1	1	1	1	1	1	1	1	1	1	1
20154293	USA	North America	2015	invasive, NOS	emm59.0	864	72	84	24	2	93	68	107	122	18	3	75	85
20154292	USA	North America	2015	invasive, NOS	emm87.0	62	67	15	10	1	72	58	87	95	1	2	52	74
20154289	USA	North America	2015	invasive, NOS	emm59.0	864	72	84	24	2	93	68	107	122	18	3	75	85
20154288	USA	North America	2015	invasive, NOS	emm59.2	864	72	84	24	2	93	68	107	122	18	3	75	85
20154168	USA	North America	2015	invasive, NOS	emm12.7	36	14	3	3	3	2	3	52	59	24	12	38	3
20154167	USA	North America	2015	invasive, NOS	emm4.0	39	4	8	4	7	51	9	8	9	7	2	8	8
20154813	USA	North America	2015	invasive, NOS	emm3.4	15	15	15	10	1	10	32	111	129	1	2	6	31
20154042	USA	North America	2015	invasive, NOS	emm108.2	14	28	87	10	1	183	91	78	240	1	2	6	31
20154038	USA	North America	2015	invasive, NOS	emm92.0	82	17	4	3	18	19	19	22	24	13	2	20	22
20154035	USA	North America	2015	invasive, NOS	emm12.0	36	14	3	3	3	2	3	52	252	24	12	38	3
20154043	USA	North America	2015	invasive, NOS	emm83.1	5	11	5	10	8	15	65	97	282	11	2	69	83
20154036	USA	North America	2015	invasive, NOS	emm92.0	82	17	4	3	18	19	19	22	24	13	2	20	22
20154044	USA	North America	2015	invasive, NOS	emm4.0	39	4	8	4	7	51	9	8	9	7	2	8	8
20154565	USA	North America	2015	invasive, NOS	emm89.0	910	2	2	2	2	2	2	2	89	2	2	2	2
20154558	USA	North America	2015	invasive, NOS	emm77.0	63	23	24	57	1	184	28	30	33	8	3	24	29
20154557	USA	North America	2015	invasive, NOS	emm1.0	856	1	1	1	1	1	1	224	134	1	1	1	1
20154555	USA	North America	2015	invasive, NOS	emm28.0	52	3	3	3	3	2	3	117	4	1	1	3	3
20154563	USA	North America	2015	invasive, NOS	emm106.0	338	14	27	132	25	106	19	225	36	15	3	27	34
20154623	USA	North America	2015	invasive, NOS	emm1.49	28	1	1	1	1	1	1	1	134	1	1	1	1
20154628	USA	North America	2015	invasive, NOS	emm75.0	150	25	26	20	24	29	29	33	48	15	3	12	33
20154166	USA	North America	2015	invasive, NOS	emm3.1	15	15	15	10	1	10	32	111	129	1	2	6	31
20154161	USA	North America	2015	invasive, NOS	emm59.0	172	72	84	24	79	93	68	107	122	18	3	75	85
20154158	USA	North America	2015	invasive, NOS	emm11.0	ND	1	97	40	61	20	20	120	139	8	3	85	95
20154946	USA	North America	2015	invasive, NOS	emm12.7	36	14	3	3	3	2	3	52	59	24	12	38	3

20154547	USA	North America	2015	invasive, NOS	emm89.0	407	2	2	2	2	2	2	2	89	2	2	2	170
20154548	USA	North America	2015	invasive, NOS	emm89.0	101	2	2	2	2	2	2	2	2	2	2	2	2
20154550	USA	North America	2015	invasive, NOS	emm6.0	382	65	80	133	55	85	62	97	130	11	20	69	82
20154944	USA	North America	2015	invasive, NOS	emm28.0	850	3	3	3	3	2	3	3	4	1	1	3	3
20154821	USA	North America	2015	invasive, NOS	emm1.0	858	1	1	1	1	1	1	1	134	1	1	1	1
20155031	USA	North America	2015	invasive, NOS	emm3.1	15	15	15	10	1	10	32	111	129	1	2	6	31
20155025	USA	North America	2015	invasive, NOS	emm54.1	302	11	5	10	20	15	16	17	13	1	2	16	31
20154049	USA	North America	2015	invasive, NOS	emm12.0	36	14	3	3	3	2	3	52	287	24	12	38	3
20154051	USA	North America	2015	invasive, NOS	emm82.0	36	14	3	3	3	2	3	52	59	24	12	38	3
20154046	USA	North America	2015	invasive, NOS	emm18.12	535	14	57	2	2	2	78	67	75	8	3	53	2
20154045	USA	North America	2015	invasive, NOS	emm77.0	63	23	24	57	1	26	28	30	33	8	3	24	29
20154048	USA	North America	2015	invasive, NOS	emm49.0	433	4	8	4	11	14	14	16	18	84	2	12	13
20154047	USA	North America	2015	invasive, NOS	emm49.0	433	4	8	4	11	14	14	16	18	84	2	12	13
20154054	USA	North America	2015	invasive, NOS	emm92.0	82	17	4	3	18	19	19	22	24	13	2	20	22
20154053	USA	North America	2015	invasive, NOS	emm92.0	82	17	4	3	18	19	19	22	24	13	2	20	22
20154615	USA	North America	2015	invasive, NOS	emm75.0	49	25	26	20	24	29	74	33	283	15	3	12	33
20154616	USA	North America	2015	invasive, NOS	emm6.64	382	65	80	62	55	185	62	97	130	11	20	69	82
20154612	USA	North America	2015	invasive, NOS	emm77.0	399	82	25	3	22	27	13	26	29	8	3	25	30
20154146	USA	North America	2015	invasive, NOS	emm28.0	52	3	3	3	3	2	3	117	4	1	1	3	3
20154630	USA	North America	2015	invasive, NOS	emm118.0	167	11	32	52	1	67	13	118	135	1	23	83	93
20154145	USA	North America	2015	invasive, NOS	emm1.29	28	1	1	1	1	1	1	1	134	1	1	1	1
20154679	USA	North America	2015	invasive, NOS	emm81.0	624	5	53	43	42	52	47	64	119	30	14	30	61
20154138	USA	North America	2015	invasive, NOS	emm4.0	39	4	8	4	7	51	9	8	9	7	2	8	8
20154143	USA	North America	2015	invasive, NOS	emm6.0	382	65	80	134	55	85	62	97	130	11	20	69	82
20154156	USA	North America	2015	invasive, NOS	emm6.0	382	65	80	62	55	85	62	97	130	11	20	69	82
20154155	USA	North America	2015	invasive, NOS	emm2.0	55	32	26	28	24	29	13	226	48	15	3	12	42
20154151	USA	North America	2015	invasive, NOS	emm77.0	63	23	24	19	1	26	28	30	33	8	3	24	29
20154148	USA	North America	2015	invasive, NOS	emm12.7	36	14	3	3	3	2	3	52	59	24	12	38	3
20154153	USA	North America	2015	invasive, NOS	emm83.1	5	11	5	10	8	15	65	97	282	11	2	69	83
20154154	USA	North America	2015	invasive, NOS	emm118.0	167	11	32	52	1	67	13	118	135	1	23	83	93
20154147	USA	North America	2015	invasive, NOS	emm28.0	52	3	171	3	3	2	3	117	4	1	1	3	3
20154150	USA	North America	2015	invasive, NOS	emm2.0	55	32	172	118	24	29	13	43	48	15	3	12	42
20154149	USA	North America	2015	invasive, NOS	emm2.0	55	32	172	28	24	29	13	43	48	15	3	12	42
20154952	USA	North America	2015	invasive, NOS	emm59.0	172	72	84	24	2	93	68	107	122	18	3	75	85
20154135	USA	North America	2015	invasive, NOS	emm4.0	39	4	8	4	7	51	9	8	9	7	2	8	8
20154131	USA	North America	2015	invasive, NOS	emm2.0	55	32	172	118	24	29	13	43	48	15	3	12	42
20154133	USA	North America	2015	invasive, NOS	emm3.1	15	15	15	10	1	10	32	111	129	1	2	6	31
20154127	USA	North America	2015	invasive, NOS	emm1.0	28	1	1	1	1	1	1	224	134	1	1	1	1
20154129	USA	North America	2015	invasive, NOS	emm12.0	36	14	173	3	3	2	3	52	59	24	12	38	3
20154384	USA	North America	2015	invasive, NOS	emm75.0	49	25	26	20	24	29	74	33	136	87	3	12	33
20154381	USA	North America	2015	invasive, NOS	emm76.4	631	130	36	27	1	36	35	4	46	21	1	4	4
20154386	USA	North America	2015	invasive, NOS	emm11.0	ND	1	6	40	61	20	20	13	139	8	3	18	4
20154796	USA	North America	2015	invasive, NOS	emm28.0	859	3	3	3	3	2	3	117	4	1	1	3	3
20154787	USA	North America	2015	invasive, NOS	emm1.0	858	1	1	1	1	1	1	1	134	1	1	1	1
20154017	USA	North America	2015	invasive, NOS	emm89.0	101	2	2	2	58	2	2	2	2	2	2	2	2
20154018	USA	North America	2015	invasive, NOS	emm89.0	101	2	2	2	2	2	2	2	2	2	2	2	2
20154019	USA	North America	2015	invasive, NOS	emm82.0	334	138	58	46	1	6	18	69	288	1	1	1	3
20154020	USA	North America	2015	invasive, NOS	emm89.0	101	2	2	2	2	2	2	2	2	2	2	2	2

20154021	USA	North America	2015	invasive, NOS	emm81.0	624	5	53	43	42	52	47	64	119	30	14	30	173
20154023	USA	North America	2015	invasive, NOS	emm82.0	334	138	58	46	1	6	18	69	288	1	1	1	3
20154022	USA	North America	2015	invasive, NOS	emm82.0	334	138	58	46	1	6	18	69	288	1	1	1	3
20154605	USA	North America	2015	invasive, NOS	emm114.8	188	14	18	67	16	21	61	108	26	46	3	78	87
20154599	USA	North America	2015	invasive, NOS	emm59.0	172	72	84	24	2	93	68	107	122	18	3	75	85
20154600	USA	North America	2015	invasive, NOS	emm118.0	167	11	32	52	1	67	13	118	135	1	23	83	93
20154792	USA	North America	2015	invasive, NOS	emm77.0	63	23	24	19	1	26	28	30	33	8	3	24	29
20154607	USA	North America	2015	invasive, NOS	emm6.0	382	65	80	62	55	85	62	97	130	11	20	69	82

Footnotes

ND = not determinded

^ primary disease: APSGN = acute post-streptococcal glomerul nephritis; ARF = acute rheumatic fever; NOS = not otherwise specified

number indicates allele number of *gac* gene; allele sequences of *gac* genes can be downloaded from PubMLST; [S] indicates gene with premature stopcodon

Supplementary Table S5 Overview of SNPs in gac gene cluster including intergenic regions in 2,021 S. pyogenes isolates

position	REF	ALT	annotation 1	S/NS	gene	locus_tag	nucl_sub	AA_sub	freq (n=2021)	annotation 2	S/NS 2	gene	nucl_sub 2	AA_sub 2	freq 2 (n=2021)
7	G	A	A	upstream_gene_variant	intergenic				4						0
12	G	A	A	upstream_gene_variant	intergenic				7						0
13	G	A	A	upstream_gene_variant	intergenic				6						0
20	A	G	G	upstream_gene_variant	intergenic				1						0
21	T	A	A	upstream_gene_variant	intergenic				1						0
23	T	C	C	upstream_gene_variant	intergenic				1						0
24	T	C	C	upstream_gene_variant	intergenic				5						0
35	A	G	G	upstream_gene_variant	intergenic				1						0
45	A	G	G	upstream_gene_variant	intergenic				11						0
47	A	G	G	upstream_gene_variant	intergenic				2						0
48	G	A	A	upstream_gene_variant	intergenic				2						0
51	A	G	G	upstream_gene_variant	intergenic				55						0
59	T	C	C	upstream_gene_variant	intergenic				3						0
69	T	C	C	upstream_gene_variant	intergenic				5						0
70	G	A	A	upstream_gene_variant	intergenic				85						0
73	T	G	G	upstream_gene_variant	intergenic				302						0
92	G	A	A	upstream_gene_variant	intergenic				12						0
93	A	T	T	upstream_gene_variant	intergenic				4						0
101	G	A	A	upstream_gene_variant	intergenic				151						0
105	A	G	G	upstream_gene_variant	intergenic				7						0
108	G	A	A	upstream_gene_variant	intergenic				1						0
111	C	A	A	upstream_gene_variant	intergenic				14						0
115	C	T	T	upstream_gene_variant	intergenic				75						0
119	A	G	G	upstream_gene_variant	intergenic				1						0
121	G	A	A	upstream_gene_variant	intergenic				6						0
122	G	A,T	A	upstream_gene_variant	intergenic				3	T	upstream_gene_variant	intergenic	c.-3759C>A		6
126	A	G	G	upstream_gene_variant	intergenic				3						0
147	A	G	G	missense_variant	gacA	M5005_Spy0602	c.10A>G	p.Ile4Val	4						0
158	C	T	T	synonymous_variant	gacA	M5005_Spy0602	c.21C>T	p.Ser7Ser	11						0
173	G	A	A	synonymous_variant	gacA	M5005_Spy0602	c.36G>A	p.Gly12Gly	34						0
176	A	G	G	synonymous_variant	gacA	M5005_Spy0602	c.39A>G	p.Thr13Thr	20						0
188	C	T	T	synonymous_variant	gacA	M5005_Spy0602	c.51C>T	p.Tyr17Tyr	8						0
204	G	A	A	missense_variant	gacA	M5005_Spy0602	c.67G>A	p.Gly23Ser	22						0
205	G	A	A	missense_variant	gacA	M5005_Spy0602	c.68G>A	p.Gly23Asp	1						0
211	A	G	G	missense_variant	gacA	M5005_Spy0602	c.74A>G	p.Asp25Gly	19						0
224	T	G	G	synonymous_variant	gacA	M5005_Spy0602	c.87T>G	p.Val29Val	1						0
233	A	G	G	synonymous_variant	gacA	M5005_Spy0602	c.96A>G	p.Ala32Ala	8						0
255	G	A	A	missense_variant	gacA	M5005_Spy0602	c.118G>A	p.Asp40Asn	1						0
257	C	A,T	A	missense_variant	gacA	M5005_Spy0602	c.120C>A	p.Asp40Glu	4	T	synonymous_variant	gacA	c.120C>T	p.Asp40Asp	1
258	A	G	G	missense_variant	gacA	M5005_Spy0602	c.121A>G	p.Lys41Glu	1						0
267	G	A	A	missense_variant	gacA	M5005_Spy0602	c.130G>A	p.Ala44Thr	11						0
270	G	A	A	missense_variant	gacA	M5005_Spy0602	c.133G>A	p.Val45Ile	8						0
277	C	T	T	missense_variant	gacA	M5005_Spy0602	c.140C>T	p.Ala47Val	5						0
284	C	T	T	synonymous_variant	gacA	M5005_Spy0602	c.147C>T	p.Val49Val	7						0
287	G	A	A	synonymous_variant	gacA	M5005_Spy0602	c.150G>A	p.Lys50Lys	3						0
292	C	G	G	missense_variant	gacA	M5005_Spy0602	c.155C>G	p.Thr52Arg	1						0
298	T	C	C	missense_variant	gacA	M5005_Spy0602	c.161T>C	p.Val54Ala	2						0
310	C	T	T	missense_variant	gacA	M5005_Spy0602	c.173C>T	p.Ala58Val	1						0
321	G	A	A	missense_variant	gacA	M5005_Spy0602	c.184G>A	p.Ala62Thr	15						0
331	C	T	T	missense_variant	gacA	M5005_Spy0602	c.194C>T	p.Ala65Val	12						0
340	A	G	G	missense_variant	gacA	M5005_Spy0602	c.203A>G	p.Asp68Gly	2						0
341	C	T	T	synonymous_variant	gacA	M5005_Spy0602	c.204C>T	p.Asp68Asp	15						0
342	G	A	A	missense_variant	gacA	M5005_Spy0602	c.205G>A	p.Glu69Lys	2						0
351	G	A	A	missense_variant	gacA	M5005_Spy0602	c.214G>A	p.Ala72Thr	38						0
352	C	T	T	missense_variant	gacA	M5005_Spy0602	c.215C>T	p.Ala72Val	13						0
353	C	T	T	synonymous_variant	gacA	M5005_Spy0602	c.216C>T	p.Ala72Ala	131						0
364	C	A	A	missense_variant	gacA	M5005_Spy0602	c.227C>A	p.Ala76Asp	4						0
375	A	G	G	missense_variant	gacA	M5005_Spy0602	c.238A>G	p.Thr80Ala	24						0
380	C	A	A	synonymous_variant	gacA	M5005_Spy0602	c.243C>A	p.Gly81Gly	45						0
386	A	G	G	synonymous_variant	gacA	M5005_Spy0602	c.249A>G	p.Glu83Glu	1						0

399	G	A	A	missense_variant	gacA	M5005_Spy0602	c.262G>A	p.Ala88Thr	4						0
400	C	T	T	missense_variant	gacA	M5005_Spy0602	c.263C>T	p.Ala88Val	4						0
412	A	G	G	missense_variant	gacA	M5005_Spy0602	c.275A>G	p.Tyr92Cys	3						0
416	G	A	A	synonymous_variant	gacA	M5005_Spy0602	c.279G>A	p.Gly93Gly	14						0
417	G	A	A	missense_variant	gacA	M5005_Spy0602	c.280G>A	p.Ala94Thr	4						0
420	A	G	G	missense_variant	gacA	M5005_Spy0602	c.283A>G	p.Thr95Ala	13						0
422	C	T	T	synonymous_variant	gacA	M5005_Spy0602	c.285C>T	p.Thr95Thr	21						0
431	C	T	T	synonymous_variant	gacA	M5005_Spy0602	c.294C>T	p.Tyr98Tyr	6						0
432	A	G	G	missense_variant	gacA	M5005_Spy0602	c.295A>G	p.Ile99Val	56						0
455	T	C	C	synonymous_variant	gacA	M5005_Spy0602	c.318T>C	p.Asp106Asp	17						0
458	C	T	T	synonymous_variant	gacA	M5005_Spy0602	c.321C>T	p.Gly107Gly	2						0
468	G	A	A	missense_variant	gacA	M5005_Spy0602	c.331G>A	p.Val111Ile	1						0
484	T	C	C	missense_variant	gacA	M5005_Spy0602	c.347T>C	p.Val116Ala	2						0
488	A	G	G	synonymous_variant	gacA	M5005_Spy0602	c.351A>G	p.Glu117Glu	6						0
490	C	T	T	missense_variant	gacA	M5005_Spy0602	c.353C>T	p.Thr118Ile	2						0
495	C	T	T	missense_variant	gacA	M5005_Spy0602	c.358C>T	p.His120Tyr	131						0
496	A	G	G	missense_variant	gacA	M5005_Spy0602	c.359A>G	p.His120Arg	1838						0
503	C	T	T	synonymous_variant	gacA	M5005_Spy0602	c.366C>T	p.Asp122Asp	3						0
507	A	G	G	missense_variant	gacA	M5005_Spy0602	c.370A>G	p.Lys124Glu	1						0
508	A	C	C	missense_variant	gacA	M5005_Spy0602	c.371A>C	p.Lys124Thr	1						0
521	C	T	T	synonymous_variant	gacA	M5005_Spy0602	c.384C>T	p.Gly128Gly	1						0
524	T	C	C	synonymous_variant	gacA	M5005_Spy0602	c.387T>C	p.Arg129Arg	3						0
532	G	A	A	missense_variant	gacA	M5005_Spy0602	c.395G>A	p.Arg132His	1						0
542	A	G	G	synonymous_variant	gacA	M5005_Spy0602	c.405A>G	p.Glu135Glu	40						0
543	C	T	T	synonymous_variant	gacA	M5005_Spy0602	c.406C>T	p.Leu136Leu	3						0
555	C	G	G	missense_variant	gacA	M5005_Spy0602	c.418C>G	p.Arg140Gly	6						0
560	C	T	T	synonymous_variant	gacA	M5005_Spy0602	c.423C>T	p.Tyr141Tyr	64						0
566	G	A	A	synonymous_variant	gacA	M5005_Spy0602	c.429G>A	p.Glu143Glu	4						0
567	C	T	T	missense_variant	gacA	M5005_Spy0602	c.430C>T	p.His144Tyr	30						0
577	T	G	G	missense_variant	gacA	M5005_Spy0602	c.440T>G	p.Ile147Ser	5						0
581	C	T	T	synonymous_variant	gacA	M5005_Spy0602	c.444C>T	p.Ile148Ile	1						0
602	A	G	G	synonymous_variant	gacA	M5005_Spy0602	c.465A>G	p.Gly155Gly	7						0
617	C	T	T	synonymous_variant	gacA	M5005_Spy0602	c.480C>T	p.Asn160Asn	1						0
627	A	G	G	missense_variant	gacA	M5005_Spy0602	c.490A>G	p.Thr164Ala	34						0
629	C	T	T	synonymous_variant	gacA	M5005_Spy0602	c.492C>T	p.Thr164Thr	18						0
630	A	C	C	missense_variant	gacA	M5005_Spy0602	c.493A>C	p.Met165Leu	2						0
632	G	A	A	missense_variant	gacA	M5005_Spy0602	c.495G>A	p.Met165Ile	43						0
635	G	A	A	synonymous_variant	gacA	M5005_Spy0602	c.498G>A	p.Glu166Glu	610						0
637	A	G	G	missense_variant	gacA	M5005_Spy0602	c.500A>G	p.Gln167Arg	6						0
638	G	A	A	synonymous_variant	gacA	M5005_Spy0602	c.501G>A	p.Gln167Gln	1						0
644	A	G	G	synonymous_variant	gacA	M5005_Spy0602	c.507A>G	p.Ala169Ala	1757						0
650	T	C	C	synonymous_variant	gacA	M5005_Spy0602	c.513T>C	p.Asn171Asn	2						0
653	C	T	T	synonymous_variant	gacA	M5005_Spy0602	c.516C>T	p.His172His	3						0
657	C	T	T	missense_variant	gacA	M5005_Spy0602	c.520C>T	p.Arg174Cys	21						0
666	G	A	A	missense_variant	gacA	M5005_Spy0602	c.529G>A	p.Val177Ile	1						0
674	T	A	A	missense_variant	gacA	M5005_Spy0602	c.537T>A	p.Asn179Lys	1						0
680	A	G	G	synonymous_variant	gacA	M5005_Spy0602	c.543A>G	p.Gln181Gln	1						0
681	C	T	T	missense_variant	gacA	M5005_Spy0602	c.544C>T	p.His182Tyr	4						0
683	C	T	T	synonymous_variant	gacA	M5005_Spy0602	c.546C>T	p.His182His	6						0
704	G	A	A	synonymous_variant	gacA	M5005_Spy0602	c.567G>A	p.Arg189Arg	1813						0
719	C	T	T	synonymous_variant	gacA	M5005_Spy0602	c.582C>T	p.Phe194Phe	1						0
722	G	T	T	missense_variant	gacA	M5005_Spy0602	c.585G>T	p.Met195Ile	1						0
723	T	A	A	missense_variant	gacA	M5005_Spy0602	c.586T>A	p.Cys196Ser	2						0
724	G	A	A	missense_variant	gacA	M5005_Spy0602	c.587G>A	p.Cys196Tyr	6						0
726	T	C	C	missense_variant	gacA	M5005_Spy0602	c.589T>C	p.Tyr197His	55						0
752	T	C	C	synonymous_variant	gacA	M5005_Spy0602	c.615T>C	p.Phe205Phe	1						0
757	A	G	G	missense_variant	gacA	M5005_Spy0602	c.620A>G	p.Tyr207Cys	5						0
758	C	T	T	synonymous_variant	gacA	M5005_Spy0602	c.621C>T	p.Tyr207Tyr	17						0
782	G	T	T	missense_variant	gacA	M5005_Spy0602	c.645G>T	p.Lys215Asn	4						0
791	C	T	T	synonymous_variant	gacA	M5005_Spy0602	c.654C>T	p.Thr218Thr	131						0
809	C	T	T	synonymous_variant	gacA	M5005_Spy0602	c.672C>T	p.Ala224Ala	12						0
832	C	T	T	missense_variant	gacA	M5005_Spy0602	c.695C>T	p.Ala232Val	10						0

834	G	A	A	missense_variant	gacA	M5005_Spy0602	c.697G>A	p.Val233Ile	3						0
842	G	C	C	synonymous_variant	gacA	M5005_Spy0602	c.705G>C	p.Val235Val	207						0
857	A	G	G	synonymous_variant	gacA	M5005_Spy0602	c.720A>G	p.Ser240Ser	4						0
866	C	T	T	synonymous_variant	gacA	M5005_Spy0602	c.729C>T	p.Phe243Phe	28						0
868	C	T	T	missense_variant	gacA	M5005_Spy0602	c.731C>T	p.Pro244Leu	25						0
870	G	A	A	missense_variant	gacA	M5005_Spy0602	c.733G>A	p.Ala245Thr	2						0
871	C	T	T	missense_variant	gacA	M5005_Spy0602	c.734C>T	p.Ala245Val	1						0
876	G	A	A	missense_variant	gacA	M5005_Spy0602	c.739G>A	p.Ala247Thr	1						0
878	C	T	T	synonymous_variant	gacA	M5005_Spy0602	c.741C>T	p.Ala247Ala	4						0
881	A	T	T	missense_variant	gacA	M5005_Spy0602	c.744A>T	p.Lys248Asn	15						0
905	T	A	A	missense_variant	gacA	M5005_Spy0602	c.768T>A	p.Asn256Lys	1						0
909	G	T	T	missense_variant	gacA	M5005_Spy0602	c.772G>T	p.Asp258Tyr	1						0
923	A	G	G	synonymous_variant	gacA	M5005_Spy0602	c.786A>G	p.Ala262Ala	39						0
926	A	G	G	synonymous_variant	gacA	M5005_Spy0602	c.789A>G	p.Thr263Thr	4						0
940	C	T	T	missense_variant	gacA	M5005_Spy0602	c.803C>T	p.Pro268Leu	2						0
948	C	A	A	missense_variant	gacA	M5005_Spy0602	c.811C>A	p.Gln271Lys	468						0
953	A	G	G	synonymous_variant	gacA	M5005_Spy0602	c.816A>G	p.Glu272Glu	34						0
959	G	A	A	synonymous_variant	gacA	M5005_Spy0602	c.822G>A	p.Leu274Leu	68						0
962	G	A	A	synonymous_variant	gacA	M5005_Spy0602	c.825G>A	p.Lys275Lys	1						0
963	G	T	T	missense_variant	gacA	M5005_Spy0602	c.826G>T	p.Ala276Ser	14						0
971	C	T	T	synonymous_variant	gacA	M5005_Spy0602	c.834C>T	p.Tyr278Tyr	1						0
989	G	A	A	synonymous_variant	gacA	M5005_Spy0602	c.852G>A	p.Lys284Lys	4						0
1002	A	G	G	upstream_gene_variant	intergenic				1						0
1013	T	C	C	upstream_gene_variant	intergenic				84						0
1016	C	T,A	A	upstream_gene_variant	intergenic				2	T	upstream_gene_variant	intergenic	c.-4653G>A		46
1017	T	G	G	upstream_gene_variant	intergenic				46						0
1018	A	G	G	upstream_gene_variant	intergenic				2						0
1020	G	A,T	A	upstream_gene_variant	intergenic				41	T	upstream_gene_variant	intergenic	c.-4657C>A		1
1021	G	C,A	A	upstream_gene_variant	intergenic				1	C	upstream_gene_variant	intergenic	c.-4658C>G		2
1022	C	T,G	G	upstream_gene_variant	intergenic				56	T	upstream_gene_variant	intergenic	c.-4659G>A		4
1023	C	T	T	upstream_gene_variant	intergenic				4						0
1025	A	C	C	upstream_gene_variant	intergenic				1						0
1037	G	A	A	upstream_gene_variant	intergenic				16						0
1039	G	A	A	upstream_gene_variant	intergenic				1						0
1044	A	G	G	upstream_gene_variant	intergenic				2						0
1054	A	G	G	upstream_gene_variant	intergenic				76						0
1069	G	A	A	upstream_gene_variant	intergenic				370						0
1076	A	G	G	upstream_gene_variant	intergenic				27						0
1078	C	T	T	upstream_gene_variant	intergenic				5						0
1080	C	T	T	upstream_gene_variant	intergenic				1						0
1082	G	A	A	upstream_gene_variant	intergenic				25						0
1087	T	C	C	upstream_gene_variant	intergenic				1						0
1094	G	A	A	upstream_gene_variant	intergenic				13						0
1097	T	C	C	upstream_gene_variant	intergenic				5						0
1106	A	G	G	upstream_gene_variant	intergenic				2						0
1117	G	A	A	missense_variant	gacB	M5005_Spy0603	c.7G>A	p.Asp3Asn	4						0
1118	A	C	C	missense_variant	gacB	M5005_Spy0603	c.8A>C	p.Asp3Ala	8						0
1144	T	G	G	missense_variant	gacB	M5005_Spy0603	c.34T>G	p.Leu12Val	1						0
1146	A	G	G	synonymous_variant	gacB	M5005_Spy0603	c.36A>G	p.Leu12Leu	34						0
1148	C	T	T	missense_variant	gacB	M5005_Spy0603	c.38C>T	p.Pro13Leu	2						0
1158	C	T	T	synonymous_variant	gacB	M5005_Spy0603	c.48C>T	p.Tyr16Tyr	15						0
1161	T	C	C	synonymous_variant	gacB	M5005_Spy0603	c.51T>C	p.Gly17Gly	13						0
1164	C	T	T	synonymous_variant	gacB	M5005_Spy0603	c.54C>T	p.Gly18Gly	2						0
1173	C	T	T	synonymous_variant	gacB	M5005_Spy0603	c.63C>T	p.Thr21Thr	386						0
1177	G	A	A	missense_variant	gacB	M5005_Spy0603	c.67G>A	p.Val23Ile	8						0
1179	C	T	T	synonymous_variant	gacB	M5005_Spy0603	c.69C>T	p.Val23Val	27						0
1186	T	C	C	synonymous_variant	gacB	M5005_Spy0603	c.76T>C	p.Leu26Leu	7						0
1191	T	C	C	synonymous_variant	gacB	M5005_Spy0603	c.81T>C	p.Ile27Ile	1						0
1204	T	C	C	missense_variant	gacB	M5005_Spy0603	c.94T>C	p.Ser32Pro	19						0
1217	G	A	A	missense_variant	gacB	M5005_Spy0603	c.107G>A	p.Arg36His	21						0
1224	T	C	C	synonymous_variant	gacB	M5005_Spy0603	c.114T>C	p.His38His	1593						0
1266	T	C	C	synonymous_variant	gacB	M5005_Spy0603	c.156T>C	p.Asp52Asp	9						0

1279	G	T	T	missense_variant	gacB	M5005_Spy0603	c.169G>T	p.Asp57Tyr	5						0
1281	T	C	C	synonymous_variant	gacB	M5005_Spy0603	c.171T>C	p.Asp57Asp	4						0
1284	C	T	T	synonymous_variant	gacB	M5005_Spy0603	c.174C>T	p.Cys58Cys	436						0
1293	T	C	C	synonymous_variant	gacB	M5005_Spy0603	c.183T>C	p.Leu61Leu	4						0
1299	C	T	T	synonymous_variant	gacB	M5005_Spy0603	c.189C>T	p.Pro63Pro	1						0
1300	C	T	T	missense_variant	gacB	M5005_Spy0603	c.190C>T	p.Pro64Ser	1						0
1301	C	A	A	missense_variant	gacB	M5005_Spy0603	c.191C>A	p.Pro64Gln	3						0
1303	A	C	C	missense_variant	gacB	M5005_Spy0603	c.193A>C	p.Lys65Gln	4						0
1311	T	A	A	synonymous_variant	gacB	M5005_Spy0603	c.201T>A	p.Gly67Gly	433						0
1312	C	T	T	missense_variant	gacB	M5005_Spy0603	c.202C>T	p.Pro68Ser	6						0
1327	G	A	A	missense_variant	gacB	M5005_Spy0603	c.217G>A	p.Ala73Thr	3						0
1328	C	T	T	missense_variant	gacB	M5005_Spy0603	c.218C>T	p.Ala73Val	2						0
1345	A	G	G	missense_variant	gacB	M5005_Spy0603	c.235A>G	p.Ile79Val	1						0
1350	C	T	T	synonymous_variant	gacB	M5005_Spy0603	c.240C>T	p.Thr80Thr	13						0
1354	G	A	A	missense_variant	gacB	M5005_Spy0603	c.244G>A	p.Ala82Thr	1						0
1356	C	T	T	synonymous_variant	gacB	M5005_Spy0603	c.246C>T	p.Ala82Ala	13						0
1359	G	A	A	synonymous_variant	gacB	M5005_Spy0603	c.249G>A	p.Leu83Leu	2						0
1367	G	A	A	missense_variant	gacB	M5005_Spy0603	c.257G>A	p.Ser86Asn	3						0
1373	A	G	G	missense_variant	gacB	M5005_Spy0603	c.263A>G	p.Gln88Arg	4						0
1375	C	A,T	A	missense_variant	gacB	M5005_Spy0603	c.265C>A	p.His89Asn	83	T	missense_variant	gacB	c.265C>T	p.His89Tyr	2
1376	A	G	G	missense_variant	gacB	M5005_Spy0603	c.266A>G	p.His89Arg	20						0
1388	A	C	C	missense_variant	gacB	M5005_Spy0603	c.278A>C	p.Asn93Thr	3						0
1389	C	T	T	synonymous_variant	gacB	M5005_Spy0603	c.279C>T	p.Asn93Asn	3						0
1404	G	A	A	synonymous_variant	gacB	M5005_Spy0603	c.294G>A	p.Val98Val	6						0
1407	G	A	A	synonymous_variant	gacB	M5005_Spy0603	c.297G>A	p.Leu99Leu	68						0
1410	C	T	T	synonymous_variant	gacB	M5005_Spy0603	c.300C>T	p.Gly100Gly	1						0
1422	C	A,T	A	synonymous_variant	gacB	M5005_Spy0603	c.312C>A	p.Gly104Gly	13	T	synonymous_variant	gacB	c.312C>T	p.Gly104Gly	44
1428	T	C	C	synonymous_variant	gacB	M5005_Spy0603	c.318T>C	p.Phe106Phe	8						0
1429	A	G	G	missense_variant	gacB	M5005_Spy0603	c.319A>G	p.Ile107Val	6						0
1437	A	G	G	synonymous_variant	gacB	M5005_Spy0603	c.327A>G	p.Pro109Pro	45						0
1443	G	A	A	synonymous_variant	gacB	M5005_Spy0603	c.333G>A	p.Val111Val	3						0
1447	C	A	A	missense_variant	gacB	M5005_Spy0603	c.337C>A	p.Gln113Lys	18						0
1450	A	G	G	missense_variant	gacB	M5005_Spy0603	c.340A>G	p.Ile114Val	14						0
1451	T	C	C	missense_variant	gacB	M5005_Spy0603	c.341T>C	p.Ile114Thr	4						0
1461	A	G	G	synonymous_variant	gacB	M5005_Spy0603	c.351A>G	p.Arg117Arg	5						0
1468	C	T	T	missense_variant	gacB	M5005_Spy0603	c.358C>T	p.Arg120Cys	2						0
1479	C	T	T	synonymous_variant	gacB	M5005_Spy0603	c.369C>T	p.Ile123Ile	2						0
1488	C	T	T	synonymous_variant	gacB	M5005_Spy0603	c.378C>T	p.Asp126Asp	3						0
1491	G	T	T	synonymous_variant	gacB	M5005_Spy0603	c.381G>T	p.Gly127Gly	11						0
1492	T	C	C	synonymous_variant	gacB	M5005_Spy0603	c.382T>C	p.Leu128Leu	45						0
1520	G	A	A	missense_variant	gacB	M5005_Spy0603	c.410G>A	p.Arg137Lys	1393						0
1531	G	A	A	missense_variant	gacB	M5005_Spy0603	c.421G>A	p.Ala141Thr	1						0
1537	C	T	T	synonymous_variant	gacB	M5005_Spy0603	c.427C>T	p.Leu143Leu	1						0
1539	G	A	A	synonymous_variant	gacB	M5005_Spy0603	c.429G>A	p.Leu143Leu	1						0
1548	C	T	T	synonymous_variant	gacB	M5005_Spy0603	c.438C>T	p.Ser146Ser	8						0
1566	A	G	G	synonymous_variant	gacB	M5005_Spy0603	c.456A>G	p.Arg152Arg	12						0
1572	T	A,C	A	synonymous_variant	gacB	M5005_Spy0603	c.462T>A	p.Ala154Ala	1	C	synonymous_variant	gacB	c.462T>C	p.Ala154Ala	1
1575	T	C	C	synonymous_variant	gacB	M5005_Spy0603	c.465T>C	p.Asp155Asp	224						0
1598	G	C	C	missense_variant	gacB	M5005_Spy0603	c.488G>C	p.Gly163Ala	2						0
1599	C	T	T	synonymous_variant	gacB	M5005_Spy0603	c.489C>T	p.Gly163Gly	3						0
1617	A	G	G	synonymous_variant	gacB	M5005_Spy0603	c.507A>G	p.Lys169Lys	6						0
1620	A	G	G	synonymous_variant	gacB	M5005_Spy0603	c.510A>G	p.Gln170Gln	1694						0
1623	T	C	C	synonymous_variant	gacB	M5005_Spy0603	c.513T>C	p.Val171Val	2						0
1636	A	C	C	missense_variant	gacB	M5005_Spy0603	c.526A>C	p.Lys176Gln	40						0
1643	G	A	A	missense_variant	gacB	M5005_Spy0603	c.533G>A	p.Cys178Tyr	105						0
1647	T	C	C	synonymous_variant	gacB	M5005_Spy0603	c.537T>C	p.Phe179Phe	97						0
1663	C	A	A	missense_variant	gacB	M5005_Spy0603	c.553C>A	p.Gln185Lys	1						0
1665	G	A	A	synonymous_variant	gacB	M5005_Spy0603	c.555G>A	p.Gln185Gln	2						0
1667	C	T	T	missense_variant	gacB	M5005_Spy0603	c.557C>T	p.Thr186Ile	1						0
1671	A	T	T	missense_variant	gacB	M5005_Spy0603	c.561A>T	p.Gln187His	2						0
1677	A	C	C	synonymous_variant	gacB	M5005_Spy0603	c.567A>C	p.Ser189Ser	1						0
1681	T	C	C	synonymous_variant	gacB	M5005_Spy0603	c.571T>C	p.Leu191Leu	232						0

1683	G	A	A	synonymous_variant	gacB	M5005_Spy0603	c.573G>A	p.Leu191Leu	18						0
1684	G	A	A	missense_variant	gacB	M5005_Spy0603	c.574G>A	p.Ala192Thr	1853						0
1685	C	T	T	missense_variant	gacB	M5005_Spy0603	c.575C>T	p.Ala192Val	4						0
1688	C	T	T	missense_variant	gacB	M5005_Spy0603	c.578C>T	p.Thr193Ile	1						0
1698	C	T	T	synonymous_variant	gacB	M5005_Spy0603	c.588C>T	p.Ser196Ser	1						0
1701	G	A	A	synonymous_variant	gacB	M5005_Spy0603	c.591G>A	p.Lys197Lys	14						0
1706	G	T	T	missense_variant	gacB	M5005_Spy0603	c.596G>T	p.Arg199Ile	26						0
1708	G	A	A	missense_variant	gacB	M5005_Spy0603	c.598G>A	p.Ala200Thr	41						0
1721	C	T	T	missense_variant	gacB	M5005_Spy0603	c.611C>T	p.Thr204Ile	1						0
1722	C	T	T	synonymous_variant	gacB	M5005_Spy0603	c.612C>T	p.Thr204Thr	39						0
1734	A	C	C	missense_variant	gacB	M5005_Spy0603	c.624A>C	p.Arg208Ser	753						0
1741	G	A	A	missense_variant	gacB	M5005_Spy0603	c.631G>A	p.Asp211Asn	3						0
1746	C	T	T	synonymous_variant	gacB	M5005_Spy0603	c.636C>T	p.Tyr212Tyr	5						0
1779	T	C	C	synonymous_variant	gacB	M5005_Spy0603	c.669T>C	p.Asn223Asn	1						0
1789	A	G	G	missense_variant	gacB	M5005_Spy0603	c.679A>G	p.Thr227Ala	43						0
1791	C	T	T	synonymous_variant	gacB	M5005_Spy0603	c.681C>T	p.Thr227Thr	1097						0
1792	G	A	A	missense_variant	gacB	M5005_Spy0603	c.682G>A	p.Ala228Thr	1						0
1793	C	T	T	missense_variant	gacB	M5005_Spy0603	c.683C>T	p.Ala228Val	4						0
1797	T	C	C	synonymous_variant	gacB	M5005_Spy0603	c.687T>C	p.Ile229Ile	4						0
1809	G	A	A	missense_variant	gacB	M5005_Spy0603	c.699G>A	p.Met233Ile	6						0
1810	G	T	T	missense_variant	gacB	M5005_Spy0603	c.700G>T	p.Ala234Ser	16						0
1817	C	T	T	missense_variant	gacB	M5005_Spy0603	c.707C>T	p.Ser236Leu	3						0
1823	A	G	G	missense_variant	gacB	M5005_Spy0603	c.713A>G	p.Lys238Arg	3						0
1824	G	A	A	synonymous_variant	gacB	M5005_Spy0603	c.714G>A	p.Lys238Lys	3						0
1826	G	A	A	missense_variant	gacB	M5005_Spy0603	c.716G>A	p.Arg239His	96						0
1842	T	C,A	A	synonymous_variant	gacB	M5005_Spy0603	c.732T>A	p.Ile244Ile	38	C	synonymous_variant	gacB	c.732T>C	p.Ile244Ile	3
1851	T	C	C	synonymous_variant	gacB	M5005_Spy0603	c.741T>C	p.His247His	114						0
1854	A	G	G	synonymous_variant	gacB	M5005_Spy0603	c.744A>G	p.Glu248Glu	49						0
1856	G	A	A	missense_variant	gacB	M5005_Spy0603	c.746G>A	p.Gly249Asp	140						0
1860	T	C	C	synonymous_variant	gacB	M5005_Spy0603	c.750T>C	p.Asn250Asn	1						0
1862	C	T	T	missense_variant	gacB	M5005_Spy0603	c.752C>T	p.Ala251Val	7						0
1884	T	G	G	synonymous_variant	gacB	M5005_Spy0603	c.774T>G	p.Ala258Ala	12						0
1889	C	T	T	missense_variant	gacB	M5005_Spy0603	c.779C>T	p.Thr260Ile	1						0
1895	G	A	A	missense_variant	gacB	M5005_Spy0603	c.785G>A	p.Cys262Tyr	17						0
1898	A	T	T	missense_variant	gacB	M5005_Spy0603	c.788A>T	p.Asp263Val	15						0
1905	C	T	T	synonymous_variant	gacB	M5005_Spy0603	c.795C>T	p.Asp265Asp	3						0
1906	C	T	T	missense_variant	gacB	M5005_Spy0603	c.796C>T	p.Pro266Ser	86						0
1928	C	T	T	missense_variant	gacB	M5005_Spy0603	c.818C>T	p.Thr273Ile	1						0
1938	C	T	T	synonymous_variant	gacB	M5005_Spy0603	c.828C>T	p.Asp276Asp	168						0
1945	C	T	T	synonymous_variant	gacB	M5005_Spy0603	c.835C>T	p.Leu279Leu	153						0
1968	G	A	A	synonymous_variant	gacB	M5005_Spy0603	c.858G>A	p.Gln286Gln	47						0
1971	T	C	C	synonymous_variant	gacB	M5005_Spy0603	c.861T>C	p.Ala287Ala	11						0
1977	C	T	T	synonymous_variant	gacB	M5005_Spy0603	c.867C>T	p.Ala289Ala	2						0
1986	T	C	C	synonymous_variant	gacB	M5005_Spy0603	c.876T>C	p.His292His	1						0
2020	C	T	T	synonymous_variant	gacB	M5005_Spy0603	c.910C>T	p.Leu304Leu	9						0
2054	T	C	C	missense_variant	gacB	M5005_Spy0603	c.944T>C	p.Val315Ala	4						0
2060	G	A	A	missense_variant	gacB	M5005_Spy0603	c.950G>A	p.Gly317Glu	27						0
2065	G	A	A	missense_variant	gacB	M5005_Spy0603	c.955G>A	p.Asp319Asn	1						0
2078	C	G	G	missense_variant	gacB	M5005_Spy0603	c.968C>G	p.Ser323Trp	8						0
2089	T	G	G	missense_variant	gacB	M5005_Spy0603	c.979T>G	p.Ser327Ala	1						0
2092	G	A	A	missense_variant	gacB	M5005_Spy0603	c.982G>A	p.Ala328Thr	5						0
2093	C	T	T	missense_variant	gacB	M5005_Spy0603	c.983C>T	p.Ala328Val	1						0
2100	T	G	G	synonymous_variant	gacB	M5005_Spy0603	c.990T>G	p.Leu330Leu	1685						0
2107	A	T,C	C	missense_variant	gacB	M5005_Spy0603	c.997A>C	p.Thr333Pro	496	T	missense_variant	gacB	c.997A>T	p.Thr333Ser	8
2115	A	G	G	synonymous_variant	gacB	M5005_Spy0603	c.1005A>G	p.Gln335Gln	1						0
2118	A	T	T	missense_variant	gacB	M5005_Spy0603	c.1008A>T	p.Lys336Asn	13						0
2128	G	A	A	missense_variant	gacB	M5005_Spy0603	c.1018G>A	p.Ala340Thr	3						0
2129	C	T	T	missense_variant	gacB	M5005_Spy0603	c.1019C>T	p.Ala340Val	39						0
2137	A	G	G	missense_variant	gacB	M5005_Spy0603	c.1027A>G	p.Ile343Val	1						0
2140	A	G	G	missense_variant	gacB	M5005_Spy0603	c.1030A>G	p.Asn344Asp	32						0
2142	C	T	T	synonymous_variant	gacB	M5005_Spy0603	c.1032C>T	p.Asn344Asn	8						0
2152	G	A	A	missense_variant	gacB	M5005_Spy0603	c.1042G>A	p.Ala348Thr	7						0

2156	G	T	T	missense_variant	gacB	M5005_Spy0603	c.1046G>T	p.Gly349Val	9						0
2167	G	T	T	missense_variant	gacB	M5005_Spy0603	c.1057G>T	p.Asp353Tyr	34						0
2171	A	G	G	missense_variant	gacB	M5005_Spy0603	c.1061A>G	p.His354Arg	1574						0
2192	C	T	T	missense_variant	gacB	M5005_Spy0603	c.1082C>T	p.Ala361Val	1						0
2201	A	G	G	missense_variant	gacB	M5005_Spy0603	c.1091A>G	p.Gln364Arg	2						0
2203	G	C	C	missense_variant	gacB	M5005_Spy0603	c.1093G>C	p.Glu365Gln	9						0
2211	C	T	T	synonymous_variant	gacB	M5005_Spy0603	c.1101C>T	p.Tyr367Tyr	34						0
2214	T	C	C	synonymous_variant	gacB	M5005_Spy0603	c.1104T>C	p.Thr368Thr	1						0
2218	G	A	A	missense_variant	gacB	M5005_Spy0603	c.1108G>A	p.Glu370Lys	13						0
2219	A	G	G	missense_variant	gacB	M5005_Spy0603	c.1109A>G	p.Glu370Gly	3						0
2226	T	C	C	synonymous_variant	gacB	M5005_Spy0603	c.1116T>C	p.Ile372Ile	13						0
2233	G	A	A	missense_variant	gacB	M5005_Spy0603	c.1123G>A	p.Glu375Lys	14						0
2238	C	T	T	synonymous_variant	gacB	M5005_Spy0603	c.1128C>T	p.Tyr376Tyr	4						0
2243	C	T	T	missense_variant	gacB	M5005_Spy0603	c.1133C>T	p.Ala378Val	4						0
2260	C	T	T	missense_variant	gacB_overlap	M5005_Spy0603	c.1150C>T	p.His384Tyr	18		synonymous_variant	gacC_overlap		p.Asn2Asn	0
2278	C	T	T	synonymous_variant	gacC	M5005_Spy0604	c.24C>T	p.Ser8Ser	1						0
2284	C	T	T	synonymous_variant	gacC	M5005_Spy0604	c.30C>T	p.Tyr10Tyr	7						0
2290	A	G	G	synonymous_variant	gacC	M5005_Spy0604	c.36A>G	p.Gly12Gly	89						0
2293	A	C	C	missense_variant	gacC	M5005_Spy0604	c.39A>C	p.Glu13Asp	4						0
2294	C	T	T	missense_variant	gacC	M5005_Spy0604	c.40C>T	p.Arg14Cys	1						0
2296	C	T	T	synonymous_variant	gacC	M5005_Spy0604	c.42C>T	p.Arg14Arg	10						0
2299	C	T	T	synonymous_variant	gacC	M5005_Spy0604	c.45C>T	p.Phe15Phe	18						0
2304	C	T	T	missense_variant	gacC	M5005_Spy0604	c.50C>T	p.Ala17Val	14						0
2312	A	G	G	missense_variant	gacC	M5005_Spy0604	c.58A>G	p.Ile20Val	1						0
2320	T	C	C	synonymous_variant	gacC	M5005_Spy0604	c.66T>C	p.Ser22Ser	1						0
2328	G	T	T	missense_variant	gacC	M5005_Spy0604	c.74G>T	p.Arg25Met	50						0
2329	G	A	A	synonymous_variant	gacC	M5005_Spy0604	c.75G>A	p.Arg25Arg	357						0
2332	A	G	G	synonymous_variant	gacC	M5005_Spy0604	c.78A>G	p.Gln26Gln	41						0
2335	A	G	G	synonymous_variant	gacC	M5005_Spy0604	c.81A>G	p.Thr27Thr	41						0
2341	C	A	A	missense_variant	gacC	M5005_Spy0604	c.87C>A	p.Asn29Lys	14						0
2342	G	A,C	A	missense_variant	gacC	M5005_Spy0604	c.88G>A	p.Asp30Asn	9	C	missense_variant	gacC	c.88G>C	p.Asp30His	1
2344	C	T	T	synonymous_variant	gacC	M5005_Spy0604	c.90C>T	p.Asp30Asp	22						0
2350	C	T	T	synonymous_variant	gacC	M5005_Spy0604	c.96C>T	p.Thr32Thr	1						0
2356	G	A	A	synonymous_variant	gacC	M5005_Spy0604	c.102G>A	p.Leu34Leu	4						0
2359	T	C	C	synonymous_variant	gacC	M5005_Spy0604	c.105T>C	p.Ile35Ile	2						0
2371	T	A,G	A	synonymous_variant	gacC	M5005_Spy0604	c.117T>A	p.Gly39Gly	1411	G	synonymous_variant	gacC	c.117T>G	p.Gly39Gly	2
2376	C	T	T	missense_variant	gacC	M5005_Spy0604	c.122C>T	p.Thr41Ile	1						0
2383	C	T	T	synonymous_variant	gacC	M5005_Spy0604	c.129C>T	p.Gly43Gly	3						0
2386	C	T	T	synonymous_variant	gacC	M5005_Spy0604	c.132C>T	p.Thr44Thr	1						0
2389	G	T	T	missense_variant	gacC	M5005_Spy0604	c.135G>T	p.Gln45His	1						0
2400	G	A	A	missense_variant	gacC	M5005_Spy0604	c.146G>A	p.Arg49His	1						0
2401	C	A	A	synonymous_variant	gacC	M5005_Spy0604	c.147C>A	p.Arg49Arg	6						0
2408	G	A	A	missense_variant	gacC	M5005_Spy0604	c.154G>A	p.Val52Ile	19						0
2410	C	T	T	synonymous_variant	gacC	M5005_Spy0604	c.156C>T	p.Val52Val	2						0
2435	A	G	G	missense_variant	gacC	M5005_Spy0604	c.181A>G	p.Ile61Val	3						0
2440	T	C	C	synonymous_variant	gacC	M5005_Spy0604	c.186T>C	p.Asn62Asn	1						0
2452	A	G	G	synonymous_variant	gacC	M5005_Spy0604	c.198A>G	p.Thr66Thr	1						0
2469	T	C	C	missense_variant	gacC	M5005_Spy0604	c.215T>C	p.Ile72Thr	1						0
2488	C	G	G	synonymous_variant	gacC	M5005_Spy0604	c.234C>G	p.Leu78Leu	25						0
2493	A	G	G	missense_variant	gacC	M5005_Spy0604	c.239A>G	p.Lys80Arg	39						0
2497	C	T	T	synonymous_variant	gacC	M5005_Spy0604	c.243C>T	p.His81His	11						0
2510	G	A	A	missense_variant	gacC	M5005_Spy0604	c.256G>A	p.Val86Ile	46						0
2527	C	T	T	synonymous_variant	gacC	M5005_Spy0604	c.273C>T	p.Asp91Asp	1						0
2533	T	C	C	synonymous_variant	gacC	M5005_Spy0604	c.279T>C	p.Asp93Asp	1						0
2537	A	G	G	missense_variant	gacC	M5005_Spy0604	c.283A>G	p.Ile95Val	167						0
2569	A	G	G	synonymous_variant	gacC	M5005_Spy0604	c.315A>G	p.Leu105Leu	1						0
2575	G	A	A	synonymous_variant	gacC	M5005_Spy0604	c.321G>A	p.Glu107Glu	16						0
2576	G	A	A	missense_variant	gacC	M5005_Spy0604	c.322G>A	p.Ala108Thr	28						0
2583	A	G	G	missense_variant	gacC	M5005_Spy0604	c.329A>G	p.Lys110Arg	1						0
2594	A	G	G	missense_variant	gacC	M5005_Spy0604	c.340A>G	p.Thr114Ala	45						0
2595	C	A	A	missense_variant	gacC	M5005_Spy0604	c.341C>A	p.Thr114Lys	128						0
2608	G	A	A	synonymous_variant	gacC	M5005_Spy0604	c.354G>A	p.Leu118Leu	42						0

2620	C	T	T	synonymous_variant	gacC	M5005_Spy0604	c.366C>T	p.Asp122Asp	9						0
2626	A	G	G	synonymous_variant	gacC	M5005_Spy0604	c.372A>G	p.Lys124Lys	2						0
2641	T	C	C	synonymous_variant	gacC	M5005_Spy0604	c.387T>C	p.His129His	3						0
2645	G	A	A	missense_variant	gacC	M5005_Spy0604	c.391G>A	p.Ala131Thr	1						0
2648	G	A	A	missense_variant	gacC	M5005_Spy0604	c.394G>A	p.Val132Ile	1886						0
2652	G	A	A	missense_variant	gacC	M5005_Spy0604	c.398G>A	p.Cys133Tyr	5						0
2654	C	T	T	missense_variant	gacC	M5005_Spy0604	c.400C>T	p.His134Tyr	9						0
2657	G	A	A	missense_variant	gacC	M5005_Spy0604	c.403G>A	p.Asp135Asn	519						0
2659	C	T	T	synonymous_variant	gacC	M5005_Spy0604	c.405C>T	p.Asp135Asp	16						0
2668	T	A	A	synonymous_variant	gacC	M5005_Spy0604	c.414T>A	p.Ile138Ile	4						0
2671	G	A	A	synonymous_variant	gacC	M5005_Spy0604	c.417G>A	p.Lys139Lys	2						0
2674	C	T	T	synonymous_variant	gacC	M5005_Spy0604	c.420C>T	p.Thr140Thr	28						0
2683	T	C	C	synonymous_variant	gacC	M5005_Spy0604	c.429T>C	p.Gly143Gly	21						0
2692	C	T	T	synonymous_variant	gacC	M5005_Spy0604	c.438C>T	p.Asn146Asn	2						0
2697	G	C	C	missense_variant	gacC	M5005_Spy0604	c.443G>C	p.Ser148Thr	1						0
2725	C	T	T	synonymous_variant	gacC	M5005_Spy0604	c.471C>T	p.Thr157Thr	2						0
2741	A	G	G	missense_variant	gacC	M5005_Spy0604	c.487A>G	p.Met163Val	1						0
2759	C	T	T	synonymous_variant	gacC	M5005_Spy0604	c.505C>T	p.Leu169Leu	2						0
2761	G	A	A	synonymous_variant	gacC	M5005_Spy0604	c.507G>A	p.Leu169Leu	5						0
2764	T	C	C	synonymous_variant	gacC	M5005_Spy0604	c.510T>C	p.Ala170Ala	2						0
2767	G	T	T	missense_variant	gacC	M5005_Spy0604	c.513G>T	p.Glu171Asp	2						0
2769	A	G	G	missense_variant	gacC	M5005_Spy0604	c.515A>G	p.Glu172Gly	4						0
2785	C	T	T	synonymous_variant	gacC	M5005_Spy0604	c.531C>T	p.Asp177Asp	1						0
2787	G	A	A	missense_variant	gacC	M5005_Spy0604	c.533G>A	p.Gly178Asp	8						0
2828	G	A	A	missense_variant	gacC	M5005_Spy0604	c.574G>A	p.Ala192Thr	1						0
2832	T	C	C	missense_variant	gacC	M5005_Spy0604	c.578T>C	p.Ile193Thr	1938						0
2833	T	C	C	synonymous_variant	gacC	M5005_Spy0604	c.579T>C	p.Ile193Ile	4						0
2839	A	G	G	synonymous_variant	gacC	M5005_Spy0604	c.585A>G	p.Lys195Lys	2						0
2858	C	T	T	missense_variant	gacC	M5005_Spy0604	c.604C>T	p.Pro202Ser	1						0
2860	A	G	G	synonymous_variant	gacC	M5005_Spy0604	c.606A>G	p.Pro202Pro	5						0
2872	C	T	T	synonymous_variant	gacC	M5005_Spy0604	c.618C>T	p.Tyr206Tyr	8						0
2875	T	C	C	synonymous_variant	gacC	M5005_Spy0604	c.621T>C	p.Arg207Arg	3						0
2878	G	T	T	missense_variant	gacC	M5005_Spy0604	c.624G>T	p.Gln208His	1						0
2885	G	A	A	missense_variant	gacC	M5005_Spy0604	c.631G>A	p.Ala211Thr	5						0
2896	G	A	A	synonymous_variant	gacC	M5005_Spy0604	c.642G>A	p.Leu214Leu	36						0
2923	G	A	A	missense_variant	gacC	M5005_Spy0604	c.669G>A	p.Met223Ile	1						0
2928	A	G	G	missense_variant	gacC	M5005_Spy0604	c.674A>G	p.Asn225Ser	3						0
2936	A	C	C	missense_variant	gacC	M5005_Spy0604	c.682A>C	p.Thr228Pro	37						0
2937	C	A,*	A	missense_variant	gacC	M5005_Spy0604	c.683C>A	p.Thr228Asn	537						1
2938	C	A	A	synonymous_variant	gacC	M5005_Spy0604	c.684C>A	p.Thr228Thr	1						0
2941	C	T	T	synonymous_variant	gacC	M5005_Spy0604	c.687C>T	p.Pro229Pro	3						0
2947	T	C	C	synonymous_variant	gacC	M5005_Spy0604	c.693T>C	p.His231His	11						0
2953	C	A	A	synonymous_variant	gacC	M5005_Spy0604	c.699C>A	p.Val233Val	1						0
2956	C	A,T	A	missense_variant	gacC	M5005_Spy0604	c.702C>A	p.Asn234Lys	1	T	synonymous_variant	gacC	c.702C>T	p.Asn234Asn	1
2968	G	T	T	missense_variant	gacC	M5005_Spy0604	c.714G>T	p.Trp238Cys	3						0
2980	A	G	G	synonymous_variant	gacC	M5005_Spy0604	c.726A>G	p.Ser242Ser	18						0
3010	T	C	C	synonymous_variant	gacC	M5005_Spy0604	c.756T>C	p.Asp252Asp	8						0
3020	A	G	G	missense_variant	gacC	M5005_Spy0604	c.766A>G	p.Lys256Glu	17						0
3023	C	G	G	missense_variant	gacC	M5005_Spy0604	c.769C>G	p.Pro257Ala	1						0
3024	C	T	T	missense_variant	gacC	M5005_Spy0604	c.770C>T	p.Pro257Leu	1						0
3031	C	T	T	synonymous_variant	gacC	M5005_Spy0604	c.777C>T	p.Asp259Asp	39						0
3032	C	T	T	missense_variant	gacC	M5005_Spy0604	c.778C>T	p.His260Tyr	1						0
3040	G	A	A	synonymous_variant	gacC	M5005_Spy0604	c.786G>A	p.Leu262Leu	2						0
3046	G	A	A	synonymous_variant	gacC	M5005_Spy0604	c.792G>A	p.Thr264Thr	16						0
3047	G	A	A	missense_variant	gacC	M5005_Spy0604	c.793G>A	p.Ala265Thr	1						0
3048	C	A	A	missense_variant	gacC	M5005_Spy0604	c.794C>A	p.Ala265Asp	4						0
3057	C	T	T	missense_variant	gacC	M5005_Spy0604	c.803C>T	p.Ser268Phe	9						0
3071	C	T	T	missense_variant	gacC	M5005_Spy0604	c.817C>T	p.Pro273Ser	9						0
3073	T	C	C	synonymous_variant	gacC	M5005_Spy0604	c.819T>C	p.Pro273Pro	5						0
3079	C	T	T	synonymous_variant	gacC	M5005_Spy0604	c.825C>T	p.Thr275Thr	28						0
3081	A	G	G	missense_variant	gacC	M5005_Spy0604	c.827A>G	p.Lys276Arg	8						0
3090	C	T	T	missense_variant	gacC	M5005_Spy0604	c.836C>T	p.Ala279Val	1						0

3096	T	C	C	missense_variant	gacC	M5005_Spy0604	c.842T>C	p.Leu281Ser	14						0
3103	G	A	A	synonymous_variant	gacC	M5005_Spy0604	c.849G>A	p.Arg283Arg	11						0
3107	G	A	A	missense_variant	gacC	M5005_Spy0604	c.853G>A	p.Gly285Ser	6						0
3114	G	A	A	missense_variant	gacC	M5005_Spy0604	c.860G>A	p.Arg287Lys	2						0
3115	A	G,C	C	missense_variant	gacC	M5005_Spy0604	c.861A>C	p.Arg287Ser	13	G	synonymous_variant	gacC	c.861A>G	p.Arg287Arg	1
3123	G	A	A	missense_variant	gacC	M5005_Spy0604	c.869G>A	p.Arg290His	4						0
3127	C	T	T	synonymous_variant	gacC	M5005_Spy0604	c.873C>T	p.Ile291Ile	29						0
3133	C	T	T	synonymous_variant	gacC	M5005_Spy0604	c.879C>T	p.His293His	64						0
3136	C	A	A	synonymous_variant	gacC	M5005_Spy0604	c.882C>A	p.Thr294Thr	3						0
3156	T	C	C	missense_variant	gacC	M5005_Spy0604	c.902T>C	p.Val301Ala	1						0
3164	T	C	C	synonymous_variant	gacC	M5005_Spy0604	c.910T>C	p.Leu304Leu	3						0
3170	G	T	T	missense_variant	gacC	M5005_Spy0604	c.916G>T	p.Gly306Cys	1						0
3187	A	G	G	splice_region_variant&stop_ret	gacC	M5005_Spy0604	c.933A>G	p.Ter311Ter	1						0
3217	A	G	G	missense_variant	gacD	M5005_Spy0605	c.28A>G	p.Ile10Val	16						0
3223	C	T	T	synonymous_variant	gacD	M5005_Spy0605	c.34C>T	p.Leu12Leu	1						0
3255	A	G	G	synonymous_variant	gacD	M5005_Spy0605	c.66A>G	p.Leu22Leu	63						0
3267	G	A	A	synonymous_variant	gacD	M5005_Spy0605	c.78G>A	p.Gly26Gly	354						0
3270	C	T	T	synonymous_variant	gacD	M5005_Spy0605	c.81C>T	p.Ser27Ser	9						0
3310	A	C	C	missense_variant	gacD	M5005_Spy0605	c.121A>C	p.Met41Leu	1						0
3315	T	C	C	synonymous_variant	gacD	M5005_Spy0605	c.126T>C	p.Phe42Phe	3						0
3318	G	A	A	synonymous_variant	gacD	M5005_Spy0605	c.129G>A	p.Thr43Thr	4						0
3330	G	A	A	synonymous_variant	gacD	M5005_Spy0605	c.141G>A	p.Leu47Leu	3						0
3333	A	G	G	synonymous_variant	gacD	M5005_Spy0605	c.144A>G	p.Val48Val	57						0
3337	A	C	C	missense_variant	gacD	M5005_Spy0605	c.148A>C	p.Ile50Leu	11						0
3351	C	T	T	synonymous_variant	gacD	M5005_Spy0605	c.162C>T	p.Arg54Arg	3						0
3352	C	T	T	synonymous_variant	gacD	M5005_Spy0605	c.163C>T	p.Leu55Leu	5						0
3354	G	A	A	synonymous_variant	gacD	M5005_Spy0605	c.165G>A	p.Leu55Leu	1						0
3359	G	T	T	missense_variant	gacD	M5005_Spy0605	c.170G>T	p.Gly57Val	9						0
3360	A	G	G	synonymous_variant	gacD	M5005_Spy0605	c.171A>G	p.Gly57Gly	1						0
3361	A	G	G	missense_variant	gacD	M5005_Spy0605	c.172A>G	p.Asn58Asp	182						0
3364	G	A	A	missense_variant	gacD	M5005_Spy0605	c.175G>A	p.Val59Ile	23						0
3367	C	T	T	missense_variant	gacD	M5005_Spy0605	c.178C>T	p.Pro60Ser	1						0
3378	A	G	G	synonymous_variant	gacD	M5005_Spy0605	c.189A>G	p.Pro63Pro	1						0
3384	G	A	A	synonymous_variant	gacD	M5005_Spy0605	c.195G>A	p.Ala65Ala	1						0
3392	T	C	C	missense_variant	gacD	M5005_Spy0605	c.203T>C	p.Leu68Ser	1						0
3393	G	A	A	synonymous_variant	gacD	M5005_Spy0605	c.204G>A	p.Leu68Leu	25						0
3409	T	C	C	missense_variant	gacD	M5005_Spy0605	c.220T>C	p.Ser74Pro	1						0
3425	C	T	T	missense_variant	gacD	M5005_Spy0605	c.236C>T	p.Ala79Val	1						0
3426	A	C	C	synonymous_variant	gacD	M5005_Spy0605	c.237A>C	p.Ala79Ala	2						0
3442	G	A	A	missense_variant	gacD	M5005_Spy0605	c.253G>A	p.Val85Ile	1						0
3452	T	C	C	missense_variant	gacD	M5005_Spy0605	c.263T>C	p.Val88Ala	3						0
3456	T	C	C	synonymous_variant	gacD	M5005_Spy0605	c.267T>C	p.Ser89Ser	2						0
3458	G	A	A	missense_variant	gacD	M5005_Spy0605	c.269G>A	p.Arg90Gln	1						0
3465	C	T	T	synonymous_variant	gacD	M5005_Spy0605	c.276C>T	p.Asp92Asp	73						0
3466	T	C	C	synonymous_variant	gacD	M5005_Spy0605	c.277T>C	p.Leu93Leu	39						0
3468	G	A	A	synonymous_variant	gacD	M5005_Spy0605	c.279G>A	p.Leu93Leu	5						0
3472	C	A	A	synonymous_variant	gacD	M5005_Spy0605	c.283C>A	p.Arg95Arg	1						0
3495	C	T	T	synonymous_variant	gacD	M5005_Spy0605	c.306C>T	p.His102His	6						0
3522	A	T	T	synonymous_variant	gacD	M5005_Spy0605	c.333A>T	p.Gly111Gly	1						0
3561	A	G	G	synonymous_variant	gacD	M5005_Spy0605	c.372A>G	p.Leu124Leu	14						0
3568	G	T	T	missense_variant	gacD	M5005_Spy0605	c.379G>T	p.Ala127Ser	1						0
3574	A	G	G	missense_variant	gacD	M5005_Spy0605	c.385A>G	p.Ile129Val	13						0
3594	A	G	G	synonymous_variant	gacD	M5005_Spy0605	c.405A>G	p.Ser135Ser	1						0
3597	G	A	A	synonymous_variant	gacD	M5005_Spy0605	c.408G>A	p.Gly136Gly	134						0
3599	A	G	G	missense_variant	gacD	M5005_Spy0605	c.410A>G	p.Tyr137Cys	32						0
3601	G	T,A	A	missense_variant	gacD	M5005_Spy0605	c.412G>A	p.Ala138Thr	41	T	missense_variant	gacD	c.412G>T	p.Ala138Ser	1
3603	T	C	C	synonymous_variant	gacD	M5005_Spy0605	c.414T>C	p.Ala138Ala	1						0
3605	A	C	C	missense_variant	gacD	M5005_Spy0605	c.416A>C	p.Tyr139Ser	62						0
3634	G	A	A	missense_variant	gacD	M5005_Spy0605	c.445G>A	p.Val149Ile	16						0
3637	G	A	A	missense_variant	gacD	M5005_Spy0605	c.448G>A	p.Val150Ile	24						0
3656	C	T	T	missense_variant	gacD	M5005_Spy0605	c.467C>T	p.Ala156Val	3						0
3714	A	G	G	synonymous_variant	gacD	M5005_Spy0605	c.525A>G	p.Val175Val	61						0

3715	C	T	T	synonymous_variant	gacD	M5005_Spy0605	c.526C>T	p.Leu176Leu	150						0
3730	A	T	T	missense_variant	gacD	M5005_Spy0605	c.541A>T	p.Met181Leu	18						0
3731	T	C	C	missense_variant	gacD	M5005_Spy0605	c.542T>C	p.Met181Thr	65						0
3774	T	C	C	synonymous_variant	gacD	M5005_Spy0605	c.585T>C	p.Asp195Asp	14						0
3777	C	T	T	synonymous_variant	gacD	M5005_Spy0605	c.588C>T	p.Ser196Ser	9						0
3778	C	T	T	missense_variant	gacD	M5005_Spy0605	c.589C>T	p.His197Tyr	1						0
3792	A	G,C	C	synonymous_variant	gacD	M5005_Spy0605	c.603A>C	p.Ala201Ala	5	G	synonymous_variant	gacD	c.603A>G	p.Ala201Ala	1
3795	G	A	A	synonymous_variant	gacD	M5005_Spy0605	c.606G>A	p.Lys202Lys	34						0
3858	G	A	A	synonymous_variant	gacD	M5005_Spy0605	c.669G>A	p.Arg223Arg	972						0
3870	G	A	A	synonymous_variant	gacD	M5005_Spy0605	c.681G>A	p.Thr227Thr	23						0
3879	G	A	A	synonymous_variant	gacD	M5005_Spy0605	c.690G>A	p.Gln230Gln	3						0
3882	G	A	A	missense_variant	gacD	M5005_Spy0605	c.693G>A	p.Met231Ile	8						0
3888	C	T	T	synonymous_variant	gacD	M5005_Spy0605	c.699C>T	p.Thr233Thr	215						0
3900	C	T	T	synonymous_variant	gacD	M5005_Spy0605	c.711C>T	p.Tyr237Tyr	3						0
3901	A	G	G	missense_variant	gacD	M5005_Spy0605	c.712A>G	p.Ile238Val	155						0
3918	A	G	G	synonymous_variant	gacD	M5005_Spy0605	c.729A>G	p.Leu243Leu	55						0
3920	T	C	C	missense_variant	gacD	M5005_Spy0605	c.731T>C	p.Val244Ala	31						0
3921	A	G	G	synonymous_variant	gacD	M5005_Spy0605	c.732A>G	p.Val244Val	1						0
3943	G	A	A	missense_variant	gacD	M5005_Spy0605	c.754G>A	p.Gly252Ser	17						0
3945	C	T	T	synonymous_variant	gacD	M5005_Spy0605	c.756C>T	p.Gly252Gly	2						0
3952	G	T	T	missense_variant	gacD	M5005_Spy0605	c.763G>T	p.Val255Phe	38						0
3954	C	T	T	synonymous_variant	gacD	M5005_Spy0605	c.765C>T	p.Val255Val	9						0
3958	A	G	G	missense_variant	gacD	M5005_Spy0605	c.769A>G	p.Lys257Glu	1						0
3959	A	G	G	missense_variant	gacD	M5005_Spy0605	c.770A>G	p.Lys257Arg	2						0
3962	A	G	G	missense_variant	gacD	M5005_Spy0605	c.773A>G	p.Lys258Arg	5						0
3966	T	C	C	synonymous_variant	gacD	M5005_Spy0605	c.777T>C	p.Asn259Asn	3						0
3990	T	C	C	synonymous_variant	gacD	M5005_Spy0605	c.801T>C	p.Ile267Ile	3						0
3996	A	G	G	missense_variant	gacE	M5005_Spy0606	c.4A>G	p.Ile2Val	1						0
3997	T	C	C	missense_variant	gacE	M5005_Spy0606	c.5T>C	p.Ile2Thr	41						0
4012	C	T	T	missense_variant	gacE	M5005_Spy0606	c.20C>T	p.Ala7Val	3						0
4022	A	C	C	synonymous_variant	gacE	M5005_Spy0606	c.30A>C	p.Val10Val	40						0
4025	G	T	T	missense_variant	gacE	M5005_Spy0606	c.33G>T	p.Gln11His	5						0
4026	C	T	T	missense_variant	gacE	M5005_Spy0606	c.34C>T	p.His12Tyr	1						0
4039	C	T	T	missense_variant	gacE	M5005_Spy0606	c.47C>T	p.Thr16Met	1						0
4040	G	A	A	synonymous_variant	gacE	M5005_Spy0606	c.48G>A	p.Thr16Thr	1603						0
4049	G	T	T	synonymous_variant	gacE	M5005_Spy0606	c.57G>T	p.Leu19Leu	1						0
4060	C	T	T	missense_variant	gacE	M5005_Spy0606	c.68C>T	p.Ala23Val	18						0
4076	C	T	T	synonymous_variant	gacE	M5005_Spy0606	c.84C>T	p.Arg28Arg	11						0
4078	C	T	T	missense_variant	gacE	M5005_Spy0606	c.86C>T	p.Thr29Met	1						0
4092	C	T	T	missense_variant	gacE	M5005_Spy0606	c.100C>T	p.Arg34Cys	1						0
4094	C	T	T	synonymous_variant	gacE	M5005_Spy0606	c.102C>T	p.Arg34Arg	12						0
4097	A	G	G	synonymous_variant	gacE	M5005_Spy0606	c.105A>G	p.Leu35Leu	45						0
4106	T	C	C	synonymous_variant	gacE	M5005_Spy0606	c.114T>C	p.Ile38Ile	1						0
4112	A	C	C	synonymous_variant	gacE	M5005_Spy0606	c.120A>C	p.Gly40Gly	1						0
4131	C	T	T	synonymous_variant	gacE	M5005_Spy0606	c.139C>T	p.Leu47Leu	9						0
4156	A	G	G	missense_variant	gacE	M5005_Spy0606	c.164A>G	p.Tyr55Cys	1						0
4160	A	G	G	synonymous_variant	gacE	M5005_Spy0606	c.168A>G	p.Lys56Lys	19						0
4163	C	T	T	synonymous_variant	gacE	M5005_Spy0606	c.171C>T	p.Gly57Gly	26						0
4175	A	G	G	synonymous_variant	gacE	M5005_Spy0606	c.183A>G	p.Gly61Gly	495						0
4176	A	G	G	missense_variant	gacE	M5005_Spy0606	c.184A>G	p.Ile62Val	1						0
4190	C	T	T	synonymous_variant	gacE	M5005_Spy0606	c.198C>T	p.Asn66Asn	77						0
4196	A	G	G	synonymous_variant	gacE	M5005_Spy0606	c.204A>G	p.Ser68Ser	1481						0
4199	A	G	G	synonymous_variant	gacE	M5005_Spy0606	c.207A>G	p.Gly69Gly	2						0
4202	A	T	T	missense_variant	gacE	M5005_Spy0606	c.210A>T	p.Lys70Asn	1						0
4203	T	A,C	A	missense_variant	gacE	M5005_Spy0606	c.211T>A	p.Ser71Thr	1	C	missense_variant	gacE	c.211T>C	p.Ser71Pro	1
4204	C	A	A	stop_gained	gacE	M5005_Spy0606	c.212C>A	p.Ser71*	1						0
4205	A	G	G	synonymous_variant	gacE	M5005_Spy0606	c.213A>G	p.Ser71Ser	4						0
4207	C	A	A	missense_variant	gacE	M5005_Spy0606	c.215C>A	p.Thr72Lys	2						0
4208	G	A	A	synonymous_variant	gacE	M5005_Spy0606	c.216G>A	p.Thr72Thr	39						0
4210	T	A	A	missense_variant	gacE	M5005_Spy0606	c.218T>A	p.Leu73His	1						0
4226	C	T	T	synonymous_variant	gacE	M5005_Spy0606	c.234C>T	p.Ser78Ser	57						0
4228	A	C	C	missense_variant	gacE	M5005_Spy0606	c.236A>C	p.Gln79Pro	1						0

4229	G	A	A	synonymous_variant	gacE	M5005_Spy0606	c.237G>A	p.Gln79Gln	11						0
4236	G	A	A	missense_variant	gacE	M5005_Spy0606	c.244G>A	p.Val82Ile	114						0
4250	G	A,T	A	synonymous_variant	gacE	M5005_Spy0606	c.258G>A	p.Gly86Gly	945	T	synonymous_variant	gacE	c.258G>T	p.Gly86Gly	2
4258	C	T	T	missense_variant	gacE	M5005_Spy0606	c.266C>T	p.Thr89Ile	19						0
4268	G	A	A	synonymous_variant	gacE	M5005_Spy0606	c.276G>A	p.Gly92Gly	93						0
4274	G	A	A	missense_variant	gacE	M5005_Spy0606	c.282G>A	p.Met94Ile	1						0
4275	G	A	A	missense_variant	gacE	M5005_Spy0606	c.283G>A	p.Val95Ile	1						0
4276	T	A	A	missense_variant	gacE	M5005_Spy0606	c.284T>A	p.Val95Asp	1						0
4277	T	C	C	synonymous_variant	gacE	M5005_Spy0606	c.285T>C	p.Val95Val	1						0
4298	G	A	A	synonymous_variant	gacE	M5005_Spy0606	c.306G>A	p.Val102Val	1						0
4331	C	T	T	synonymous_variant	gacE	M5005_Spy0606	c.339C>T	p.Asn113Asn	31						0
4355	G	A	A	synonymous_variant	gacE	M5005_Spy0606	c.363G>A	p.Leu121Leu	37						0
4369	A	G	G	missense_variant	gacE	M5005_Spy0606	c.377A>G	p.Asp126Gly	1						0
4370	C	T	T	synonymous_variant	gacE	M5005_Spy0606	c.378C>T	p.Asp126Asp	13						0
4381	A	C	C	missense_variant	gacE	M5005_Spy0606	c.389A>C	p.Asp130Ala	3						0
4388	C	T	T	synonymous_variant	gacE	M5005_Spy0606	c.396C>T	p.Tyr132Tyr	18						0
4415	G	A	A	synonymous_variant	gacE	M5005_Spy0606	c.423G>A	p.Leu141Leu	1						0
4416	C	A	A	missense_variant	gacE	M5005_Spy0606	c.424C>A	p.His142Asn	3						0
4418	T	C	C	synonymous_variant	gacE	M5005_Spy0606	c.426T>C	p.His142His	2						0
4457	C	T,G	G	synonymous_variant	gacE	M5005_Spy0606	c.465C>G	p.Gly155Gly	15	T	synonymous_variant	gacE	c.465C>T	p.Gly155Gly	2
4490	T	C	C	synonymous_variant	gacE	M5005_Spy0606	c.498T>C	p.Ile166Ile	9						0
4495	C	T	T	missense_variant	gacE	M5005_Spy0606	c.503C>T	p.Ala168Val	4						0
4496	C	T	T	synonymous_variant	gacE	M5005_Spy0606	c.504C>T	p.Ala168Ala	4						0
4499	A	G	G	synonymous_variant	gacE	M5005_Spy0606	c.507A>G	p.Gln169Gln	1						0
4505	C	T	T	synonymous_variant	gacE	M5005_Spy0606	c.513C>T	p.Asp171Asp	6						0
4526	G	A	A	synonymous_variant	gacE	M5005_Spy0606	c.534G>A	p.Val178Val	12						0
4571	T	C	C	synonymous_variant	gacE	M5005_Spy0606	c.579T>C	p.Tyr193Tyr	1						0
4576	T	C	C	missense_variant	gacE	M5005_Spy0606	c.584T>C	p.Met195Thr	2						0
4579	A	G	G	missense_variant	gacE	M5005_Spy0606	c.587A>G	p.Glu196Gly	4						0
4587	G	T	T	missense_variant	gacE	M5005_Spy0606	c.595G>T	p.Asp199Tyr	3						0
4643	T	C	C	synonymous_variant	gacE	M5005_Spy0606	c.651T>C	p.Tyr217Tyr	15						0
4655	A	G	G	synonymous_variant	gacE	M5005_Spy0606	c.663A>G	p.Ala221Ala	45						0
4659	C	T	T	missense_variant	gacE	M5005_Spy0606	c.667C>T	p.Leu223Phe	1						0
4673	T	C	C	synonymous_variant	gacE	M5005_Spy0606	c.681T>C	p.Gly227Gly	3						0
4692	G	A	A	missense_variant	gacE	M5005_Spy0606	c.700G>A	p.Glu234Lys	1						0
4700	T	G	G	missense_variant	gacE	M5005_Spy0606	c.708T>G	p.Phe236Leu	1						0
4702	A	G	G	missense_variant	gacE	M5005_Spy0606	c.710A>G	p.Asp237Gly	2						0
4703	T	C	C	synonymous_variant	gacE	M5005_Spy0606	c.711T>C	p.Asp237Asp	31						0
4721	T	C	C	synonymous_variant	gacE	M5005_Spy0606	c.729T>C	p.Ser243Ser	7						0
4722	G	A	A	missense_variant	gacE	M5005_Spy0606	c.730G>A	p.Val244Ile	1						0
4741	C	T	T	missense_variant	gacE	M5005_Spy0606	c.749C>T	p.Ala250Val	2						0
4746	G	C	C	missense_variant	gacE	M5005_Spy0606	c.754G>C	p.Asp252His	1						0
4749	G	A	A	missense_variant	gacE	M5005_Spy0606	c.757G>A	p.Ala253Thr	1						0
4753	T	C	C	missense_variant	gacE	M5005_Spy0606	c.761T>C	p.Met254Thr	6						0
4758	G	A	A	missense_variant	gacE	M5005_Spy0606	c.766G>A	p.Ala256Thr	62						0
4771	C	T	T	missense_variant	gacE	M5005_Spy0606	c.779C>T	p.Ser260Phe	2						0
4786	C	T	T	missense_variant	gacE	M5005_Spy0606	c.794C>T	p.Ala265Val	1						0
4802	G	A	A	synonymous_variant	gacE	M5005_Spy0606	c.810G>A	p.Val270Val	49						0
4811	C	T	T	synonymous_variant	gacE	M5005_Spy0606	c.819C>T	p.Ile273Ile	8						0
4817	T	C	C	synonymous_variant	gacE	M5005_Spy0606	c.825T>C	p.Asn275Asn	5						0
4818	C	T	T	missense_variant	gacE	M5005_Spy0606	c.826C>T	p.Pro276Ser	2						0
4819	C	T	T	missense_variant	gacE	M5005_Spy0606	c.827C>T	p.Pro276Leu	2						0
4828	C	T	T	missense_variant	gacE	M5005_Spy0606	c.836C>T	p.Thr279Ile	10						0
4855	T	C	C	missense_variant	gacE	M5005_Spy0606	c.863T>C	p.Val288Ala	1						0
4862	T	C	C	synonymous_variant	gacE	M5005_Spy0606	c.870T>C	p.Tyr290Tyr	1						0
4865	A	G	G	synonymous_variant	gacE	M5005_Spy0606	c.873A>G	p.Glu291Glu	7						0
4869	T	C	C	synonymous_variant	gacE	M5005_Spy0606	c.877T>C	p.Leu293Leu	11						0
4878	G	A	A	missense_variant	gacE	M5005_Spy0606	c.886G>A	p.Asp296Asn	24						0
4886	C	T	T	synonymous_variant	gacE	M5005_Spy0606	c.894C>T	p.Thr298Thr	4						0
4893	G	A	A	missense_variant	gacE	M5005_Spy0606	c.901G>A	p.Ala301Thr	1						0
4895	C	T,A	A	synonymous_variant	gacE	M5005_Spy0606	c.903C>A	p.Ala301Ala	13	T	synonymous_variant	gacE	c.903C>T	p.Ala301Ala	1
4925	T	C	C	synonymous_variant	gacE	M5005_Spy0606	c.933T>C	p.Ile311Ile	1						0

4934	C	T	T	synonymous_variant	gacE	M5005_Spy0606	c.942C>T	p.Tyr314Tyr	6						0
4937	C	T	T	synonymous_variant	gacE	M5005_Spy0606	c.945C>T	p.Asn315Asn	1						0
4940	C	T	T	synonymous_variant	gacE	M5005_Spy0606	c.948C>T	p.Asp316Asp	4						0
4967	T	C	C	synonymous_variant	gacE	M5005_Spy0606	c.975T>C	p.Gly325Gly	1						0
4973	T	G	G	synonymous_variant	gacE	M5005_Spy0606	c.981T>G	p.Gly327Gly	762						0
4981	G	A	A	missense_variant	gacE	M5005_Spy0606	c.989G>A	p.Cys330Tyr	1						0
4987	C	T	T	missense_variant	gacE	M5005_Spy0606	c.995C>T	p.Ser332Phe	1						0
4988	C	T	T	synonymous_variant	gacE	M5005_Spy0606	c.996C>T	p.Ser332Ser	263						0
4991	C	T	T	synonymous_variant	gacE	M5005_Spy0606	c.999C>T	p.Tyr333Tyr	1						0
5000	A	G	G	synonymous_variant	gacE	M5005_Spy0606	c.1008A>G	p.Gln336Gln	1						0
5023	A	C	C	missense_variant	gacE	M5005_Spy0606	c.1031A>C	p.Lys344Thr	1						0
5025	C	T	T	synonymous_variant	gacE	M5005_Spy0606	c.1033C>T	p.Leu345Leu	12						0
5045	C	T	T	synonymous_variant	gacE	M5005_Spy0606	c.1053C>T	p.Val351Val	7						0
5057	C	T	T	synonymous_variant	gacE	M5005_Spy0606	c.1065C>T	p.Asn355Asn	2						0
5067	C	T	T	synonymous_variant	gacE	M5005_Spy0606	c.1075C>T	p.Leu359Leu	2						0
5072	C	A	A	synonymous_variant	gacE	M5005_Spy0606	c.1080C>A	p.Leu360Leu	3						0
5077	C	T	T	missense_variant	gacE	M5005_Spy0606	c.1085C>T	p.Ser362Leu	3						0
5080	C	A	A	missense_variant	gacE	M5005_Spy0606	c.1088C>A	p.Thr363Lys	4						0
5086	A	G	G	missense_variant	gacE	M5005_Spy0606	c.1094A>G	p.Glu365Gly	1						0
5094	C	T	T	missense_variant	gacE	M5005_Spy0606	c.1102C>T	p.Pro368Ser	2						0
5116	A	G	G	missense_variant	gacE	M5005_Spy0606	c.1124A>G	p.Asn375Ser	38						0
5122	T	C	C	missense_variant	gacE	M5005_Spy0606	c.1130T>C	p.Ile377Thr	2						0
5133	G	A	A	missense_variant	gacE	M5005_Spy0606	c.1141G>A	p.Asp381Asn	12						0
5138	G	A	A	synonymous_variant	gacE	M5005_Spy0606	c.1146G>A	p.Leu382Leu	43						0
5139	T	C	C	missense_variant	gacE	M5005_Spy0606	c.1147T>C	p.Ser383Pro	1						0
5153	A	G	G	synonymous_variant	gacE	M5005_Spy0606	c.1161A>G	p.Ser387Ser	8						0
5155	C	T	T	missense_variant	gacE	M5005_Spy0606	c.1163C>T	p.Ala388Val	1						0
5162	C	T	T	synonymous_variant	gacE	M5005_Spy0606	c.1170C>T	p.Gly390Gly	220						0
5166	T	G	G	missense_variant	gacE	M5005_Spy0606	c.1174T>G	p.Tyr392Asp	1						0
5172	C	A	A	missense_variant	gacE	M5005_Spy0606	c.1180C>A	p.Arg394Ser	1						0
5187	G	T	T	missense_variant	gacE	M5005_Spy0606	c.1195G>T	p.Val399Phe	65						0
5188	T	A	A	missense_variant	gacE	M5005_Spy0606	c.1196T>A	p.Val399Asp	1						0
5193	C	T	T	missense_variant	gacE	M5005_Spy0606	c.1201C>T	p.His401Tyr	5						0
5202	G	C	C	upstream_gene_variant	intergenic				2						0
5215	G	A	A	upstream_gene_variant	intergenic				1						0
5229	A	C	C	missense_variant	gacF	M5005_Spy0607	c.7A>C	p.Lys3Gln	2						0
5243	T	C	C	synonymous_variant	gacF	M5005_Spy0607	c.21T>C	p.Ile7Ile	6						0
5252	T	C	C	synonymous_variant	gacF	M5005_Spy0607	c.30T>C	p.Asn10Asn	10						0
5265	C	A	A	missense_variant	gacF	M5005_Spy0607	c.43C>A	p.Pro15Thr	18						0
5279	T	G	G	missense_variant	gacF	M5005_Spy0607	c.57T>G	p.Asp19Glu	1						0
5285	T	C	C	synonymous_variant	gacF	M5005_Spy0607	c.63T>C	p.Leu21Leu	2						0
5288	C	T	T	synonymous_variant	gacF	M5005_Spy0607	c.66C>T	p.Asp22Asp	4						0
5292	T	C	C	missense_variant	gacF	M5005_Spy0607	c.70T>C	p.Phe24Leu	19						0
5297	A	T	T	missense_variant	gacF	M5005_Spy0607	c.75A>T	p.Leu25Phe	31						0
5318	G	A	A	synonymous_variant	gacF	M5005_Spy0607	c.96G>A	p.Glu32Glu	131						0
5355	G	A	A	missense_variant	gacF	M5005_Spy0607	c.133G>A	p.Asp45Asn	56						0
5356	A	G	G	missense_variant	gacF	M5005_Spy0607	c.134A>G	p.Asp45Gly	3						0
5357	C	T	T	synonymous_variant	gacF	M5005_Spy0607	c.135C>T	p.Asp45Asp	55						0
5362	G	T	T	missense_variant	gacF	M5005_Spy0607	c.140G>T	p.Arg47Leu	5						0
5377	G	C	C	missense_variant	gacF	M5005_Spy0607	c.155G>C	p.Ser52Thr	4						0
5393	T	C	C	synonymous_variant	gacF	M5005_Spy0607	c.171T>C	p.Ser57Ser	28						0
5425	C	T	T	missense_variant	gacF	M5005_Spy0607	c.203C>T	p.Thr68Ile	43						0
5459	A	G	G	synonymous_variant	gacF	M5005_Spy0607	c.237A>G	p.Ala79Ala	2						0
5486	T	C	C	synonymous_variant	gacF	M5005_Spy0607	c.264T>C	p.Ile88Ile	1						0
5498	T	C	C	synonymous_variant	gacF	M5005_Spy0607	c.276T>C	p.Asp92Asp	17						0
5515	C	T	T	missense_variant	gacF	M5005_Spy0607	c.293C>T	p.Thr98Met	12						0
5516	G	A	A	synonymous_variant	gacF	M5005_Spy0607	c.294G>A	p.Thr98Thr	39						0
5537	A	G	G	synonymous_variant	gacF	M5005_Spy0607	c.315A>G	p.Lys105Lys	7						0
5562	A	C	C	missense_variant	gacF	M5005_Spy0607	c.340A>C	p.Lys114Gln	18						0
5563	A	C	C	missense_variant	gacF	M5005_Spy0607	c.341A>C	p.Lys114Thr	6						0
5566	G	A	A	missense_variant	gacF	M5005_Spy0607	c.344G>A	p.Arg115Gln	10						0
5597	T	C	C	synonymous_variant	gacF	M5005_Spy0607	c.375T>C	p.Phe125Phe	1						0

5598	G	T	T	missense_variant	gacF	M5005_Spy0607	c.376G>T	p.Val126Leu	118						0
5602	A	C	C	missense_variant	gacF	M5005_Spy0607	c.380A>C	p.Asn127Thr	30						0
5615	C	T	T	synonymous_variant	gacF	M5005_Spy0607	c.393C>T	p.Asp131Asp	30						0
5619	T	C	C	synonymous_variant	gacF	M5005_Spy0607	c.397T>C	p.Leu133Leu	21						0
5625	G	A	A	missense_variant	gacF	M5005_Spy0607	c.403G>A	p.Ala135Thr	4						0
5636	T	C	C	synonymous_variant	gacF	M5005_Spy0607	c.414T>C	p.Phe138Phe	1						0
5641	C	T	T	missense_variant	gacF	M5005_Spy0607	c.419C>T	p.Ser140Phe	5						0
5648	T	C	C	synonymous_variant	gacF	M5005_Spy0607	c.426T>C	p.His142His	25						0
5652	C	T	T	missense_variant	gacF	M5005_Spy0607	c.430C>T	p.Pro144Ser	1						0
5661	G	A	A	missense_variant	gacF	M5005_Spy0607	c.439G>A	p.Asp147Asn	1						0
5663	T	C	C	synonymous_variant	gacF	M5005_Spy0607	c.441T>C	p.Asp147Asp	1						0
5705	A	T	T	synonymous_variant	gacF	M5005_Spy0607	c.483A>T	p.Ala161Ala	1						0
5715	T	C	C	synonymous_variant	gacF	M5005_Spy0607	c.493T>C	p.Leu165Leu	578						0
5723	T	C	C	synonymous_variant	gacF	M5005_Spy0607	c.501T>C	p.Asp167Asp	1						0
5730	T	C	C	synonymous_variant	gacF	M5005_Spy0607	c.508T>C	p.Leu170Leu	22						0
5733	A	G	G	missense_variant	gacF	M5005_Spy0607	c.511A>G	p.Met171Val	67						0
5757	A	G	G	missense_variant	gacF	M5005_Spy0607	c.535A>G	p.Asn179Asp	1						0
5763	G	A	A	missense_variant	gacF	M5005_Spy0607	c.541G>A	p.Asp181Asn	9						0
5770	C	T	T	missense_variant	gacF	M5005_Spy0607	c.548C>T	p.Pro183Leu	1						0
5774	C	T	T	synonymous_variant	gacF	M5005_Spy0607	c.552C>T	p.Asp184Asp	12						0
5786	T	C	C	synonymous_variant	gacF	M5005_Spy0607	c.564T>C	p.Asp188Asp	306						0
5808	C	T	T	synonymous_variant	gacF	M5005_Spy0607	c.586C>T	p.Leu196Leu	506						0
5822	C	T	T	synonymous_variant	gacF	M5005_Spy0607	c.600C>T	p.Thr200Thr	2						0
5837	A	C	C	synonymous_variant	gacF	M5005_Spy0607	c.615A>C	p.Ser205Ser	62						0
5844	A	G	G	missense_variant	gacF	M5005_Spy0607	c.622A>G	p.Ile208Val	1068						0
5852	C	T	T	synonymous_variant	gacF	M5005_Spy0607	c.630C>T	p.Arg210Arg	5						0
5856	A	G	G	missense_variant	gacF	M5005_Spy0607	c.634A>G	p.Thr212Ala	1						0
5858	A	G	G	synonymous_variant	gacF	M5005_Spy0607	c.636A>G	p.Thr212Thr	2						0
5876	A	G	G	synonymous_variant	gacF	M5005_Spy0607	c.654A>G	p.Arg218Arg	21						0
5881	C	T	T	missense_variant	gacF	M5005_Spy0607	c.659C>T	p.Thr220Ile	4						0
5884	A	G	G	missense_variant	gacF	M5005_Spy0607	c.662A>G	p.Asp221Gly	9						0
5891	A	C	C	synonymous_variant	gacF	M5005_Spy0607	c.669A>C	p.Pro223Pro	9						0
5901	T	C	C	missense_variant	gacF	M5005_Spy0607	c.679T>C	p.Tyr227His	1891						0
5914	A	G	G	missense_variant	gacF	M5005_Spy0607	c.692A>G	p.Lys231Arg	47						0
5916	C	T	T	synonymous_variant	gacF	M5005_Spy0607	c.694C>T	p.Leu232Leu	142						0
5933	A	T	T	missense_variant	gacF	M5005_Spy0607	c.711A>T	p.Leu237Phe	1						0
5939	C	T	T	synonymous_variant	gacF	M5005_Spy0607	c.717C>T	p.Tyr239Tyr	12						0
5961	G	A	A	missense_variant	gacF	M5005_Spy0607	c.739G>A	p.Asp247Asn	17						0
5973	G	A	A	missense_variant	gacF	M5005_Spy0607	c.751G>A	p.Val251Met	136						0
5979	G	A	A	missense_variant	gacF	M5005_Spy0607	c.757G>A	p.Glu253Lys	801						0
5981	A	G	G	synonymous_variant	gacF	M5005_Spy0607	c.759A>G	p.Glu253Glu	6						0
5982	C	T	T	synonymous_variant	gacF	M5005_Spy0607	c.760C>T	p.Leu254Leu	22						0
5987	A	G	G	synonymous_variant	gacF	M5005_Spy0607	c.765A>G	p.Leu255Leu	104						0
6012	A	G	G	missense_variant	gacF	M5005_Spy0607	c.790A>G	p.Ile264Val	1						0
6058	C	T	T	missense_variant	gacF	M5005_Spy0607	c.836C>T	p.Thr279Ile	4						0
6060	A	G	G	missense_variant	gacF	M5005_Spy0607	c.838A>G	p.Ile280Val	18						0
6062	C	T	T	synonymous_variant	gacF	M5005_Spy0607	c.840C>T	p.Ile280Ile	11						0
6068	T	C	C	synonymous_variant	gacF	M5005_Spy0607	c.846T>C	p.Asp282Asp	21						0
6073	C	T	T	missense_variant	gacF	M5005_Spy0607	c.851C>T	p.Thr284Ile	2						0
6079	A	G	G	missense_variant	gacF	M5005_Spy0607	c.857A>G	p.Gln286Arg	4						0
6088	C	T	T	missense_variant	gacF	M5005_Spy0607	c.866C>T	p.Thr289Ile	2						0
6097	T	C	C	missense_variant	gacF	M5005_Spy0607	c.875T>C	p.Val292Ala	2						0
6098	G	A,T	A	synonymous_variant	gacF	M5005_Spy0607	c.876G>A	p.Val292Val	51	T	synonymous_variant	gacF	c.876G>T	p.Val292Val	22
6104	G	A	A	synonymous_variant	gacF	M5005_Spy0607	c.882G>A	p.Leu294Leu	40						0
6155	A	G	G	synonymous_variant	gacF	M5005_Spy0607	c.933A>G	p.Gln311Gln	40						0
6157	A	T	T	missense_variant	gacF	M5005_Spy0607	c.935A>T	p.Tyr312Phe	1						0
6158	C	T	T	synonymous_variant	gacF	M5005_Spy0607	c.936C>T	p.Tyr312Tyr	33						0
6162	G	A	A	missense_variant	gacF	M5005_Spy0607	c.940G>A	p.Ala314Thr	1						0
6190	A	G	G	missense_variant	gacF	M5005_Spy0607	c.968A>G	p.Tyr323Cys	20						0
6191	C	T	T	synonymous_variant	gacF	M5005_Spy0607	c.969C>T	p.Tyr323Tyr	1						0
6198	A	C	C	missense_variant	gacF	M5005_Spy0607	c.976A>C	p.Lys326Gln	2						0
6254	T	C	C	missense_variant	gacG	M5005_Spy0608	c.28T>C	p.Phe10Leu	1						0

6278	G	A	A	missense_variant	gacG	M5005_Spy0608	c.52G>A	p.Ala18Thr	5						0
6291	A	G	G	missense_variant	gacG	M5005_Spy0608	c.65A>G	p.Tyr22Cys	3						0
6298	G	A	A	synonymous_variant	gacG	M5005_Spy0608	c.72G>A	p.Leu24Leu	24						0
6307	G	T	T	missense_variant	gacG	M5005_Spy0608	c.81G>T	p.Met27Ile	7						0
6310	A	C	C	missense_variant	gacG	M5005_Spy0608	c.84A>C	p.Arg28Ser	5						0
6311	T	A	A	missense_variant	gacG	M5005_Spy0608	c.85T>A	p.Ser29Thr	1						0
6329	G	A	A	missense_variant	gacG	M5005_Spy0608	c.103G>A	p.Val35Ile	77						0
6345	G	A	A	missense_variant	gacG	M5005_Spy0608	c.119G>A	p.Ser40Asn	2						0
6348	A	G	G	missense_variant	gacG	M5005_Spy0608	c.122A>G	p.Lys41Arg	64						0
6354	G	A	A	missense_variant	gacG	M5005_Spy0608	c.128G>A	p.Ser43Asn	2						0
6356	C	T	T	missense_variant	gacG	M5005_Spy0608	c.130C>T	p.His44Tyr	5						0
6358	C	T	T	synonymous_variant	gacG	M5005_Spy0608	c.132C>T	p.His44His	537						0
6361	A	G	G	synonymous_variant	gacG	M5005_Spy0608	c.135A>G	p.Glu45Glu	2						0
6362	G	A	A	missense_variant	gacG	M5005_Spy0608	c.136G>A	p.Asp46Asn	18						0
6372	G	A	A	missense_variant	gacG	M5005_Spy0608	c.146G>A	p.Arg49His	149						0
6376	C	T	T	synonymous_variant	gacG	M5005_Spy0608	c.150C>T	p.Leu50Leu	1						0
6382	C	T	T	synonymous_variant	gacG	M5005_Spy0608	c.156C>T	p.Asn52Asn	302						0
6383	C	T	T	missense_variant	gacG	M5005_Spy0608	c.157C>T	p.His53Tyr	1						0
6385	C	T	T	synonymous_variant	gacG	M5005_Spy0608	c.159C>T	p.His53His	10						0
6387	G	T,A	A	missense_variant	gacG	M5005_Spy0608	c.161G>A	p.Cys54Tyr	38	T	missense_variant	gacG	c.161G>T	p.Cys54Phe	3
6392	A	G	G	missense_variant	gacG	M5005_Spy0608	c.166A>G	p.Ile56Val	18						0
6403	T	C	C	synonymous_variant	gacG	M5005_Spy0608	c.177T>C	p.Phe59Phe	1						0
6404	T	C	C	synonymous_variant	gacG	M5005_Spy0608	c.178T>C	p.Leu60Leu	77						0
6445	C	T	T	synonymous_variant	gacG	M5005_Spy0608	c.219C>T	p.His73His	3						0
6459	T	C	C	missense_variant	gacG	M5005_Spy0608	c.233T>C	p.Ile78Thr	1938						0
6461	A	G	G	missense_variant	gacG	M5005_Spy0608	c.235A>G	p.Met79Val	24						0
6464	G	A	A	missense_variant	gacG	M5005_Spy0608	c.238G>A	p.Gly80Arg	1						0
6465	G	A	A	missense_variant	gacG	M5005_Spy0608	c.239G>A	p.Gly80Glu	1						0
6472	C	T	T	synonymous_variant	gacG	M5005_Spy0608	c.246C>T	p.Asp82Asp	2						0
6475	G	A	A	synonymous_variant	gacG	M5005_Spy0608	c.249G>A	p.Lys83Lys	730						0
6476	T	C	C	synonymous_variant	gacG	M5005_Spy0608	c.250T>C	p.Leu84Leu	4						0
6484	G	A	A	synonymous_variant	gacG	M5005_Spy0608	c.258G>A	p.Glu86Glu	2						0
6490	C	T	T	synonymous_variant	gacG	M5005_Spy0608	c.264C>T	p.Asp88Asp	6						0
6492	C	T	T	missense_variant	gacG	M5005_Spy0608	c.266C>T	p.Ser89Leu	3						0
6493	A	G	G	synonymous_variant	gacG	M5005_Spy0608	c.267A>G	p.Ser89Ser	3						0
6533	G	C	C	missense_variant	gacG	M5005_Spy0608	c.307G>C	p.Glu103Gln	1						0
6540	C	G	G	missense_variant	gacG	M5005_Spy0608	c.314C>G	p.Ala105Gly	738						0
6542	C	A	A	missense_variant	gacG	M5005_Spy0608	c.316C>A	p.Pro106Thr	3						0
6556	T	C	C	synonymous_variant	gacG	M5005_Spy0608	c.330T>C	p.Asn110Asn	30						0
6569	G	A	A	missense_variant	gacG	M5005_Spy0608	c.343G>A	p.Glu115Lys	6						0
6571	G	A	A	synonymous_variant	gacG	M5005_Spy0608	c.345G>A	p.Glu115Glu	9						0
6572	A	G	G	missense_variant	gacG	M5005_Spy0608	c.346A>G	p.Thr116Ala	1021						0
6575	G	A	A	missense_variant	gacG	M5005_Spy0608	c.349G>A	p.Val117Ile	7						0
6580	C	T	T	synonymous_variant	gacG	M5005_Spy0608	c.354C>T	p.Asp118Asp	39						0
6603	G	A	A	missense_variant	gacG	M5005_Spy0608	c.377G>A	p.Arg126Lys	6						0
6605	G	T	T	missense_variant	gacG	M5005_Spy0608	c.379G>T	p.Gly127Cys	1						0
6607	T	C	C	synonymous_variant	gacG	M5005_Spy0608	c.381T>C	p.Gly127Gly	1						0
6614	G	A	A	missense_variant	gacG	M5005_Spy0608	c.388G>A	p.Ala130Thr	130						0
6667	T	C	C	synonymous_variant	gacG	M5005_Spy0608	c.441T>C	p.Ile147Ile	1						0
6713	G	A	A	missense_variant	gacG	M5005_Spy0608	c.487G>A	p.Glu163Lys	14						0
6724	A	G	G	synonymous_variant	gacG	M5005_Spy0608	c.498A>G	p.Gln166Gln	5						0
6736	G	A	A	synonymous_variant	gacG	M5005_Spy0608	c.510G>A	p.Gln170Gln	885						0
6738	A	G	G	missense_variant	gacG	M5005_Spy0608	c.512A>G	p.His171Arg	6						0
6742	T	C	C	synonymous_variant	gacG	M5005_Spy0608	c.516T>C	p.Tyr172Tyr	276						0
6752	T	C	C	synonymous_variant	gacG	M5005_Spy0608	c.526T>C	p.Leu176Leu	1						0
6761	A	G	G	missense_variant	gacG	M5005_Spy0608	c.535A>G	p.Ile179Val	3						0
6769	A	G	G	synonymous_variant	gacG	M5005_Spy0608	c.543A>G	p.Leu181Leu	160						0
6774	A	G	G	missense_variant	gacG	M5005_Spy0608	c.548A>G	p.Glu183Gly	1						0
6778	C	A	A	synonymous_variant	gacG	M5005_Spy0608	c.552C>A	p.Gly184Gly	1						0
6789	A	G	G	missense_variant	gacG	M5005_Spy0608	c.563A>G	p.Gln188Arg	2						0
6790	G	A	A	synonymous_variant	gacG	M5005_Spy0608	c.564G>A	p.Gln188Gln	1						0
6796	C	A	A	synonymous_variant	gacG	M5005_Spy0608	c.570C>A	p.Val190Val	14						0

6812	G	A	A	missense_variant	gacG	M5005_Spy0608	c.586G>A	p.Ala196Thr	141						0
6813	C	T	T	missense_variant	gacG	M5005_Spy0608	c.587C>T	p.Ala196Val	110						0
6831	C	T	T	missense_variant	gacG	M5005_Spy0608	c.605C>T	p.Pro202Leu	8						0
6833	C	T	T	missense_variant	gacG	M5005_Spy0608	c.607C>T	p.His203Tyr	2						0
6838	G	A	A	synonymous_variant	gacG	M5005_Spy0608	c.612G>A	p.Pro204Pro	1						0
6856	T	C	C	synonymous_variant	gacG	M5005_Spy0608	c.630T>C	p.Asn210Asn	4						0
6859	A	G	G	synonymous_variant	gacG	M5005_Spy0608	c.633A>G	p.Pro211Pro	1						0
6869	T	C	C	synonymous_variant	gacG	M5005_Spy0608	c.643T>C	p.Leu215Leu	794						0
6871	A	G	G	synonymous_variant	gacG	M5005_Spy0608	c.645A>G	p.Leu215Leu	92						0
6875	C	T	T	missense_variant	gacG	M5005_Spy0608	c.649C>T	p.His217Tyr	1						0
6919	G	A	A	synonymous_variant	gacG	M5005_Spy0608	c.693G>A	p.Gln231Gln	1						0
6920	C	T,A	A	missense_variant	gacG	M5005_Spy0608	c.694C>A	p.His232Asn	18	T	missense_variant	gacG	c.694C>T	p.His232Tyr	4
6926	G	A	A	missense_variant	gacG	M5005_Spy0608	c.700G>A	p.Ala234Thr	3						0
6934	C	T	T	synonymous_variant	gacG	M5005_Spy0608	c.708C>T	p.Tyr236Tyr	2						0
6938	T	C	C	synonymous_variant	gacG	M5005_Spy0608	c.712T>C	p.Leu238Leu	1688						0
6950	C	G	G	missense_variant	gacG	M5005_Spy0608	c.724C>G	p.Arg242Gly	35						0
6952	A	G	G	synonymous_variant	gacG	M5005_Spy0608	c.726A>G	p.Arg242Arg	2						0
6955	A	G	G	synonymous_variant	gacG	M5005_Spy0608	c.729A>G	p.Glu243Glu	2						0
6960	C	T	T	missense_variant	gacG	M5005_Spy0608	c.734C>T	p.Thr245Ile	1						0
6961	C	T	T	synonymous_variant	gacG	M5005_Spy0608	c.735C>T	p.Thr245Thr	9						0
6962	A	T	T	missense_variant	gacG	M5005_Spy0608	c.736A>T	p.Asn246Tyr	76						0
6967	T	C	C	synonymous_variant	gacG	M5005_Spy0608	c.741T>C	p.Tyr247Tyr	3						0
6973	A	G	G	missense_variant	gacG	M5005_Spy0608	c.747A>G	p.Ile249Met	25						0
6974	G	A	A	missense_variant	gacG	M5005_Spy0608	c.748G>A	p.Asp250Asn	1						0
6979	T	C	C	synonymous_variant	gacG	M5005_Spy0608	c.753T>C	p.Leu251Leu	1						0
6983	G	A	A	missense_variant	gacG	M5005_Spy0608	c.757G>A	p.Val253Ile	1						0
7012	A	G	G	synonymous_variant	gacG	M5005_Spy0608	c.786A>G	p.Pro262Pro	3						0
7016	A	G	G	missense_variant	gacG	M5005_Spy0608	c.790A>G	p.Thr264Ala	1938						0
7017	C	T	T	missense_variant	gacG	M5005_Spy0608	c.791C>T	p.Thr264Ile	2						0
7020	A	G	G	missense_variant	gacG	M5005_Spy0608	c.794A>G	p.Lys265Arg	31						0
7041	A	C	C	missense_variant	gacG	M5005_Spy0608	c.815A>C	p.Tyr272Ser	1						0
7042	C	T	T	synonymous_variant	gacG	M5005_Spy0608	c.816C>T	p.Tyr272Tyr	1						0
7048	C	T	T	synonymous_variant	gacG	M5005_Spy0608	c.822C>T	p.Asn274Asn	1						0
7051	C	T	T	synonymous_variant	gacG	M5005_Spy0608	c.825C>T	p.Cys275Cys	3						0
7053	A	G	G	missense_variant	gacG	M5005_Spy0608	c.827A>G	p.Gln276Arg	2						0
7060	A	G	G	synonymous_variant	gacG	M5005_Spy0608	c.834A>G	p.Leu278Leu	1						0
7067	C	A	A	missense_variant	gacG	M5005_Spy0608	c.841C>A	p.Gln281Lys	154						0
7083	T	C	C	missense_variant	gacG	M5005_Spy0608	c.857T>C	p.Val286Ala	4						0
7112	G	A	A	missense_variant	gacG	M5005_Spy0608	c.886G>A	p.Asp296Asn	2						0
7118	C	A	A	missense_variant	gacG	M5005_Spy0608	c.892C>A	p.Leu298Ile	1						0
7132	A	T	T	missense_variant	gacG	M5005_Spy0608	c.906A>T	p.Leu302Phe	1						0
7136	G	A	A	missense_variant	gacG	M5005_Spy0608	c.910G>A	p.Ala304Thr	17						0
7137	C	T	T	missense_variant	gacG	M5005_Spy0608	c.911C>T	p.Ala304Val	3						0
7141	C	T,A	A	missense_variant	gacG	M5005_Spy0608	c.915C>A	p.Phe305Leu	101	T	synonymous_variant	gacG	c.915C>T	p.Phe305Phe	17
7145	A	G,T	G	missense_variant	gacG	M5005_Spy0608	c.919A>G	p.Asn307Asp	1907	T	missense_variant	gacG	c.919A>T	p.Asn307Tyr	18
7147	C	T	T	synonymous_variant	gacG	M5005_Spy0608	c.921C>T	p.Asn307Asn	5						0
7152	A	G	G	missense_variant	gacG	M5005_Spy0608	c.926A>G	p.Asn309Ser	1						0
7156	C	T	T	synonymous_variant	gacG	M5005_Spy0608	c.930C>T	p.Phe310Phe	1						0
7172	A	G	G	missense_variant	gacG	M5005_Spy0608	c.946A>G	p.Ile316Val	3						0
7177	A	G	G	synonymous_variant	gacG	M5005_Spy0608	c.951A>G	p.Thr317Thr	21						0
7199	A	G	G	missense_variant	gacG	M5005_Spy0608	c.973A>G	p.Lys325Glu	15						0
7202	G	A	A	missense_variant	gacG	M5005_Spy0608	c.976G>A	p.Glu326Lys	19						0
7221	A	G	G	missense_variant	gacG	M5005_Spy0608	c.995A>G	p.Gln332Arg	2						0
7225	A	T,G	T	missense_variant	gacG	M5005_Spy0608	c.999A>T	p.Arg333Ser	17	G	synonymous_variant	gacG	c.999A>G	p.Arg333Arg	2
7230	G	T	T	missense_variant	gacG	M5005_Spy0608	c.1004G>T	p.Gly335Val	18						0
7233	A	G	G	missense_variant	gacG	M5005_Spy0608	c.1007A>G	p.Lys336Arg	10						0
7242	A	G	G	missense_variant	gacG	M5005_Spy0608	c.1016A>G	p.Asp339Gly	1						0
7249	C	T	T	synonymous_variant	gacG	M5005_Spy0608	c.1023C>T	p.Arg341Arg	3						0
7333	C	T	T	synonymous_variant	gacG	M5005_Spy0608	c.1107C>T	p.Phe369Phe	1						0
7393	G	A	A	synonymous_variant	gacG	M5005_Spy0608	c.1167G>A	p.Leu389Leu	4						0
7397	G	A	A	missense_variant	gacG	M5005_Spy0608	c.1171G>A	p.Asp391Asn	4						0
7405	C	T	T	synonymous_variant	gacG	M5005_Spy0608	c.1179C>T	p.Leu393Leu	3						0

7408	G	T	T	synonymous_variant	gacG	M5005_Spy0608	c.1182G>T	p.Val394Val	33						0
7413	C	A	A	missense_variant	gacG	M5005_Spy0608	c.1187C>A	p.Pro396Gln	1						0
7427	C	A	A	missense_variant	gacG	M5005_Spy0608	c.1201C>A	p.Leu401Ile	1						0
7433	G	A	A	missense_variant	gacG	M5005_Spy0608	c.1207G>A	p.Ala403Thr	6						0
7434	C	T,G	G	missense_variant	gacG	M5005_Spy0608	c.1208C>G	p.Ala403Gly	129	T	missense_variant	gacG	c.1208C>T	p.Ala403Val	18
7445	G	A,C	A	missense_variant	gacG	M5005_Spy0608	c.1219G>A	p.Asp407Asn	735	C	missense_variant	gacG	c.1219G>C	p.Asp407His	25
7457	A	G	G	missense_variant	gacG	M5005_Spy0608	c.1231A>G	p.Ile411Val	9						0
7459	T	C	C	synonymous_variant	gacG	M5005_Spy0608	c.1233T>C	p.Ile411Ile	3						0
7468	A	G	G	synonymous_variant	gacG	M5005_Spy0608	c.1242A>G	p.Ala414Ala	1						0
7504	C	T	T	synonymous_variant	gacG	M5005_Spy0608	c.1278C>T	p.Val426Val	1						0
7516	T	C	C	synonymous_variant	gacG	M5005_Spy0608	c.1290T>C	p.Asn430Asn	4						0
7528	C	T	T	synonymous_variant	gacG	M5005_Spy0608	c.1302C>T	p.Ile434Ile	2						0
7535	G	A,C	A	missense_variant	gacG	M5005_Spy0608	c.1309G>A	p.Glu437Lys	17	C	missense_variant	gacG	c.1309G>C	p.Glu437Gln	5
7566	T	C	C	missense_variant	gacG	M5005_Spy0608	c.1340T>C	p.Val447Ala	1						0
7567	T	A	A	synonymous_variant	gacG	M5005_Spy0608	c.1341T>A	p.Val447Val	1						0
7573	A	T	T	missense_variant	gacG	M5005_Spy0608	c.1347A>T	p.Lys449Asn	1						0
7577	A	T	T	missense_variant	gacG	M5005_Spy0608	c.1351A>T	p.Ile451Phe	1						0
7581	A	T	T	missense_variant	gacG	M5005_Spy0608	c.1355A>T	p.Asp452Val	1						0
7585	T	C	C	synonymous_variant	gacG	M5005_Spy0608	c.1359T>C	p.Phe453Phe	6						0
7589	G	A	A	missense_variant	gacG	M5005_Spy0608	c.1363G>A	p.Ala455Thr	118						0
7597	T	C	C	synonymous_variant	gacG	M5005_Spy0608	c.1371T>C	p.Asp457Asp	1						0
7604	G	A	A	missense_variant	gacG	M5005_Spy0608	c.1378G>A	p.Val460Ile	4						0
7639	C	T	T	synonymous_variant	gacG	M5005_Spy0608	c.1413C>T	p.Tyr471Tyr	12						0
7663	T	C	C	synonymous_variant	gacG	M5005_Spy0608	c.1437T>C	p.Asp479Asp	1						0
7668	A	G	G	missense_variant	gacG	M5005_Spy0608	c.1442A>G	p.Glu481Gly	1						0
7675	T	C	C	synonymous_variant	gacG	M5005_Spy0608	c.1449T>C	p.Thr483Thr	2						0
7696	A	G	G	synonymous_variant	gacG	M5005_Spy0608	c.1470A>G	p.Glu490Glu	473						0
7724	G	A	A	missense_variant	gacG	M5005_Spy0608	c.1498G>A	p.Ala500Thr	3						0
7733	C	A	A	synonymous_variant	gacG	M5005_Spy0608	c.1507C>A	p.Arg503Arg	12						0
7739	C	T	T	missense_variant	gacG	M5005_Spy0608	c.1513C>T	p.Leu505Phe	129						0
7753	G	A	A	synonymous_variant	gacG	M5005_Spy0608	c.1527G>A	p.Ala509Ala	1						0
7760	G	A	A	missense_variant	gacG	M5005_Spy0608	c.1534G>A	p.Asp512Asn	1949						0
7761	A	G	G	missense_variant	gacG	M5005_Spy0608	c.1535A>G	p.Asp512Gly	1						0
7790	C	T	T	missense_variant	gacG	M5005_Spy0608	c.1564C>T	p.Pro522Ser	94						0
7805	C	T	T	missense_variant	gacG	M5005_Spy0608	c.1579C>T	p.Pro527Ser	1						0
7823	C	T	T	synonymous_variant	gacG	M5005_Spy0608	c.1597C>T	p.Leu533Leu	2						0
7852	T	C	C	synonymous_variant	gacG	M5005_Spy0608	c.1626T>C	p.Ala542Ala	52						0
7857	C	G	G	missense_variant	gacG	M5005_Spy0608	c.1631C>G	p.Thr544Ser	11						0
7861	C	T	T	synonymous_variant	gacG	M5005_Spy0608	c.1635C>T	p.Tyr545Tyr	1						0
7888	C	A	A	synonymous_variant	gacG	M5005_Spy0608	c.1662C>A	p.Ile554Ile	1						0
7891	A	G	G	synonymous_variant	gacG	M5005_Spy0608	c.1665A>G	p.Lys555Lys	3						0
7900	G	A	A	synonymous_variant	gacG	M5005_Spy0608	c.1674G>A	p.Leu558Leu	1701						0
7912	C	T	T	synonymous_variant	gacG	M5005_Spy0608	c.1686C>T	p.Ile562Ile	856						0
7918	T	A	A	synonymous_variant	gacG	M5005_Spy0608	c.1692T>A	p.Gly564Gly	4						0
7922	G	A	A	missense_variant	gacG	M5005_Spy0608	c.1696G>A	p.Ala566Thr	2						0
7927	A	T,G	T	missense_variant	gacG	M5005_Spy0608	c.1701A>T	p.Lys567Asn	17	G	synonymous_variant	gacG	c.1701A>G	p.Lys567Lys	1
7936	G	A	A	synonymous_variant	gacG	M5005_Spy0608	c.1710G>A	p.Lys570Lys	17						0
7948	G	T	T	missense_variant	gacG	M5005_Spy0608	c.1722G>T	p.Leu574Phe	2						0
7964	C	T	T	missense_variant	gacG	M5005_Spy0608	c.1738C>T	p.Leu580Phe	2						0
7980	C	T	T	synonymous_variant	gacH	M5005_Spy0609	c.12C>T	p.Asp4Asp	2						0
7982	C	T	T	missense_variant	gacH	M5005_Spy0609	c.14C>T	p.Thr5Ile	18						0
7994	C	T	T	missense_variant	gacH	M5005_Spy0609	c.26C>T	p.Thr9Ile	2						0
8010	T	C	C	synonymous_variant	gacH	M5005_Spy0609	c.42T>C	p.Ile14Ile	75						0
8013	T	C,A	A	missense_variant	gacH	M5005_Spy0609	c.45T>A	p.Ser15Arg	43	C	synonymous_variant	gacH	c.45T>C	p.Ser15Ser	57
8016	C	T	T	synonymous_variant	gacH	M5005_Spy0609	c.48C>T	p.His16His	82						0
8017	C	T	T	missense_variant	gacH	M5005_Spy0609	c.49C>T	p.His17Tyr	4						0
8024	T	C	C	missense_variant	gacH	M5005_Spy0609	c.56T>C	p.Ile19Thr	442						0
8028	T	C	C	synonymous_variant	gacH	M5005_Spy0609	c.60T>C	p.Leu20Leu	2						0
8038	G	A	A	missense_variant	gacH	M5005_Spy0609	c.70G>A	p.Gly24Ser	68						0
8058	C	T	T	synonymous_variant	gacH	M5005_Spy0609	c.90C>T	p.Tyr30Tyr	391						0
8067	G	T	T	synonymous_variant	gacH	M5005_Spy0609	c.99G>T	p.Ala33Ala	6						0
8077	G	A	A	missense_variant	gacH	M5005_Spy0609	c.109G>A	p.Val37Ile	9						0

8081	G	A	A	missense_variant	gacH	M5005_Spy0609	c.113G>A	p.Ser38Asn	42						0
8086	A	G	G	missense_variant	gacH	M5005_Spy0609	c.118A>G	p.Thr40Ala	1						0
8088	G	A	A	synonymous_variant	gacH	M5005_Spy0609	c.120G>A	p.Thr40Thr	3						0
8089	G	A	A	missense_variant	gacH	M5005_Spy0609	c.121G>A	p.Ala41Thr	903						0
8094	A	G	G	synonymous_variant	gacH	M5005_Spy0609	c.126A>G	p.Gln42Gln	6						0
8098	G	A	A	missense_variant	gacH	M5005_Spy0609	c.130G>A	p.Val44Ile	6						0
8105	A	C	C	missense_variant	gacH	M5005_Spy0609	c.137A>C	p.Tyr46Ser	3						0
8110	G	A	A	missense_variant	gacH	M5005_Spy0609	c.142G>A	p.Ala48Thr	1794						0
8111	C	T	T	missense_variant	gacH	M5005_Spy0609	c.143C>T	p.Ala48Val	12						0
8116	T	C	C	synonymous_variant	gacH	M5005_Spy0609	c.148T>C	p.Leu50Leu	1						0
8129	C	T	T	missense_variant	gacH	M5005_Spy0609	c.161C>T	p.Ser54Phe	1						0
8136	G	T	T	synonymous_variant	gacH	M5005_Spy0609	c.168G>T	p.Val56Val	2						0
8139	A	C	C	synonymous_variant	gacH	M5005_Spy0609	c.171A>C	p.Gly57Gly	9						0
8162	T	C	C	missense_variant	gacH	M5005_Spy0609	c.194T>C	p.Leu65Ser	1						0
8191	C	A	A	synonymous_variant	gacH	M5005_Spy0609	c.223C>A	p.Arg75Arg	10						0
8193	A	C	C	synonymous_variant	gacH	M5005_Spy0609	c.225A>C	p.Arg75Arg	1						0
8197	T	C	C	synonymous_variant	gacH	M5005_Spy0609	c.229T>C	p.Leu77Leu	203						0
8199	G	A	A	synonymous_variant	gacH	M5005_Spy0609	c.231G>A	p.Leu77Leu	3						0
8200	T	C	C	missense_variant	gacH	M5005_Spy0609	c.232T>C	p.Phe78Leu	33						0
8222	T	C	C	missense_variant	gacH	M5005_Spy0609	c.254T>C	p.Val85Ala	20						0
8239	G	A	A	missense_variant	gacH	M5005_Spy0609	c.271G>A	p.Val91Ile	10						0
8241	C	T	T	synonymous_variant	gacH	M5005_Spy0609	c.273C>T	p.Val91Val	1						0
8244	C	T	T	synonymous_variant	gacH	M5005_Spy0609	c.276C>T	p.Ser92Ser	1						0
8250	G	C	C	missense_variant	gacH	M5005_Spy0609	c.282G>C	p.Trp94Cys	1						0
8269	C	T	T	synonymous_variant	gacH	M5005_Spy0609	c.301C>T	p.Leu101Leu	3						0
8274	T	C	C	synonymous_variant	gacH	M5005_Spy0609	c.306T>C	p.Asn102Asn	1						0
8276	A	T	T	missense_variant	gacH	M5005_Spy0609	c.308A>T	p.Asp103Val	6						0
8280	A	T	T	missense_variant	gacH	M5005_Spy0609	c.312A>T	p.Lys104Asn	4						0
8281	C	T	T	missense_variant	gacH	M5005_Spy0609	c.313C>T	p.Arg105Trp	1						0
8283	G	A	A	synonymous_variant	gacH	M5005_Spy0609	c.315G>A	p.Arg105Arg	1						0
8303	C	T	T	missense_variant	gacH	M5005_Spy0609	c.335C>T	p.Thr112Ile	1						0
8319	T	C	C	synonymous_variant	gacH	M5005_Spy0609	c.351T>C	p.Tyr117Tyr	48						0
8330	C	A	A	missense_variant	gacH	M5005_Spy0609	c.362C>A	p.Ala121Asp	1						0
8334	A	G	G	synonymous_variant	gacH	M5005_Spy0609	c.366A>G	p.Leu122Leu	1						0
8339	C	A,T	A	missense_variant	gacH	M5005_Spy0609	c.371C>A	p.Ser124Tyr	206	T	missense_variant	gacH	c.371C>T	p.Ser124Phe	4
8344	C	T	T	missense_variant	gacH	M5005_Spy0609	c.376C>T	p.Leu126Phe	10						0
8346	C	T	T	synonymous_variant	gacH	M5005_Spy0609	c.378C>T	p.Leu126Leu	1						0
8348	T	G	G	missense_variant	gacH	M5005_Spy0609	c.380T>G	p.Ile127Ser	2						0
8352	T	C	C	synonymous_variant	gacH	M5005_Spy0609	c.384T>C	p.Ile128Ile	1						0
8358	G	A	A	synonymous_variant	gacH	M5005_Spy0609	c.390G>A	p.Val130Val	3						0
8361	G	A	A	missense_variant	gacH	M5005_Spy0609	c.393G>A	p.Met131Ile	43						0
8363	C	T	T	missense_variant	gacH	M5005_Spy0609	c.395C>T	p.Ala132Val	1						0
8364	C	T	T	synonymous_variant	gacH	M5005_Spy0609	c.396C>T	p.Ala132Ala	13						0
8365	A	G	G	missense_variant	gacH	M5005_Spy0609	c.397A>G	p.Thr133Ala	1						0
8367	T	C	C	synonymous_variant	gacH	M5005_Spy0609	c.399T>C	p.Thr133Thr	1						0
8370	A	G	G	synonymous_variant	gacH	M5005_Spy0609	c.402A>G	p.Leu134Leu	18						0
8382	G	A	A	synonymous_variant	gacH	M5005_Spy0609	c.414G>A	p.Leu138Leu	55						0
8384	T	C	C	missense_variant	gacH	M5005_Spy0609	c.416T>C	p.Val139Ala	1						0
8385	A	C	C	synonymous_variant	gacH	M5005_Spy0609	c.417A>C	p.Val139Val	2						0
8387	C	T	T	missense_variant	gacH	M5005_Spy0609	c.419C>T	p.Ala140Val	12						0
8422	T	C	C	synonymous_variant	gacH	M5005_Spy0609	c.454T>C	p.Leu152Leu	9						0
8425	G	T	T	missense_variant	gacH	M5005_Spy0609	c.457G>T	p.Gly153Cys	88						0
8447	C	T	T	missense_variant	gacH	M5005_Spy0609	c.479C>T	p.Ser160Phe	1						0
8451	A	G	G	synonymous_variant	gacH	M5005_Spy0609	c.483A>G	p.Val161Val	12						0
8470	G	A	A	missense_variant	gacH	M5005_Spy0609	c.502G>A	p.Val168Met	5						0
8480	A	G	G	missense_variant	gacH	M5005_Spy0609	c.512A>G	p.Asn171Ser	257						0
8483	A	G	G	missense_variant	gacH	M5005_Spy0609	c.515A>G	p.Asp172Gly	5						0
8491	C	T	T	synonymous_variant	gacH	M5005_Spy0609	c.523C>T	p.Leu175Leu	3						0
8502	A	G	G	synonymous_variant	gacH	M5005_Spy0609	c.534A>G	p.Leu178Leu	4						0
8510	C	A	A	missense_variant	gacH	M5005_Spy0609	c.542C>A	p.Ser181Tyr	1						0
8530	A	G	G	missense_variant	gacH	M5005_Spy0609	c.562A>G	p.Thr188Ala	3						0
8531	C	G	G	missense_variant	gacH	M5005_Spy0609	c.563C>G	p.Thr188Arg	502						0

8534	C	T	T	missense_variant	gacH	M5005_Spy0609	c.566C>T	p.Ala189Val	1						0
8537	G	A	A	missense_variant	gacH	M5005_Spy0609	c.569G>A	p.Gly190Asp	13						0
8544	C	A	A	missense_variant	gacH	M5005_Spy0609	c.576C>A	p.Ser192Arg	3						0
8552	C	T	T	missense_variant	gacH	M5005_Spy0609	c.584C>T	p.Ala195Val	16						0
8556	G	A	A	synonymous_variant	gacH	M5005_Spy0609	c.588G>A	p.Leu196Leu	6						0
8570	G	A	A	missense_variant	gacH	M5005_Spy0609	c.602G>A	p.Gly201Glu	2						0
8571	A	G	G	synonymous_variant	gacH	M5005_Spy0609	c.603A>G	p.Gly201Gly	1						0
8572	C	T	T	synonymous_variant	gacH	M5005_Spy0609	c.604C>T	p.Leu202Leu	1						0
8574	G	A	A	synonymous_variant	gacH	M5005_Spy0609	c.606G>A	p.Leu202Leu	4						0
8576	T	C,G	C	missense_variant	gacH	M5005_Spy0609	c.608T>C	p.Leu203Ser	2	G	missense_variant	gacH	c.608T>G	p.Leu203Trp	1
8578	A	T	T	missense_variant	gacH	M5005_Spy0609	c.610A>T	p.Ile204Leu	30						0
8579	T	C	C	missense_variant	gacH	M5005_Spy0609	c.611T>C	p.Ile204Thr	1						0
8580	A	C	C	synonymous_variant	gacH	M5005_Spy0609	c.612A>C	p.Ile204Ile	2						0
8584	T	C,A	A	missense_variant	gacH	M5005_Spy0609	c.616T>A	p.Leu206Ile	11	C	synonymous_variant	gacH	c.616T>C	p.Leu206Leu	1
8587	C	T	T	missense_variant	gacH	M5005_Spy0609	c.619C>T	p.Leu207Phe	1						0
8589	T	C	C	synonymous_variant	gacH	M5005_Spy0609	c.621T>C	p.Leu207Leu	11						0
8592	C	A	A	synonymous_variant	gacH	M5005_Spy0609	c.624C>A	p.Val208Val	2						0
8603	C	T	T	missense_variant	gacH	M5005_Spy0609	c.635C>T	p.Thr212Ile	56						0
8611	T	G	G	missense_variant	gacH	M5005_Spy0609	c.643T>G	p.Tyr215Asp	1						0
8642	A	G	G	missense_variant	gacH	M5005_Spy0609	c.674A>G	p.Lys225Arg	5						0
8643	G	C	C	missense_variant	gacH	M5005_Spy0609	c.675G>C	p.Lys225Asn	58						0
8645	C	T	T	missense_variant	gacH	M5005_Spy0609	c.677C>T	p.Ser226Leu	6						0
8653	C	T	T	missense_variant	gacH	M5005_Spy0609	c.685C>T	p.Pro229Ser	302						0
8654	C	T	T	missense_variant	gacH	M5005_Spy0609	c.686C>T	p.Pro229Leu	92						0
8672	C	T	T	missense_variant	gacH	M5005_Spy0609	c.704C>T	p.Ala235Val	9						0
8674	A	G	G	missense_variant	gacH	M5005_Spy0609	c.706A>G	p.Thr236Ala	1						0
8680	A	G	G	missense_variant	gacH	M5005_Spy0609	c.712A>G	p.Ser238Gly	12						0
8683	T	C	C	synonymous_variant	gacH	M5005_Spy0609	c.715T>C	p.Leu239Leu	35						0
8700	T	C	C	synonymous_variant	gacH	M5005_Spy0609	c.732T>C	p.Val244Val	13						0
8706	C	T	T	synonymous_variant	gacH	M5005_Spy0609	c.738C>T	p.Asn246Asn	7						0
8715	C	T	T	synonymous_variant	gacH	M5005_Spy0609	c.747C>T	p.Phe249Phe	156						0
8724	A	C	C	synonymous_variant	gacH	M5005_Spy0609	c.756A>C	p.Gly252Gly	374						0
8745	G	T	T	missense_variant	gacH	M5005_Spy0609	c.777G>T	p.Leu259Phe	1						0
8751	A	G	G	synonymous_variant	gacH	M5005_Spy0609	c.783A>G	p.Gly261Gly	2						0
8759	T	C	C	missense_variant	gacH	M5005_Spy0609	c.791T>C	p.Val264Ala	17						0
8767	G	A	A	missense_variant	gacH	M5005_Spy0609	c.799G>A	p.Gly267Arg	3						0
8769	A	G	G	synonymous_variant	gacH	M5005_Spy0609	c.801A>G	p.Gly267Gly	7						0
8770	G	A	A	missense_variant	gacH	M5005_Spy0609	c.802G>A	p.Ala268Thr	54						0
8771	C	T	T	missense_variant	gacH	M5005_Spy0609	c.803C>T	p.Ala268Val	2						0
8772	T	C	C	synonymous_variant	gacH	M5005_Spy0609	c.804T>C	p.Ala268Ala	1						0
8797	A	C	C	missense_variant	gacH	M5005_Spy0609	c.829A>C	p.Thr277Pro	1						0
8799	A	G	G	synonymous_variant	gacH	M5005_Spy0609	c.831A>G	p.Thr277Thr	7						0
8806	G	T	T	missense_variant	gacH	M5005_Spy0609	c.838G>T	p.Ala280Ser	1						0
8809	C	A	A	missense_variant	gacH	M5005_Spy0609	c.841C>A	p.Leu281Ile	92						0
8820	C	T	T	synonymous_variant	gacH	M5005_Spy0609	c.852C>T	p.Tyr284Tyr	4						0
8825	T	C	C	missense_variant	gacH	M5005_Spy0609	c.857T>C	p.Ile286Thr	1						0
8845	A	C	C	missense_variant	gacH	M5005_Spy0609	c.877A>C	p.Thr293Pro	30						0
8852	T	C	C	missense_variant	gacH	M5005_Spy0609	c.884T>C	p.Phe295Ser	16						0
8863	A	C	C	missense_variant	gacH	M5005_Spy0609	c.895A>C	p.Ile299Leu	11						0
8866	C	T	T	synonymous_variant	gacH	M5005_Spy0609	c.898C>T	p.Leu300Leu	1						0
8867	T	C	C	missense_variant	gacH	M5005_Spy0609	c.899T>C	p.Leu300Pro	6						0
8873	C	A	A	missense_variant	gacH	M5005_Spy0609	c.905C>A	p.Thr302Lys	1						0
8877	T	A	A	synonymous_variant	gacH	M5005_Spy0609	c.909T>A	p.Ile303Ile	3						0
8878	A	T	T	missense_variant	gacH	M5005_Spy0609	c.910A>T	p.Ile304Phe	1						0
8883	T	G	G	synonymous_variant	gacH	M5005_Spy0609	c.915T>G	p.Ser305Ser	2						0
8887	G	T	T	missense_variant	gacH	M5005_Spy0609	c.919G>T	p.Val307Phe	1						0
8889	T	A	A	synonymous_variant	gacH	M5005_Spy0609	c.921T>A	p.Val307Val	1						0
8897	T	A	A	stop_gained	gacH	M5005_Spy0609	c.929T>A	p.Leu310*	3						0
8902	G	A	A	missense_variant	gacH	M5005_Spy0609	c.934G>A	p.Glu312Lys	1						0
8906	C	A	A	stop_gained	gacH	M5005_Spy0609	c.938C>A	p.Ser313*	1						0
8909	T	G,*	G	missense_variant	gacH	M5005_Spy0609	c.941T>G	p.Met314Arg	3						1
8914	A	G	G	missense_variant	gacH	M5005_Spy0609	c.946A>G	p.Ser316Gly	2						0

8916	C	G	G	missense_variant	gacH	M5005_Spy0609	c.948C>G	p.Ser316Arg	2						0
8922	G	A	A	synonymous_variant	gacH	M5005_Spy0609	c.954G>A	p.Pro318Pro	510						0
8933	C	G	G	missense_variant	gacH	M5005_Spy0609	c.965C>G	p.Thr322Ser	7						0
8947	T	C	C	synonymous_variant	gacH	M5005_Spy0609	c.979T>C	p.Leu327Leu	1						0
8973	C	T	T	synonymous_variant	gacH	M5005_Spy0609	c.1005C>T	p.Ser335Ser	11						0
8989	G	T,A	A	missense_variant	gacH	M5005_Spy0609	c.1021G>A	p.Val341Met	39	T	missense_variant	gacH	c.1021G>T	p.Val341Leu	1
9004	G	A	A	missense_variant	gacH	M5005_Spy0609	c.1036G>A	p.Val346Ile	7						0
9007	G	T	T	missense_variant	gacH	M5005_Spy0609	c.1039G>T	p.Val347Leu	2						0
9033	A	G	G	synonymous_variant	gacH	M5005_Spy0609	c.1065A>G	p.Val355Val	1383						0
9046	C	T	T	missense_variant	gacH	M5005_Spy0609	c.1078C>T	p.His360Tyr	1						0
9050	G	A	A	missense_variant	gacH	M5005_Spy0609	c.1082G>A	p.Gly361Asp	34						0
9069	A	T	T	missense_variant	gacH	M5005_Spy0609	c.1101A>T	p.Lys367Asn	1						0
9078	G	A	A	missense_variant	gacH	M5005_Spy0609	c.1110G>A	p.Met370Ile	1						0
9082	C	T	T	missense_variant	gacH	M5005_Spy0609	c.1114C>T	p.Pro372Ser	1						0
9087	C	T	T	synonymous_variant	gacH	M5005_Spy0609	c.1119C>T	p.Val373Val	1						0
9095	C	T	T	missense_variant	gacH	M5005_Spy0609	c.1127C>T	p.Ala376Val	52						0
9100	G	A	A	missense_variant	gacH	M5005_Spy0609	c.1132G>A	p.Ala378Thr	18						0
9103	G	C	C	missense_variant	gacH	M5005_Spy0609	c.1135G>C	p.Val379Leu	2						0
9115	T	G,A	A	missense_variant	gacH	M5005_Spy0609	c.1147T>A	p.Phe383Ile	197	G	missense_variant	gacH	c.1147T>G	p.Phe383Val	70
9126	T	C	C	synonymous_variant	gacH	M5005_Spy0609	c.1158T>C	p.Ser386Ser	48						0
9129	T	C	C	synonymous_variant	gacH	M5005_Spy0609	c.1161T>C	p.Cys387Cys	2						0
9136	T	C	C	synonymous_variant	gacH	M5005_Spy0609	c.1168T>C	p.Leu390Leu	1						0
9145	T	C	C	missense_variant	gacH	M5005_Spy0609	c.1177T>C	p.Phe393Leu	6						0
9166	A	G	G	missense_variant	gacH	M5005_Spy0609	c.1198A>G	p.Lys400Glu	1						0
9172	T	C	C	synonymous_variant	gacH	M5005_Spy0609	c.1204T>C	p.Leu402Leu	18						0
9179	G	A	A	missense_variant	gacH	M5005_Spy0609	c.1211G>A	p.Gly404Asp	2						0
9186	G	T	T	synonymous_variant	gacH	M5005_Spy0609	c.1218G>T	p.Pro406Pro	1						0
9197	C	T	T	missense_variant	gacH	M5005_Spy0609	c.1229C>T	p.Ala410Val	6						0
9216	C	T	T	synonymous_variant	gacH	M5005_Spy0609	c.1248C>T	p.Asp416Asp	1240						0
9222	C	T	T	synonymous_variant	gacH	M5005_Spy0609	c.1254C>T	p.Asn418Asn	48						0
9228	A	G	G	synonymous_variant	gacH	M5005_Spy0609	c.1260A>G	p.Leu420Leu	2						0
9244	G	A	A	missense_variant	gacH	M5005_Spy0609	c.1276G>A	p.Ala426Thr	3						0
9268	G	A	A	missense_variant	gacH	M5005_Spy0609	c.1300G>A	p.Val434Ile	1						0
9283	G	A	A	missense_variant	gacH	M5005_Spy0609	c.1315G>A	p.Val439Met	23						0
9291	G	A	A	synonymous_variant	gacH	M5005_Spy0609	c.1323G>A	p.Lys441Lys	2						0
9296	T	A	A	missense_variant	gacH	M5005_Spy0609	c.1328T>A	p.Ile443Lys	1						0
9301	G	A	A	missense_variant	gacH	M5005_Spy0609	c.1333G>A	p.Glu445Lys	1						0
9306	A	G	G	synonymous_variant	gacH	M5005_Spy0609	c.1338A>G	p.Lys446Lys	9						0
9310	A	G	G	missense_variant	gacH	M5005_Spy0609	c.1342A>G	p.Thr448Ala	7						0
9313	A	G	G	missense_variant	gacH	M5005_Spy0609	c.1345A>G	p.Asn449Asp	6						0
9321	C	T	T	synonymous_variant	gacH	M5005_Spy0609	c.1353C>T	p.Ser451Ser	229						0
9322	C	A	A	missense_variant	gacH	M5005_Spy0609	c.1354C>A	p.Gln452Lys	3						0
9327	A	C	C	missense_variant	gacH	M5005_Spy0609	c.1359A>C	p.Glu453Asp	117						0
9335	C	T	T	missense_variant	gacH	M5005_Spy0609	c.1367C>T	p.Ala456Val	1						0
9336	G	A	A	synonymous_variant	gacH	M5005_Spy0609	c.1368G>A	p.Ala456Ala	7						0
9344	C	T	T	missense_variant	gacH	M5005_Spy0609	c.1376C>T	p.Ala459Val	1						0
9384	C	T	T	synonymous_variant	gacH	M5005_Spy0609	c.1416C>T	p.Asp472Asp	1						0
9393	T	C	C	synonymous_variant	gacH	M5005_Spy0609	c.1425T>C	p.Asn475Asn	13						0
9395	A	G	G	missense_variant	gacH	M5005_Spy0609	c.1427A>G	p.Asn476Ser	1						0
9400	G	A	A	missense_variant	gacH	M5005_Spy0609	c.1432G>A	p.Ala478Thr	61						0
9435	C	T	T	synonymous_variant	gacH	M5005_Spy0609	c.1467C>T	p.Ser489Ser	636						0
9450	T	G	G	missense_variant	gacH	M5005_Spy0609	c.1482T>G	p.Asp494Glu	22						0
9461	A	G	G	missense_variant	gacH	M5005_Spy0609	c.1493A>G	p.Asn498Ser	16						0
9463	G	A	A	missense_variant	gacH	M5005_Spy0609	c.1495G>A	p.Val499Ile	5						0
9467	C	T,A	A	missense_variant	gacH	M5005_Spy0609	c.1499C>A	p.Thr500Asn	7	T	missense_variant	gacH	c.1499C>T	p.Thr500Ile	1
9474	C	T	T	synonymous_variant	gacH	M5005_Spy0609	c.1506C>T	p.Ser502Ser	2						0
9475	C	T	T	missense_variant	gacH	M5005_Spy0609	c.1507C>T	p.His503Tyr	1						0
9477	C	T	T	synonymous_variant	gacH	M5005_Spy0609	c.1509C>T	p.His503His	170						0
9500	C	T	T	missense_variant	gacH	M5005_Spy0609	c.1532C>T	p.Ala511Val	1						0
9502	A	G	G	missense_variant	gacH	M5005_Spy0609	c.1534A>G	p.Ile512Val	1						0
9513	C	T	T	synonymous_variant	gacH	M5005_Spy0609	c.1545C>T	p.Ser515Ser	4						0
9522	G	T,A	A	synonymous_variant	gacH	M5005_Spy0609	c.1554G>A	p.Ala518Ala	40	T	synonymous_variant	gacH	c.1554G>T	p.Ala518Ala	10

9525	A	G	G	synonymous_variant	gacH	M5005_Spy0609	c.1557A>G	p.Gly519Gly	8						0
9526	C	T	T	missense_variant	gacH	M5005_Spy0609	c.1558C>T	p.Leu520Phe	27						0
9528	C	T	T	synonymous_variant	gacH	M5005_Spy0609	c.1560C>T	p.Leu520Leu	33						0
9546	C	T	T	synonymous_variant	gacH	M5005_Spy0609	c.1578C>T	p.Tyr526Tyr	2						0
9549	G	A	A	synonymous_variant	gacH	M5005_Spy0609	c.1581G>A	p.Gly527Gly	1						0
9558	G	T	T	synonymous_variant	gacH	M5005_Spy0609	c.1590G>T	p.Thr530Thr	1117						0
9579	G	A	A	synonymous_variant	gacH	M5005_Spy0609	c.1611G>A	p.Thr537Thr	6						0
9586	A	G	G	missense_variant	gacH	M5005_Spy0609	c.1618A>G	p.Ser540Gly	1						0
9589	T	G	G	missense_variant	gacH	M5005_Spy0609	c.1621T>G	p.Leu541Val	36						0
9592	C	T	T	missense_variant	gacH	M5005_Spy0609	c.1624C>T	p.Pro542Ser	5						0
9594	T	C	C	synonymous_variant	gacH	M5005_Spy0609	c.1626T>C	p.Pro542Pro	1						0
9609	T	G	G	synonymous_variant	gacH	M5005_Spy0609	c.1641T>G	p.Ser547Ser	1						0
9614	C	T	T	missense_variant	gacH	M5005_Spy0609	c.1646C>T	p.Ser549Leu	1						0
9618	A	G	G	synonymous_variant	gacH	M5005_Spy0609	c.1650A>G	p.Val550Val	3						0
9624	T	C	C	synonymous_variant	gacH	M5005_Spy0609	c.1656T>C	p.Val552Val	79						0
9639	C	T	T	synonymous_variant	gacH	M5005_Spy0609	c.1671C>T	p.Val557Val	2						0
9645	T	C	C	synonymous_variant	gacH	M5005_Spy0609	c.1677T>C	p.Pro559Pro	116						0
9648	A	C	C	missense_variant	gacH	M5005_Spy0609	c.1680A>C	p.Lys560Asn	7						0
9654	C	T	T	synonymous_variant	gacH	M5005_Spy0609	c.1686C>T	p.Ala562Ala	10						0
9659	C	A	A	missense_variant	gacH	M5005_Spy0609	c.1691C>A	p.Pro564His	1						0
9712	G	T	T	missense_variant	gacH	M5005_Spy0609	c.1744G>T	p.Ala582Ser	1						0
9723	C	T	T	synonymous_variant	gacH	M5005_Spy0609	c.1755C>T	p.Asn585Asn	4						0
9765	C	A,T	A	synonymous_variant	gacH	M5005_Spy0609	c.1797C>A	p.Ser599Ser	103	T	synonymous_variant	gacH	c.1797C>T	p.Ser599Ser	2
9787	T	G	G	missense_variant	gacH	M5005_Spy0609	c.1819T>G	p.Ser607Ala	1						0
9807	C	T	T	synonymous_variant	gacH	M5005_Spy0609	c.1839C>T	p.Asn613Asn	2						0
9819	C	T	T	synonymous_variant	gacH	M5005_Spy0609	c.1851C>T	p.Val617Val	10						0
9822	T	C	C	synonymous_variant	gacH	M5005_Spy0609	c.1854T>C	p.Gly618Gly	9						0
9834	C	T	T	synonymous_variant	gacH	M5005_Spy0609	c.1866C>T	p.Ser622Ser	48						0
9878	G	T	T	missense_variant	gacH	M5005_Spy0609	c.1910G>T	p.Ser637Ile	1						0
9879	T	C	C	synonymous_variant	gacH	M5005_Spy0609	c.1911T>C	p.Ser637Ser	76						0
9880	G	C	C	missense_variant	gacH	M5005_Spy0609	c.1912G>C	p.Glu638Gln	4						0
9885	C	T	T	synonymous_variant	gacH	M5005_Spy0609	c.1917C>T	p.Ser639Ser	2						0
9894	C	T	T	synonymous_variant	gacH	M5005_Spy0609	c.1926C>T	p.Phe642Phe	4						0
9942	T	C	C	synonymous_variant	gacH	M5005_Spy0609	c.1974T>C	p.Pro658Pro	3						0
9945	A	G	G	synonymous_variant	gacH	M5005_Spy0609	c.1977A>G	p.Glu659Glu	2						0
9952	G	A	A	missense_variant	gacH	M5005_Spy0609	c.1984G>A	p.Val662Ile	43						0
9975	G	A	A	synonymous_variant	gacH	M5005_Spy0609	c.2007G>A	p.Thr669Thr	18						0
9996	A	T	T	synonymous_variant	gacH	M5005_Spy0609	c.2028A>T	p.Leu676Leu	1						0
10011	G	A	A	synonymous_variant	gacH	M5005_Spy0609	c.2043G>A	p.Arg681Arg	4						0
10020	G	A	A	synonymous_variant	gacH	M5005_Spy0609	c.2052G>A	p.Ser684Ser	2						0
10033	G	A	A	missense_variant	gacH	M5005_Spy0609	c.2065G>A	p.Glu689Lys	17						0
10040	G	A	A	missense_variant	gacH	M5005_Spy0609	c.2072G>A	p.Arg691Lys	37						0
10043	C	A	A	missense_variant	gacH	M5005_Spy0609	c.2075C>A	p.Ala692Glu	1						0
10048	T	C	C	synonymous_variant	gacH	M5005_Spy0609	c.2080T>C	p.Leu694Leu	22						0
10061	C	T	T	missense_variant	gacH	M5005_Spy0609	c.2093C>T	p.Thr698Ile	1						0
10062	A	C	C	synonymous_variant	gacH	M5005_Spy0609	c.2094A>C	p.Thr698Thr	1						0
10071	C	T	T	synonymous_variant	gacH	M5005_Spy0609	c.2103C>T	p.Asn701Asn	9						0
10074	G	A	A	synonymous_variant	gacH	M5005_Spy0609	c.2106G>A	p.Lys702Lys	18						0
10075	C	A	A	missense_variant	gacH	M5005_Spy0609	c.2107C>A	p.Pro703Thr	3						0
10083	A	G	G	synonymous_variant	gacH	M5005_Spy0609	c.2115A>G	p.Thr705Thr	1						0
10085	T	C	C	missense_variant	gacH	M5005_Spy0609	c.2117T>C	p.Val706Ala	18						0
10089	G	A,T	A	synonymous_variant	gacH	M5005_Spy0609	c.2121G>A	p.Val707Val	3	T	synonymous_variant	gacH	c.2121G>T	p.Val707Val	2
10113	T	C	C	synonymous_variant	gacH	M5005_Spy0609	c.2145T>C	p.Gly715Gly	2						0
10137	C	T	T	synonymous_variant	gacH	M5005_Spy0609	c.2169C>T	p.Asn723Asn	1						0
10181	G	A	A	missense_variant	gacH	M5005_Spy0609	c.2213G>A	p.Ser738Asn	2						0
10199	A	G	G	missense_variant	gacH	M5005_Spy0609	c.2231A>G	p.Lys744Arg	289						0
10226	G	A	A	missense_variant	gacH	M5005_Spy0609	c.2258G>A	p.Ser753Asn	26						0
10233	T	C	C	synonymous_variant	gacH	M5005_Spy0609	c.2265T>C	p.Phe755Phe	1						0
10245	A	G	G	synonymous_variant	gacH	M5005_Spy0609	c.2277A>G	p.Leu759Leu	20						0
10269	A	G	G	synonymous_variant	gacH	M5005_Spy0609	c.2301A>G	p.Val767Val	4						0
10272	A	G	G	synonymous_variant	gacH	M5005_Spy0609	c.2304A>G	p.Ser768Ser	6						0
10314	T	C	C	synonymous_variant	gacH	M5005_Spy0609	c.2346T>C	p.Ser782Ser	11						0

10315	G	A	A	missense_variant	gacH	M5005_Spy0609	c.2347G>A	p.Val783Ile	1						0
10317	C	T	T	synonymous_variant	gacH	M5005_Spy0609	c.2349C>T	p.Val783Val	8						0
10328	C	T	T	missense_variant	gacH	M5005_Spy0609	c.2360C>T	p.Pro787Leu	22						0
10335	T	C	C	synonymous_variant	gacH	M5005_Spy0609	c.2367T>C	p.Ser789Ser	1						0
10336	C	T	T	missense_variant	gacH	M5005_Spy0609	c.2368C>T	p.Pro790Ser	4						0
10339	G	A	A	missense_variant	gacH	M5005_Spy0609	c.2371G>A	p.Glu791Lys	7						0
10349	C	T	T	missense_variant	gacH	M5005_Spy0609	c.2381C>T	p.Ala794Val	1						0
10357	A	G	G	missense_variant	gacH	M5005_Spy0609	c.2389A>G	p.Asn797Asp	19						0
10363	T	C	C	synonymous_variant	gacH	M5005_Spy0609	c.2395T>C	p.Leu799Leu	25						0
10372	A	G	G	missense_variant	gacH	M5005_Spy0609	c.2404A>G	p.Ile802Val	92						0
10373	T	C	C	missense_variant	gacH	M5005_Spy0609	c.2405T>C	p.Ile802Thr	1						0
10377	A	G	G	synonymous_variant	gacH	M5005_Spy0609	c.2409A>G	p.Gln803Gln	1						0
10435	T	C	C	missense_variant	gacH	M5005_Spy0609	c.2467T>C	p.Ser823Pro	31						0
10455	G	T	T	upstream_gene_variant	intergenic				6						0
10456	G	A	A	upstream_gene_variant	intergenic				1						0
10459	G	A	A	upstream_gene_variant	intergenic				4						0
10463	G	A	A	upstream_gene_variant	intergenic				32						0
10465	G	A,T	A	upstream_gene_variant	intergenic				4	T	upstream_gene_variant	intergenic	c.-157G>T		20
10471	G	A	A	upstream_gene_variant	intergenic				1225						0
10473	A	G	G	upstream_gene_variant	intergenic				1218						0
10487	G	T	T	upstream_gene_variant	intergenic				2						0
10498	C	T	T	upstream_gene_variant	intergenic				5						0
10500	A	G	G	upstream_gene_variant	intergenic				23						0
10504	A	G	G	upstream_gene_variant	intergenic				1						0
10514	G	A	A	upstream_gene_variant	intergenic				6						0
10518	G	A	A	upstream_gene_variant	intergenic				384						0
10525	A	G	G	upstream_gene_variant	intergenic				348						0
10526	C	T	T	upstream_gene_variant	intergenic				4						0
10528	A	C	C	upstream_gene_variant	intergenic				4						0
10529	G	A	A	upstream_gene_variant	intergenic				33						0
10530	G	A	A	upstream_gene_variant	intergenic				7						0
10539	A	T	T	upstream_gene_variant	intergenic				24						0
10540	T	C	C	upstream_gene_variant	intergenic				33						0
10542	A	G	G	upstream_gene_variant	intergenic				1						0
10551	A	G	G	upstream_gene_variant	intergenic				71						0
10554	G	A	A	upstream_gene_variant	intergenic				2						0
10562	C	T,A	A	upstream_gene_variant	intergenic				6	T	upstream_gene_variant	intergenic	c.-60C>T		3
10563	G	A	A	upstream_gene_variant	intergenic				1						0
10564	T	C	C	upstream_gene_variant	intergenic				11						0
10570	A	G	G	upstream_gene_variant	intergenic				2						0
10583	C	T,A	A	upstream_gene_variant	intergenic				1	T	upstream_gene_variant	intergenic	c.-39C>T		37
10601	A	C	C	upstream_gene_variant	intergenic				44						0
10663	T	G,C	G	missense_variant	gacI	M5005_Spy0610	c.42T>G	p.Ser14Arg	33	C	synonymous_variant	gacI	c.42T>C	p.Ser14Ser	5
10666	T	C	C	synonymous_variant	gacI	M5005_Spy0610	c.45T>C	p.Asn15Asn	4						0
10670	G	A	A	missense_variant	gacI	M5005_Spy0610	c.49G>A	p.Val17Ile	12						0
10682	C	A,T	A	missense_variant	gacI	M5005_Spy0610	c.61C>A	p.Arg21Ser	3	T	missense_variant	gacI	c.61C>T	p.Arg21Cys	1
10695	C	T	T	missense_variant	gacI	M5005_Spy0610	c.74C>T	p.Ser25Leu	15						0
10702	C	T	T	synonymous_variant	gacI	M5005_Spy0610	c.81C>T	p.Ala27Ala	14						0
10703	C	T	T	missense_variant	gacI	M5005_Spy0610	c.82C>T	p.Pro28Ser	109						0
10741	G	A	A	synonymous_variant	gacI	M5005_Spy0610	c.120G>A	p.Thr40Thr	6						0
10750	G	A	A	synonymous_variant	gacI	M5005_Spy0610	c.129G>A	p.Thr43Thr	25						0
10754	G	A	A	missense_variant	gacI	M5005_Spy0610	c.133G>A	p.Ala45Thr	16						0
10765	A	G	G	synonymous_variant	gacI	M5005_Spy0610	c.144A>G	p.Gln48Gln	3						0
10766	A	G	G	missense_variant	gacI	M5005_Spy0610	c.145A>G	p.Lys49Glu	144						0
10768	A	C	C	missense_variant	gacI	M5005_Spy0610	c.147A>C	p.Lys49Asn	26						0
10777	C	T,G	G	missense_variant	gacI	M5005_Spy0610	c.156C>G	p.Phe52Leu	24	T	synonymous_variant	gacI	c.156C>T	p.Phe52Phe	3
10783	T	G	G	synonymous_variant	gacI	M5005_Spy0610	c.162T>G	p.Val54Val	29						0
10796	A	G	G	missense_variant	gacI	M5005_Spy0610	c.175A>G	p.Ile59Val	1						0
10802	C	T	T	synonymous_variant	gacI	M5005_Spy0610	c.181C>T	p.Leu61Leu	106						0
10821	T	A	A	missense_variant	gacI	M5005_Spy0610	c.200T>A	p.Val67Glu	1						0
10836	G	A	A	missense_variant	gacI	M5005_Spy0610	c.215G>A	p.Arg72His	49						0
10848	G	T	T	missense_variant	gacI	M5005_Spy0610	c.227G>T	p.Arg76Ile	3						0

10861	C	T	T	synonymous_variant	gacI	M5005_Spy0610	c.240C>T	p.Asp80Asp	28						0
10868	G	A	A	missense_variant	gacI	M5005_Spy0610	c.247G>A	p.Val83Ile	1						0
10891	G	A	A	synonymous_variant	gacI	M5005_Spy0610	c.270G>A	p.Gln90Gln	33						0
10903	C	T	T	synonymous_variant	gacI	M5005_Spy0610	c.282C>T	p.Cys94Cys	322						0
10911	A	G,T	G	missense_variant	gacI	M5005_Spy0610	c.290A>G	p.Glu97Gly	40	T	missense_variant	gacI	c.290A>T	p.Glu97Val	3
10912	A	G	G	synonymous_variant	gacI	M5005_Spy0610	c.291A>G	p.Glu97Glu	1						0
10914	A	C	C	missense_variant	gacI	M5005_Spy0610	c.293A>C	p.Lys98Thr	10						0
10924	G	A	A	synonymous_variant	gacI	M5005_Spy0610	c.303G>A	p.Glu101Glu	55						0
10966	A	T,G	G	synonymous_variant	gacI	M5005_Spy0610	c.345A>G	p.Arg115Arg	63	T	synonymous_variant	gacI	c.345A>T	p.Arg115Arg	1
10978	A	G	G	synonymous_variant	gacI	M5005_Spy0610	c.357A>G	p.Lys119Lys	23						0
10993	A	G	G	synonymous_variant	gacI	M5005_Spy0610	c.372A>G	p.Ser124Ser	3						0
11008	T	C	C	synonymous_variant	gacI	M5005_Spy0610	c.387T>C	p.Arg129Arg	1						0
11011	C	T	T	synonymous_variant	gacI	M5005_Spy0610	c.390C>T	p.Ile130Ile	1						0
11014	C	T	T	synonymous_variant	gacI	M5005_Spy0610	c.393C>T	p.Gly131Gly	716						0
11027	A	G	G	missense_variant	gacI	M5005_Spy0610	c.406A>G	p.Thr136Ala	21						0
11029	T	A,*	A	synonymous_variant	gacI	M5005_Spy0610	c.408T>A	p.Thr136Thr	125						1
11041	G	A	A	synonymous_variant	gacI	M5005_Spy0610	c.420G>A	p.Ala140Ala	1						0
11052	G	A	A	missense_variant	gacI	M5005_Spy0610	c.431G>A	p.Gly144Glu	120						0
11074	G	T	T	synonymous_variant	gacI	M5005_Spy0610	c.453G>T	p.Thr151Thr	1						0
11080	T	G	G	synonymous_variant	gacI	M5005_Spy0610	c.459T>G	p.Gly153Gly	1						0
11081	C	T	T	synonymous_variant	gacI	M5005_Spy0610	c.460C>T	p.Leu154Leu	8						0
11086	G	A	A	synonymous_variant	gacI	M5005_Spy0610	c.465G>A	p.Arg155Arg	18						0
11091	T	C	C	missense_variant	gacI	M5005_Spy0610	c.470T>C	p.Ile157Thr	11						0
11095	C	T	T	synonymous_variant	gacI	M5005_Spy0610	c.474C>T	p.Asp158Asp	29						0
11100	C	T	T	missense_variant	gacI	M5005_Spy0610	c.479C>T	p.Ser160Leu	1						0
11110	A	C	C	missense_variant	gacI	M5005_Spy0610	c.489A>C	p.Glu163Asp	1						0
11123	C	T	T	missense_variant	gacI	M5005_Spy0610	c.502C>T	p.His168Tyr	29						0
11125	T	G	G	missense_variant	gacI	M5005_Spy0610	c.504T>G	p.His168Gln	2						0
11129	C	T	T	missense_variant	gacI	M5005_Spy0610	c.508C>T	p.Pro170Ser	20						0
11161	T	C	C	synonymous_variant	gacI	M5005_Spy0610	c.540T>C	p.Val180Val	1						0
11165	G	A	A	missense_variant	gacI	M5005_Spy0610	c.544G>A	p.Val182Ile	1						0
11175	G	A	A	missense_variant	gacI	M5005_Spy0610	c.554G>A	p.Ser185Asn	17						0
11196	T	C	C	missense_variant	gacI	M5005_Spy0610	c.575T>C	p.Ile192Thr	38						0
11224	C	T,G	G	synonymous_variant	gacI	M5005_Spy0610	c.603C>G	p.Gly201Gly	25	T	synonymous_variant	gacI	c.603C>T	p.Gly201Gly	1
11233	A	G	G	synonymous_variant	gacI	M5005_Spy0610	c.612A>G	p.Ser204Ser	38						0
11242	G	A	A	synonymous_variant	gacI	M5005_Spy0610	c.621G>A	p.Ser207Ser	261						0
11247	C	T	T	missense_variant	gacI	M5005_Spy0610	c.626C>T	p.Thr209Met	17						0
11272	A	G	G	synonymous_variant	gacI	M5005_Spy0610	c.651A>G	p.Lys217Lys	2						0
11294	G	T	T	missense_variant	gacI	M5005_Spy0610	c.673G>T	p.Val225Phe	3						0
11298	G	A	A	missense_variant	gacI	M5005_Spy0610	c.677G>A	p.Arg226Lys	2						0
11299	A	G	G	synonymous_variant	gacI	M5005_Spy0610	c.678A>G	p.Arg226Arg	90						0
11310	A	G	G	missense_variant	gacI	M5005_Spy0610	c.689A>G	p.Asn230Ser	2						0
11311	T	A	A	missense_variant	gacI	M5005_Spy0610	c.690T>A	p.Asn230Lys	2						0
11360	A	C	C	missense_variant	gacJ	M5005_Spy0611	c.42A>C	p.Leu14Phe	1						0
11362	C	T	T	missense_variant	gacJ	M5005_Spy0611	c.44C>T	p.Thr15Ile	6						0
11367	G	A	A	missense_variant	gacJ	M5005_Spy0611	c.49G>A	p.Val17Ile	17						0
11387	A	G	G	synonymous_variant	gacJ	M5005_Spy0611	c.69A>G	p.Leu23Leu	9						0
11396	A	G	G	synonymous_variant	gacJ	M5005_Spy0611	c.78A>G	p.Lys26Lys	1						0
11399	T	C	C	synonymous_variant	gacJ	M5005_Spy0611	c.81T>C	p.Asp27Asp	1778						0
11426	G	A	A	synonymous_variant	gacJ	M5005_Spy0611	c.108G>A	p.Leu36Leu	125						0
11453	A	G	G	synonymous_variant	gacJ	M5005_Spy0611	c.135A>G	p.Leu45Leu	22						0
11469	C	T	T	missense_variant	gacJ	M5005_Spy0611	c.151C>T	p.Pro51Ser	6						0
11472	A	G	G	missense_variant	gacJ	M5005_Spy0611	c.154A>G	p.Asn52Asp	20						0
11478	T	C	C	synonymous_variant	gacJ	M5005_Spy0611	c.160T>C	p.Leu54Leu	1						0
11480	A	G	G	synonymous_variant	gacJ	M5005_Spy0611	c.162A>G	p.Leu54Leu	2						0
11482	A	G	G	missense_variant	gacJ	M5005_Spy0611	c.164A>G	p.Asn55Ser	16						0
11483	T	A	A	missense_variant	gacJ	M5005_Spy0611	c.165T>A	p.Asn55Lys	2						0
11484	A	G	G	missense_variant	gacJ	M5005_Spy0611	c.166A>G	p.Lys56Glu	14						0
11487	A	T	T	missense_variant	gacJ	M5005_Spy0611	c.169A>T	p.Met57Leu	6						0
11498	C	T	T	synonymous_variant	gacJ	M5005_Spy0611	c.180C>T	p.Phe60Phe	115						0
11507	T	A,C	A	synonymous_variant	gacJ	M5005_Spy0611	c.189T>A	p.Ile63Ile	3	C	synonymous_variant	gacJ	c.189T>C	p.Ile63Ile	17
11514	C	T	T	missense_variant	gacJ	M5005_Spy0611	c.196C>T	p.Pro66Ser	4						0

11526	T	C	C	synonymous_variant	gacJ	M5005_Spy0611	c.208T>C	p.Leu70Leu	92						0
11552	T	C	C	synonymous_variant	gacJ	M5005_Spy0611	c.234T>C	p.Ser78Ser	1						0
11567	T	C	C	synonymous_variant	gacJ	M5005_Spy0611	c.249T>C	p.Phe83Phe	1						0
11571	C	T	T	synonymous_variant	gacJ	M5005_Spy0611	c.253C>T	p.Leu85Leu	1						0
11585	T	C	C	synonymous_variant	gacJ	M5005_Spy0611	c.267T>C	p.Ile89Ile	727						0
11610	C	T	T	missense_variant	gacJ	M5005_Spy0611	c.292C>T	p.Arg98Cys	10						0
11618	C	T	T	synonymous_variant	gacJ	M5005_Spy0611	c.300C>T	p.Thr100Thr	2						0
11624	A	G	G	synonymous_variant	gacJ	M5005_Spy0611	c.306A>G	p.Glu102Glu	39						0
11636	G	T	T	missense_variant	gacJ	M5005_Spy0611	c.318G>T	p.Met106Ile	3						0
11650	C	A	A	missense_variant	gacJ	M5005_Spy0611	c.332C>A	p.Thr111Asn	1						0
11661	T	G	G	missense_variant	gacK	M5005_Spy0612	c.9T>G	p.Asn3Lys	4						0
11663	C	T	T	missense_variant	gacK	M5005_Spy0612	c.11C>T	p.Pro4Leu	4						0
11664	T	C	C	synonymous_variant	gacK	M5005_Spy0612	c.12T>C	p.Pro4Pro	30						0
11666	C	T	T	missense_variant	gacK	M5005_Spy0612	c.14C>T	p.Ser5Leu	51						0
11681	C	T	T	missense_variant	gacK	M5005_Spy0612	c.29C>T	p.Thr10Met	1						0
11682	G	A	A	synonymous_variant	gacK	M5005_Spy0612	c.30G>A	p.Thr10Thr	2						0
11691	A	G	G	synonymous_variant	gacK	M5005_Spy0612	c.39A>G	p.Leu13Leu	3						0
11699	T	C	C	missense_variant	gacK	M5005_Spy0612	c.47T>C	p.Met16Thr	33						0
11711	T	C	C	missense_variant	gacK	M5005_Spy0612	c.59T>C	p.Leu20Ser	6						0
11721	T	C	C	synonymous_variant	gacK	M5005_Spy0612	c.69T>C	p.Ala23Ala	1						0
11767	T	C	C	missense_variant	gacK	M5005_Spy0612	c.115T>C	p.Ser39Pro	22						0
11769	G	T	T	synonymous_variant	gacK	M5005_Spy0612	c.117G>T	p.Ser39Ser	1						0
11770	G	T	T	missense_variant	gacK	M5005_Spy0612	c.118G>T	p.Ala40Ser	181						0
11790	C	T	T	synonymous_variant	gacK	M5005_Spy0612	c.138C>T	p.Ala46Ala	30						0
11863	A	G	G	missense_variant	gacK	M5005_Spy0612	c.211A>G	p.Ile71Val	1						0
11865	T	G	G	missense_variant	gacK	M5005_Spy0612	c.213T>G	p.Ile71Met	5						0
11886	T	C	C	synonymous_variant	gacK	M5005_Spy0612	c.234T>C	p.Ser78Ser	1						0
11889	G	A	A	synonymous_variant	gacK	M5005_Spy0612	c.237G>A	p.Gln79Gln	12						0
11898	G	A	A	synonymous_variant	gacK	M5005_Spy0612	c.246G>A	p.Val82Val	107						0
11899	G	A	A	missense_variant	gacK	M5005_Spy0612	c.247G>A	p.Ala83Thr	14						0
11901	A	G	G	synonymous_variant	gacK	M5005_Spy0612	c.249A>G	p.Ala83Ala	16						0
11916	T	C	C	synonymous_variant	gacK	M5005_Spy0612	c.264T>C	p.Cys88Cys	1						0
11931	C	T	T	synonymous_variant	gacK	M5005_Spy0612	c.279C>T	p.Ala93Ala	67						0
11934	A	G	G	missense_variant	gacK	M5005_Spy0612	c.282A>G	p.Ile94Met	3						0
11950	A	G	G	missense_variant	gacK	M5005_Spy0612	c.298A>G	p.Thr100Ala	1						0
11984	T	C	C	missense_variant	gacK	M5005_Spy0612	c.332T>C	p.Ile111Thr	22						0
11992	C	T	T	synonymous_variant	gacK	M5005_Spy0612	c.340C>T	p.Leu114Leu	1						0
12008	G	A	A	missense_variant	gacK	M5005_Spy0612	c.356G>A	p.Arg119Lys	1						0
12039	G	A	A	synonymous_variant	gacK	M5005_Spy0612	c.387G>A	p.Gln129Gln	1						0
12048	C	T	T	synonymous_variant	gacK	M5005_Spy0612	c.396C>T	p.Phe132Phe	1						0
12055	C	T	T	missense_variant	gacK	M5005_Spy0612	c.403C>T	p.His135Tyr	2						0
12063	G	A	A	synonymous_variant	gacK	M5005_Spy0612	c.411G>A	p.Arg137Arg	88						0
12075	A	G	G	synonymous_variant	gacK	M5005_Spy0612	c.423A>G	p.Ala141Ala	1						0
12090	C	T	T	synonymous_variant	gacK	M5005_Spy0612	c.438C>T	p.Ala146Ala	93						0
12100	A	G	G	missense_variant	gacK	M5005_Spy0612	c.448A>G	p.Thr150Ala	8						0
12102	T	C	C	synonymous_variant	gacK	M5005_Spy0612	c.450T>C	p.Thr150Thr	3						0
12114	G	A	A	missense_variant	gacK	M5005_Spy0612	c.462G>A	p.Met154Ile	10						0
12115	G	A	A	missense_variant	gacK	M5005_Spy0612	c.463G>A	p.Val155Met	14						0
12117	G	T	T	synonymous_variant	gacK	M5005_Spy0612	c.465G>T	p.Val155Val	2						0
12130	A	T	T	missense_variant	gacK	M5005_Spy0612	c.478A>T	p.Ile160Leu	5						0
12133	T	C	C	synonymous_variant	gacK	M5005_Spy0612	c.481T>C	p.Leu161Leu	1						0
12135	A	G	G	synonymous_variant	gacK	M5005_Spy0612	c.483A>G	p.Leu161Leu	1						0
12140	C	A	A	missense_variant	gacK	M5005_Spy0612	c.488C>A	p.Ser163Tyr	17						0
12144	A	G	G	synonymous_variant	gacK	M5005_Spy0612	c.492A>G	p.Lys164Lys	10						0
12150	T	C	C	synonymous_variant	gacK	M5005_Spy0612	c.498T>C	p.Leu166Leu	6						0
12164	T	C	C	missense_variant	gacK	M5005_Spy0612	c.512T>C	p.Val171Ala	1						0
12169	G	A	A	missense_variant	gacK	M5005_Spy0612	c.517G>A	p.Val173Ile	2						0
12170	T	C	C	missense_variant	gacK	M5005_Spy0612	c.518T>C	p.Val173Ala	27						0
12172	T	G	G	missense_variant	gacK	M5005_Spy0612	c.520T>G	p.Cys174Gly	4						0
12197	T	C	C	missense_variant	gacK	M5005_Spy0612	c.545T>C	p.Met182Thr	4						0
12207	C	T	T	synonymous_variant	gacK	M5005_Spy0612	c.555C>T	p.Asp185Asp	4						0
12213	T	C	C	synonymous_variant	gacK	M5005_Spy0612	c.561T>C	p.Gly187Gly	85						0

12214	C	T	T	missense_variant	gacK	M5005_Spy0612	c.562C>T	p.His188Tyr	203						0
12244	A	G	G	missense_variant	gacK	M5005_Spy0612	c.592A>G	p.Ser198Gly	2						0
12257	G	A	A	missense_variant	gacK	M5005_Spy0612	c.605G>A	p.Ser202Asn	6						0
12259	A	G	G	missense_variant	gacK	M5005_Spy0612	c.607A>G	p.Asn203Asp	6						0
12270	T	C	C	synonymous_variant	gacK	M5005_Spy0612	c.618T>C	p.Phe206Phe	110						0
12306	A	G	G	synonymous_variant	gacK	M5005_Spy0612	c.654A>G	p.Pro218Pro	58						0
12311	T	C	C	missense_variant	gacK	M5005_Spy0612	c.659T>C	p.Phe220Ser	1						0
12359	C	T	T	missense_variant	gacK	M5005_Spy0612	c.707C>T	p.Ala236Val	3						0
12367	C	G	G	missense_variant	gacK	M5005_Spy0612	c.715C>G	p.Leu239Val	1						0
12374	C	T	T	missense_variant	gacK	M5005_Spy0612	c.722C>T	p.Thr241Met	50						0
12377	C	T	T	missense_variant	gacK	M5005_Spy0612	c.725C>T	p.Thr242Ile	2						0
12383	G	A	A	missense_variant	gacK	M5005_Spy0612	c.731G>A	p.Gly244Asp	1						0
12388	G	A	A	missense_variant	gacK	M5005_Spy0612	c.736G>A	p.Val246Ile	501						0
12392	C	T	T	missense_variant	gacK	M5005_Spy0612	c.740C>T	p.Ala247Val	9						0
12396	G	A	A	synonymous_variant	gacK	M5005_Spy0612	c.744G>A	p.Leu248Leu	3						0
12399	C	T	T	synonymous_variant	gacK	M5005_Spy0612	c.747C>T	p.Gly249Gly	4						0
12435	C	A	A	synonymous_variant	gacK	M5005_Spy0612	c.783C>A	p.Ala261Ala	2						0
12450	G	A	A	synonymous_variant	gacK	M5005_Spy0612	c.798G>A	p.Leu266Leu	1						0
12453	A	C	C	synonymous_variant	gacK	M5005_Spy0612	c.801A>C	p.Leu267Leu	22						0
12472	C	T	T	missense_variant	gacK	M5005_Spy0612	c.820C>T	p.His274Tyr	782						0
12494	C	T	T	missense_variant	gacK	M5005_Spy0612	c.842C>T	p.Ala281Val	4						0
12501	C	T	T	synonymous_variant	gacK	M5005_Spy0612	c.849C>T	p.Ile283Ile	2						0
12511	A	G	G	missense_variant	gacK	M5005_Spy0612	c.859A>G	p.Ile287Val	5						0
12547	G	A	A	missense_variant	gacK	M5005_Spy0612	c.895G>A	p.Ala299Thr	5						0
12570	G	A	A	synonymous_variant	gacK	M5005_Spy0612	c.918G>A	p.Leu306Leu	1						0
12575	C	T	T	missense_variant	gacK	M5005_Spy0612	c.923C>T	p.Ala308Val	64						0
12592	T	C	C	synonymous_variant	gacK	M5005_Spy0612	c.940T>C	p.Leu314Leu	5						0
12604	C	A	A	missense_variant	gacK	M5005_Spy0612	c.952C>A	p.Pro318Thr	7						0
12605	C	T	T	missense_variant	gacK	M5005_Spy0612	c.953C>T	p.Pro318Leu	2						0
12618	A	G	G	missense_variant	gacK	M5005_Spy0612	c.966A>G	p.Ile322Met	1188						0
12619	C	T	T	synonymous_variant	gacK	M5005_Spy0612	c.967C>T	p.Leu323Leu	4						0
12627	T	C	C	synonymous_variant	gacK	M5005_Spy0612	c.975T>C	p.Gly325Gly	34						0
12633	C	A	A	missense_variant	gacK	M5005_Spy0612	c.981C>A	p.Asn327Lys	2						0
12639	G	A	A	synonymous_variant	gacK	M5005_Spy0612	c.987G>A	p.Thr329Thr	14						0
12654	T	C	C	synonymous_variant	gacK	M5005_Spy0612	c.1002T>C	p.Asp334Asp	1287						0
12670	C	T	T	synonymous_variant	gacK	M5005_Spy0612	c.1018C>T	p.Leu340Leu	4						0
12681	G	A	A	synonymous_variant	gacK	M5005_Spy0612	c.1029G>A	p.Ser343Ser	5						0
12689	G	T	T	missense_variant	gacK	M5005_Spy0612	c.1037G>T	p.Ser346Ile	1						0
12695	C	T	T	missense_variant	gacK	M5005_Spy0612	c.1043C>T	p.Ala348Val	39						0
12719	C	T	T	missense_variant	gacK	M5005_Spy0612	c.1067C>T	p.Thr356Ile	1						0
12730	A	C	C	missense_variant	gacK	M5005_Spy0612	c.1078A>C	p.Lys360Gln	22						0
12731	A	C	C	missense_variant	gacK	M5005_Spy0612	c.1079A>C	p.Lys360Thr	33						0
12745	C	A	A	missense_variant	gacK	M5005_Spy0612	c.1093C>A	p.Leu365Ile	2						0
12753	A	G	G	synonymous_variant	gacK	M5005_Spy0612	c.1101A>G	p.Pro367Pro	33						0
12762	A	G	G	synonymous_variant	gacK	M5005_Spy0612	c.1110A>G	p.Gly370Gly	9						0
12765	A	G	G	synonymous_variant	gacK	M5005_Spy0612	c.1113A>G	p.Gly371Gly	12						0
12777	G	A	A	synonymous_variant	gacK	M5005_Spy0612	c.1125G>A	p.Ser375Ser	4						0
12784	A	G	G	missense_variant	gacK	M5005_Spy0612	c.1132A>G	p.Ile378Val	5						0
12786	T	C	C	synonymous_variant	gacK	M5005_Spy0612	c.1134T>C	p.Ile378Ile	3						0
12787	A	G	G	missense_variant	gacK	M5005_Spy0612	c.1135A>G	p.Thr379Ala	6						0
12793	C	T	T	missense_variant	gacK	M5005_Spy0612	c.1141C>T	p.Leu381Phe	1						0
12803	T	C	C	missense_variant	gacK	M5005_Spy0612	c.1151T>C	p.Met384Thr	33						0
12809	A	C	C	missense_variant	gacK	M5005_Spy0612	c.1157A>C	p.Tyr386Ser	1						0
12813	T	C	C	synonymous_variant	gacK	M5005_Spy0612	c.1161T>C	p.His387His	1445						0
12823	G	T	T	missense_variant	gacK	M5005_Spy0612	c.1171G>T	p.Ala391Ser	3						0
12831	G	A	A	synonymous_variant	gacK	M5005_Spy0612	c.1179G>A	p.Leu393Leu	1						0
12859	T	C	C	synonymous_variant	gacK	M5005_Spy0612	c.1207T>C	p.Leu403Leu	18						0
12873	T	C	C	synonymous_variant	gacK	M5005_Spy0612	c.1221T>C	p.Ile407Ile	17						0
12909	G	A	A	synonymous_variant	gacK	M5005_Spy0612	c.1257G>A	p.Lys419Lys	19						0
12912	A	G	G	synonymous_variant	gacK	M5005_Spy0612	c.1260A>G	p.Gly420Gly	143						0
12927	A	G	G	synonymous_variant	gacK_overlap	M5005_Spy0613			4		missense_variant	gacL_overlap	c.8A>G	p.Gln3Arg	0
12930	C	T	T	synonymous_variant	gacK_overlap	M5005_Spy0613			5		missense_variant	gacL_overlap	c.11C>T	p.Ser4Phe	0

12932	A	G	G	missense_variant	gacK_overlap	M5005_Spy0612	c.1280A>G	p.Tyr427Cys	9		missense_variant	gacL_overlap		p.Met5Val	0
12934	G	A	A	missense_variant	gacK_overlap	M5005_Spy0612	c.1282G>A	p.Asp428Asn	5		missense_variant	gacL_overlap		p.Met5Ile	0
12938	A	G	G	synonymous_variant	gacK_overlap	M5005_Spy0613			1		missense_variant	gacL_overlap	c.19A>G	p.Lys7Glu	0
12950	C	T	T	missense_variant	gacL	M5005_Spy0613	c.31C>T	p.Leu11Phe	4						0
12991	G	T	T	missense_variant	gacL	M5005_Spy0613	c.72G>T	p.Leu24Phe	20						0
12998	C	T	T	missense_variant	gacL	M5005_Spy0613	c.79C>T	p.Leu27Phe	581						0
12999	T	C	C	missense_variant	gacL	M5005_Spy0613	c.80T>C	p.Leu27Pro	5						0
13001	C	T,A	A	missense_variant	gacL	M5005_Spy0613	c.82C>A	p.Leu28Met	6	T	synonymous_variant	gacL	c.82C>T	p.Leu28Leu	17
13019	G	A	A	missense_variant	gacL	M5005_Spy0613	c.100G>A	p.Ala34Thr	5						0
13020	C	T	T	missense_variant	gacL	M5005_Spy0613	c.101C>T	p.Ala34Val	7						0
13021	C	T	T	synonymous_variant	gacL	M5005_Spy0613	c.102C>T	p.Ala34Ala	7						0
13032	A	G	G	missense_variant	gacL	M5005_Spy0613	c.113A>G	p.Asn38Ser	11						0
13036	A	G	G	synonymous_variant	gacL	M5005_Spy0613	c.117A>G	p.Leu39Leu	6						0
13067	G	A	A	missense_variant	gacL	M5005_Spy0613	c.148G>A	p.Val50Met	22						0
13072	C	T	T	synonymous_variant	gacL	M5005_Spy0613	c.153C>T	p.Ser51Ser	138						0
13084	A	G	G	missense_variant	gacL	M5005_Spy0613	c.165A>G	p.Ile55Met	3						0
13108	G	A	A	synonymous_variant	gacL	M5005_Spy0613	c.189G>A	p.Glu63Glu	6						0
13117	G	A	A	synonymous_variant	gacL	M5005_Spy0613	c.198G>A	p.Thr66Thr	18						0
13118	A	C	C	missense_variant	gacL	M5005_Spy0613	c.199A>C	p.Ile67Leu	10						0
13127	A	C	C	missense_variant	gacL	M5005_Spy0613	c.208A>C	p.Ile70Leu	3						0
13133	G	A	A	missense_variant	gacL	M5005_Spy0613	c.214G>A	p.Val72Ile	6						0
13134	T	C	C	missense_variant	gacL	M5005_Spy0613	c.215T>C	p.Val72Ala	127						0
13141	A	G	G	synonymous_variant	gacL	M5005_Spy0613	c.222A>G	p.Gly74Gly	10						0
13143	C	T	T	missense_variant	gacL	M5005_Spy0613	c.224C>T	p.Thr75Met	1						0
13145	A	C,G	C	missense_variant	gacL	M5005_Spy0613	c.226A>C	p.Ile76Leu	403	G	missense_variant	gacL	c.226A>G	p.Ile76Val	19
13157	A	G	G	missense_variant	gacL	M5005_Spy0613	c.238A>G	p.Ile80Val	9						0
13165	T	C	C	synonymous_variant	gacL	M5005_Spy0613	c.246T>C	p.Pro82Pro	88						0
13180	T	G	G	synonymous_variant	gacL	M5005_Spy0613	c.261T>G	p.Pro87Pro	6						0
13189	T	C	C	synonymous_variant	gacL	M5005_Spy0613	c.270T>C	p.Tyr90Tyr	1						0
13249	C	T	T	synonymous_variant	gacL	M5005_Spy0613	c.330C>T	p.Asn110Asn	3						0
13282	G	A	A	synonymous_variant	gacL	M5005_Spy0613	c.363G>A	p.Lys121Lys	26						0
13288	T	C	C	synonymous_variant	gacL	M5005_Spy0613	c.369T>C	p.Ile123Ile	80						0
13309	T	C	C	synonymous_variant	gacL	M5005_Spy0613	c.390T>C	p.Phe130Phe	4						0
13310	A	C	C	missense_variant	gacL	M5005_Spy0613	c.391A>C	p.Ile131Leu	5						0
13319	A	G	G	missense_variant	gacL	M5005_Spy0613	c.400A>G	p.Asn134Asp	1						0
13327	A	T	T	missense_variant	gacL	M5005_Spy0613	c.408A>T	p.Lys136Asn	259						0
13329	C	T	T	missense_variant	gacL	M5005_Spy0613	c.410C>T	p.Ala137Val	30						0
13342	C	T	T	synonymous_variant	gacL	M5005_Spy0613	c.423C>T	p.Ser141Ser	31						0
13346	A	G	G	missense_variant	gacL	M5005_Spy0613	c.427A>G	p.Arg143Gly	5						0
13351	G	A	A	synonymous_variant	gacL	M5005_Spy0613	c.432G>A	p.Glu144Glu	2						0
13396	T	C	C	synonymous_variant	gacL	M5005_Spy0613	c.477T>C	p.Phe159Phe	4						0
13405	A	G	G	missense_variant	gacL	M5005_Spy0613	c.486A>G	p.Ile162Met	9						0
13436	G	A	A	missense_variant	gacL	M5005_Spy0613	c.517G>A	p.Ala173Thr	38						0
13437	C	T	T	missense_variant	gacL	M5005_Spy0613	c.518C>T	p.Ala173Val	9						0
13439	C	T	T	synonymous_variant	gacL	M5005_Spy0613	c.520C>T	p.Leu174Leu	3						0
13444	T	C	C	synonymous_variant	gacL	M5005_Spy0613	c.525T>C	p.Asp175Asp	2						0
13483	C	T	T	synonymous_variant	gacL	M5005_Spy0613	c.564C>T	p.Cys188Cys	1						0
13486	C	T	T	synonymous_variant	gacL	M5005_Spy0613	c.567C>T	p.Asn189Asn	1						0
13489	T	C	C	synonymous_variant	gacL	M5005_Spy0613	c.570T>C	p.Leu190Leu	4						0
13490	A	G	G	missense_variant	gacL	M5005_Spy0613	c.571A>G	p.Ile191Val	2						0
13495	C	T	T	synonymous_variant	gacL	M5005_Spy0613	c.576C>T	p.Ser192Ser	20						0
13504	G	A	A	missense_variant	gacL	M5005_Spy0613	c.585G>A	p.Met195Ile	6						0
13508	G	T,A	A	missense_variant	gacL	M5005_Spy0613	c.589G>A	p.Ala197Thr	33	T	missense_variant	gacL	c.589G>T	p.Ala197Ser	2
13520	A	G	G	missense_variant	gacL	M5005_Spy0613	c.601A>G	p.Ile201Val	8						0
13528	G	A	A	synonymous_variant	gacL	M5005_Spy0613	c.609G>A	p.Leu203Leu	28						0
13530	C	T	T	missense_variant	gacL	M5005_Spy0613	c.611C>T	p.Ser204Phe	1						0
13543	A	G	G	synonymous_variant	gacL	M5005_Spy0613	c.624A>G	p.Lys208Lys	16						0
13579	C	T	T	synonymous_variant	gacL	M5005_Spy0613	c.660C>T	p.Asn220Asn	2						0
13597	T	C	C	synonymous_variant	gacL	M5005_Spy0613	c.678T>C	p.Ser226Ser	13						0
13639	A	G	G	synonymous_variant	gacL	M5005_Spy0613	c.720A>G	p.Thr240Thr	1						0
13647	T	G	G	missense_variant	gacL	M5005_Spy0613	c.728T>G	p.Leu243Trp	1						0
13665	C	T	T	missense_variant	gacL	M5005_Spy0613	c.746C>T	p.Ser249Leu	3						0

13668	C	T	T	missense_variant	gacL	M5005_Spy0613	c.749C>T	p.Ser250Leu	34						0
13670	A	G	G	missense_variant	gacL	M5005_Spy0613	c.751A>G	p.Lys251Glu	33						0
13686	A	G	G	missense_variant	gacL	M5005_Spy0613	c.767A>G	p.Tyr256Cys	72						0
13689	A	G	G	missense_variant	gacL	M5005_Spy0613	c.770A>G	p.Asn257Ser	2						0
13691	A	G	G	missense_variant	gacL	M5005_Spy0613	c.772A>G	p.Thr258Ala	1						0
13693	C	T	T	synonymous_variant	gacL	M5005_Spy0613	c.774C>T	p.Thr258Thr	362						0
13702	C	T	T	synonymous_variant	gacL	M5005_Spy0613	c.783C>T	p.Phe261Phe	2						0
13711	A	G	G	synonymous_variant	gacL	M5005_Spy0613	c.792A>G	p.Leu264Leu	7						0
13715	C	T	T	synonymous_variant	gacL	M5005_Spy0613	c.796C>T	p.Leu266Leu	1						0
13717	A	G	G	synonymous_variant	gacL	M5005_Spy0613	c.798A>G	p.Leu266Leu	4						0
13727	C	T	T	synonymous_variant	gacL	M5005_Spy0613	c.808C>T	p.Leu270Leu	61						0
13730	G	A	A	missense_variant	gacL	M5005_Spy0613	c.811G>A	p.Val271Ile	1						0
13761	T	C	C	missense_variant	gacL	M5005_Spy0613	c.842T>C	p.Val281Ala	12						0
13764	G	A	A	missense_variant	gacL	M5005_Spy0613	c.845G>A	p.Cys282Tyr	2						0
13768	T	C	C	synonymous_variant	gacL	M5005_Spy0613	c.849T>C	p.Leu283Leu	144						0
13770	C	T	T	missense_variant	gacL	M5005_Spy0613	c.851C>T	p.Pro284Leu	1						0
13787	G	A	A	missense_variant	gacL	M5005_Spy0613	c.868G>A	p.Glu290Lys	25						0
13791	A	G	G	missense_variant	gacL	M5005_Spy0613	c.872A>G	p.Lys291Arg	1						0
13793	T	A	A	missense_variant	gacL	M5005_Spy0613	c.874T>A	p.Phe292Ile	1						0
13795	C	T,G	G	missense_variant	gacL	M5005_Spy0613	c.876C>G	p.Phe292Leu	3	T	synonymous_variant	gacL	c.876C>T	p.Phe292Phe	21
13806	C	T	T	missense_variant	gacL	M5005_Spy0613	c.887C>T	p.Thr296Ile	7						0
13822	G	A	A	synonymous_variant	gacL	M5005_Spy0613	c.903G>A	p.Leu301Leu	31						0
13838	C	T	T	synonymous_variant	gacL	M5005_Spy0613	c.919C>T	p.Leu307Leu	8						0
13840	A	G	G	synonymous_variant	gacL	M5005_Spy0613	c.921A>G	p.Leu307Leu	1						0
13882	T	C	C	synonymous_variant	gacL	M5005_Spy0613	c.963T>C	p.Tyr321Tyr	35						0
13904	G	A	A	missense_variant	gacL	M5005_Spy0613	c.985G>A	p.Val329Ile	3						0
13989	T	C	C	missense_variant	gacL	M5005_Spy0613	c.1070T>C	p.Ile357Thr	2						0
13993	A	G	G	synonymous_variant	gacL	M5005_Spy0613	c.1074A>G	p.Arg358Arg	22						0
13996	T	C	C	synonymous_variant	gacL	M5005_Spy0613	c.1077T>C	p.His359His	24						0
14010	T	A	A	missense_variant	gacL	M5005_Spy0613	c.1091T>A	p.Leu364Gln	1						0
14014	T	C	C	synonymous_variant	gacL	M5005_Spy0613	c.1095T>C	p.Ile365Ile	32						0
14026	T	C	C	synonymous_variant	gacL	M5005_Spy0613	c.1107T>C	p.Asn369Asn	18						0
14029	T	A	A	synonymous_variant	gacL	M5005_Spy0613	c.1110T>A	p.Ile370Ile	22						0
14035	G	A	A	synonymous_variant	gacL	M5005_Spy0613	c.1116G>A	p.Gln372Gln	1						0
14036	T	G	G	missense_variant	gacL	M5005_Spy0613	c.1117T>G	p.Phe373Val	1						0
14041	C	T	T	synonymous_variant	gacL	M5005_Spy0613	c.1122C>T	p.Gly374Gly	41						0
14056	T	C	C	synonymous_variant	gacL	M5005_Spy0613	c.1137T>C	p.Gly379Gly	281						0
14065	C	T	T	synonymous_variant	gacL	M5005_Spy0613	c.1146C>T	p.Asn382Asn	6						0
14066	C	A	A	missense_variant	gacL	M5005_Spy0613	c.1147C>A	p.Leu383Ile	2						0
14068	T	A	A	synonymous_variant	gacL	M5005_Spy0613	c.1149T>A	p.Leu383Leu	418						0
14072	C	T	T	missense_variant	gacL	M5005_Spy0613	c.1153C>T	p.Pro385Ser	1						0
14074	G	C	C	synonymous_variant	gacL	M5005_Spy0613	c.1155G>C	p.Pro385Pro	17						0
14085	G	C	C	missense_variant	gacL	M5005_Spy0613	c.1166G>C	p.Cys389Ser	16						0
14089	C	T	T	synonymous_variant	gacL	M5005_Spy0613	c.1170C>T	p.Phe390Phe	1						0
14099	G	A	A	missense_variant	gacL	M5005_Spy0613	c.1180G>A	p.Val394Ile	2						0
14106	T	A	A	missense_variant	gacL	M5005_Spy0613	c.1187T>A	p.Ile396Asn	1						0
14114	G	A	A	missense_variant	gacL	M5005_Spy0613	c.1195G>A	p.Ala399Thr	7						0
14118	G	C	C	missense_variant	gacL	M5005_Spy0613	c.1199G>C	p.Ser400Thr	240						0
14128	T	C	C	synonymous_variant	gacL	M5005_Spy0613	c.1209T>C	p.Thr403Thr	1						0
14133	A	G	G	missense_variant	gacL	M5005_Spy0613	c.1214A>G	p.Asn405Ser	16						0
14137	T	C	C	synonymous_variant	gacL	M5005_Spy0613	c.1218T>C	p.Ile406Ile	3						0
14138	G	T	T	missense_variant	gacL	M5005_Spy0613	c.1219G>T	p.Val407Leu	1						0
14147	A	C	C	missense_variant	gacL	M5005_Spy0613	c.1228A>C	p.Met410Leu	45						0
14158	C	T	T	synonymous_variant	gacL	M5005_Spy0613	c.1239C>T	p.Ile413Ile	1						0
14162	G	A	A	missense_variant	gacL	M5005_Spy0613	c.1243G>A	p.Val415Ile	1						0
14182	T	C	C	synonymous_variant	gacL	M5005_Spy0613	c.1263T>C	p.Gly421Gly	3						0
14204	C	T,A	A	missense_variant	gacL	M5005_Spy0613	c.1285C>A	p.Leu429Met	2	T	synonymous_variant	gacL	c.1285C>T	p.Leu429Leu	1
14215	A	G	G	synonymous_variant	gacL	M5005_Spy0613	c.1296A>G	p.Thr432Thr	8						0
14224	G	C	C	synonymous_variant	gacL	M5005_Spy0613	c.1305G>C	p.Gly435Gly	5						0
14226	C	T	T	missense_variant	gacL	M5005_Spy0613	c.1307C>T	p.Ser436Phe	2						0
14234	G	A	A	missense_variant	gacL	M5005_Spy0613	c.1315G>A	p.Val439Ile	1						0
14236	C	A,T	A	synonymous_variant	gacL	M5005_Spy0613	c.1317C>A	p.Val439Val	283	T	synonymous_variant	gacL	c.1317C>T	p.Val439Val	1

14261	A	G	G	missense_variant	gacL	M5005_Spy0613	c.1342A>G	p.Ile448Val	26						0
14266	A	G	G	synonymous_variant	gacL	M5005_Spy0613	c.1347A>G	p.Gly449Gly	3						0
14272	C	T	T	synonymous_variant	gacL	M5005_Spy0613	c.1353C>T	p.Ile451Ile	14						0
14273	C	A	A	missense_variant	gacL	M5005_Spy0613	c.1354C>A	p.Pro452Thr	5						0
14278	A	G	G	synonymous_variant	gacL	M5005_Spy0613	c.1359A>G	p.Leu453Leu	39						0
14284	G	T,A	T	missense_variant	gacL	M5005_Spy0613	c.1365G>T	p.Leu455Phe	51	A	synonymous_variant	gacL	c.1365G>A	p.Leu455Leu	1
14307	A	G	G	missense_variant	gacL	M5005_Spy0613	c.1388A>G	p.Gln463Arg	1						0
14319	A	C	C	missense_variant	gacL	M5005_Spy0613	c.1400A>C	p.Gln467Pro	1						0
14327	G	A	A	missense_variant	gacL	M5005_Spy0613	c.1408G>A	p.Asp470Asn	1						0
14328	A	G,C	C	missense_variant	gacL	M5005_Spy0613	c.1409A>C	p.Asp470Ala	1	G	missense_variant	gacL	c.1409A>G	p.Asp470Gly	2
14332	C	A,T	A	synonymous_variant	gacL	M5005_Spy0613	c.1413C>A	p.Ile471Ile	10	T	synonymous_variant	gacL	c.1413C>T	p.Ile471Ile	1
14335	T	G	G	synonymous_variant	gacL	M5005_Spy0613	c.1416T>G	p.Leu472Leu	1						0
14347	G	A	A	synonymous_variant	gacL	M5005_Spy0613	c.1428G>A	p.Leu476Leu	2						0
14357	G	A	A	missense_variant	gacL	M5005_Spy0613	c.1438G>A	p.Val480Ile	278						0
14362	G	T	T	synonymous_variant	gacL	M5005_Spy0613	c.1443G>T	p.Ser481Ser	6						0
14363	C	T	T	missense_variant	gacL	M5005_Spy0613	c.1444C>T	p.Leu482Phe	2						0
14368	G	T	T	missense_variant	gacL	M5005_Spy0613	c.1449G>T	p.Leu483Phe	77						0
14377	T	C	C	synonymous_variant	gacL	M5005_Spy0613	c.1458T>C	p.Leu486Leu	1947						0
14387	A	G	G	missense_variant	gacL	M5005_Spy0613	c.1468A>G	p.Met490Val	1						0
14407	T	A,C	A	stop_gained	gacL	M5005_Spy0613	c.1488T>A	p.Tyr496*	1	C	synonymous_variant	gacL	c.1488T>C	p.Tyr496Tyr	1
